# Copper(i)-catalyzed sulfonylative Suzuki–Miyaura cross-coupling†
†Electronic supplementary information (ESI) available. See DOI: 10.1039/c6sc05483h


**DOI:** 10.1039/c6sc05483h

**Published:** 2017-02-28

**Authors:** Yiding Chen, Michael C. Willis

**Affiliations:** a Department of Chemistry , University of Oxford , Chemical Research Laboratory , Mansfield Road , Oxford , OX1 3TA , UK . Email: michael.willis@chem.ox.ac.uk

## Abstract

Using a simple copper(i) catalyst has allowed a high yielding sulfonylative-Suzuki–Miyaura cross-coupling reaction to be developed. The process provides a single step route to diaryl sulfones from the direct combination of aryl boronic acids, sulfur dioxide and aryl iodides, and represents the first sulfonylative variant of a classic cross-coupling reaction. Sulfur dioxide is delivered from the surrogate reagent, DABSO. Variation of the reaction conditions allowed interruption of the sulfonylative-Suzuki coupling, resulting in the formation of a presumed Cu–sulfinate intermediate. These sulfinates could be trapped as their sodium salts and treated with electrophiles to allow access to arylalkyl sulfones, β-hydroxyl sulfones, sulfonamides and sulfonyl fluorides.

## Abstract 2

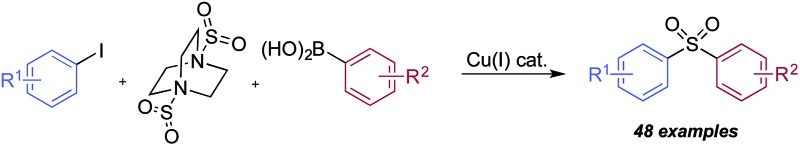
A sulfonylative variant of a “classic” cross-coupling, with a broad substrate scope. The electrophilic trapping of a sulfinate intermediate was also possible.

## Introduction

**Fig. 1:**
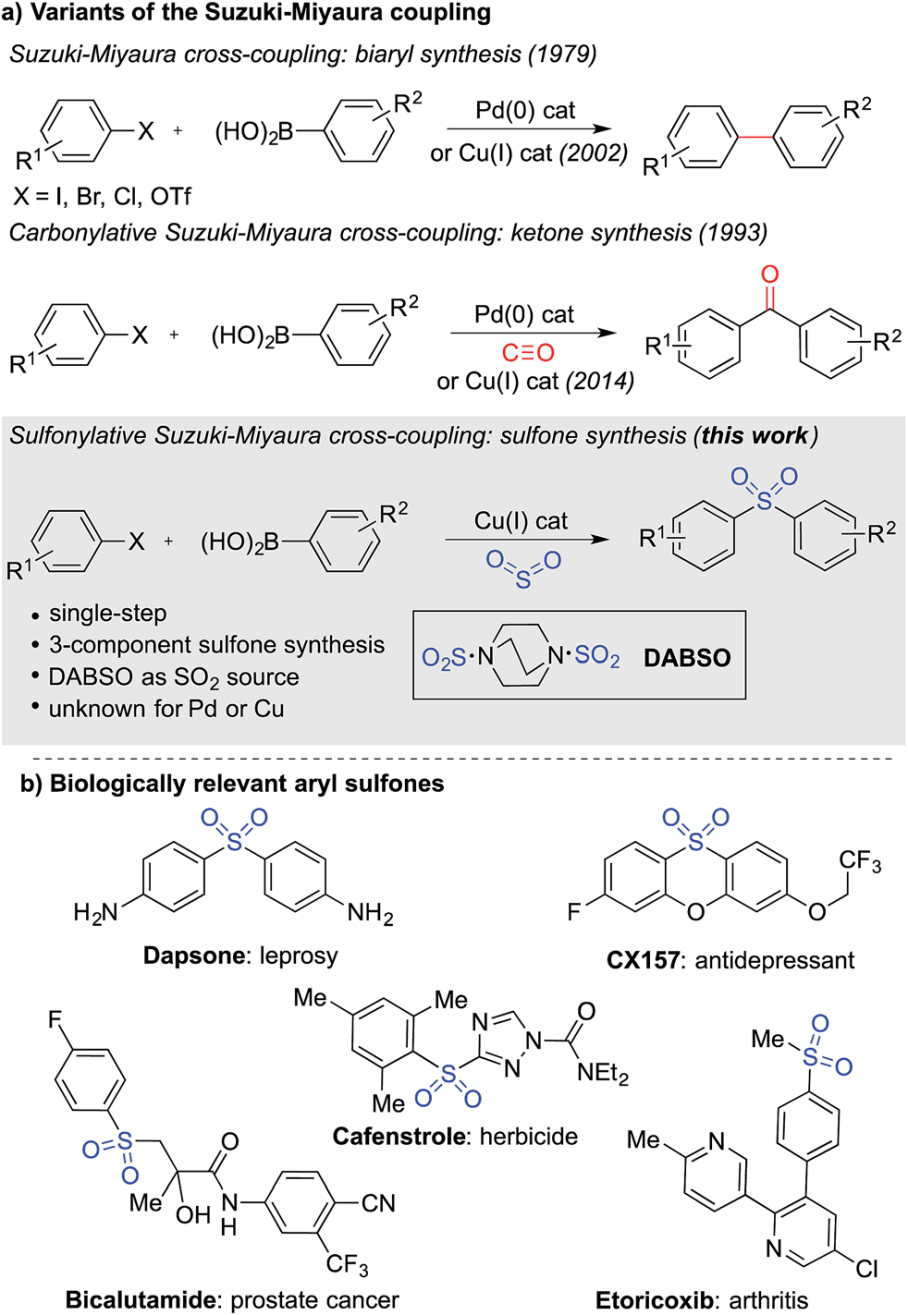
(a) Variants of the Suzuki–Miyaura cross-coupling reaction, and (b) examples of biologically relevant sulfones.

The Suzuki–Miyaura cross-coupling reaction^1^ is the preeminent method for forming carbon–carbon bonds in the pharmaceutical industry.^2^ This classic reaction combines aryl boronates and boronic acids with aryl halides under the action of a palladium catalyst, delivering biaryl products in an efficient manner. The popularity of this method stems from the wide availability and low-toxicity of boron-derived substrates, mild reaction conditions, associated good functional group tolerance, and general robustness of the transformations. Carbonylative versions of these reactions have also been developed,^3^ in which a molecule of carbon monoxide is incorporated in the coupling reaction, diverting the process from biaryl formation and resulting in a ketone product.^4^ However, despite the electronic similarity between carbon monoxide and sulfur dioxide,^5^ a sulfonylative variant, which would provide valuable sulfone products, has not been realized (Fig. 1a). When the utility of the sulfone functional group is considered – they feature in numerous biologically active molecules, including many marketed pharmaceuticals and agrochemicals (Fig. 1b), are common motifs in designed materials, and they serve as versatile intermediates for organic synthesis – this absence is striking.^6^ In this Edge Article, we show that by using a copper(i) catalyst, a high yielding sulfonylative-Suzuki–Miyaura cross-coupling reaction is possible.

## Results and discussion

A variety of methods are available for the synthesis of sulfones,^6,7^ including a number of transition metal-catalyzed approaches.^8^ However, many of these classical syntheses are either multi-step, or employ harmful, non-selective reagents, significantly detracting from their utility. Recent years have seen catalytic methods for sulfur dioxide incorporation^9^ emerge as useful tools for the preparation of sulfonyl-derived functional groups.^10^ In particular, palladium-catalyzed aryl sulfinate formation has been reported using both aryl halides^11^ and aryl boronic acids^12^ as the starting aryl substrates. The sulfinates formed in these reactions can be converted to sulfones, usually by employing a simple electrophilic trap, but this is typically achieved in the second step of the overall reaction.^12*b*^ Separately, aryl sulfinates have been employed as nucleophiles in a number of palladium-catalyzed coupling reactions with aryl halides.^8f,13^ We postulated that the integration of these two separate palladium-catalyzed transformations into a single reaction would provide a convenient route for the formation of diarylsulfones, while maintaining many of the desirable features of the classic Suzuki–Miyaura cross-coupling reaction. Despite the encouraging precedents for the separate steps of the proposed coupling, we were never able to achieve the transformation using palladium catalysis. A possible complicating factor with these reactions is the known palladium-catalyzed desulfinative coupling of aryl sulfinates and aryl halides.^14^

Despite this set back, we were still drawn to the advantages that a sulfonylative Suzuki–Miyaura coupling would provide, in particular, the ability to prepare sulfones from the combination of aryl halides and aryl boronic acids – arguably the two coupling partners of choice for synthetic chemists – would provide a conceptually simple, direct route to these valuable products. We turned our attention to the use of alternative catalysts, and were aware that copper(i) catalysts have also been reported to promote the coupling of aryl sulfinates with aryl halides.^15,16^ Although copper-catalyzed aryl sulfinate formation using sulfur dioxide has also been reported, only the less-available aryl triethoxysilanes can be used as substrates,^17^ with the use of boronic acids as substrates being unknown.^18^ Despite this crucial step being unprecedented, we were attracted to the cost-of-goods and sustainability advantages that are potentially available with copper-catalyzed transformations,^19^ relative to those using palladium, and elected to explore the development of a copper(i)-catalyzed process. Indeed, these advantages have also been recognized in the development of Cu(i)-catalyzed variants of the classic Suzuki–Miyaura cross-coupling reaction,^20^ as well as a carbonylative version.^21^

We began our investigation by exploring the coupling of 4-iodotoluene **1a**, phenyl boronic acid **2a**, and sulfur dioxide. To avoid the direct use of gaseous sulfur dioxide, we employed a surrogate reagent,^22^ the bis-adduct of sulfur dioxide combined with DABCO, DABSO, which is a bench stable colorless solid and is commercially available.^23^ Pleasingly, after a variety of copper(i) salts, ligands, solvents, reaction temperatures and additives were explored, we were able to identify a set of optimal conditions, and these are shown in Table 1. The ESI† provides full details, however, the key variations are noted in Table 1. The use of an electron-rich bipyridine ligand, **L4**,^24^ in combination with the copper salt Cu(MeCN)_4_BF_4_ and the polar aprotic solvent DMPU, allowed an efficient transformation to be achieved.

**Table 1:** Selected optimization data for the formation of sulfone **3a** from the coupling of aryl iodide **1a** and boronic acid **2a** ^*a*^

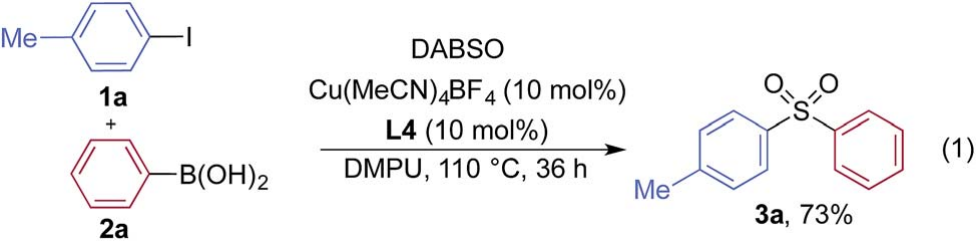		
Entry	Variation from eqn (1)	Yield
1	Cs_2_CO_3_ added (2.0 equiv.)	16%
2	No Cu, no ligand	0%
3	No ligand	25%
4	**L1** (10%)	40%
5	**L2** (10%)	47%
6	**L3** (10%)	44%
7	DMF as solvent	49%
8	130 °C	70%
9	24 h	60%
10	O_2_ atmosphere	3%
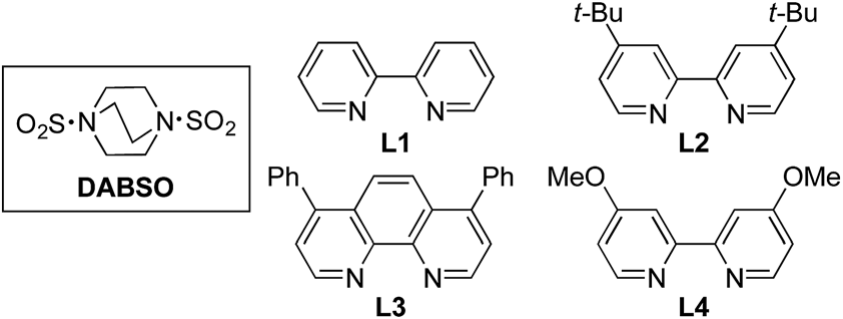		

^*a*^Reaction conditions: **1a** (0.2 mmol, 1.0 equiv.), DABSO (0.3 mmol, 1.5 equiv.), **2a** (0.6 mmol, 3.0 equiv.), solvent (1.0 mL). Yields were measured by HPLC using an internal standard.

We then used the optimized reaction conditions to explore the scope of the reaction with respect to the variation of the aryl iodide coupling partner, while maintaining phenyl boronic acid as the second aryl component (Table 2). Generally, the process was tolerant of a broad range of both electron-donating and electron-withdrawing functional groups, located at all positions on the aromatic ring. The notable examples include product **3g**, which features sulfur atoms with two different oxidation states, an arrangement that would be challenging to prepare using standard sulfone methodology, and sulfone products **3i** and **3j** which contain free primary amine and phenol groups, respectively. Reactive functional groups such as aldehyde (**3n**), ketone (**3k**) and nitrile (**3l**) were also incorporated without incident. Heterocycle-derived iodides could be transformed to sulfones, with indole (**3t**), quinoline (**3u**), pyridine (**3v**) and pyrazole (**3w**) derived products being obtained. The products **3x** and **3y** demonstrate that alkenyl iodide substrates can also be employed. These scoping experiments were routinely performed on a 0.2 mmol scale, however, larger scale reactions were also possible. For example, sulfone **3e** was synthesised on a semi-preparative 1 gram scale (5 mmol).

**Table 2:** Variation of the aryl iodide coupling partner in the copper-catalyzed sulfonylative Suzuki–Miyaura reaction^*a*^

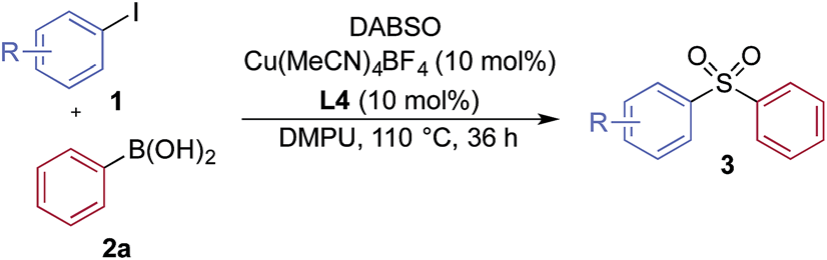
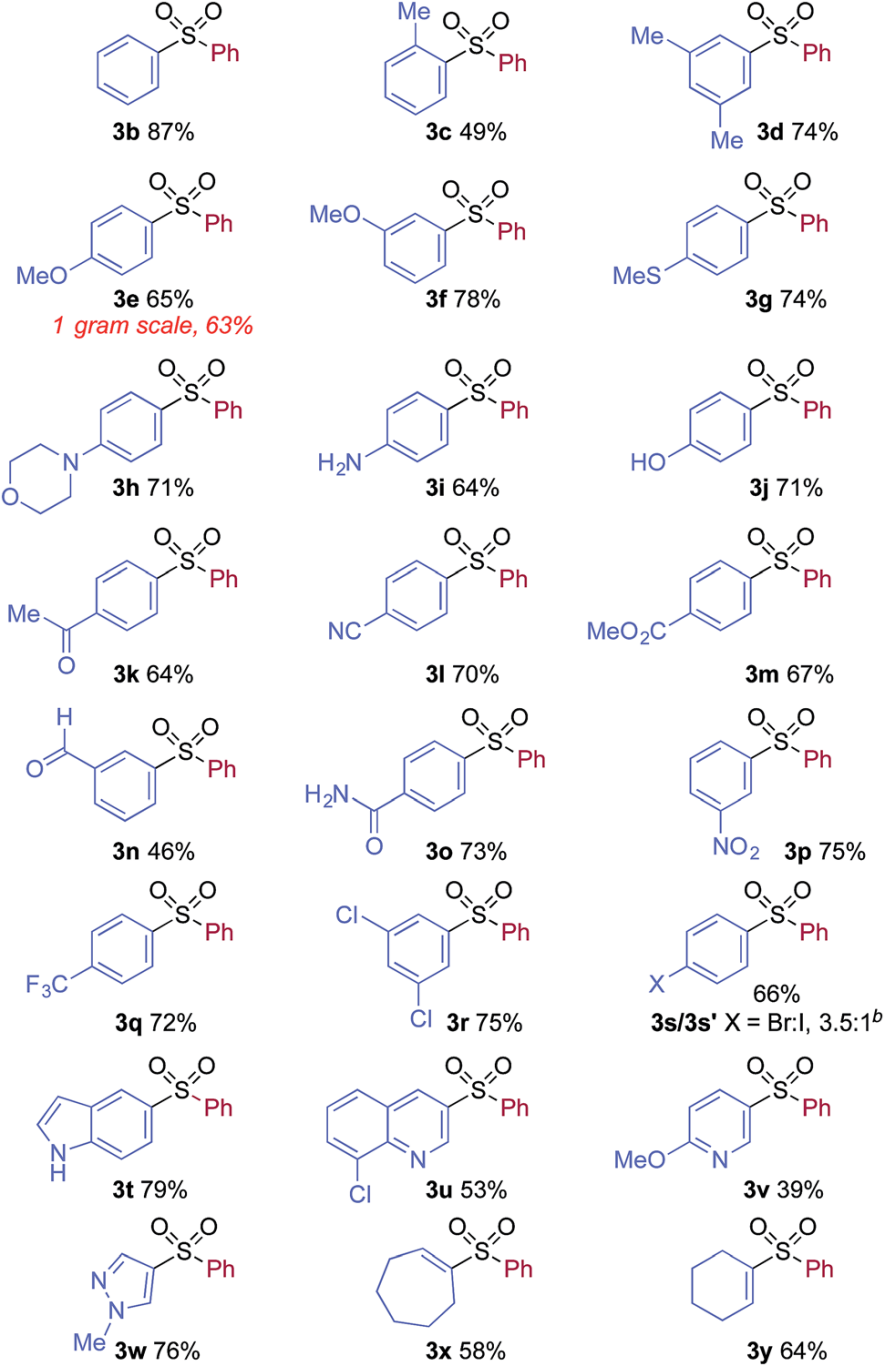

^*a*^Reaction conditions: aryl iodide **1** (0.2 mmol, 1.0 equiv.), DABSO (0.3 mmol, 1.5 equiv.), phenyl boronic acid **2a** (0.6 mmol, 3.0 equiv.), Cu(MeCN)_4_BF_4_ (10 mol%), **L4** (10 mol%), DMPU (1.0 mL), 110 °C, 36 h. Isolated yields.

^*b*^Using 4-bromo-iodobenzene as substrate.

A significant variation of the boronic acid coupling partner was also possible (Table 3). Either 4-iodotoluene or 4-morpholino-iodobenzene was employed as the coupling partner in these transformations, with the choice being dictated by the ease of product purification. A broad range of useful functionalities could be introduced using this chemistry, for example, ether (**4d**), sulfide (**4e**), alcohol (**4f**), amine (**4g**), carbamate (**4h**), amide (**4i**), aryl chloride (**4l,n,o**), silane (**4m**), and aldehyde (**4q**) groups were all tolerated under the reaction conditions. As with the iodide component, heterocyclic variants (**4r,s**) and alkenyl boronic acids were also useful substrates, with the latter delivering unsaturated sulfone products (**4t,u**).

**Table 3:** Variation of the aryl boronic acid coupling partner in the copper-catalyzed sulfonylative Suzuki–Miyaura reaction^*a*^

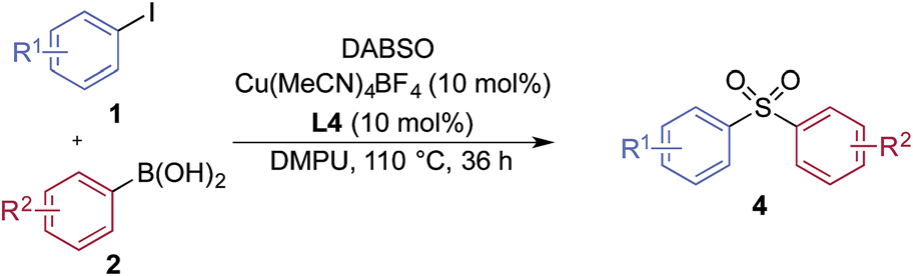
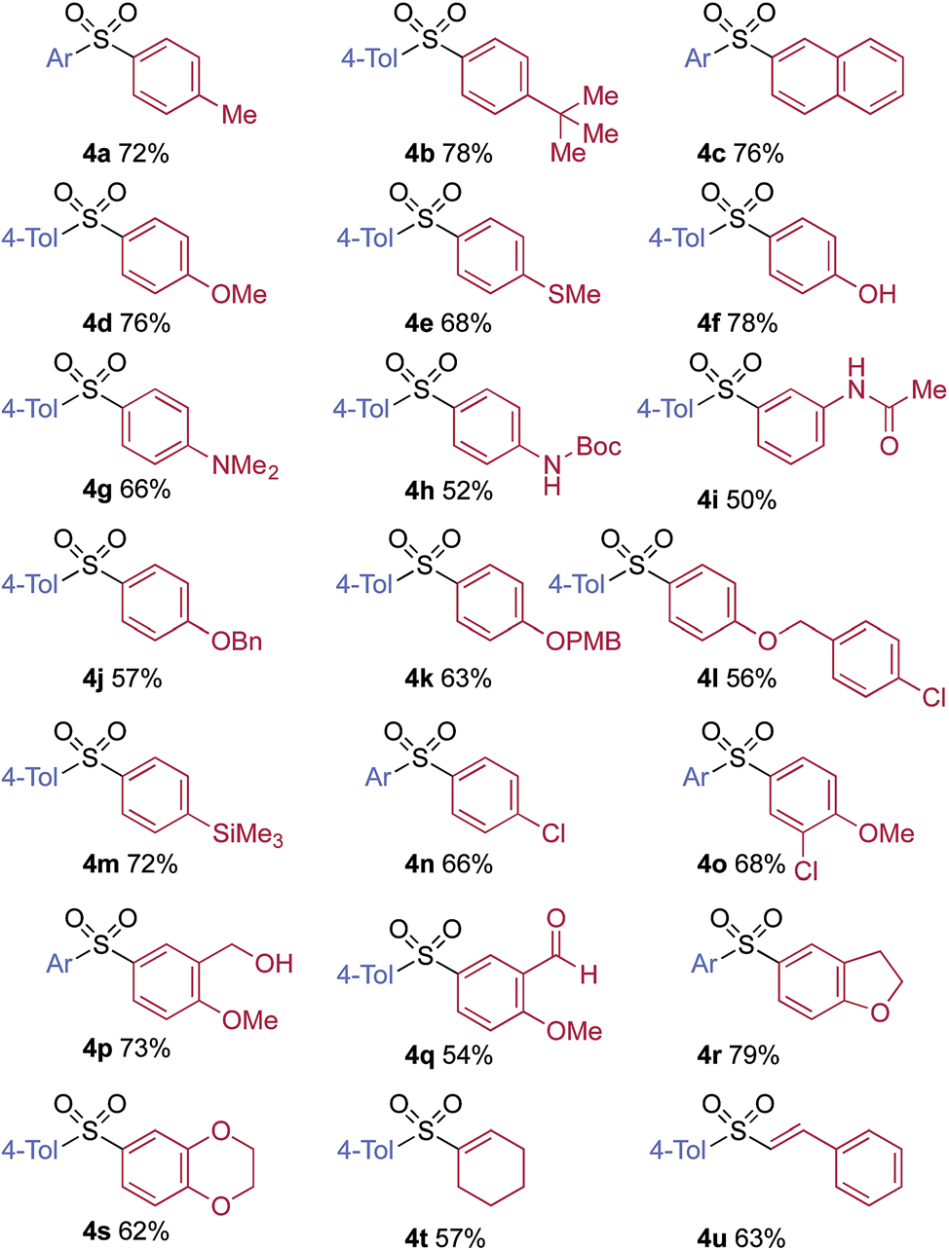

^*a*^Ar = 4-morpholino–C_6_H_4_. Reaction conditions: aryl iodide **1** (0.2 mmol, 1.0 equiv.), DABSO (0.3 mmol, 1.5 equiv.), boronic acid **2** (0.6 mmol, 3.0 equiv.), Cu(MeCN)_4_BF_4_ (10 mol%), **L4** (10 mol%), DMPU (1.0 mL), 110 °C, 36 h. Isolated yields.

One of the key challenges in developing a direct copper-catalyzed sulfonylative Suzuki–Miyaura reaction was establishing the viability of the copper-catalyzed sulfinate formation from the combination of an aryl boronic acid and sulfur dioxide. Although the broad scope of the targeted reaction, as shown in Tables 2 and 3, goes a significant way towards validating this hypothesis, we wanted to develop reaction conditions that would allow the copper-catalyzed synthesis of discrete sulfinate intermediates. This would then allow these intermediates to be trapped with a variety of electrophiles to deliver structurally diverse products, and would complement the recently described palladium-catalyzed variant of this transformation.^12^ By employing a stoichiometric amount of NaBF_4_ in combination with Cu(MeCN)_4_BF_4_ as a catalyst, we were able to interrupt the sulfonylative Suzuki coupling and realize an efficient method for the preparation of copper sulfinates from aryl boronic acids (Table 4). Metal sulfinates are not useful products in their own right, and are in fact challenging to isolate, however, they can be converted to a selection of useful sulfonyl-derived functional groups by treatment with appropriate electrophiles. For example, *in situ* treatment of the sulfinate with an alkyl bromide delivers alkylaryl sulfone products (**6a–e**).^25^ The use of epoxides as the electrophiles provides β-hydroxy sulfones (**6f–j**).^25*b*^ Treatment with *in situ* generated chloroamines provides sulfonamides (**6k–o**),^26^ and treatment with NFSI, which is a source of electrophilic fluorine, generates the corresponding sulfonyl fluorides (**6p–t**).^27^

**Table 4:** Copper-catalyzed sulfinate formation and onwards conversion to alkylaryl sulfones, β-hydroxy sulfones, sulfonamides and sulfonyl fluorides^*a*^

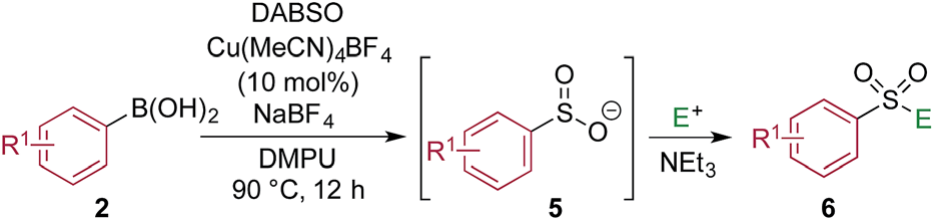
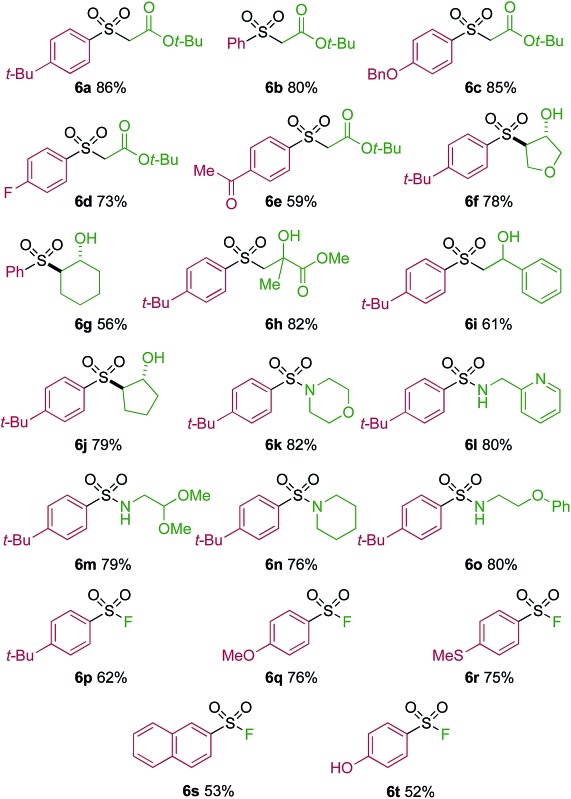

^*a*^Reaction conditions DABSO (0.1 mmol, 0.5 equiv.), boronic acid **2** (0.2 mmol, 1.0 equiv.), NaBF_4_ (1.0 mmol, 5.0 equiv.), DMPU (1 mL), 90 °C, 12 h; then, for **6a–6e**: Et_3_N (0.3 mmol, 1.5 equiv.), *t*-butyl-bromoacetate (0.4 mmol, 2.0 equiv.); for **6f–6j**: Et_3_N (0.3 mmol, 1.5 equiv.), epoxide (0.4 mmol, 2.0 equiv.), H_2_O (2 mL); for **6k–6o**: Et_3_N (0.3 mmol, 1.5 equiv.), amine (0.4 mmol, 2.0 equiv.), NaOCl (0.4 mmol, 2% aq. solution, 2.0 equiv.); for **6p–6t**: NFSI (0.3 mmol, 1.5 equiv.), DMPU (0.2 mL). Isolated yields.

## Conclusions

In realizing a copper-catalyzed sulfonylative Suzuki–Miyaura cross-coupling, a work-horse reaction of the pharmaceutical industry has been transformed to deliver valuable sulfone products. This process represents the first true sulfonylative-variant of a classic cross-coupling process. A good variation of both coupling partners is possible, delivering sulfone products in high yields. The demonstration that the combination of copper(i) catalysts, aryl boronic acids and a sulfur dioxide surrogate generates aryl sulfinate intermediates is also significant, and bodes well for the development of new reactions which exploit these key intermediates, access to which has been limited when using benign, readily available substrates and sustainable catalysts.
