# Supplementary material for: Copper(i)-catalyzed sulfonylative Suzuki–Miyaura cross-coupling
Source: Chem Sci. 2017 Feb 28;8(4):3249–53. doi: 10.1039/c6sc05483h (PMC5424447; doi:10.1039/c6sc05483h)
Supplement: Supplementary file 1 [file SC-008-C6SC05483H-s001.pdf]

Electronic Supplementary Material (ESI) for Chemical Science.
This journal is © The Royal Society of Chemistry 2017

Copper-catalysed sulfonylative Suzuki-Miyaura cross-coupling

Yiding Chen and Michael C. Willis*

Department of Chemistry, University of Oxford, Chemistry Research Laboratory, Mansfield Road, Oxford, OX1 3TA, United Kingdom.

Supporting Information

Content

1. General Information: 2
2. Optimisation on copper(I) catalysed biarylsulfone synthesis 3
   1.1 Catalyst screening 3
   1.2 Ligand screening 4
   1.3 Solvent screening 6
3. Synthesis of biarylsulfones 7
   GENERAL PROCEDURE A for the synthesis of biarylsulfones: 7
4. Synthesis of *tert*-butyl 2-(arylsulfonyl)acetate 31
   GENERAL PROCEDURE B for the synthesis of *tert*-butyl 2-(arylsulfonyl)acetate 31
5. Synthesis of *β*-hydroxysulfones 34
   GENERAL PROCEDURE C for the synthesis of *β*-hydroxysulfones 34
6. Synthesis of sulfonamides 37
   GENERAL PROCEDURE D for the synthesis of sulfonamides 37
7. Synthesis of sulfonyl fluorides 40
   GENERAL PROCEDURE E for the synthesis of sulfonyl fluorides 40
8. References: 42
9. ^{1}H NMR and ^{13}C NHR spectra 43

1. General Information:

All procedures below were conducted under inert nitrogen atmosphere unless stated otherwise. Reagents were purchased from Sigma-Aldrich, Alfa Aesar, Acros and Fluorochem and were used as supplied unless stated otherwise. 1,2-Dichloroethane and 1,4-dioxane were distilled from CaH_2. All dry solvents i.e. THF, MeOH, MeCN and toluene were dried over 4 Å molecular sieves and through anhydrous alumina columns using an Innovative Technology Inc. PS-400-7 solvent purification system. Other solvents, i.e. sulfolane, DMF, DMA, DMSO, DMI (1,3-dimethyl-2-imidazolidinone), DMPU (1,3-dimethyl-3,4,5,6-tetrahydro-2(1*H*)-pyrimidinone) and work-up solvents, were employed directly from commercial sources, i.e. Sigma-Aldrich unless stated otherwise. Petroleum ether refers to the fractions of petrol collected between 40-60 °C b.p.

Reactions were monitored *via* thin layer chromatography (TLC) on pre-coated aluminium plates (Merck Kieselgel 60 F_{254}). Products were visualized by UV light (254 nm) and/or with $KMnO_4$ stain. Flash column chromatography was conducted using silica gel 60 (Geduran Si 60, 40-63 µm) with head pressure from nitrogen tap.

^{1}H NMR, ^{13}C NMR and ^{19}F NMR data were obtained from a Bruker Avance AV 500 or a Bruker Avance AV 400 NMR spectrometer. Chemical shifts (δ) are referenced to the residual solvent as $CDCl_3$ or DMSO-d_6 in the unit of parts per million (ppm). Coupling constants *J* are quoted in the unit of hertz (Hz). Proton and carbon multiplicity is recorded as singlet (s), doublet (d), triplet (t), quartet (q), multiplet (m) and broad (br). All compounds examined were dried *in vacuo* to remove residual solvents. Determination of inseparable compounds were carried out on 500 MHz ^{1}H NMR, 125 MHz ^{13}C NMR spectra.

Low resolution mass spectra (LRMS) were recorded on a Fisons Platform II spectrometer. High resolution mass spectrometry (HRMS) was performed on a Bruker MicroTof spectrometer using electrospray ionization method (ESI) or on a Micromass LCT spectrometer using filed ionization method (FI) or electron ionization (CI).

Infra-red spectra were recorded neat on a Bruker Tensor 27 FT-IR spectrometer using a PIKE Miracle ATR module.

All compounds listed in the paper are >98% purity. Some sulfone products appear to be very hydroscopic therefore contain 0.2-0.5 mol equivalents of water (2-5 wt%) present in the ^{1}H NMR spectra as shown below.

2. Optimisation on copper(I) catalysed biarylsulfone synthesis

1.1 Catalyst screening

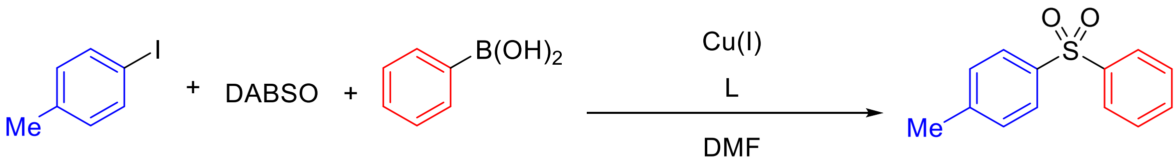

1a 2a 3a

Entry	Cu catalyst (mol%)	Ligand (mol%)	HPLC Yield of 3a (%)
1	CuBr (40%)	-	11
2	CuI (40%)	-	4
3	CuI (40%)	1,10-phenanthroline (40%)	6
4	CuOAc (40%)	-	13
5	Cu_2O (40%)	-	25
6	Cu_2O (10%)	1,10-phenanthroline (20%)	27
7	CuSCN (20%)	-	5
8	CuSCN (20%)	1,10-phenanthroline (20%)	3
9	$CuFe_2O_4$ (10%)	-	7
10	$CuFe_2O_4$ (10%)	1,10-phenanthroline (10%)	7
11	Cu(IPr) (10%)	-	Trace
12	Cu(IPr) (10%)	1,10-phenanthroline (20%)	Trace
13	Cu-thiophene-carboxylate (10%)	-	19
14	Cu-thiophene-carboxylate (10%)	1,10-phenanthroline (20%)	13
15	Cu-methylsalicylate (10%)	1,10-phenanthroline (20%)	9
16	$(CuOTf)_2PhH$ (10%)	-	26
17	$(CuOTf)_2PhH$ (10%)	1,10-phenanthroline (20%)	42
18	$CuCF_3Phen$ (10%)	-	37
**19**	**$Cu(MeCN)_4BF_4$ (10%)**	**-**	**23**
**20**	**$Cu(MeCN)_4BF_4$ (10%)**	**1,10-phenanthroline (10%)**	**32**

Reaction conditions: 1a (0.2 mmmol, 1.0 eqiv.), DABSO (0.3 mmol, 1.5 equiv.), 2a (0.6 mmol, 3.0 equiv.), DMF (1 mL), 110 °C, N_2, 14 h.

1.2 Ligand screening

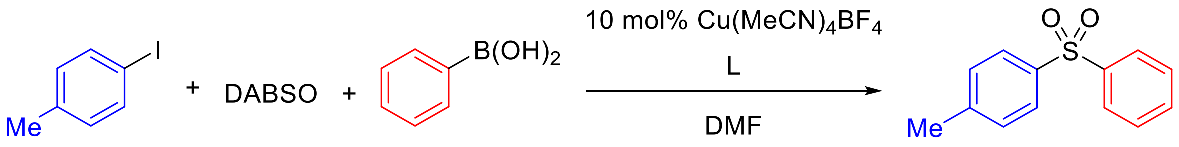

1a 2a 3a

L =

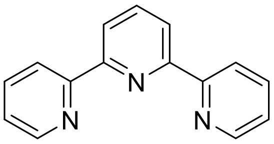

4% (20 mol%)

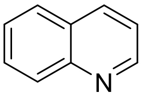

22% (20 mol%)

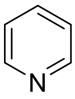

22% (20 mol%)

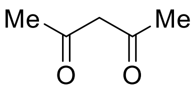

28% (20 mol%)

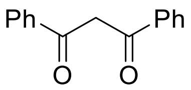

20% (20 mol%)

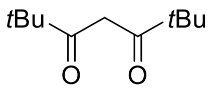

16% (20 mol%)

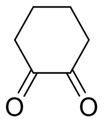

15% (20%)

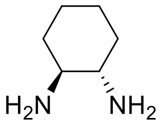

13% (20 mol%)

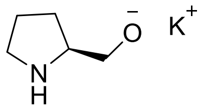

13% (20 mol%)

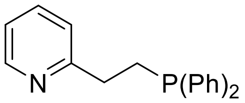

5% (20 mol%)

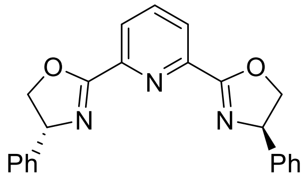

22% (20 mol%)

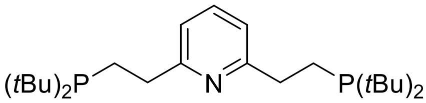

No Reaction (20 mol%)

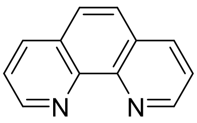

32% (20 mol%)

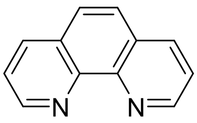

38% (10 mol%)

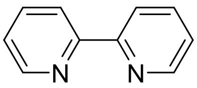

33% (20 mol%)

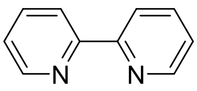

38% (10 mol%)

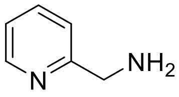

3% (10 mol%)

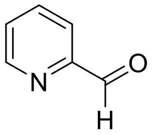

37% (10 mol%)

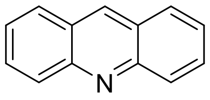

24% (20 mol%)

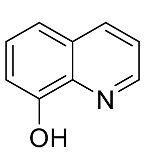

18% (10 mol%)

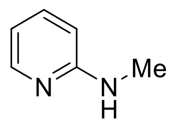

18% (10 mol%)

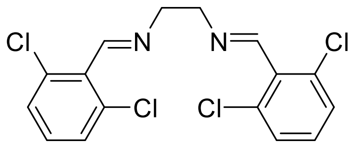

0% (10 mol%)

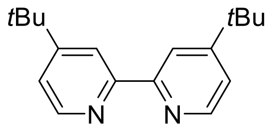

44% (10 mol%)

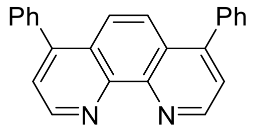

44% (10 mol%)

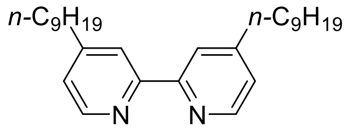

40% (10 mol%)

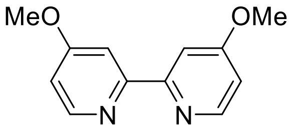

49% (10 mol%)

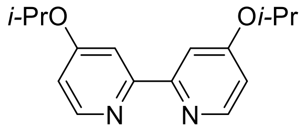

48% (10 mol%)

Reaction conditions: 1a (0.2 mmmol, 1.0 eqiv.), DABSO (0.3 mmol, 1.5 equiv.), 2a (0.6 mmol, 3.0 equiv.), $Cu(MeCN)_4BF_4$ (0.02 mmol, 10 mol%), DMF (1 mL), 110 °C, N_2, 14 h.

1.3 Solvent screening

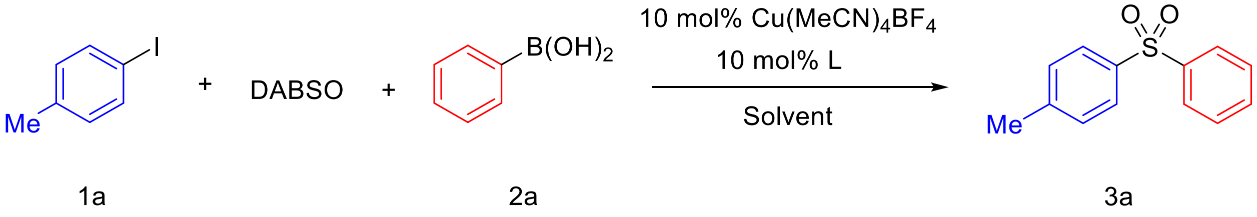

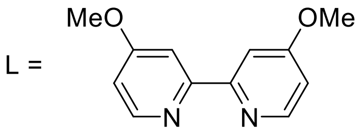

Entry	Solvent	HPLC Yield of 3a (%)
1	DMF	49
2	Dioxane	0
3	Dichloromethane	0
4	Toluene	0
5	*tert*-Butanol	37
6	Nitrobenzene	Trace
7	Benzonitrile	16
8	Nitromethane	18
9[a]	Sulfolane	60
10	NMP	55
11	DMSO	35
12	DMA	33
13	DMI	53
**14**	**DMPU**	**60**

[a] Product inseparable with the solvent.

Reaction conditions: 1a (0.2 mmol, 1.0 eqiv.), DABSO (0.3 mmol, 1.5 equiv.), 2a (0.6 mmol, 3.0 equiv.), $Cu(MeCN)_4BF_4$ (0.02 mmol, 10 mol%), ligand (0.02 mol, 10 mol%), solvent (1 mL), 110 °C, N_2, 14 h.

3. Synthesis of biarylsulfones:

GENERAL PROCEDURE A for the synthesis of biarylsulfones:

Phenyl *p*-tolyl sulfone 3a

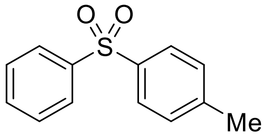

Phenylboronic acid (73 mg, 0.6 mmol, 3.0 eq.), $Cu(MeCN)_4BF_4$ (6.4 mg, 0.02 mmol, 10 mol%), DABSO (72 mg, 0.3 mmol, 1.5 eq.), 4,4'-dimethoxy-bipyridine (4.3 mg, 0.02 mmol, 10 mol%) and 4-iodotoluene (44 mg, 0.2 mmol, 1.0 eq.) were mixed and dissolved in DMPU (1 mL) under nitrogen. Aryl iodide was added *via* syringe if liquid at room temperature. The reaction mixture was placed in a pre-heated oil bath at 110 °C and stirred for 36 hours prior to cooling to room temperature. Water (10 mL) was then added, and the resultant mixture was extracted with Et_2O (3 × 10 mL). Combined organic phases were washed with brine (3 × 10 mL), dried over $MgSO_4$, filtered and concentrated *in vacuo*. The crude product was purified *via* flash column chromatography (25% Et_2O in petroleum ether) to give the title compound as a white solid (34 mg, 73%).

^{1}**H NMR** (400 MHz, $CDCl_3$) δ 7.86 (d, *J* = 7.2 Hz, 2H, H_{Ar}), 7.76 (d, *J* = 8.1 Hz, 2H, H_{Ar}), 7.50 – 7.45 (m, 1H, H_{Ar}), 7.45 – 7.39 (m, 2H, H_{Ar}), 7.23(d, *J* = 8.1 Hz, 2H, H_{Ar}), 2.32 (s, 3H, CH_3); ^{13}**C NMR** (100 MHz, $CDCl_3$) δ 144.2, 142, 138.6, 133.0, 129.9, 129.2, 127.7, 127.5, 21.6. **LRMS** (ESI, m/z) 233.0 ($[M+H]^+$, 100%). **HRMS** (ESI) calcd for $C_{13}H_{13}O_2S$ $[M+H]^+$ 233.0631, found 233.0634. **M.p.**: 123 – 125 °C (lit. 124 – 125 °C). The data recorded are consistent with the literature.[1]

Diphenyl sulfone 3b

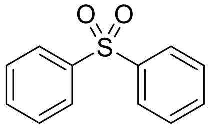

General procedure A was followed with phenylboronic acid (73 mg, 0.6 mmol, 3.0 eq.), $Cu(MeCN)_4BF_4$ (6.4 mg, 0.02 mmol, 10 mol%), DABSO (72 mg, 0.3 mmol, 1.5 eq.), 4,4'-dimethoxy-bipyridine (4.3 mg, 0.02 mmol, 10 mol%) and 4-iodobenzene (23 µL, 0.2 mmol, 1.0 eq.). The product was purified *via* flash column chromatography (20% Et_2O in petroleum ether) to give the titled product as a white solid (37 mg, 87%).

^{1}**H NMR** (400 MHz, $CDCl_3$) δ 7.92 – 7.85 (m, 4H, H_{Ar}), 7.52 – 7.48 (m, 2H, H_{Ar}), 7.44 – 7.39 (m, 4H, H_{Ar}); ^{13}**C NMR** (100 MHz, $CDCl_3$) δ 141.6, 133.2, 129.3, 127.7. **LRMS** (ESI, m/z) 217.1 ($[M-H]^-$, 100%); **HRMS** (ESI) calcd for $C_{12}H_{10}O_2SNa$ $[M+Na]^+$ 241.0294, found 241.0294. **M.p.**: 123 – 124 °C (lit. 122 – 124 °C). The data recorded are consistent with the literature.[2]

Phenyl *o*-tolyl sulfone 3c

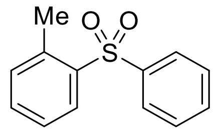

General procedure A was followed with phenylboronic acid (73 mg, 0.6 mmol, 3.0 eq.), $Cu(MeCN)_4BF_4$ (6.4 mg, 0.01 mmol, 10 mol%), DABSO (72 mg, 0.3 mmol, 1.5 eq.), 4,4'-dimethoxy-bipyridine (4.3 mg, 0.01 mmol, 10 mol%) and 2-iodotoluene (25 µL, 0.2 mmol, 1.0 eq.). The product was purified *via* flash column chromatography (15% Et_2O in petroleum ether) to give the titled product as a white solid (23 mg, 49%).

^{1}**H NMR** (400 MHz, $CDCl_3$) δ 8.15 (dd, *J* = 7.9, 1.4 Hz, 1H, H_{Ar}), 7.82 – 7.76 (m, 2H, H_{Ar}), 7.55 – 7.48 (m, 1H, H_{Ar}), 7.46 – 7.38 (m, 3H, H_{Ar}), 7.37 – 7.30 (m, 1H, H_{Ar}), 7.16 (d, *J* = 7.5 Hz, 1H, H_{Ar}), 2.37 (s, 3H, CH_3); ^{13}**C NMR** (100 MHz, $CDCl_3$) δ 141.3, 138.8, 138.0, 133.6, 133.0, 132.7, 129.5, 129.0, 127.7, 126.5, 20.2. **LRMS** (ESI, m/z) 233.0 ($[M+H]^+$, 100%); **HRMS** (ESI) calcd for $C_{13}H_{13}O_2SNa$ $[M+H]^+$ 233.0631, found 233.0633. **M.p.**: 74 – 75 °C (lit. 73 – 75 °C). The data recorded are consistent with the literature.[3]

3,5-Dimethyl-1-(phenylsulfonyl)benzene 3d

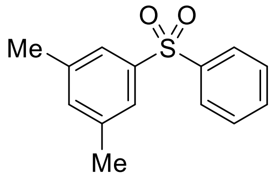

General procedure A was followed with phenylboronic acid (73 mg, 0.6 mmol, 3.0 eq.), $Cu(MeCN)_4BF_4$ (6.4 mg, 0.02 mmol, 10 mol%), DABSO (72 mg, 0.3 mmol, 1.5 eq.), 4,4'-dimethoxy-bipyridine (4.3 mg, 0.02 mmol, 10 mol%) and 1-iodo-3,5-dimethylbenzene (28 µL, 0.2 mmol, 1.0 eq.). The product was purified *via* flash column chromatography (20% Et_2O in petroleum ether) to give the titled product as a white solid (36 mg, 74%).

^{1}**H NMR** (400 MHz, $CDCl_3$) δ 7.89 – 7.84 (m, 2H, H_{Ar}), 7.50 – 7.46 (m, 3H, H_{Ar}), 7.45 – 7.40 (m, 2H, H_{Ar}), 7.09 (s, 1H, H_{Ar}), 2.28 (s, 6H, CH_3); ^{13}**C NMR** (100 MHz, $CDCl_3$) δ 141.9, 141.2, 139.4, 135.0, 133.0, 129.2, 127.6, 125.2, 21.2. **LRMS** (ESI, m/z) 247.1 ($[M+H]^+$, 100%); **HRMS** (ESI) calcd for $C_{14}H_{15}O_2S$ $[M+H]^+$ 247.0787, found 247.0790. **M.p.**: 89 – 90 °C (lit. 88 – 90 °C). The data recorded are consistent with the literature.[4]

4-Methoxyphenyl phenyl sulfone 3e

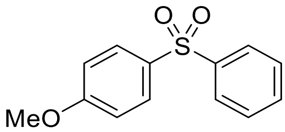

General procedure A was followed with phenylboronic acid (73 mg, 0.6 mmol, 3.0 eq.), $Cu(MeCN)_4BF_4$ (6.4 mg, 0.02 mmol, 10 mol%), DABSO (72 mg, 0.3 mmol, 1.5 eq.), 4,4'-dimethoxy-bipyridine (4.3 mg, 0.02 mmol, 10 mol%) and 4-iodoanisole (47 mg, 0.2 mmol, 1.0 eq.). The product was purified *via* flash column chromatography (20% Et_2O in petroleum ether) to give the titled product as a white solid (32 mg, 65%).

^{1}**H NMR** (400 MHz, $CDCl_3$) δ 7.87 – 7.83 (m, 2H, H_{Ar}), 7.82 – 7.79 (m, 2H, H_{Ar}), 7.50 – 7.44 (m, 1H, H_{Ar}), 7.44 – 7.38 (m, 2H, H_{Ar}), 6.93 – 6.84 (m, 2H, H_{Ar}), 3.77 (s, 3H, OCH_3); ^{13}**C NMR** (100 MHz, $CDCl_3$) δ 163.4, 142.3, 133.1, 132.9, 129.9, 129.2, 127.3, 114.5, 55.7. **LRMS** (ESI, m/z) 247.0 ($[M-H]^-$, 100%); **HRMS** (ESI) calcd for $C_{13}H_{12}O_3SNa$ $[M+Na]^+$ 271.0399, found 271.0340. **M.p.**: 89 – 90 °C (lit. 89 – 90 °C). The data recorded are consistent with the literature.[5]

3-Methoxyphenyl phenyl sulfone 3f

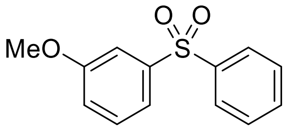

General procedure A was followed with phenylboronic acid (73 mg, 0.6 mmol, 3.0 eq.), $Cu(MeCN)_4BF_4$ (6.4 mg, 0.02 mmol, 10 mol%), DABSO (72 mg, 0.3 mmol, 1.5 eq.), 4,4'-dimethoxy-bipyridine (4.3 mg, 0.02 mmol, 10 mol%) and 3-iodoanisole (47 mg, 0.2 mmol, 1.0 eq.). The product was purified *via* flash column chromatography (30% Et_2O in petroleum ether) to give the titled product as a white solid (39 mg, 78%).

^{1}**H NMR** (400 MHz, $CDCl_3$) δ 7.90 – 7.84 (m, 2H, H_{Ar}), 7.52 – 7.45 (m, 1H, H_{Ar}), 7.45 – 7.40 (m, 3H, H_{Ar}), 7.39 – 7.37 (m, 1H, H_{Ar}), 7.35 – 7.29 (m, 1H, H_{Ar}), 7.04 – 6.95 (m, 1H, H_{Ar}), 3.76 (s, 3H, OCH_3); ^{13}**C NMR** (100 MHz, $CDCl_3$) δ 160.0, 142.7, 141.5, 133.2, 130.4, 129.3, 127.7, 119.9, 119.6, 112.3, 55.7. **LRMS** (ESI, m/z) 249.0 ($[M+H]^+$, 100%); **HRMS** (ESI) calcd for $C_{13}H_{13}O_3S$ $[M]^+$ 249.0580, found 249.0582. **M.p.**: 82°C (lit. 82 °C). The data recorded are consistent with the literature.[3]

4-(Benzenesulfonyl)phenyl methyl sulphide 3g

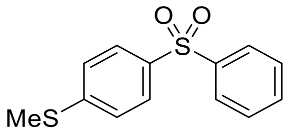

General procedure A was followed with phenylboronic acid (73 mg, 0.6 mmol, 3.0 eq.), $Cu(MeCN)_4BF_4$ (6.4 mg, 0.02 mmol, 10 mol%), DABSO (72 mg, 0.3 mmol, 1.5 eq.), 4,4'-dimethoxy-bipyridine (4.3 mg, 0.02 mmol, 10 mol%) and 4-iodothioanisole (50 mg, 0.2 mmol, 1.0 eq.). The product was purified *via* flash column chromatography (30% Et_2O in petroleum ether) to give the titled product as a white solid (39 mg, 74%).

^{1}**H NMR** (400 MHz, $CDCl_3$) δ 7.87 – 7.81 (m, 2H, H_{Ar}), 7.78 – 7.69 (m, 2H, H_{Ar}), 7.52 – 7.45 (m, 1H, H_{Ar}), 7.44 – 7.37 (m, 2H, H_{Ar}), 7.24 – 7.17 (m, 2H, H_{Ar}), 2.41 (s, 3H, CH_3); ^{13}**C NMR** (100 MHz, $CDCl_3$) δ 146.7, 141.9, 137.2, 133.1, 129.3, 128.0, 127.5, 125.5, 14.7. **LRMS** (ESI, m/z) 265.0 ($[M+H]^+$, 100%); **HRMS** (ESI) calcd for $C_{13}H_{13}O_2S_2$ $[M+H]^+$ 265.0352, found 265.0354. **M.p.**: 110 – 112 °C (lit. 110 – 111°C). The data recorded are consistent with the literature.[6]

4-[4-(Phenylsulfonyl)phenyl]morpholine 3h

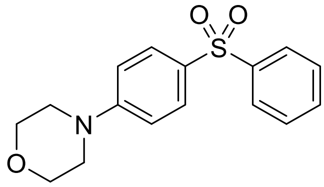

General procedure A was followed with phenylboronic acid (73 mg, 0.6 mmol, 3.0 eq.), $Cu(MeCN)_4BF_4$ (6.4 mg, 0.02 mmol, 10 mol%), DABSO (72 mg, 0.3 mmol, 1.5 eq.), 4,4'-dimethoxy-bipyridine (4.3 mg, 0.02 mmol, 10 mol%) and 4-(4-iodophenyl)morpholine (58 mg, 0.2 mmol, 1.0 eq.). The product was purified *via* flash column chromatography (60% Et_2O in petroleum ether) to give the titled product as a white solid (43 mg, 71%).

^{1}**H NMR** (400 MHz, $CDCl_3$) δ 7.87 – 7.80 (m, 2H, H_{Ar}), 7.77 – 7.68 (m, 2H, H_{Ar}), 7.47 – 7.42 (m, 1H, H_{Ar}), 7.42 – 7.35 (m, 2H, H_{Ar}), 6.86 – 6.77 (m, 2H, H_{Ar}), 3.78 – 3.42 (m, 4H, OCH_2), 3.22 – 3.17 (m, 4H, NCH_2); ^{13}**C NMR** (100 MHz, $CDCl_3$) δ 154.1, 142.8, 132.6, 130.0, 129.5, 129.1, 127.2, 113.8, 66.5, 47.4. **LRMS** (ESI, m/z) 304.1 ($[M+H]^+$, 100%); **HRMS** (ESI) calcd for $C_{16}H_{18}O_3NS$ $[M+H]^+$ 304.1002, found 304.1004. **IR** ν_{max} (film): 3062, 2850, 1591, 1507, 1449, 1297 (SO_2), 1245, 1150 (SO_2), 1105, 927, 762, 651 cm^{-1}. **M.p.**: 157 – 160 °C.

4-Aminophenyl phenyl sulfone 3i

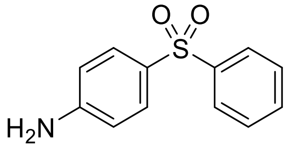

General procedure A was followed with phenylboronic acid (73 mg, 0.6 mmol, 3.0 eq.), $Cu(MeCN)_4BF_4$ (6.4 mg, 0.02 mmol, 10 mol%), DABSO (72 mg, 0.3 mmol, 1.5 eq.), 4,4'-dimethoxy-bipyridine (4.3 mg, 0.02 mmol, 10 mol%) and 4-iodoaniline (44 mg, 0.2 mmol, 1.0 eq.). The product was purified *via* flash column chromatography (70% Et_2O in petroleum ether) to give the titled product as a white solid (30 mg, 64%).
^{1}**H NMR** (400 MHz, $CDCl_3$) δ 7.87 – 7.77 (m, 2H, H_{Ar}), 7.67 – 7.58 (m, 2H, H_{Ar}), 7.47 – 7.42 (m, 1H, H_{Ar}), 7.41 – 7.34 (m, 2H, H_{Ar}), 6.63 – 6.52 (m, 2H, H_{Ar}), 4.11 (br s, 2H, NH_2); ^{13}**C NMR** (100 MHz, $CDCl_3$) δ 151.1, 142.9, 132.5, 129.9, 129.4, 129.1, 127.1, 114.2. **LRMS** (ESI, m/z) 256.0 ($[M+Na]^+$, 100%); **HRMS** (ESI) calcd for $C_{12}H_{11}O_2NSNa$ $[M+Na]^+$ 256.0403, found 256.0402. **M.p.**: 168 – 170 °C (lit. 169-172 °C). The data recorded are consistent with the literature.[7]

4-Hydroxyphenyl phenyl sulfone 3j

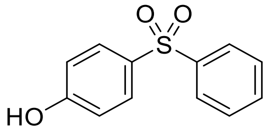

General procedure A was followed with phenylboronic acid (73 mg, 0.6 mmol, 3.0 eq.), $Cu(MeCN)_4BF_4$ (6.4 mg, 0.02 mmol, 10 mol%), DABSO (72 mg, 0.3 mmol, 1.5 eq.), 4,4'-dimethoxy-bipyridine (4.3 mg, 0.02 mmol, 10 mol%) and 4-iodophenol (44 mg, 0.2 mmol, 1.0 eq.). The product was purified *via* flash column chromatography (50% Et_2O in petroleum ether) to give the titled product as a white solid (36 mg, 71%).
^{1}**H NMR** (400 MHz, $CDCl_3$) δ 7.87 – 7.78 (m, 2H, H_{Ar}), 7.73 (d, *J* = 8.8 Hz, 2H, H_{Ar}), 7.51 – 7.45 (m, 1H, H_{Ar}), 7.45 – 7.37 (m, 2H, H_{Ar}), 6.85 (d, *J* = 8.8 Hz, 2H, H_{Ar}), 6.22 (br s, 1H, O*H*); ^{13}**C NMR** (100 MHz, $CDCl_3$) δ 160.3, 142, 133.1, 132.7, 130.1, 129.3, 127.3, 116.2. **LRMS** (ESI, m/z) 257.0 ($[M+Na]^+$, 100%); **HRMS** (ESI) calcd for $C_{12}H_{10}O_3SNa$ $[M+Na]^+$ 257.0243, found 257.0243. **M.p.**: 136 – 138 °C (lit. 136 – 137 °C). The data recorded are consistent with the literature.[8]

4'-(Phenylsulfonyl)acetophenone 3k

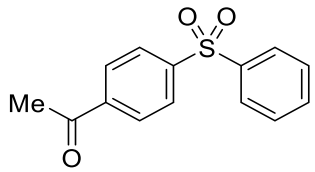

General procedure A was followed with phenylboronic acid (73 mg, 0.6 mmol, 3.0 eq.), $Cu(MeCN)_4BF_4$ (6.4 mg, 0.02 mmol, 10 mol%), DABSO (72 mg, 0.3 mmol, 1.5 eq.), 4,4'-dimethoxy-bipyridine (4.3 mg, 0.02 mmol, 10 mol%) and 4'-iodoacetophenone (49 mg, 0.2 mmol, 1.0 eq.). The product was purified *via* flash column chromatography (30% Et_2O in petroleum ether) to give the titled product as a white solid (33 mg, 64%).

^{1}**H NMR** (400 MHz, $CDCl_3$) δ 8.03 – 7.94 (m, 4H, H_{Ar}), 7.91 – 7.86 (m, 2H, H_{Ar}), 7.56 – 7.50 (m, 1H, H_{Ar}), 7.49 – 7.42 (m, 2H, H_{Ar}), 2.55 (s, 3H, $COCH_3$); ^{13}**C NMR** (100 MHz, $CDCl_3$) δ 196.7, 145.5, 140.8, 140.3, 133.7, 129.5, 129.1, 128.0, 127.9, 26.9. **LRMS** (ESI, m/z) 283.0 ([M+Na]$^+$, 100%); **HRMS** (ESI) calcd for $C_{14}H_{12}O_3SNa$ [M+Na]$^+$ 283.0399, found 283.0401. **M.p.**: 133 – 135 °C (lit. 133 – 135 °C). The data recorded are consistent with the literature.[9]

4-(Phenylsulfonyl)benzonitrile 3l

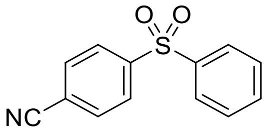

General procedure A was followed with phenylboronic acid (73 mg, 0.6 mmol, 3.0 eq.), $Cu(MeCN)_4BF_4$ (6.4 mg, 0.02 mmol, 10 mol%), DABSO (72 mg, 0.3 mmol, 1.5 eq.), 4,4'-dimethoxy-bipyridine (4.3 mg, 0.02 mmol, 10 mol%) and 4-iodobenzonitrile (46 mg, 0.2 mmol, 1.0 eq.). The product was purified *via* flash column chromatography (40% Et_2O in petroleum ether) to give the titled product as a white solid (34 mg, 70%).

^{1}**H NMR** (400 MHz, $CDCl_3$) δ 7.99 (d, *J* = 8.7 Hz, 2H, H_{Ar}), 7.88 (d, *J* = 7.1 Hz, 2H, H_{Ar}), 7.73 (d, *J* = 8.7 Hz, 2H, H_{Ar}), 7.59 – 7.54 (m, 1H, H_{Ar}), 7.48 (t, *J* = 7.5 Hz, 2H, H_{Ar}); ^{13}**C NMR** (100 MHz, $CDCl_3$) δ 145.9, 140.1, 134.1, 133.1, 129.7, 128.3, 128.0, 117.2, 117.0. **LRMS** (ESI, m/z) 266.0 ([M+Na]$^+$, 100%); **HRMS** (ESI) calcd for $C_{13}H_9O_2NSNa$ [M+Na]$^+$ 266.0246, found 266.0248. **M.p.**: 127 – 129 °C (lit. 125 – 127 °C). The data recorded are consistent with the literature.[10]

Methyl 4-(phenylsulfonyl)benzoate 3m

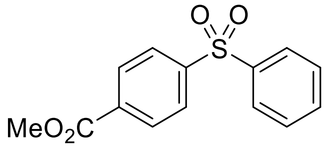

General procedure A was followed with phenylboronic acid (73 mg, 0.6 mmol, 3.0 eq.), $Cu(MeCN)_4BF_4$ (6.4 mg, 0.02 mmol, 10 mol%), DABSO (72 mg, 0.3 mmol, 1.5 eq.), 4,4'-dimethoxy-bipyridine (4.3 mg, 0.02 mmol, 10 mol%) and methyl 4-iodobenzoate (52 mg, 0.2 mmol, 1.0 eq.). The product was purified *via* flash column chromatography (30% Et_2O in petroleum ether) to give the titled product as a white solid (37 mg, 67%).

^{1}**H NMR** (400 MHz, $CDCl_3$) δ 8.08 (d, *J* = 8.7 Hz, 2H, H_{Ar}), 7.94 (d, *J* = 8.7 Hz, 2H, H_{Ar}), 7.91 – 7.85 (m, 2H, H_{Ar}), 7.56 – 7.50 (m, 1H, H_{Ar}), 7.49 – 7.42 (m, 2H, H_{Ar}), 3.86 (s, 3H, CO_2CH_3); ^{13}**C NMR** (100 MHz, $CDCl_3$) δ 165.5, 145.5, 140.8, 134.3, 133.7, 130.5, 129.5, 127.9, 127.7, 52.7. **LRMS** (ESI, m/z) 299.0 ($[M+Na]^+$, 100%); **HRMS** (ESI) calcd for $C_{14}H_{12}O_4SNa$ $[M+Na]^+$ 299.0349, found 299.0348. **M.p.**: 145 – 147 °C (lit. 147 °C). The data recorded are consistent with the literature.[11]

3-(Phenylsulfonyl)benzaldehyde 3n

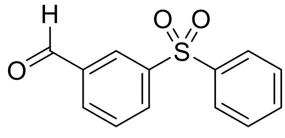

General procedure A was followed with phenylboronic acid (73 mg, 0.6 mmol, 3.0 eq.), $Cu(MeCN)_4BF_4$ (6.4 mg, 0.02 mmol, 10 mol%), DABSO (72 mg, 0.3 mmol, 1.5 eq.), 4,4'-dimethoxy-bipyridine (4.3 mg, 0.02 mmol, 10 mol%) and 3-iodobenzaldehyde (46 mg, 0.2 mmol, 1.0 eq.). The product was purified *via* flash column chromatography (50% Et_2O in petroleum ether) to give the titled product as a white solid (22 mg, 46%).

^{1}**H NMR** (400 MHz, $CDCl_3$) δ 9.99 (s, 1H, C*H*O), 8.40 – 8.32 (m, 1H, H_{Ar}), 8.13 (ddd, *J* = 7.8, 1.9, 1.2 Hz, 1H, H_{Ar}), 8.01 (d, *J* = 7.7 Hz, 1H, H_{Ar}), 7.92 – 7.89 (m, 2H, H_{Ar}), 7.64 (t, *J* = 7.8 Hz, 1H, H_{Ar}), 7.56 – 7.52 (m, 1H, H_{Ar}), 7.50 – 7.45 (m, 2H, H_{Ar}); ^{13}**C NMR** (100 MHz, $CDCl_3$) δ 190.3, 143.2, 140.7, 137.1, 133.7, 133.5, 132.9, 130.3, 129.6, 128.8, 127.9. **LRMS** (ESI, m/z) 247.0 ($[M+H]^+$, 100%); **HRMS** (ESI) calcd for $C_{13}H_{11}O_3S$ $[M+H]^+$ 247.0423, found 247.0424. **IR** ν_{max} (film): 3065, 2849, 1702 (CO), 1595, 1582, 1447, 1323, 1306 (SO_2), 1204, 1151 (SO_2), 1095, 900, 730, 687 cm^{-1}. **M.p.**: 86 – 89 °C.

4-Benzenesulfonyl-benzoic acid amide 3o

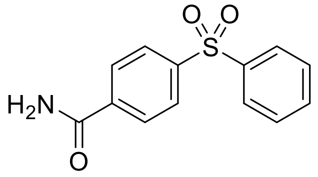

General procedure A was followed with phenylboronic acid (73 mg, 0.6 mmol, 3.0 eq.), $Cu(MeCN)_4BF_4$ (6.4 mg, 0.02 mmol, 10 mol%), DABSO (72 mg, 0.3 mmol, 1.5 eq.), 4,4'-dimethoxy-bipyridine (4.3 mg, 0.02 mmol, 10 mol%) and 4-iodobenzamide (49 mg, 0.2 mmol, 1.0 eq.). The product was purified *via* flash column chromatography (10% EtOAc degraded to 70% EtOAc in petroleum ether) to give the titled product as a white solid (38 mg, 73%).

^{1}**H NMR** (400 MHz, DMSO-*d*$_6$) δ 8.20 (br s, 1H, N*H*), 8.04 (s, 4H, H_{Ar}), 8.01 – 7.96 (m, 2H, H_{Ar}), 7.74 – 7.68 (m, 1H, H_{Ar}), 7.68 – 7.60 (m, 3H, *NH* and H_{Ar}); ^{13}**C NMR** (100 MHz, DMSO-*d*$_6$) δ 167.1, 143.7, 141.1, 139.4, 134.5, 130.3, 129.2, 128.0, 127.9. **LRMS** (ESI, m/z) 284.0 ([M+Na]$^+$, 100%); **HRMS** (ESI) calcd for $C_{13}H_{11}O_3NSNa$ [M+Na]$^+$ 284.0352, found 284.0352. **IR** ν_{max} (film): 3418 (NH_2), 2922, 1684 (CO), 1467, 1296 (SO_2), 1205, 1162 (SO_2), 1103, 1040, 996, 863, 723, 665 cm^{-1}. **M.p.**: 173 – 175 °C.

3-Nitrodiphenyl sulfone 3p

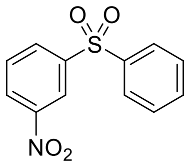

General procedure A was followed with phenylboronic acid (73 mg, 0.6 mmol, 3.0 eq.), $Cu(MeCN)_4BF_4$ (6.4 mg, 0.02 mmol, 10 mol%), DABSO (72 mg, 0.3 mmol, 1.5 eq.), 4,4'-dimethoxy-bipyridine (4.3 mg, 0.02 mmol, 10 mol%) and 3-iodonitrobenzene (50 mg, 0.2 mmol, 1.0 eq.). The product was purified *via* flash column chromatography (40% Et_2O in petroleum ether) to give the titled product as a white solid (39 mg, 75%).

^{1}**H NMR** (400 MHz, $CDCl_3$) δ 8.70 (app. t, *J* = 2.0 Hz, 1H, H_{Ar}), 8.35 (ddd, *J* = 8.0, 2.0, 1.1 Hz, 1H, H_{Ar}), 8.21 (ddd, *J* = 8.0, 2.0, 1.1 Hz, 1H, H_{Ar}), 7.95 – 7.89 (m, 2H, H_{Ar}), 7.67 (app. t, *J* = 8.0 Hz, 1H, H_{Ar}), 7.60 – 7.54 (m, 1H, H_{Ar}), 7.53 – 7.45 (m, 2H, H_{Ar}); ^{13}**C NMR** (100 MHz, $CDCl_3$) δ 148.4, 144.0, 140.1, 134.1, 133.1, 130.8, 129.8, 128.0, 127.7, 123.0. **LRMS** (ESI, m/z) 286.0 ([M+Na]$^+$, 100%); **HRMS** (ESI) calcd for $C_{12}H_9O_4NSNa$ [M+Na]$^+$ 286.0145, found 286.0146. **M.p.**: 78 – 80 °C (lit. 79 – 81 °C). The data recorded are consistent with the literature.[12]

1-(Phenylsulfonyl)-4-(trifluoromethyl)benzene 3q

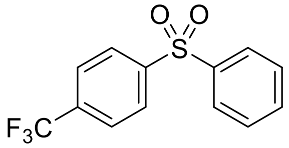

General procedure A was followed with phenylboronic acid (73 mg, 0.6 mmol, 3.0 eq.), $Cu(MeCN)_4BF_4$ (6.4 mg, 0.02 mmol, 10 mol%), DABSO (72 mg, 0.3 mmol, 1.5 eq.), 4,4'-dimethoxy-bipyridine (4.3 mg, 0.02 mmol, 10 mol%) and 4-iodobenzotrifluoride (29 µL, 0.2 mmol, 1.0 eq.). The product was purified *via* flash column chromatography (20% Et_2O in petroleum ether) to give the titled product as a white solid (41 mg, 72%).

^{1}**H NMR** (500 MHz, $CDCl_3$) δ 8.00 (d, *J* = 8.2 Hz, 2H, H_{Ar}), 7.93 – 7.84 (m, 2H, H_{Ar}), 7.70 (d, *J* = 8.2 Hz, 2H, H_{Ar}), 7.59 – 7.51 (m, 1H, H_{Ar}), 7.46 – 7.42 (m, 2H, H_{Ar}); ^{13}**C NMR** (125 MHz, $CDCl_3$) δ 145.2, 140.6, 134.9 (q, $^2J_{C-F}$ = 33 Hz, C_{Ar}), 133.8, 129.6, 128.2, 127.9, 126.5 (q, $^3J_{C-F}$ = 4 Hz, C_{Ar}), 123.1 (q, $^1J_{C-F}$ = 273 Hz, C_{Ar}); ^{19}**F NMR** (376 MHz, $CDCl_3$) δ -63.2. **LRMS** (ESI, m/z) 287.0 ($[M+H]^+$, 100%). **HRMS** (EI) calcd for $C_{13}H_{10}F_3O_2S$ $[M]^+$ 287.0348, found 287.0448. **M.p.**: 93 – 95 °C (lit. 91 – 92 °C). The data recorded are consistent with the literature.[5]

3,5-Dichloro-1-(phenylsulfonyl)benzene 3r

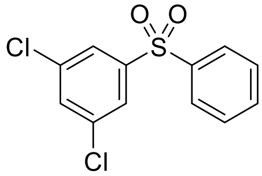

General procedure A was followed with phenylboronic acid (73 mg, 0.6 mmol, 3.0 eq.), $Cu(MeCN)_4BF_4$ (6.4 mg, 0.02 mmol, 10 mol%), DABSO (72 mg, 0.3 mmol, 1.5 eq.), 4,4'-dimethoxy-bipyridine (4.3 mg, 0.02 mmol, 10 mol%) and 1-iodo-3,5-dichlorobenzene (55 mg, 0.2 mmol, 1.0 eq.). The product was purified *via* flash column chromatography (20% Et_2O in petroleum ether) to give the titled product as a white solid (43 mg, 75%).

^{1}**H NMR** (400 MHz, $CDCl_3$) δ 7.89 – 7.86 (m, 2H, H_{Ar}), 7.74 (d, *J* = 1.9 Hz, 2H, H_{Ar}), 7.60 – 7.54 (m, 1H, H_{Ar}), 7.52 – 7.44 (m, 3H, H_{Ar}); ^{13}**C NMR** (100 MHz, $CDCl_3$) δ 144.6, 140.3, 136.3, 134.0, 133.3, 129.7, 128.0, 126.0. **LRMS** (ESI, m/z) 308.2 ($[^{35}M+Na]^+$, 100%); **HRMS** (ESI) calcd for $C_{12}H_8O_2Cl_2SNa$ $[^{35}M+Na]^+$ 308.9514, found 308.9515. **IR** ν_{max} (film): 3071, 1566, 1477, 1449, 1329 (SO_2), 1179, 1163 (SO_2), 1140, 866, 802, 718, 686 cm^{-1}. **M.p.**: 125 – 128 °C.

4-Phenylsulfonylbromobenzene and 4-phenylsulfonyliodobenzene 3s/3s’

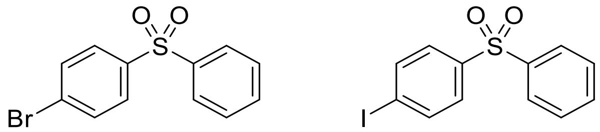

General procedure A was followed with phenylboronic acid (73 mg, 0.6 mmol, 3.0 eq.), $Cu(MeCN)_4BF_4$ (6.4 mg, 0.02 mmol, 10 mol%), DABSO (72 mg, 0.3 mmol, 1.5 eq.), 4,4’-dimethoxy-bipyridine (4.3 mg, 0.02 mmol, 10 mol%) and 1-bromo-4-iodobenzene (57 mg, 0.2 mmol, 1.0 eq.). The product was purified *via* flash column chromatography (50% Et_2O in petroleum ether) to give the titled inseparable products as a white solid (39 mg), in a ratio of 3:1.

4-Phenylsulfonylbromobenzene

^{1}**H NMR** (400 MHz, $CDCl_3$) δ 7.89 – 7.83 (m, 2H, H_{Ar}), 7.75 – 7.71 (m, 2H, H_{Ar}), 7.60 – 7.55 (m, 2H, H_{Ar}), 7.54 – 7.49 (m, 1H, H_{Ar}), 7.48 – 7.39 (m, 2H, H_{Ar}); ^{13}**C NMR** (125 MHz, $CDCl_3$) δ 141.2, 140.7, 133.5, 132.6, 129.4, 129.2, 128.5, 127.7. **LRMS** (ESI, m/z) 296.9 ($[M+H]^+$, 100%); **HRMS** (ESI) calcd for $C_{12}H_9O_2{}^{79}BrSNa$ $[^{79}M+Na]^+$ 318.9399 and $C_{12}H_9O_2{}^{81}BrSNa$ $[^{81}M+Na]^+$ 320.9378, found 318.9400 and 320.9379. The data recorded are consistent with the literature.[13]

4-Phenylsulfonyliodobenzene

^{1}**H NMR** (400 MHz, $CDCl_3$) δ 7.88 – 7.83 (m, 2H, H_{Ar}), 7.79 (d, *J* = 8.5 Hz, 2H, H_{Ar}), 7.60 – 7.55 (m, 2H, H_{Ar}), 7.54 – 7.49 (m, 1H, H_{Ar}), 7.48 – 7.39 (m, 2H, H_{Ar}); ^{13}**C NMR** (125 MHz, $CDCl_3$) δ 141.4, 141.2, 138.6, 133.5, 129.4, 129.0, 127.7, 101.0. **LRMS** (ESI, m/z) 366.9 ($[M+H]^+$, 100%); **HRMS** (ESI) calcd for $C_{12}H_9O_2ISNa$ $[M+Na]^+$ 366.9260, found 366.9261. The data recorded are consistent with the literature.[14]

IR ν_{max} (film): 3087, 2917, 2849, 1572, 1446, 1387, 1320 (SO_2), 1155 (SO_2), 1104, 1068, 1008, 822, 741, 689, 686 cm^{-1}.

5-(Phenylsulfonyl)indole 3t

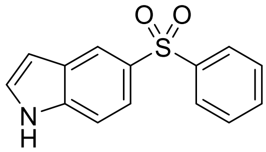

General procedure A was followed with phenylboronic acid (73 mg, 0.6 mmol, 3.0 eq.), $Cu(MeCN)_4BF_4$ (6.4 mg, 0.02 mmol, 10 mol%), DABSO (72 mg, 0.3 mmol, 1.5 eq.), 4,4'-dimethoxy-bipyridine (4.3 mg, 0.02 mmol, 10 mol%) and 5-iodoindole (49 mg, 0.2 mmol, 1.0 eq.). The product was purified *via* flash column chromatography (20% EtOAc in petroleum ether) to give the titled product as a white solid (40 mg, 79%).

^{1}**H NMR** (400 MHz, DMSO-d_6) δ 11.68 (br s, 1H, N*H*), 8.25 (d, *J* = 1.3 Hz, 1H, H_{Ar}), 7.93 (dd, *J* = 8.2, 1.3 Hz, 2H, H_{Ar}), 7.66 – 7.51 (m, 6H, H_{Ar}), 6.66 (d, *J* = 3.0 Hz, 1H, H_{Ar}); ^{13}**C NMR** (100 MHz, DMSO-d_6) δ 143.2, 138.4, 133.4, 131.6, 130.0, 128.9, 127.7, 127.3, 121.5, 120.1, 112.9, 103.3. **LRMS** (ESI, m/z) 280.0 ([M+Na]$^+$, 100%); **HRMS** (ESI) calcd for $C_{14}H_{11}O_2NNaS$ [M+Na]$^+$ 280.0403, found 280.0403. **IR** ν_{max} (film): 3427 (NH), 2923, 1660, 1431, 1302 (SO2), 1208, 1150 (SO2), 1107, 1042, 996, 767, 731 cm^{-1}. **M.p.**: 133 – 135 °C.

8-Chloro-3-(phenylsulfonyl)quinoline 3u

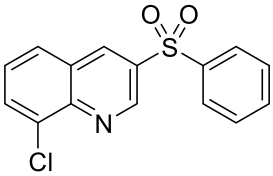

General procedure A was followed with phenylboronic acid (73 mg, 0.6 mmol, 3.0 eq.), $Cu(MeCN)_4BF_4$ (6.4 mg, 0.02 mmol, 10 mol%), DABSO (72 mg, 0.3 mmol, 1.5 eq.), 4,4'-dimethoxy-bipyridine (4.3 mg, 0.02 mmol, 10 mol%) and 8-chloro-3-iodoquinoline (58 mg, 0.2 mmol, 1.0 eq.). The product was purified *via* flash column chromatography (60% Et_2O in petroleum ether) to give the titled product as a white solid (32 mg, 53%).

^{1}**H NMR** (400 MHz, $CDCl_3$) δ 9.30 (d, *J* = 2.3 Hz, 1H, H_{Ar}), 8.78 (d, *J* = 2.3 Hz, 1H, H_{Ar}), 8.00 – 7.95 (m. 2H, H_{Ar}), 7.92 (dd, *J* = 7.5, 1.3 Hz, 1H, H_{Ar}), 7.84 (dd, *J* = 8.3, 1.3 Hz, 1H, H_{Ar}), 7.58 – 7.52 (m, 2H, H_{Ar}), 7.51 – 7.45 (m, 2H, H_{Ar}); ^{13}**C NMR** (100 MHz, $CDCl_3$) δ 147.8, 145.5, 140.6, 137.2, 135.8, 134.1, 134.0, 132.8, 129.7, 128.5, 128.2, 127.9, 127.8. **LRMS** (ESI, m/z) 304.0 ([^{35}M+H]$^+$, 100%), 306.0 ([^{37}M+H]$^+$, 25%); **HRMS** (ESI) calcd for $C_{15}H_{11}O_2NClS$ [^{35}M+H]$^+$ 304.1094, found 304.1094. **M.p.**: 227 – 229 °C (lit. 226 – 227 °C). The data recorded are consistent with the literature.[15]

2-Methoxy-5-(phenylsulfonyl)pyridine 3v

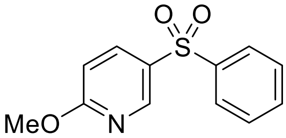

General procedure A was followed with phenylboronic acid (73 mg, 0.6 mmol, 3.0 eq.), $Cu(MeCN)_4BF_4$ (6.4 mg, 0.02 mmol, 10 mol%), DABSO (72 mg, 0.3 mmol, 1.5 eq.), 4,4'-dimethoxy-bipyridine (4.3 mg, 0.02 mmol, 10 mol%) and 5-iodo-2-methoxypyridine (47 mg, 0.2 mmol, 1.0 eq.). The product was purified *via* flash column chromatography (10% EtOAc in petroleum ether) to give the titled product as a white solid (19 mg, 39%).

^{1}**H NMR** (400 MHz, $CDCl_3$) δ 8.69 (dd, *J* = 2.5, 0.5 Hz, 1H, H_{Ar}), 7.93 (dd, *J* = 8.8, 2.5 Hz, 1H, H_{Ar}), 7.88 – 7.84 (m, 2H, H_{Ar}), 7.54 – 7.50 (m, 1H, H_{Ar}), 7.48 – 7.44 (m, 2H, H_{Ar}), 6.73 (dd, *J* = 8.8, 0.5 Hz, 1H, H_{Ar}), 3.91 (s, 3H, OCH_3); ^{13}**C NMR** (125 MHz, $CDCl_3$) δ 165.7, 147.2, 140.7, 136.6, 132.3, 129.9, 128.4, 126.4, 110.7, 53.3. **LRMS** (ESI, m/z) 250.1 ($[M+H]^+$, 100%); **HRMS** (ESI) calcd for $C_{12}H_{12}O_3NS$ $[M+H]^+$ 250.0532, found 250.0534. **IR** ν_{max} (film): 3062, 2949, 1589, 1483, 1447, 1374, 1323 (SO_2), 1307, 1286, 1160 (SO_2), 1112, 1014, 833, 756, 727, 689 cm^{-1}. **M.p.**: 88 – 91 °C.

1-Methyl-4-(phenylsulfonyl)pyrazole 3w

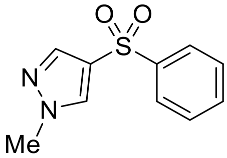

General procedure A was followed with phenylboronic acid (73 mg, 0.6 mmol, 3.0 eq.), $Cu(MeCN)_4BF_4$ (6.4 mg, 0.02 mmol, 10 mol%), DABSO (72 mg, 0.3 mmol, 1.5 eq.), 4,4'-dimethoxy-bipyridine (4.3 mg, 0.02 mmol, 10 mol%) and 4-iodo-1-methyl-1H-pyrrazole (42 mg, 0.2 mmol, 1.0 eq.). The product was purified *via* flash column chromatography (20% EtOAc in petroleum ether) to give the titled product as a white solid (33 mg, 76%).

^{1}**H NMR** (400 MHz, $CDCl_3$) δ 7.90 – 7.84 (m, 2H, H_{Ar}), 7.77 (s, 1H, H_{Ar}), 7.72 (s, 1H, H_{Ar}), 7.54 – 7.47 (m, 1H, H_{Ar}), 7.46 – 7.41 (m, 2H, H_{Ar}), 3.85 (s, 3H, CH_3); ^{13}**C NMR** (100 MHz, $CDCl_3$) δ 142.8, 139.1, 133.1, 132.2, 129.3, 126.9, 124.5, 39.7. **LRMS** (ESI, m/z) 223.1 ($[M+H]^+$, 100%); **HRMS** (ESI) calcd for $C_{10}H_{11}O_2N_2S$ $[M+H]^+$ 223.0536, found 223.0538. **M.p.**: 109 – 110 °C (lit. 107 – 108 °C). The data recorded are consistent with the literature.[16]

1-Cyclohepten-1-yl-phenylsulfone 3x

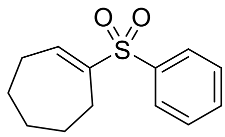

General procedure A was followed with phenylboronic acid (73 mg, 0.6 mmol, 3.0 eq.), $Cu(MeCN)_4BF_4$ (6.4 mg, 0.02 mmol, 10 mol%), DABSO (72 mg, 0.3 mmol, 1.5 eq.), 4,4'-dimethoxy-bipyridine (4.3 mg, 0.02 mmol, 10 mol%) and 1-iodocycloheptene (44 mg, 0.2 mmol, 1.0 eq.). The product was purified *via* flash column chromatography (30% Et_2O in petroleum ether) to give the titled product as a colourless oil which solidified when left standing (27 mg, 58%).

^{1}**H NMR** (400 MHz, $CDCl_3$) δ 7.81 – 7.74 (m, 2H, H_{Ar}), 7.56 – 7.49 (m, 1H, H_{Ar}), 7.49 – 7.41 (m, 2H, H_{Ar}), 7.24 (t, *J* = 6.5 Hz, 1H, C*H*), 2.34 – 2.25 (m, 4H, CH_2), 1.71 – 1.57 (m, 2H, CH_2), 1.52 – 1.42 (m, 2H, CH_2), 1.36 – 1.27 (m, 2H, CH_2); ^{13}**C NMR** (100 MHz, $CDCl_3$) δ 144.3, 143.1, 139.6, 133.0, 129.1, 128.0, 31.3, 28.6, 27.6, 26.1, 25.4. **LRMS** (ESI, m/z) 237.1 ($[M+H]^+$, 100%); **HRMS** (ESI) calcd for $C_{13}H_{17}O_2S$ $[M+H]^+$ 237.0944, found 237.0946. **M.p.**: 33 – 35 °C (lit. 32 – 35 °C). The data recorded are consistent with the literature.[17]

1-Cyclohexen-1-yl-phenylsulfone 3y

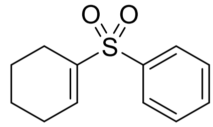

General procedure A was followed with phenylboronic acid (73 mg, 0.6 mmol, 3.0 eq.), $Cu(MeCN)_4BF_4$ (6.4 mg, 0.02 mmol, 10 mol%), DABSO (72 mg, 0.3 mmol, 1.5 eq.), 4,4'-dimethoxy-bipyridine (4.3 mg, 0.02 mmol, 10 mol%) and 1-iodocyclohexene (42 mg, 0.2 mmol, 1.0 eq.). The product was purified *via* flash column chromatography (30% Et_2O in petroleum ether) to give the titled product as a colourless oil which solidified when left standing (28 mg, 64%).

^{1}**H NMR** (400 MHz, $CDCl_3$) δ 7.82 – 7.77 (m, 2H, H_{Ar}), 7.56 – 7.51 (m, 1H, H_{Ar}), 7.49 – 7.43 (m, 2H, H_{Ar}), 7.00 (dt, *J* = 3.9, 2.2 Hz, 1H, C*H*), 2.25 – 2.15 (m, 2H, SO_2CCH_2), 2.12 – 2.06 (m, 1H, SO_2CCHCH_2), 1.62 – 1.54 (m, 2H, SO_2CCHCH_2), 1.54 – 1.46 (m, 2H, $SO_2CCH_2CH_2$); ^{13}**C NMR** (100 MHz, $CDCl_3$) δ 139.8, 139.4, 138.5, 133.1, 129.1, 128.0, 25.5, 22.8, 21.8, 20.8. **LRMS** (ESI, m/z) 223.1 ($[M+H]^+$, 100%); **HRMS** (ESI) calcd for $C_{12}H_{15}O_2S$ $[M+H]^+$ 223.0787, found 223.0790. **M.p.**: 42 – 44 °C (lit. 43 °C). The data recorded are consistent with the literature.[18]

4-(4-Tosylphenyl)morpholine 4a

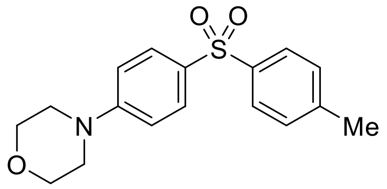

General procedure A was followed with 4-tolylboronic acid (82 mg, 0.6 mmol, 3.0 eq.), $Cu(MeCN)_4BF_4$ (6.4 mg, 0.02 mmol, 10 mol%), DABSO (72 mg, 0.3 mmol, 1.5 eq.), 4,4'-dimethoxy-bipyridine (4.3 mg, 0.02 mmol, 10 mol%) and 4-(4-iodophenyl)morpholine (58 mg, 0.2 mmol, 1.0 eq.). The product was purified *via* flash column chromatography (20% EtOAc in petroleum ether) to give the titled product as a white solid (46 mg, 72%).

^{1}**H NMR** (400 MHz, $CDCl_3$) δ 7.76 – 7.65 (m, 4H, H_{Ar}), 7.19 (d, *J* = 8.0 Hz, 2H, H_{Ar}), 6.80 (d, *J* = 9.1 Hz, 2H, H_{Ar}), 3.78 – 3.72 (m, 4H, OCH_2), 3.22 – 3.14 (m, 4H, NCH_2), 2.31 (s, 3H, CH_3); ^{13}**C NMR** (100 MHz, $CDCl_3$) δ 154.0, 143.4, 139.9, 130.6, 129.8, 129.3, 127.2, 113.9, 66.5, 47.4, 21.5. **LRMS** (ESI, m/z) 318.1 ($[M+H]^+$, 100%); **HRMS** (ESI) calcd for $C_{17}H_{20}O_3NS$ $[M+H]^+$ 318.1158, found 318.1151. **IR** ν_{max} (film): 3062, 2580, 1591, 1507, 1449, 1297 (SO_2), 1245, 1150 (SO_2), 1121, 1105, 927, 821, 651 cm^{-1}. **M.p.**: 150 – 153 °C.

(4-*tert*-Butylphenyl)-*p*-tolyl sulfone 4b

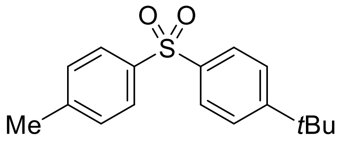

General procedure A was followed with 4-*tert*-butylphenylboronic acid (107 mg, 0.6 mmol, 3.0 eq.), $Cu(MeCN)_4BF_4$ (6.4 mg, 0.02 mmol, 10 mol%), DABSO (72 mg, 0.3 mmol, 1.5 eq.), 4,4'-dimethoxy-bipyridine (4.3 mg, 0.02 mmol, 10 mol%) and 4-iodotoluene (44 mg, 0.2 mmol, 1.0 eq.). The product was purified *via* flash column chromatography (20% Et_2O in petroleum ether) to give the titled product as a white solid (45 mg, 78%).

^{1}**H NMR** (400 MHz, $CDCl_3$) δ 7.79 – 7.73 (m, 4H, H_{Ar}), 7.42 (d, *J* = 8.7 Hz, 2H, H_{Ar}), 7.22 (d, *J* = 8.0 Hz, 2H, H_{Ar}), 2.32 (s, 3H, CH_3), 1.23 (s, 9H, $C(CH_3)_3$); ^{13}**C NMR** (100 MHz, $CDCl_3$) δ 156.9, 143.9, 139.0, 138.9, 129.9, 127.7, 127.4, 126.3, 35.2, 31.1, 21.6. **LRMS** (ESI, m/z) 289.1 ($[M+H]^+$, 100%); **HRMS** (ESI) calcd for $C_{17}H_{21}O_2S$ $[M+H]^+$ 289.1257, found 289.1258. **M.p.**: 86 – 87 °C (lit. 80 – 81 °C). The data recorded are consistent with the literature.[19]

4-[4-(Naphthalen-2-ylsulfonyl)phenyl]morpholine 4c

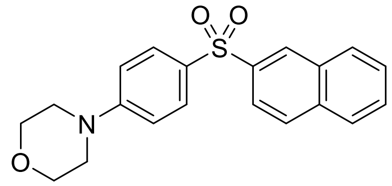

General procedure A was followed with 2-naphthylboronic acid (103 mg, 0.6 mmol, 3.0 eq.), $Cu(MeCN)_4BF_4$ (6.4 mg, 0.02 mmol, 10 mol%), DABSO (72 mg, 0.3 mmol, 1.5 eq.), 4,4'-dimethoxy-bipyridine (4.3 mg, 0.02 mmol, 10 mol%) and 4-(4-iodophenyl)morpholine (57.8 mg, 0.2 mmol, 1.0 eq.). The product was purified *via* flash column chromatography (20% EtOAc in petroleum ether) to give the titled product as a white solid (54 mg, 76%).

^{1}**H NMR** (400 MHz, $CDCl_3$) δ 8.48 – 8.41 (m, 1H, H_{Ar}), 7.89 (dd, *J* = 7.3, 1.6 Hz, 1H, H_{Ar}), 7.85 – 7.72 (m, 5H, H_{Ar}), 7.57 – 7.48 (m, 2H, H_{Ar}), 6.80 (d, *J* = 9.1 Hz, 2H, H_{Ar}), 3.78 – 3.71 (m, 4H, OCH_2), 3.21 – 3.14 (m, 4H, NCH_2); ^{13}**C NMR** (100 MHz, $CDCl_3$) δ 154.1, 139.6, 134.8, 132.3, 130.1, 129.6, 129.5, 129.3, 128.8, 128.2, 127.9, 127.5, 122.6, 113.9, 66.5, 47.4. **LRMS** (ESI, m/z) 376.1 ($[M+Na]^+$, 100%); **HRMS** (ESI) calcd for $C_{20}H_{19}O_3NaNS$ $[M+Na]^+$ 376.0978, found 376.0974. **IR** ν_{max} (film): 3649, 2980, 2856, 1591, 1505, 1449, 1299 (SO_2), 1245, 1149 (SO_2), 1131, 1095, 927, 819, 763, 649 cm^{-1}. **M.p.**: 196 – 199 °C.

4-Tolyl 4-methoxyphenyl sulfone 4d

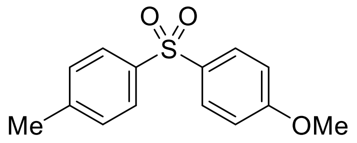

General procedure A was followed with 4-methoxyboronic acid (91 mg, 0.6 mmol, 3.0 eq.), $Cu(MeCN)_4BF_4$ (6.4 mg, 0.02 mmol, 10 mol%), DABSO (72 mg, 0.3 mmol, 1.5 eq.), 4,4'-dimethoxy-bipyridine (4.3 mg, 0.02 mmol, 10 mol%) and 4-iodotoluene (44 mg, 0.2 mmol, 1.0 eq.). The product was purified *via* flash column chromatography (30% Et_2O in petroleum ether) to give the titled product as a white solid (40 mg, 76%).

^{1}**H NMR** (400 MHz, $CDCl_3$) δ 7.82 – 7.76 (m, 2H, H_{Ar}), 7.76 – 7.70 (m, 2H, H_{Ar}), 7.22 – 7.18 (m, 2H, H_{Ar}), 6.90 – 6.85 (m, 2H, H_{Ar}), 3.76 (s, 3H, OCH_3), 2.32 (s, 3H, CCH_3); ^{13}**C NMR** (100 MHz, $CDCl_3$) δ 163.2, 143.7, 139.4, 135.6, 129.8, 129.7, 127.4, 114.4, 55.6, 21.6. **LRMS** (ESI, m/z) 263.1 ($[M+H]^+$, 100%); **HRMS** (ESI) calcd for $C_{14}H_{15}O_3S$ $[M+H]^+$ 263.0736, found 263.0738. **M.p.**: 103 – 104 °C (lit. 103 – 104 °C). The data recorded are consistent with the literature.[19]

4-Tolyl 4-methylthiophenyl sulfone 4e

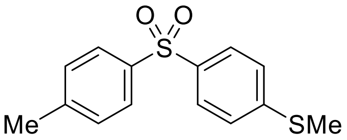

General procedure A was followed with 4-methylthiophenylboronic acid (101 mg, 0.6 mmol, 3.0 eq.), $Cu(MeCN)_4BF_4$ (6.4 mg, 0.02 mmol, 10 mol%), DABSO (72 mg, 0.3 mmol, 1.5 eq.), 4,4'-dimethoxy-bipyridine (4.3 mg, 0.02 mmol, 10 mol%) and 4-iodotoluene (44 mg, 0.2 mmol, 1.0 eq.). The product was purified *via* flash column chromatography (30% Et_2O in petroleum ether) to give the titled product as a white solid (38 mg, 68%).

^{1}**H NMR** (400 MHz, $CDCl_3$) δ 7.78 – 7.69 (m, 4H, H_{Ar}), 7.24 – 7.18 (m, 4H, H_{Ar}), 2.42 (s, 3H, SCH_3), 2.32 (s, 3H, CCH_3); ^{13}**C NMR** (100 MHz, $CDCl_3$) δ 146.3, 144.0, 139.0, 137.6, 129.9, 127.8, 127.5, 125.5, 21.6, 14.7. **LRMS** (ESI, m/z) 279.0 ($[M+H]^+$, 100%), 301.0 ($[M+Na]^+$, 30%); **HRMS** (ESI) calcd for $C_{14}H_{15}O_3S_2$ $[M+H]^+$ 279.0508, found 279.0510. **IR** ν_{max} (film): 3046, 2922, 1580, 1493, 1397, 1314 (SO_2), 1154 (SO_2), 1112, 1085, 817, 757, 661 cm^{-1}. **M.p.**: 136 – 139 °C.

4-Tosylphenol 4f

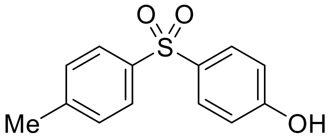

General procedure A was followed with 4-hydroxyphenylboronic acid (132 mg, 0.6 mmol, 3.0 eq.), $Cu(MeCN)_4BF_4$ (6.4 mg, 0.02 mmol, 10 mol%), DABSO (72 mg, 0.3 mmol, 1.5 eq.), 4,4'-dimethoxy-bipyridine (4.3 mg, 0.02 mmol, 10 mol%) and 4-iodotoluene (44 mg, 0.2 mmol, 1.0 eq.). The product was purified *via* flash column chromatography (40% EtOAc in petroleum ether) to give the titled product as a white solid (38 mg, 78%) with less than 2% of inseparable impurity.

^{1}**H NMR** (400 MHz, $CDCl_3$) δ 7.73 – 7.68 (m, 4H, H_{Ar}), 7.22 – 7.19 (m, 2H, H_{Ar}), 6.83 (d, *J* = 8.9 Hz, 2H, H_{Ar}), 6.32 (b, 1H, OH), 2.32 (s, 3H, CCH_3); ^{13}**C NMR** (100 MHz, $CDCl_3$) δ 160.2, 144.0, 139.0, 133.1, 129.9, 129.9, 127.3, 116.1, 21.6. **LRMS** (ESI, m/z) 249.1 ($[M+H]^+$, 100%), 271.0 ($[M+Na]^+$, 40%); **HRMS** (ESI) calcd for $C_{13}H_{13}O_3S$ $[M+H]^+$ 249.0580, found 249.0583. **M.p.**: 137 – 139 °C (lit. 138 °C). The data recorded are consistent with the literature.[9]

***N*,*N*-Dimethyl-4-tosylaniline 4g**

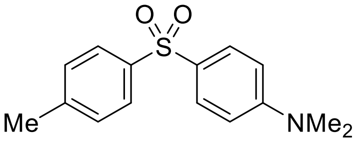

General procedure A was followed with 4-(dimethylamino)phenylboronic acid (99 mg, 0.6 mmol, 3.0 eq.), $Cu(MeCN)_4BF_4$ (6.4 mg, 0.02 mmol, 10 mol%), DABSO (72 mg, 0.3 mmol, 1.5 eq.), 4,4'-dimethoxy-bipyridine (4.3 mg, 0.02 mmol, 10 mol%) and 4-iodotoluene (44 mg, 0.2 mmol, 1.0 eq.). The product was purified *via* flash column chromatography (30% EtOAc in petroleum ether) to give the titled product as a white solid (36 mg, 66%).

^{1}**H NMR** (500 MHz, $CDCl_3$) δ 7.70 (d, *J* = 8.1 Hz, 2H, H_{Ar}), 7.67 (d, *J* = 9.1 Hz, 2H, H_{Ar}), 7.17 (d, *J* = 8.1 Hz, 2H, H_{Ar}), 6.57 (d, *J* = 9.1 Hz, 2H, H_{Ar}), 2.94 (s, 6H, $N(CH_3)_2$), 2.32 (s, 3H, CCH_3); ^{13}**C NMR** (125 MHz, $CDCl_3$) δ 153.0, 143.0, 140.5, 129.7, 129.3, 127.0, 127.0, 111.1, 40.1, 21.5. **LRMS** (ESI, m/z) 276.1 ($[M+H]^+$, 100%), 298.0 ($[M+Na]^+$, 30%); **HRMS** (ESI) calcd for $C_{15}H_{18}O_2NS$ $[M+H]^+$ 276.1053, found 276.1054. **M.p.**: 212 – 214 °C (lit. 212 – 213 °C). The data recorded are consistent with the literature.[20]

***tert*-Butyl (4-tosylphenyl)carbamate 4h**

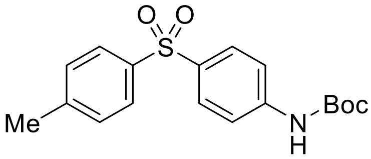

General procedure A was followed with 4-(*N*-Boc-amino)phenylboronic acid (142 mg, 0.6 mmol, 3.0 eq.), $Cu(MeCN)_4BF_4$ (6.4 mg, 0.02 mmol, 10 mol%), DABSO (72 mg, 0.3 mmol, 1.5 eq.), 4,4'-dimethoxy-bipyridine (4.3 mg, 0.02 mmol, 10 mol%) and 4-iodotoluene (44 mg, 0.2 mmol, 1.0 eq.). The product was purified *via* flash column chromatography (30% EtOAc in petroleum ether) to give the titled product as a white solid (36 mg, 52%), with less than 2% inseparable impurity.

^{1}**H NMR** (500 MHz, $CDCl_3$) δ 7.77 (d, *J* = 8.8 Hz, 2H, H_{Ar}), 7.72 (d, *J* = 8.3 Hz, 2H, H_{Ar}), 7.40 (d, *J* = 8.8 Hz, 2H, H_{Ar}), 7.20 (d, *J* = 8.3 Hz, 2H, H_{Ar}), 6.60 (br s, 1H, N*H*), 2.32 (s, 3H, CCH_3), 1.44 (s, 9H, $C(CH_3)_3$); ^{13}**C NMR** (125 MHz, $CDCl_3$) δ 152.0, 143.9, 142.8, 139.2, 135.4, 129.9, 129.0, 127.5, 118.1, 81.6, 28.2, 21.6. **LRMS** (ESI, m/z) 370.1 ($[M+Na]^+$, 100%); **HRMS** (ESI) calcd for $C_{18}H_{21}O_4NaS$ $[M+Na]^+$ 370.1084, found 370.1083. **IR** v_{max} (film): 3341 (NH), 2926, 1730 (CO), 1592, 1522, 1403, 1368, 1321 (SO_2), 1232, 1147 (SO_2), 1107, 835, 708, 688, 646 cm^{-1}. **M.p.**: 186 – 188 °C.

_N_-(3-Tosylphenyl)acetamide 4i

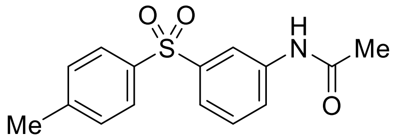

General procedure A was followed with 3-acetamidophenylboronic acid (107 mg, 0.6 mmol, 3.0 eq.), $Cu(MeCN)_4BF_4$ (6.4 mg, 0.02 mmol, 10 mol%), DABSO (72 mg, 0.3 mmol, 1.5 eq.), 4,4'-dimethoxy-bipyridine (4.3 mg, 0.02 mmol, 10 mol%) and 4-iodotoluene (44 mg, 0.2 mmol, 1.0 eq.). The product was purified *via* flash column chromatography (10% EtOAc in petroleum ether) to give the titled product as a white solid (29 mg, 50%).

^{1}**H NMR** (400 MHz, $CDCl_3$) δ 7.92 (d, *J* = 8.1 Hz, 1H, H_{Ar}), 7.88 (bs, 2H, H_{Ar}, O*H*), 7.75 (d, *J* = 8.2 Hz, 2H, H_{Ar}), 7.55 (d, *J* = 7.8 Hz, 1H, H_{Ar}), 7.37 (app. t, *J* = 8.1 Hz, 1H, H_{Ar}), 7.23 (d, *J* = 8.2 Hz, 2H, H_{Ar}), 2.33 (s, 3H, C*H*$_3$), 2.09 (s, 3H, COC*H*$_3$); ^{13}**C NMR** (100 MHz, $CDCl_3$) δ 168.7, 144.5, 142.4, 139.1, 138.2, 130.2, 130.0, 127.7, 124.3, 122.7, 118.1, 24.5, 21.6. **LRMS** (ESI, m/z) 312.1 ($[M+Na]^+$, 100%); **HRMS** (ESI) calcd for $C_{15}H_{15}O_3NNaS$ $[M+Na]^+$ 312.0665, found 312.0662. **IR** ν_{max} (film): 3317 (NH), 2980, 1673 (CO), 1593, 1540, 1478, 1421, 1373 (SO_2), 1301, 1148 (SO_2), 1099, 814, 794, 703, 685 cm^{-1}. **M.p.**: 137 – 140 °C.

1-(Benzyloxy)-4-tosylbenzene 4j

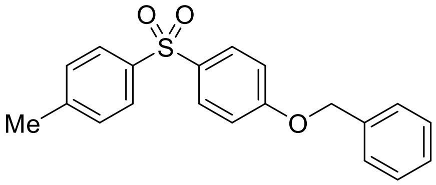

General procedure A was followed with 4-benzyloxyphenylboronic acid (137 mg, 0.6 mmol, 3.0 eq.), $Cu(MeCN)_4BF_4$ (6.4 mg, 0.02 mmol, 10 mol%), DABSO (72 mg, 0.3 mmol, 1.5 eq.), 4,4'-dimethoxy-bipyridine (4.3 mg, 0.02 mmol, 10 mol%) and 4-iodotoluene (44 mg, 0.2 mmol, 1.0 eq.). The product was purified *via* flash column chromatography (10% EtOAc in petroleum ether) to give the titled product as a white solid (38 mg, 57%).

^{1}**H NMR** (400 MHz, $CDCl_3$) δ 7.78 (d, *J* = 9.0 Hz, 2H, H_{Ar}), 7.72 (d, *J* = 8.3 Hz, 2H, H_{Ar}), 7.34 – 7.29 (m, 4H, H_{Ar}), 7.29 – 7.24 (m, 1H, H_{Ar}), 7.20 (d, *J* = 8.1 Hz, 2H, H_{Ar}), 6.95 (d, *J* = 9.0 Hz, 2H, H_{Ar}), 5.02 (s, 2H, C*H*$_2$), 2.31 (s, 3H, C*H*$_3$); ^{13}**C NMR** (100 MHz, $CDCl_3$) δ 162.4, 143.8, 139.4, 135.8, 133.8, 129.9, 129.7, 128.8, 128.4, 127.5, 127.4, 115.3, 70.4, 21.6. **LRMS** (ESI, m/z) 339.1 ($[M+H]^+$, 100%), 361.0 ($[M+Na]^+$, 30%); **HRMS** (ESI) calcd for $C_{20}H_{19}O_3S$ $[M+H]^+$ 339.1049, found 339.1052. **M.p.**: 165 – 167 °C (lit. 201 °C). The data recorded are consistent with the literature.[21]

1-Methoxy-4[(4-tosylphenoxy)methyl]benzene 4k

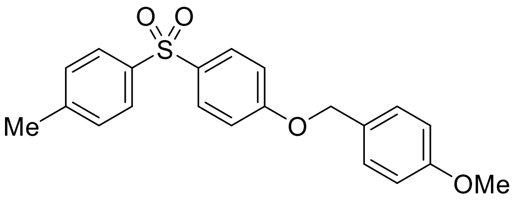

General procedure A was followed with 4-(4-methoxybenzloxy)phenylboronic acid (155 mg, 0.6 mmol, 3.0 eq.), Cu(MeCN)$_4$BF$_4$ (6.4 mg, 0.02 mmol, 10 mol%), DABSO (72 mg, 0.3 mmol, 1.5 eq.), 4,4'-dimethoxy-bipyridine (4.3 mg, 0.02 mmol, 10 mol%) and 4-iodotoluene (44 mg, 0.2 mmol, 1.0 eq.). The product was purified *via* flash column chromatography (10% EtOAc in petroleum ether) to give the titled product as a light yellow solid (46 mg, 63%).

^{1}**H NMR** (400 MHz, CDCl$_3$) δ 7.78 (d, *J* = 9.0 Hz, 2H, H_{Ar}), 7.72 (d, *J* = 8.1 Hz, 2H, H_{Ar}), 7.24 (d, *J* = 8.8 Hz, 2H, H_{Ar}), 7.20 (d, *J* = 8.1 Hz, 2H, H_{Ar}), 6.94 (d, *J* = 9.0 Hz, 2H, H_{Ar}), 6.84 (d, *J* = 8.8 Hz, 2H, H_{Ar}), 4.93 (s, 2H, OCH_2), 3.74 (s, 3H, OCH_3), 2.31 (s, 3H, CH_3); ^{13}**C NMR** (100 MHz, CDCl$_3$) δ 162.4, 159.7, 143.8, 139.4, 133.7, 129.8, 129.7, 129.3, 127.8, 127.4, 115.3, 114.2, 70.2, 55.3, 21.6. **LRMS** (ESI, m/z) 391.1 ([M+Na]$^+$, 100%); **HRMS** (ESI) calcd for $C_{21}H_{20}O_4NaS$ [M+Na]$^+$ 391.0986, found 391.0968. **IR** ν_{max} (film): 3066, 2999, 1593, 1493, 1318 (SO$_2$), 1297, 1247, 1178, 1149 (SO$_2$), 1105, 850, 707, 689, 647 cm^{-1}. **M.p.**: 156 – 159 °C.

1-Chloro-4-[(4-tosylphenoxy)methyl]benzene 4l

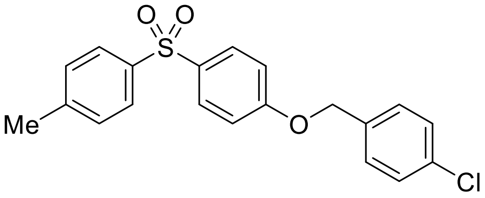

General procedure A was followed with 4-(4-chlorobenzloxy)phenylboronic acid (158 mg, 0.6 mmol, 3.0 eq.), Cu(MeCN)$_4$BF$_4$ (6.4 mg, 0.02 mmol, 10 mol%), DABSO (72 mg, 0.3 mmol, 1.5 eq.), 4,4'-dimethoxy-bipyridine (4.3 mg, 0.02 mmol, 10 mol%) and 4-iodotoluene (44 mg, 0.2 mmol, 1.0 eq.). The product was purified *via* flash column chromatography (10% EtOAc in petroleum ether) to give the titled product as a white solid (42 mg, 56%).

^{1}**H NMR** (500 MHz, CDCl$_3$) δ 7.79 (d, *J* = 8.9 Hz, 2H, H_{Ar}), 7.72 (d, *J* = 8.3 Hz, 2H, H_{Ar}), 7.32-7.23 (m, 4H, H_{Ar}), 7.21 (d, *J* = 8.1 Hz, 2H, H_{Ar}), 6.93 (d, *J* = 8.9 Hz, 2H, H_{Ar}), 4.98 (s, 2H, OCH_2), 2.32 (s, 3H, CH_3); ^{13}**C NMR** (125 MHz, CDCl$_3$) δ 162.1, 143.8, 139.3, 134.3, 134.3, 134.1, 129.9, 129.8, 129.0, 128.8, 127.4, 115.2, 69.6, 21.6. **LRMS** (ESI, m/z) 395.0 ([^{35}M+Na]$^+$, 100%); **HRMS** (ESI) calcd for $C_{20}H_{17}O_3ClNaS$ [^{35}M+Na]$^+$ 395.0479, found 395.0480. **IR** ν_{max} (film): 3440, 2979, 1593, 1494, 1318 (SO$_2$), 1299, 1255, 1152 (SO$_2$), 1106, 1015, 811, 721, 669 cm^{-1}. **M.p.**: 137 – 140 °C.

Trimethyl(4-tosylphenyl)silane 4m

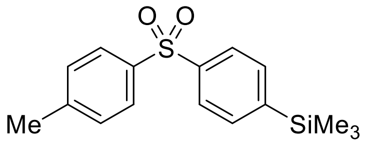

General procedure A was followed with 4-(trimethylsilyl)phenylboronic acid (116 mg, 0.6 mmol, 3.0 eq.), $Cu(MeCN)_4BF_4$ (6.4 mg, 0.02 mmol, 10 mol%), DABSO (72 mg, 0.3 mmol, 1.5 eq.), 4,4'-dimethoxy-bipyridine (4.3 mg, 0.02 mmol, 10 mol%) and 4-iodotoluene (44 mg, 0.2 mmol, 1.0 eq.). The product was purified *via* flash column chromatography (10% EtOAc in petroleum ether) to give the titled product as a white solid (44 mg, 72%).

^{1}**H NMR** (400 MHz, $CDCl_3$) δ 7.77 (d, *J* = 8.2 Hz, 2H, H_{Ar}), 7.72 (d, *J* = 8.3 Hz, 2H, H_{Ar}), 7.52 (d, *J* = 8.3 Hz, 2H, H_{Ar}), 7.18 (d, *J* = 8.2 Hz, 2H, H_{Ar}), 2.28 (s, 3H, CH_3), 0.15 (s, 9H, $Si(CH_3)$)); ^{13}**C NMR** (100 MHz, $CDCl_3$) δ 148.8, 145.5, 143.5, 140.1, 135.5, 131.3, 129.1, 127.7, 23.0, -0.0. **LRMS** (ESI, m/z) 305.1 ($[M+H]^+$, 30%); 327.0 ($[M+Na]^+$, 100%); **HRMS** (ESI) calcd for $C_{16}H_{21}O_2SSi$ $[M+H]^+$ 305.106, found 305.1029. **M.p.**: 101 – 102 °C (lit. 99 °C). The data recorded are consistent with the literature.[22]

4-{4-[(4-Chlorophenyl)sulfonyl]phenyl}morpholine 4n

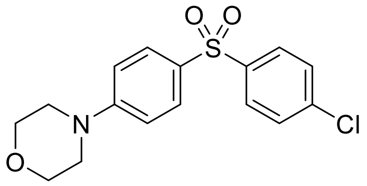

General procedure A was followed with 4-chlorophenylboronic acid (94 mg, 0.6 mmol, 3.0 eq.), $Cu(MeCN)_4BF_4$ (6.4 mg, 0.02 mmol, 10 mol%), DABSO (72 mg, 0.3 mmol, 1.5 eq.), 4,4'-dimethoxy-bipyridine (4.3 mg, 0.02 mmol, 10 mol%) and 4-(4-iodophenyl)morpholine (58 mg, 0.2 mmol, 1.0 eq.). The product was purified *via* flash column chromatography (50% EtOAc in petroleum ether) to give the titled product as a white solid (44 mg, 66%).

^{1}**H NMR** (400 MHz, $CDCl_3$) δ 7.76 (d, *J* = 8.8 Hz, 2H, H_{Ar}), 7.70 (d, *J* = 9.1 Hz, 2H, H_{Ar}), 7.36 (d, *J* = 8.8 Hz, 2H, H_{Ar}), 6.81 (d, *J* = 9.1 Hz, 2H, H_{Ar}), 3.81 – 3.71 (m, 4H, OCH_2), 3.28 – 3.17 (m, 4H, NCH_2); ^{13}**C NMR** (100 MHz, $CDCl_3$) δ 154.2, 141.4, 139.1, 129.5, 129.4, 128.6, 113.8, 66.4, 47.3, one quaternary carbon is not seen on the spectrum. **LRMS** (ESI, m/z) 360.0 ($[M+Na]^+$, 100%); **HRMS** (ESI) calcd for $C_{16}H_{16}O_3NClNaS$ $[M+Na]^+$ 360.0432, found 360.0432. **IR** v_{max} (film): 3086, 2850, 1589, 1506, 1383, 1306 (SO_2), 1245, 1149 (SO_2), 1103, 1012, 926, 823, 767, 615 cm^{-1}. **M.p.**: 140 – 143 °C.

4-{4-[(3-Chloro-4-methoxyphenyl)sulfonyl]phenyl}morpholine 4o

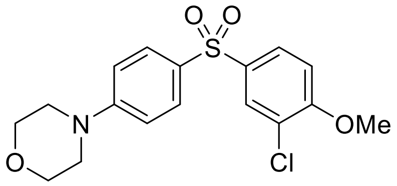

General procedure A was followed with 3-chloro-4-methoxyphenylboronic acid (112 mg, 0.6 mmol, 3.0 eq.), $Cu(MeCN)_4BF_4$ (6.4 mg, 0.02 mmol, 10 mol%), DABSO (72 mg, 0.3 mmol, 1.5 eq.), 4,4'-dimethoxy-bipyridine (4.3 mg, 0.02 mmol, 10 mol%) and 4-(4-iodophenyl)morpholine (58 mg, 0.2 mmol, 1.0 eq.). The product was purified *via* flash column chromatography (50% EtOAc in petroleum ether) to give the titled product as a white solid (50 mg, 68%).

^{1}**H NMR** (500 MHz, $CDCl_3$) δ 7.81 (d, *J* = 2.3 Hz, 1H, H_{Ar}), 7.73 (dd, *J* = 8.7, 2.3 Hz, 1H, H_{Ar}), 7.70 (d, *J* = 9.1 Hz, 2H, H_{Ar}), 6.89 (d, *J* = 8.7 Hz, 1H, H_{Ar}), 6.81 (d, *J* = 9.1 Hz, 2H, H_{Ar}), 3.86 (s, 3H, OCH_3), 3.80-3.73 (m, 4H, OCH_2), 3.25-3.17 (m, 4H, NCH_2); ^{13}**C NMR** (125 MHz, $CDCl_3$) δ 158.3, 154.1, 135.4, 130.1, 129.3, 127.5, 123.4, 113.9, 111.7, 66.5, 56.5, 47.4, one quaternary carbon not observed. **LRMS** (ESI, m/z) 390.1 ([^{35}M+Na]$^+$, 100%); **HRMS** (ESI) calcd for $C_{17}H_{18}O_4NClNaS$ [^{35}M+Na]$^+$ 390.0537, found 390.0539. **IR** ν_{max} (film): 3073, 2921, 2850, 1590, 1490, 1300 (SO_2), 1275, 1246, 1150 (SO_2), 1107, 1062, 927, 821, 762, 609 cm^{-1}. **M.p.**: 157 – 159 °C.

{2-Methoxy-5-[(4-morpholinophenyl)sulfonyl]phenyl}methanol 4p

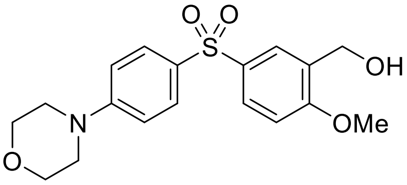

General procedure A was followed with 3-hydroxymethyl-4-methoxyphenylboronic acid (109 mg, 0.6 mmol, 3.0 eq.), $Cu(MeCN)_4BF_4$ (6.4 mg, 0.02 mmol, 10 mol%), DABSO (72 mg, 0.3 mmol, 1.5 eq.), 4,4'-dimethoxy-bipyridine (4.3 mg, 0.02 mmol, 10 mol%) and 4-(4-iodophenyl)morpholine (58 mg, 0.2 mmol, 1.0 eq.). The product was purified *via* flash column chromatography (50% EtOAc in petroleum ether) to give the titled product as a white solid (53 mg, 73%).

^{1}**H NMR** (400 MHz, $CDCl_3$) δ 7.80 (dd, *J* = 8.6, 2.3 Hz, 1H, H_{Ar}), 7.77 (d, *J* = 2.3 Hz, 1H, H_{Ar}), 7.71 (d, *J* = 9.1 Hz, 2H, H_{Ar}), 6.85 (d, *J* = 8.6 Hz, 1H, H_{Ar}), 6.79 (d, *J* = 9.1 Hz, 2H, H_{Ar}), 4.60 (d, *J* = 6.6 Hz, 2H, HOCH_2), 3.82 (s, 3H, OCH_3), 3.78-3.73 (m, 4H, OCH_2), 3.22-3.13 (m, 4H, NCH_2), 2.13 (t, *J* = 6.6 Hz, 1H, O*H*); ^{13}**C NMR** (100 MHz, $CDCl_3$) δ 160.4, 153.9, 134.5, 130.9, 130.3, 129.2, 128.7, 127.4, 113.9, 110.2, 66.5, 61.1, 55.8, 47.5. **LRMS** (ESI, m/z) 364.1 ([M+H]$^+$, 100%), 386.0 ([M+Na]$^+$, 40%); **HRMS** (ESI) calcd for $C_{18}H_{22}O_5NS$ [M+H]$^+$ 364.1213, found 364.1214. **IR** ν_{max} (film): 3457 (OH), 2922, 2852, 1591, 1491, 1450, 1296 (SO_2), 1246, 1190, 1133 (SO_2), 1096, 1048, 821, 762, 684, 608 cm^{-1}. **M.p.**: 194 – 196 °C.

2-Methoxy-5-tosylbenzaldehyde 4q

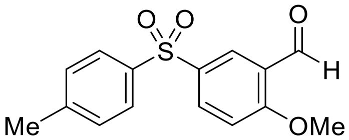

General procedure A was followed with 3-formyl-4-methoxyphenylboronic acid (108 mg, 0.6 mmol, 3.0 eq.), $Cu(MeCN)_4BF_4$ (6.4 mg, 0.02 mmol, 10 mol%), DABSO (72 mg, 0.3 mmol, 1.5 eq.), 4,4'-dimethoxy-bipyridine (4.3 mg, 0.02 mmol, 10 mol%) and 4-iodotoluene (44 mg, 0.2 mmol, 1.0 eq.). The product was purified *via* flash column chromatography (50% EtOAc in petroleum ether) to give the titled product as a white solid (31 mg, 54%).

^{1}**H NMR** (500 MHz, $CDCl_3$) δ 10.34 (s, 1H, C*H*O), 8.27 (d, *J* = 2.5 Hz, 1H, H_{Ar}), 8.06 (dd, *J* = 8.9, 2.5 Hz, 1H, H_{Ar}), 7.75 (d, *J* = 8.2 Hz, 2H, H_{Ar}), 7.23 (d, *J* = 8.2 Hz, 2H, H_{Ar}), 7.02 (d, *J* = 8.9 Hz, 1H, H_{Ar}), 3.92 (s, 3H, OCH_3), 2.32 (s, 3H, CH_3); ^{13}**C NMR** (125 MHz, $CDCl_3$) δ 188.0, 164.5, 144.3, 138.6, 134.7, 134.7, 130.0, 128.8, 127.6, 125.0, 112.4, 56.4, 21.6. **LRMS** (ESI, m/z) 291.0 ([M+H]$^+$, 30%), 313.0 ([M+Na]$^+$, 100%); **HRMS** (ESI) calcd for $C_{15}H_{14}O_4NaNS$ [M+Na]$^+$ 313.0505, found 313.0507. **IR** ν_{max} (film): 2917, 2850, 1684 (CO), 1595, 1485, 1395, 1321 (SO_2), 1278, 1252, 1184, 1153 (SO_2), 1089, 1017, 911, 817, 681 cm^{-1}. **M.p.**: 120 – 123 °C.

4-{4-[(2,3-Dihydrobenzofuran-5-yl)sulfonyl]phenyl}morpholine 4r

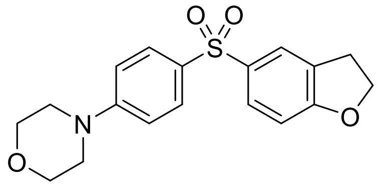

General procedure A was followed with 2,3-dihydrobenzofuran-5-boronic acid (98 mg, 0.6 mmol, 3.0 eq.), $Cu(MeCN)_4BF_4$ (6.4 mg, 0.02 mmol, 10 mol%), DABSO (72 mg, 0.3 mmol, 1.5 eq.), 4,4'-dimethoxy-bipyridine (4.3 mg, 0.02 mmol, 10 mol%) and 4-(4-iodophenyl)morpholine (57.8 mg, 0.2 mmol, 1.0 eq.). The product was purified *via* flash column chromatography (50% EtOAc in petroleum ether) to give the titled product as a white solid (54 mg, 79%).

^{1}**H NMR** (400 MHz, $CDCl_3$) δ 7.70 (d, *J* = 9.1 Hz, 2H, H_{Ar}), 7.66-7.61 (m, 2H, H_{Ar}), 6.80 (d, *J* = 9.1 Hz, 2H, H_{Ar}), 6.73 (d, *J* = 9.0 Hz, 1H, H_{Ar}), 4.56 (t, *J* = 8.8 Hz, 2H, $ArOCH_2CH_2$), 3.78-3.71 (m, 4H, CH_2OCH_2), 3.22-3.07 (m, 6H, NCH_2 and $ArOCH_2CH_2$); ^{13}**C NMR** (100 MHz, $CDCl_3$) δ 163.8, 153.9, 134.4, 131.2, 129.1, 128.8, 128.4, 124.5, 113.9, 109.6, 72.3, 66.5, 47.5, 29.1. **LRMS** (ESI, m/z) 368.1 ([M+Na]$^+$, 100%); **HRMS** (ESI) calcd for $C_{18}H_{19}O_4NaNS$ [M+Na]$^+$ 368.0927, found 368.0923. **IR** ν_{max} (film): 2927, 2835, 1590, 1482, 1381, 1328, 1296 (SO_2), 1239, 1173, 1135 (SO_2), 1124, 1088, 923, 890, 819, 694, 607 cm^{-1}. **M.p.**: 188 – 191 °C.

6-Tosyl-2,3-dihydrobenzo-1,4-dioxine 4s

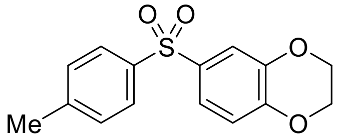

General procedure A was followed with 1,4-benzodioxane-6-boronic acid (108 mg, 0.6 mmol, 3.0 eq.), $Cu(MeCN)_4BF_4$ (6.4 mg, 0.02 mmol, 10 mol%), DABSO (72 mg, 0.3 mmol, 1.5 eq.), 4,4'-dimethoxy-bipyridine (4.3 mg, 0.02 mmol, 10 mol%) and 4-iodotoluene (44 mg, 0.2 mmol, 1.0 eq.). The product was purified *via* flash column chromatography (40% Et_2O in petroleum ether) to give the titled product as a white solid (36 mg, 62%).

^{1}**H NMR** (400 MHz, $CDCl_3$) δ 7.72 (d, *J* = 8.3 Hz, 2H, H_{Ar}), 7.40 – 7.32 (m, 2H, H_{Ar}), 7.21 (d, *J* = 8.1 Hz, 2H, H_{Ar}), 6.85 (d, *J* = 8.3 Hz, 1H, H_{Ar}), 4.25 – 4.15 (m, 4H, OCH_2), 2.32 (s, 3H, CH_3); ^{13}**C NMR** (100 MHz, $CDCl_3$) δ 147.7, 143.8, 143.7, 139.2, 134.4, 129.8, 127.5, 121.3, 118.0, 117.2, 64.5, 64.1, 21.6. **LRMS** (ESI, m/z) 313.1 ([M+Na]$^+$, 100%); **HRMS** (ESI) calcd for $C_{15}H_{14}O_4NaS$ [M+Na]$^+$ 313.0505, found 313.0508. **IR** ν_{max} (film): 3065, 2923, 1496, 1286 (SO_2), 1254, 1150 (SO_2), 1095, 1063, 878, 815, 710, 664 cm^{-1}. **M.p.**: 148 – 151 °C.

1-(Cyclohex-1-en-1-ylsulfonyl)-4-methylbenzene 4t

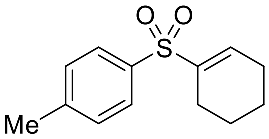

General procedure A was followed with 1-cyclohexen-1-yl-boronic acid (76 mg, 0.6 mmol, 3.0 eq.), $Cu(MeCN)_4BF_4$ (6.4 mg, 0.02 mmol, 10 mol%), DABSO (72 mg, 0.3 mmol, 1.5 eq.), 4,4'-dimethoxy-bipyridine (4.3 mg, 0.02 mmol, 10 mol%) and 4-iodotoluene (44 mg, 0.2 mmol, 1.0 eq.). The product was purified *via* flash column chromatography (20% Et_2O in petroleum ether) to give the titled product as a white solid (27 mg, 57%).

^{1}**H NMR** (400 MHz, $CDCl_3$) δ 7.67 (d, *J* = 7.9 Hz, 2H, H_{Ar}), 7.25 (d, *J* = 7.9 Hz, 2H, H_{Ar}), 6.96 (tt, *J* = 3.8, 1.7 Hz, 1H, C*H*), 2.36 (s, 3H, CH_3), 2.22 – 2.15 (m, 2H, SO_2CCH_2), 2.12 – 2.06 (m, 2H, $SO_2CCHCH2$), 1.62 – 1.54 (m, 2H, SO_2CCHCH_2), 1.52 – 1.46 (m, 2H, $SO_2CCH_2CH_2$); ^{13}**C NMR** (100 MHz, $CDCl_3$) δ 144.0, 140.0, 137.9, 136.5, 129.7, 128.1, 25.5, 22.8, 21.8, 21.6, 20.8. **LRMS** (ESI, m/z) 237.1 ($[M+H]^+$, 100%); **HRMS** (ESI) calcd for $C_{13}H_{17}O_2S$ $[M+H]^+$ 237.0944, found 237.0947. **M.p.**: 66 – 67 °C (lit. 64 – 65 °C). The data recorded are consistent with the literature.[23]

***trans*-1-Methyl-4-(styrylsulfonyl)benzene 4u**

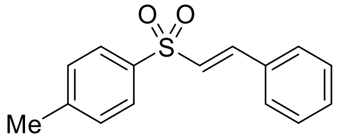

General procedure A was followed with *trans*-2-phenylvinylboronic acid (89 mg, 0.6 mmol, 3.0 eq.), $Cu(MeCN)_4BF_4$ (6.4 mg, 0.02 mmol, 10 mol%), DABSO (72 mg, 0.3 mmol, 1.5 eq.), 4,4'-dimethoxy-bipyridine (4.3 mg, 0.02 mmol, 10 mol%) and 4-iodotoluene (44 mg, 0.2 mmol, 1.0 eq.). The product was purified *via* flash column chromatography (20% Et_2O in petroleum ether) to give the titled product as a white solid (32 mg, 63%).

^{1}**H NMR** (400 MHz, $CDCl_3$) δ 7.76 (d, *J* = 8.3 Hz, 2H, H_{Ar}), 7.59 (d, *J* = 15.4 Hz, 1H, SO_2CH), 7.44 – 7.39 (m, 2H, H_{Ar}), 7.37 – 7.30 (m, 3H, H_{Ar}), 7.27 (d, *J* = 9.3 Hz, 2H, H_{Ar}), 6.78 (d, *J* = 15.4 Hz, 1H, ArC*H*), 2.37 (s, 3H, CH_3); ^{13}**C NMR** (100 MHz, $CDCl_3$) δ 144.4, 142, 137.7, 132.4, 131.1, 130.0, 129.1, 128.5, 127.7, 127.6, 21.7. **LRMS** (ESI, m/z) 259.0 ($[M+H]^+$, 100%); **HRMS** (ESI) calcd for $C_{15}H_{15}O_2S$ $[M+H]^+$ 259.0787, found 259.0790. **M.p.**: 110 – 111 °C (lit. 110 – 112 °C). The data recorded are consistent with the literature.[24]

4. Synthesis of *tert*-butyl 2-(arylsulfonyl)acetate

GENERAL PROCEDURE B for the synthesis of *tert*-butyl 2-(arylsulfonyl)acetate

4-*tert*-Butyl 2-{[4-(*tert*-butyl)phenyl]sulfonyl}acetate 6a

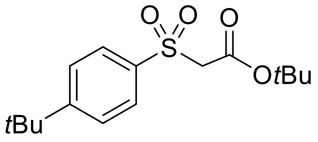

4-*tert*-Butylphenylboronic acid (36 mg, 0.2 mmol, 1.0 eq.), $Cu(MeCN)_4BF_4$ (6.4 mg, 0.02 mmol, 10 mol%), DABSO (24 mg, 0.1 mmol, 0.5 eq.) were mixed and dissolved in DMPU (1 mL) under nitrogen. The reaction mixture was placed in a pre-heated oil bath at 90 °C and stirred for 12 hours prior to cooling to room temperature. Et_3N (42 µL, 0.3 mmol, 1.5 eq.) was then added, and *tert*-butylbromoacetate (59 µL, 0.4 mmol, 2.0 eq.) was immediately injected dropwise. The resultant mixture was stirred at room temperature for 2 hours before being quenched with water (10 mL) and extracted with Et_2O (3 × 10 mL). Combined organic phases were washed with brine (3 × 10 mL), dried over $MgSO_4$, filtered and concentrated *in vacuo*. The crude product was purified *via* flash column chromatography (40% Et_2O in petroleum ether) to give the title compound as a white solid (54 mg, 86%).

^{1}**H NMR** (400 MHz, $CDCl_3$) δ 7.79 (d, *J* = 8.8 Hz, 2H, H_{Ar}), 7.51 (d, *J* = 8.8 Hz, 2H, H_{Ar}), 3.96 (s, 2H, CH_2), 1.28 (s, 18H, 2 × $C(CH_3)_3$); ^{13}**C NMR** (100 MHz, $CDCl_3$) δ 161.4, 158.1, 135.9, 128.4, 126.2, 83.5, 62.2, 35.3, 31.1, 27.8. **LRMS** (ESI, m/z) 335.0 ($[M+Na]^+$, 100%). **HRMS** (ESI) calcd for $C_{16}H_{24}O_4NaS$ $[M+Na]^+$ 335.1288, found 335.1284. **M.p.**: 82 – 84 °C (lit. 107 – 108 °C). The data recorded are consistent with the literature.[25]

***tert*-Butyl 2-(phenylsulfonyl)acetate 6b**

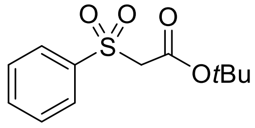

General procedure B was followed with phenylboronic acid (24 mg, 0.2 mmol, 1.0 eq.), $Cu(MeCN)_4BF_4$ (6.4 mg, 0.02 mmol, 10 mol%), DABSO (24 mg, 0.1 mmol, 0.5 eq.), Et_3N (42 µL, 0.3 mmol, 1.5 eq.) and *tert*-butylbromoacetate (59 µL, 0.4 mmol, 2.0 eq. The crude product was purified *via* flash column chromatography (40% Et_2O in petroleum ether) to give the title compound as a colourless oil (41 mg, 80%).

^{1}**H NMR** (400 MHz, $CDCl_3$) δ 7.88 (dd, *J* = 8.4, 1.3 Hz, 2H, H_{Ar}), 7.65-7.58 (m, 1H, H_{Ar}), 7.55-7.49 (m, 2H, H_{Ar}), 3.97 (s, 2H, CH_2), 1.29 (s, 9H, $C(CH_3)_3$); ^{13}**C NMR** (100 MHz, $CDCl_3$) δ 161.3, 138.9, 134.1, 129.2, 128.6, 83.7, 62.1, 27.7. **LRMS** (ESI, m/z) 279.1 ($[M+Na]^+$, 100%); **HRMS** (ESI) calcd for $C_{12}H_{16}O_4NaS$ $[M+Na]^+$ 279.0662, found 279.0662. The data recorded are consistent with the literature.[26]

***tert*-Butyl 2-{[4-(benzyloxy)phenyl]sulfonyl}acetate 6c**

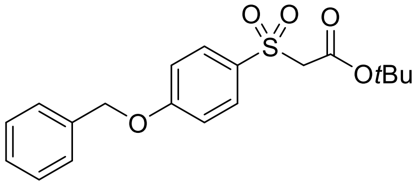

General procedure B was followed with 4-(benzyloxy)phenylboronic acid (46 mg, 0.2 mmol, 1.0 eq.), $Cu(MeCN)_4BF_4$ (6.4 mg, 0.02 mmol, 10 mol%), DABSO (24 mg, 0.1 mmol, 0.5 eq.), Et_3N (42 µL, 0.3 mmol, 1.5 eq.) and *tert*-butylbromoacetate (59 µL, 0.4 mmol, 2.0 eq.). The crude product was purified *via* flash column chromatography (50% Et_2O in petroleum ether) to give the title compound as a white solid (62 mg, 85%).

^{1}**H NMR** (400 MHz, $CDCl_3$) δ 7.79 (d, *J* = 9.0 Hz, 2H, H_{Ar}), 7.37-7.25 (m, 5H, H_{Ar}), 7.02 (d, *J* = 9.0 Hz, 2H, H_{Ar}), 5.07 (s, 2H, OCH_2), 3.93 (s, 2H, SO_2CH_2), 1.30 (s, 9H, $C(CH_3)_3$); ^{13}**C NMR** (100 MHz, $CDCl_3$) δ 163.2, 161.6, 135.7, 130.9, 130.7, 128.8, 128.5, 127.5, 115.1, 83.5, 70.4, 62.4, 27.7. **LRMS** (ESI, m/z) 361.1 ($[M-H]^-$, 100%); **HRMS** (ESI) calcd for $C_{19}H_{21}O_5S$ $[M-H]^-$ 361.1115, found 361.1115. **IR** ν_{max} (film): 3658, 2980, 1731 (CO), 1593, 1497, 1393, 1326 (SO_2), 1258, 1144 (SO_2), 1086, 954, 834, 723, 699 cm^{-1}. **M.p.**: 81 – 83 °C.

***tert*-Butyl 2-[(4-fluorophenyl)sulfonyl]acetate 6d**

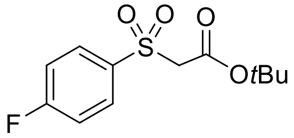

General procedure B was followed with 4-(fluoro)phenylboronic acid (28 mg, 0.2 mmol, 1.0 eq.), $Cu(MeCN)_4BF_4$ (6.4 mg, 0.02 mmol, 10 mol%), DABSO (24 mg, 0.1 mmol, 0.5 eq.), Et_3N (42 µL, 0.3 mmol, 1.5 eq.) and *tert*-butylbromoacetate (59 µL, 0.4 mmol, 2.0 eq.). The crude product was purified *via* flash column chromatography (30% Et_2O in petroleum ether) to give the title compound as a colourless oil (40 mg, 73%).

^{1}**H NMR** (400 MHz, $CDCl_3$) δ 7.96-7.88 (m, 2H, H_{Ar}), 7.24-7.18 (m, 2H, H_{Ar}), 3.97 (s, 2H, CH_2), 1.32 (s, 9H, $C(CH_3)_3$); ^{13}**C NMR** (100 MHz, $CDCl_3$) δ 166.1 (d, $^1J_{C-F}$ = 257 Hz, C_{Ar}), 161.3, 134.9 (d, $^4J_{C-F}$ = 3.3 Hz, C_{Ar}), 131.6 (d, $^3J_{C-F}$ = 9.7 Hz, C_{Ar}), 116.5 (d, $^2J_{C-F}$ = 22.8 Hz, C_{Ar}), 83.8, 62.1, 27.7. ^{19}**F NMR** (376 MHz, $CDCl_3$) δ -102.6. **LRMS** (ESI, m/z) 297 ($[M+H]^+$, 100%). **HRMS** (ESI) calcd for $C_{12}H_{15}O_4NaFS$ $[M+Na]^+$ 297.0567, found 297.0569. The data recorded are consistent with the literature.[25]

***tert*-Butyl 2-{[4-(benzyloxy)phenyl]sulfonyl}acetate 6e**

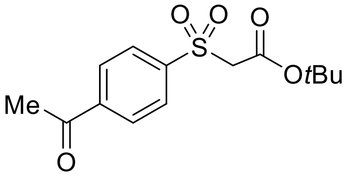

General procedure B was followed with 4-acetylphenylboronic acid (33 mg, 0.2 mmol, 1.0 eq.), $Cu(MeCN)_4BF_4$ (6.4 mg, 0.02 mmol, 10 mol%), DABSO (24 mg, 0.1 mmol, 0.5 eq.), Et_3N (42 µL, 0.3 mmol, 1.5 eq.) and *tert*-butylbromoacetate (59 µL, 0.4 mmol, 2.0 eq.). The crude product was purified *via* flash column chromatography (40% Et_2O in petroleum ether) to give the title compound as a white solid (35 mg, 59%).

^{1}**H NMR** (400 MHz, $CDCl_3$) δ 8.07 (d, *J* = 8.7 Hz, 2H, H_{Ar}), 7.99 (d, *J* = 8.7 Hz, 2H, H_{Ar}), 4.00 (s, 2H, C*H*$_2$), 2.61 (s, 3H, C*H*$_3$), 1.32 (s, 9H, C(C*H*$_3$)$_3$); ^{13}**C NMR** (100 MHz, $CDCl_3$) δ 196.6, 161.1, 142.6, 141.1, 129.0, 128.9, 84.1, 61.9, 27.7, 27.0. **LRMS** (ESI, m/z) 321.1 ([M+H]$^+$, 100%); **HRMS** (ESI) calcd for $C_{14}H_{18}O_5NaS$ [M+H]$^+$ 321.0767, found 321.0768. **M.p.**: 83 – 85 °C (lit. 85 °C). The data recorded are consistent with the literature.[4]

5. Synthesis of *β*-hydroxysulfones

GENERAL PROCEDURE C for the synthesis of *β*-hydroxysulfones

4-{[4-(*tert*-Butyl)phenyl]sulfonyl}tetrahydrofuran-3-ol 6f

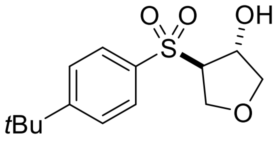

4-*tert*-Butylphenylboronic acid (36 mg, 0.2 mmol, 1.0 eq.), $Cu(MeCN)_4BF_4$ (6.4 mg, 0.02 mmol, 10 mol%), DABSO (24 mg, 0.1 mmol, 0.5 eq.) were mixed and dissolved in DMPU (1 mL) under nitrogen. The reaction mixture was placed in a pre-heated oil bath at 90 °C and stirred for 12 hours prior to cooling to room temperature. Et_3N (42 µL, 0.3 mmol, 1.5 eq.) was then added, and the mixture was diluted with water (1 mL). 3,4-Epoxytetrahydrofuran (35 mg, 0.4 mmol, 2.0 eq.) was injected in a suspension of water (1 mL). The resultant mixture was stirred at room temperature for 2 hours before being quenched with water (10 mL) and extracted with Et_2O (3 × 10 mL). Combined organic phases were washed with brine (3 × 10 mL), dried over $MgSO_4$, filtered and concentrated *in vacuo*. The crude product was purified *via* flash column chromatography (50% Et_2O in petroleum ether) to give the title compound as a white solid (44 mg, 78%).

^{1}**H NMR** (400 MHz, $CDCl_3$) δ 7.76 (d, *J* = 8.7 Hz, 2H, H_{Ar}), 7.53 (d, *J* = 8.7 Hz, 2H, H_{Ar}), 4.86-4.79 (m, 1H, (OH)C*H*), 4.12-3.98 (m, 2H, $CH(SO_2)CH_2$), 3.96-3.87 (m, 1H, $CH(OH)CH_aH_b$), 3.71-3.60 (m, 2H, $CH(OH)CH_aH_b$ and SO_2CH), 2.45 (d, *J* = 5.2 Hz, 1H, O*H*), 1.29 (s, 9H, $C(CH_3)_3$); ^{13}**C NMR** (100 MHz, $CDCl_3$) δ 158.4, 135.1, 128.2, 126.7, 74.8, 72.6, 71.9, 67.2, 35.4, 31.0. **LRMS** (ESI, m/z) 307.1 ($[M+Na]^+$, 100%); **HRMS** (ESI) calcd for $C_{14}H_{20}O_4NaS$ $[M+Na]^+$ 307.0975, found 307.0973. **IR** ν_{max} (film): 3425 (OH), 2980, 2971, 1594, 1463, 1398, 1307 (SO_2), 1291, 1150 (SO_2), 1108, 1082, 967, 840, 757, 631 cm^{-1}. **M.p.**: 109 – 112 °C.

2-(Phenylsulfonyl)cyclohexan-1-ol 6g

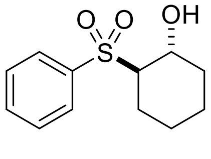

General procedure C was followed with phenylboronic acid (24 mg, 0.2 mmol, 1.0 eq.), $Cu(MeCN)_4BF_4$ (6.4 mg, 0.02 mmol, 10 mol%), DABSO (24 mg, 0.1 mmol, 0.5 eq.), Et_3N (42 µL, 0.3 mmol, 1.5 eq.) and cyclohexene oxide (40 µL, 0.4 mmol, 2.0 eq.). The crude product was purified *via* flash column chromatography (50% Et_2O in petroleum ether) to give the title compound as a white solid (27 mg, 56%).

^{1}**H NMR** (400 MHz, $CDCl_3$) δ 7.87-7.82 (m, 2H, H_{Ar}), 7.66-7.59 (m, 1H, H_{Ar}), 7.56-7.49 (m, 2H, H_{Ar}), 4.23 (d, *J* = 1.1 Hz, 1H, O*H*), 3.89-3.81 (m, 1H, (OH)C*H*), 2.92 (ddd, *J* = 12.4, 9.7, 3.9 Hz, 1H, SO_2CH), 2.11-2.01 (m, 1H, $CH(OH)CH_aH_b$), 1.87-1.80 (m, 1H, $CH(SO_2)CH_aH_b$), 1.70-1.59 (m, 2H, CH_2), 1.30-0.96 (m, 4H, $CH(OH)CH_aH_b$, $CH(SO_2)CH_aH_b$ and CH_2); ^{13}**C NMR** (100 MHz, $CDCl_3$) δ 136.8, 134.2, 129.3, 129.1, 69.0, 68.2, 34.2, 25.7, 24.6, 23.6. **LRMS** (ESI, m/z) 263.0 ($[M+Na]^+$, 100%); **HRMS** (ESI) calcd for $C_{12}H_{16}O_3NaS$ $[M+Na]^+$ 263.0712, found 263.0712. **M.p.**: 103 – 105 °C (lit. 106 – 107 °C). The data recorded are consistent with the literature.[27]

Methyl 3-{[4-(*tert*-butyl)phenyl]sulfonyl}-2-hydroxy-2-methylpropanoate 6h

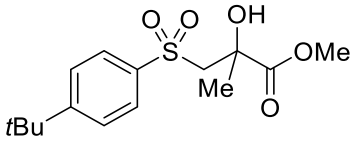

General procedure C was followed with 4-*tert*-butylphenylboronic acid (36 mg, 0.2 mmol, 1.0 eq.), $Cu(MeCN)_4BF_4$ (6.4 mg, 0.02 mmol, 10 mol%), DABSO (24 mg, 0.1 mmol, 0.5 eq.), Et_3N (42 µL, 0.3 mmol, 1.5 eq.) and methyl 2-methylglycidate (42 µL, 0.4 mmol, 2.0 eq. The crude product was purified *via* flash column chromatography (60% Et_2O in petroleum ether) to give the title compound as a white solid (51 mg, 82%).

^{1}**H NMR** (400 MHz, $CDCl_3$) δ 7.74 (d, *J* = 8.7 Hz, 2H, H_{Ar}), 7.49 (d, *J* = 8.7 Hz, 2H, H_{Ar}), 3.75 (s, 1H, O*H*), 3.70 (s, 3H, CO_2CH_3), 3.67 (d, *J* = 14.6 Hz, 1H, $SO_2CH_aH_b$), 3.47 (d, *J* = 14.6 Hz, 1H, $SO_2CH_aH_b$), 1.39 (s, 3H, CCH_3), 1.27 (s, 9H, $C(CH_3)_3$); ^{13}**C NMR** (100 MHz, $CDCl_3$) δ 174.4, 157.8, 137.4, 127.9, 126.2, 72.4, 63.9, 53.4, 35.3, 31.1, 27.2. **LRMS** (ESI, m/z) 337.1 ([M+Na]$^+$, 100%). **HRMS** (ESI) calcd for $C_{15}H_{22}O_5NaS$ [M+Na]$^+$ 337.1080, found 337.1078. **IR** ν_{max} (film): 3497 (OH), 2871, 1743 (CO), 1594, 1494, 1453, 1317 (SO_2), 1291, 1206, 1150 (SO_2), 1108, 1083, 982, 840, 820, 761 cm^{-1}. **M.p.**: 122 – 125 °C.

2-{[4-(*tert*-Butyl)phenyl]sulfonyl}-1-phenylethan-1-ol 6i

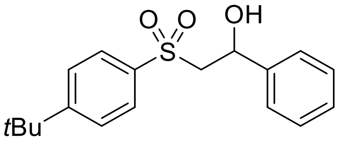

General procedure C was followed with 4-*tert*-butylphenylboronic acid (36 mg, 0.2 mmol, 1.0 eq.), $Cu(MeCN)_4BF_4$ (6.4 mg, 0.02 mmol, 10 mol%), DABSO (24 mg, 0.1 mmol, 0.5 eq.), Et_3N (42 µL, 0.3 mmol, 1.5 eq.) and styrene oxide (46 µL, 0.4 mmol, 2.0 eq.). The crude product was purified *via* flash column chromatography (30% Et_2O in petroleum ether) to give the title compound as a colourless oil which solidified when left standing (39 mg, 61%).

^{1}**H NMR** (400 MHz, $CDCl_3$) δ 7.80 (d, *J* = 8.6 Hz, 2H, H_{Ar}), 7.52 (d, *J* = 8.6 Hz, 2H, H_{Ar}), 7.30-7.20 (m, 5H, H_{Ar}), 5.22 (d, *J* = 10.1 Hz, 1H, O*H*), 3.69 (d, *J* = 2.0 Hz, 1H, $SO_2CH_aH_b$), 3.42 (dd, *J* = 14.3, 10.1 Hz, 1H, (OH)C*H*), 3.27 (dd, *J* = 14.3, 1.8 Hz, 1H, $SO_2CH_aH_b$), 1.29 (s, 9H, $C(CH_3)_3$); ^{13}**C NMR** (100 MHz, $CDCl_3$) δ 158.2, 140.7, 136.1, 128.8, 128.3, 127.9, 126.5, 125.7, 68.4, 64.0, 35.4, 31.1. **LRMS** (ESI, m/z) 341.1 ([M+Na]$^+$, 100%); **HRMS** (ESI) calcd for $C_{18}H_{22}O_3NaS$ [M+Na]$^+$ 341.1182, found 341.1170. **IR** ν_{max} (film): 3497 (OH), 3064, 2961, 1595, 1496, 1455, 1399, 1306 (SO_2), 1289, 1200, 1149 (SO_2), 1108, 1086, 841, 781, 700, 649 cm^{-1}. **M.p.**: 56 – 58 °C.

2-{[4-(*tert*-Butyl)phenyl]sulfonyl}cyclopentan-1-ol 6j

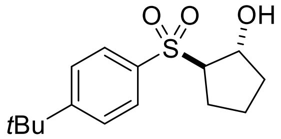

General procedure C was followed with 4-*tert*-butylphenylboronic acid (36 mg, 0.2 mmol, 1.0 eq.), $Cu(MeCN)_4BF_4$ (6.4 mg, 0.02 mmol, 10 mol%), DABSO (24 mg, 0.1 mmol, 0.5 eq.), Et_3N (42 µL, 0.3 mmol, 1.5 eq.) and cyclopentene oxide (35 µL, 0.4 mmol, 2.0 eq.). The crude product was purified *via* flash column chromatography (30% Et_2O in petroleum ether) to give the title compound as a colourless oil which solidified when left standing (45 mg, 79%).

^{1}**H NMR** (400 MHz, $CDCl_3$) δ 7.75 (d, *J* = 8.7 Hz, 2H, H_{Ar}), 7.51 (d, *J* = 8.7 Hz, 2H, H_{Ar}), 4.62 (app. qd, *J* = 6.5, 2.8 Hz, 1H, OHC*H*), 3.29 (td, *J* = 8.7, 6.3 Hz, 1H, SO_2CH), 2.53 (d, *J* = 2.8 Hz, 1H, O*H*), 2.09-1.98 (m, 1H, CH(OH)CH_aH_b), 1.93-1.84 (m, 2H, SO_2CHCH_2), 1.74-1.58 (m, 3H, CH(OH)CH_aH_b, CH(OH)CH_2CH_2), 1.28 (s, 9H, C(CH_3)$_3$); ^{13}**C NMR** (100 MHz, $CDCl_3$) δ 157.8, 135.4, 128.3, 126.4, 73.1, 71.4, 35.3, 34.2, 31.1, 26.1, 21.8. **LRMS** (ESI, m/z) 305.1 ($[M+Na]^+$, 100%); **HRMS** (ESI) calcd for $C_{15}H_{22}O_3NaS$ $[M+Na]^+$ 305.1182, found 305.1184. **IR** ν_{max} (film): 3486 (OH), 2963, 1594, 1399, 1303 (SO_2), 1288, 1146 (SO_2), 1107, 1084, 985, 840, 755, 631 cm^{-1}. **M.p.**: 61 – 64 °C.

6. Synthesis of sulfonamides

GENERAL PROCEDURE D for the synthesis of sulfonamides

4-{[4-(*tert*-Butyl)phenyl]sulfonyl}morpholine 6k

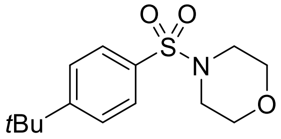

4-*tert*-Butylphenylboronic acid (36 mg, 0.2 mmol, 1.0 eq.), $Cu(MeCN)_4BF_4$ (6.4 mg, 0.02 mmol, 10 mol%), DABSO (24 mg, 0.1 mmol, 0.5 eq.) were mixed and dissolved in DMPU (1 mL) under nitrogen. The reaction mixture was put into a pre-heated oil bath at 90 °C and stirred for 12 hours prior to cooling to room temperature. Et_3N (42 µL, 0.3 mmol, 1.5 eq.) was added, followed by morpholine (35 µL, 0.4 mmol, 2.0 eq.). NaOCl (1.20 mL, 2% aqueous solution w/w, 0.4 mmol, 2.0 eq.) was then added dropwise. The resultant mixture was stirred at room temperature for 3 hours before being quenched with water (10 mL) and extracted with Et_2O (3 × 10 mL). Combined organic phases were washed with brine (3 × 10 mL), dried over $MgSO_4$, filtered and concentrated *in vacuo*. The crude product was purified *via* flash column chromatography (10% EtOAc in petroleum ether) to give the title compound as a white solid (46 mg, 82%).

^{1}**H NMR** (400 MHz, $CDCl_3$) δ 7.61 (d, *J* = 8.6 Hz, 2H, H_{Ar}), 7.48 (d, *J* = 8.6 Hz, 2H, H_{Ar}), 3.73-3.61 (m, 4H, OCH_2), 2.98-2.88 (m, 4H, NCH_2), 1.28 (s, 9H, $C(CH_3)_3$); ^{13}**C NMR** (100 MHz, $CDCl_3$) δ 156.9, 132.0, 127.8, 126.1, 66.1, 46.0, 35.2, 31.1. **LRMS** (ESI, m/z) 284.1 ($[M+H]^+$, 100%); **HRMS** (ESI) calcd for $C_{14}H_{22}O_3S$ $[M+H]^+$ 284.1315, found 284.1316. **M.p.**: 150 – 152 °C. The data recorded are consistent with the literature.[28]

4-(*tert*-Butyl)-*N*-(pyridin-2-ylmethyl)benzenesulfonamide 6l

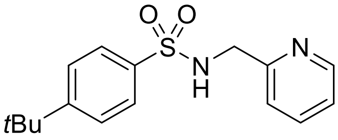

General procedure D was followed with 4-*tert*-butylphenylboronic acid (36 mg, 0.2 mmol, 1.0 eq.), $Cu(MeCN)_4BF_4$ (6.4 mg, 0.02 mmol, 10 mol%), DABSO (24 mg, 0.1 mmol, 0.5 eq.), Et_3N (42 µL, 0.3 mmol, 1.5 eq.), 2-picolylamine (41 µL, 0.4 mmol, 2.0 eq.) and NaOCl (1.2 mL, 2% aqueous solution w/w, 0.4 mmol, 2.0 eq.). The crude product was purified *via* flash column chromatography (10% EtOAc in petroleum ether) to give the title compound as a colourless oil (48 mg, 80%).

^{1}**H NMR** (400 MHz, $CDCl_3$) δ 8.36 (d, *J* = 4.4 Hz, 1H, H_{Ar}), 7.69 (d, *J* = 8.7 Hz, 2H, H_{Ar}), 7.50 (dd, *J* = 7.8, 1.8 Hz, 1H, H_{Ar}), 7.36 (d, *J* = 8.7 Hz, 2H, H_{Ar}), 7.09 (d, *J* = 7.8 Hz, 1H, H_{Ar}), 7.08-7.03 (m, 1H, H_{Ar}), 5.94 (t, *J* = 5.3 Hz, 1H, N*H*), 4.19 (d, *J* = 5.6 Hz, 2H, NCH_2), 1.23 (s, 9H, $C(CH_3)_3$); ^{13}**C NMR** (100 MHz, $CDCl_3$) δ 156.3, 155.0, 149.0, 136.7, 136.5, 127.0, 126.0, 122.6, 122.0, 47.6, 35.1, 31.1. **LRMS** (ESI, m/z) 305.1 ($[M+H]^+$, 100%); **HRMS** (ESI) calcd for $C_{16}H_{21}O_2N_2S$ $[M+H]^+$ 305.1318, found 305.1316. The data recorded are consistent with the literature.[29]

4-(*tert*-Butyl)-*N*-(2,2-dimethoxyethyl)benzenesulfonamide 6m

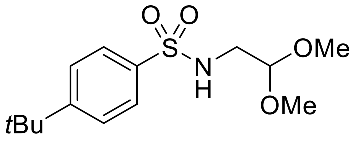

General procedure D was followed with 4-*tert*-butylphenylboronic acid (36 mg, 0.2 mmol, 1.0 eq.), $Cu(MeCN)_4BF_4$ (6.4 mg, 0.02 mmol, 10 mol%), DABSO (24 mg, 0.1 mmol, 0.5 eq.), Et_3N (42 µL, 0.3 mmol, 1.5 eq.), 2,2-dimethoxyethylamine (43 µL, 0.4 mmol, 2.0 eq.) and NaOCl (1.2 mL, 2% aqueous solution w/w, 0.4 mmol, 2.0 eq. The crude product was purified *via* flash column chromatography (10% EtOAc in petroleum ether) to give the title compound as a colourless oil which solidified when left standing (47 mg, 79%).

^{1}**H NMR** (400 MHz, $CDCl_3$) δ 7.71 (d, *J* = 8.5 Hz, 2H, H_{Ar}), 7.45 (d, *J* = 8.5 Hz, 2H, H_{Ar}), 4.56 (t, *J* = 6.2 Hz, 1H, N*H*), 4.27 (t, *J* = 5.6 Hz, 1H, NCH_2CH), 3.26 (s, 6H, OCH_3), 2.98 (t, *J* = 6.0 Hz, 2H, NCH_2), 1.27 (s, 9H, $C(CH_3)_3$); ^{13}**C NMR** (100 MHz, $CDCl_3$) δ 156.6, 136.6, 126.9, 126.2, 102.7, 54.8, 44.6, 35.2, 31.1. **LRMS** (ESI, m/z) 324 ($[M+Na]^+$, 100%); **HRMS** (ESI) calcd for $C_{14}H_{23}O_4NNaS$ $[M+Na]^+$ 324.1240, found 324.1239. **IR** ν_{max} (film): 3278 (NH), 2963, 1596, 1463, 1330 (SO_2), 1197, 1164 (SO_2), 1134, 1112, 1087, 977, 885, 838, 753, 628 cm^{-1}. **M.p.**: 45 – 49 °C.

1-{[4-(*tert*-Butyl)phenyl]sulfonyl}piperidine 6n

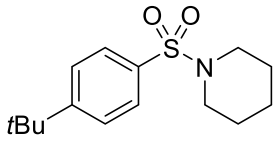

General procedure D was followed with 4-*tert*-butylphenylboronic acid (36 mg, 0.2 mmol, 1.0 eq.), $Cu(MeCN)_4BF_4$ (6.4 mg, 0.02 mmol, 10 mol%), DABSO (24 mg, 0.1 mmol, 0.5 eq.), Et_3N (42 µL, 0.3 mmol, 1.5 eq.), piperidine (39 µL, 0.4 mmol, 2.0 eq.) and NaOCl (1.2 mL, 2% aqueous solution w/w, 0.4 mmol, 2.0 eq.). The crude product was purified *via* flash column chromatography (10% EtOAc in petroleum ether) to give the title compound as a white solid (43 mg, 76%).

^{1}**H NMR** (400 MHz, $CDCl_3$) δ 7.60 (d, *J* = 8.7 Hz, 2H, H_{Ar}), 7.45 (d, *J* = 8.7 Hz, 2H, H_{Ar}), 2.95-2.88 (m, 4H, NCH_2), 1.58 (app. p, *J* = 5.9 Hz, 4H, NCH_2CH_2), 1.39-1.32 (m, 2H, $NCH_2CH_2CH_2$), 1.28 (s, 9H, $C(CH_3)_3$); ^{13}**C NMR** (100 MHz, $CDCl_3$) δ 156.2, 133.3, 127.6, 125.9, 46.9, 35.1, 31.1, 25.2, 23.5. **LRMS** (ESI, m/z) 304.1 ($[M+Na]^+$, 100%); **HRMS** (ESI) calcd for $C_{15}H_{23}O_2NNaS$ $[M+Na]^+$ 304.1346, found 304.1341. **M.p.**: 127 – 130 °C (lit. 114 – 115 °C). The data recorded are consistent with the literature.[30]

4-(*tert*-Butyl)-*N*-(2-phenoxyethyl)benzenesulfonamide 6o

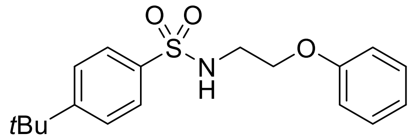

General procedure D was followed with 4-*tert*-butylphenylboronic acid (36 mg, 0.2 mmol, 1.0 eq.), $Cu(MeCN)_4BF_4$ (6.4 mg, 0.02 mmol, 10 mol%), DABSO (24 mg, 0.1 mmol, 0.5 eq.), Et_3N (42 µL, 0.3 mmol, 1.5 eq.), 2-phenoxyethylamine (52 µL, 0.4 mmol, 2.0 eq.) and NaOCl (1.2 mL, 2% aqueous solution w/w, 0.4 mmol, 2.0 eq.). The crude product was purified *via* flash column chromatography (10% EtOAc in petroleum ether) to give the title compound as a colourless oil which solidified when left standing (53 mg, 80%).

^{1}**H NMR** (400 MHz, $CDCl_3$) δ 7.73 (d, *J* = 8.8 Hz, 2H, H_{Ar}), 7.42 (d, *J* = 8.8 Hz, 2H, H_{Ar}), 7.24-7.13 (m, 2H, H_{Ar}), 6.91-6.85 (m, 1H, H_{Ar}), 6.74-6.68 (m, 2H, H_{Ar}), 4.94 (t, *J* = 6.2 Hz, 1H, N*H*), 3.92-3.83 (m, 2H, OCH_2), 3.35-3.25 (m, 2H, NCH_2), 1.26 (s, 9H, $C(CH_3)_3$); ^{13}**C NMR** (100 MHz, $CDCl_3$) δ 158.0, 156.6, 136.9, 129.6, 126.9, 126.2, 121.4, 114.4, 66.2, 42.6, 35.2, 31.1. **LRMS** (ESI, m/z) 356.1 ($[M+Na]^+$, 100%); **HRMS** (ESI) calcd for $C_{18}H_{23}O_3NNaS$ $[M+Na]^+$ 356.1291, found 356.1290. **IR** ν_{max} (film): 3283 (NH), 2962, 1598, 1496, 1398, 1325 (SO_2), 1244, 1161 (SO_2), 1112, 1087, 961, 835, 691, 628 cm^{-1}. **M.p.**: 38 – 41 °C.

7. Synthesis of sulfonyl fluorides

GENERAL PROCEDURE E for the synthesis of sulfonyl fluorides

4-(*tert*-Butyl)benzenesulfonyl fluoride 6p

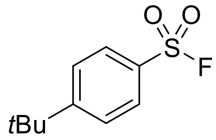

4-*tert*-Butylphenylboronic acid (36 mg, 0.2 mmol, 1.0 eq.), Cu(MeCN)$_4$BF$_4$ (6.4 mg, 0.02 mmol, 10 mol%), DABSO (24 mg, 0.1 mmol, 0.5 eq.) were mixed and dissolved in DMPU (1 mL) under nitrogen. The reaction mixture was put into a pre-heated oil bath at 90 °C and stirred for 12 hours prior to cooling to 0 °C. NFSI (95 mg, 0.3 mmol, 1.5 eq.) was pre-dissolved in DMPU (0.2 mL) and added dropwise. The resultant mixture was warmed to room temperature and stirred for 3 hours before being quenched with water (10 mL) and extracted with Et$_2$O (3 × 10 mL). Combined organic phases were washed with brine (3 × 10 mL), dried over MgSO$_4$, filtered and concentrated *in vacuo*. The crude product was purified *via* flash column chromatography (10% EtOAc in petroleum ether) to give the title compound as a white solid (27 mg, 62%).

^{1}**H NMR** (500 MHz, CDCl$_3$) δ 7.87 (d, *J* = 8.4 Hz, 2H, H_{Ar}), 7.56 (d, *J* = 8.4 Hz, 2H, H_{Ar}), 1.30 (s, 9H, C(CH_3)$_3$); ^{13}**C NMR** (125 MHz, CDCl$_3$) δ 160.0, 130.0 (d, $^2J_{C\text{-}F}$ = 24.2 Hz, C_{Ar}), 128.4, 126.7, 35.6, 31.0; ^{19}**F NMR** (376 MHz, CDCl$_3$) δ 66.2. **HRMS** (CI) calcd for $C_{10}H_{17}FNO_2S$ [M+NH$_4$]$^+$ 234.0964, found 234.0960. **M.p.**: 53 – 55 °C. The data recorded are consistent with the literature.[31]

4-Methoxybenzenesulfonyl fluoride 6q

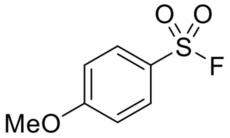

General procedure E was followed with 4-methoxyphenylboronic acid (30 mg, 0.2 mmol, 1.0 eq.), Cu(MeCN)$_4$BF$_4$ (6.4 mg, 0.02 mmol, 10 mol%), DABSO (24 mg, 0.1 mmol, 0.5 eq.) and NFSI (95 mg, 0.3 mmol, 1.5 eq.). The crude product was purified *via* flash column chromatography (10% EtOAc in petroleum ether) to give the title compound as colourless oil (29 mg, 76%).

^{1}**H NMR** (500 MHz, CDCl$_3$) δ 7.88 (d, *J* = 8.9 Hz, 2H, H_{Ar}), 7.00 (d, *J* = 8.9 Hz, 2H, H_{Ar}), 3.85 (s, 3H, OCH_3); ^{13}**C NMR** (125 MHz, CDCl$_3$) δ 165.2, 130.9, 124.2 (d, $^2J_{C\text{-}F}$ = 24.7 Hz, C_{Ar}), 114.9, 55.9; ^{19}**F NMR** (376 MHz, CDCl$_3$) δ 67.3. The data recorded are consistent with the literature.[31]

4-Methylthiobenzenesulfonyl fluoride 6r

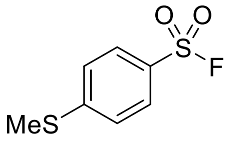

General procedure E was followed with 4-(methylthio)phenylboronic acid (34 mg, 0.2 mmol, 1.0 eq.), $Cu(MeCN)_4BF_4$ (6.4 mg, 0.02 mmol, 10 mol%), DABSO (24 mg, 0.1 mmol, 0.5 eq.) and NFSI (95 mg, 0.3 mmol, 1.5 eq.). The crude product was purified *via* flash column chromatography (10% EtOAc in petroleum ether) to give the title compound as a colourless oil (31 mg, 75%).
^{1}**H NMR** (400 MHz, $CDCl_3$) δ 7.80 (d, *J* = 8.6 Hz, 2H, H_{Ar}), 7.31 (d, *J* = 8.6 Hz, 2H, H_{Ar}), 2.49 (s, 3H, SCH_3); ^{13}**C NMR** (125 MHz, $CDCl_3$) δ 150.3, 128.6, 128.1 (d, $^2J_{C-F}$ = 24.8 Hz, C_{Ar}), 125.4, 14.7; ^{19}**F NMR** (376 MHz, $CDCl_3$) δ 66.8. **HRMS** (CI) calcd for $C_7H_{11}FNO_2S_2$ $[M+NH_4]^+$ 224215, found 224212. **IR** ν_{max} (film): 1576, 1448, 1395 (SO_2), 1210, 1193, 1108 (SO_2), 1081, 818, 769, 738, 626 cm^{-1}.

Naphthalene-2-sulfonyl fluoride 6s

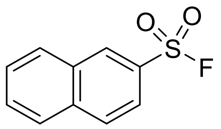

General procedure E was followed with 2-naphthylboronic acid (34 mg, 0.2 mmol, 1.0 eq.), $Cu(MeCN)_4BF_4$ (6.4 mg, 0.02 mmol, 10 mol%), DABSO (24 mg, 0.1 mmol, 0.5 eq.) and NFSI (95 mg, 0.3 mmol, 1.5 eq.). The crude product was purified *via* flash column chromatography (10% EtOAc in petroleum ether) to give the title compound as a white solid (22 mg, 53%).
^{1}**H NMR** (400 MHz, $CDCl_3$) δ 8.57 – 8.54 (m, 1H, H_{Ar}), 8.03-7.95 (m, 2H, H_{Ar}), 7.93 – 7.85 (m, 2H, H_{Ar}), 7.71 – 7.60 (m, 2H, H_{Ar}); ^{13}**C NMR** (125 MHz, $CDCl_3$) δ 136.0, 131.8, 131.0, 130.4, 130.1, 129.8 (d, $^2J_{C-F}$ = 24.9 Hz, C_{Ar}), 129.6, 128.3, 128.2, 122.2; ^{19}**F NMR** (376 MHz, $CDCl_3$) δ 66.3. **IR** ν_{max} (film): 2981, 2889, 1589, 1401 (SO_2), 1217, 1151 (SO_2), 1079, 954, 861, 756, 667 cm^{-1}. **M.p.**: 85 – 87 °C (lit. 85 – 87 °C). The data recorded are consistent with the literature.[31]

4-Hydroxybenzenesulfonyl fluoride 6t

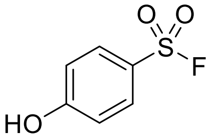

General procedure E was followed with 4-hydroxyphenylboronic acid (28 mg, 0.2 mmol, 1.0 eq.), $Cu(MeCN)_4BF_4$ (6.4 mg, 0.02 mmol, 10 mol%), DABSO (24 mg, 0.1 mmol, 0.5 eq.) and NFSI (95 mg, 0.3 mmol, 1.5 eq.). The crude product was purified *via* flash column chromatography (10% EtOAc in petroleum ether) to give the title compound as a white solid (18 mg, 52%).
^{1}**H NMR** (500 MHz, $CDCl_3$) δ 7.84 (d, *J* = 8.8 Hz, 2H, H_{Ar}), 6.95 (d, *J* = 8.8 Hz, 2H, H_{Ar}), 6.02 (b, 1H, OH); ^{13}**C NMR** (125 MHz, $CDCl_3$) δ 161.8, 131.2, 124.4 (d, $^2J_{C-F}$ = 25.0 Hz, C_{Ar}), 116.4; ^{19}**F NMR** (376 MHz, $CDCl_3$) δ 67.1. **LRMS** (ESI, m/z) 175.0 ($[M-H]^-$, 100%); **HRMS** (ESI) calcd for $C_6H_4O_3FS$ $[M-H]^-$ 174.9871, found 174.9869. **M.p.**: 74 – 75 °C (lit. 74 – 76 °C). The data recorded are consistent with the literature.[32]

8. References:

1. E. J. Emmett, B. R. Hayter and M. C. Willis, *Angew. Chem. Int. Ed.*, 2013, **52**, 12679-12683.
2. N. Fukuda and T. Ikemoto, *J. Org. Chem.*, 2010, **75**, 4629-4631.
3. N. Margraf and G. Manolikakes, *J. Org. Chem.*, 2015, **80**, 2582-2600.
4. E. J. Emmett, B. R. Hayter and M. C. Willis, *Angew. Chem. Int. Ed.*, 2014, **53**, 10204-10208.
5. N. Umierski and G. Manolikakes, *Org. Lett.*, 2013, **15**, 188-191.
6. T. Kawai, Y. Kodera, N. Furukawa, S. Oae, M. Ishida, T. Takeda and S. Wakabayashi, *Phosphorus Sulfur*, 1987, **34**, 139-148.
7. I. K. Khanna, R. M. Weier, Y. Yu, P. W. Collins, J. M. Miyashiro, C. M. Koboldt, A. W. Veenhuizen, J. L. Currie, K. Seibert and P. C. Isakson, *J. Med. Chem.*, 1997, **40**, 1619-1633.
8. W. Zhu and D. W. Ma, *J. Org. Chem.*, 2005, **70**, 2696-2700.
9. B. T. V. Srinivas, V. S. Rawat, K. Konda and B. Sreedhar, *Adv. Synth. Catal.*, 2014, **356**, 805-817.
10. Q. Wu, Y. Luo, A. Lei and J. You, *J. Am. Chem. Soc.*, 2016, **138**, 2885-2888.
11. H. Yang, Y. Li, M. Jiang, J. Wang and H. Fu, *Chem. Eur. J.*, 2011, **17**, 5652-5660.
12. C. Shen, J. Xu, W. Yu and P. Zhang, *Green Chem.*, 2014, **16**, 3007-3012.
13. Y. Fu, W. Zhu, X. Zhao, H. Huegel, Z. Wu, Y. Su, Z. Du, D. Huang and Y. Hu, *Org. Biomol. Chem.*, 2014, **12**, 4295-4299.
14. J. Marquie, A. Laporterie, J. Dubac, N. Roques and J. R. Desmurs, *J. Org. Chem.*, 2001, **66**, 421-425.
15. J. Colomb, G. Becker, S. Fieux, L. Zimmer and T. Billard, *J. Med. Chem.*, 2014, **57**, 3884-3890.
16. M. G. Hoffmann, *Tetrahedron*, 1995, **51**, 9511-9518.
17. P. B. Hopkins and P. L. Fuchs, *J. Org. Chem.*, 1978, **43**, 1208-1217.
18. R. R. Wolff, V. Basava, R. M. Giuliano, W. J. Boyko and J. H. Schauble, *Can. J. Chem.*, 2006, **84**, 667-675.
19. X. Yang, L. Shi and H. Fu, *Synlett*, 2014, **25**, 847-852.
20. S. Cacchi, G. Fabrizi, A. Goggiamani, L. M. Parisi and R. Bernini, *J. Org. Chem.*, 2004, **69**, 5608-5614.
21. B. P. Bandgar, S. V. Bettigeri and J. Phopase, *Org. Lett.*, 2004, **6**, 2105-2108.
22. Bhattach.Sn, C. Eaborn and D. R. M. Walton, *J Chem. Soc. C*, 1969, 1367-&.
23. P. Katrun, S. Chiampanichayakul, K. Korworapan, M. Pohmakotr, V. Reutrakul, T. Jaipetch and C. Kuhakarn, *Eur. J. Org. Chem.*, 2010, 5633-5641.
24. X. W. Li, Y. L. Xu, W. Q. Wu, C. Jiang, C. R. Qi and H. F. Jiang, *Chem. Eur. J.*, 2014, **20**, 7911-7915.
25. A. S. Deeming, C. J. Russell and M. C. Willis, *Angew. Chem. Int. Ed.*, 2016, **55**, 747-750.
26. B. N. Rocke, K. B. Bahnck, M. Herr, S. Lavergne, V. Mascitti, C. Perreault, J. Polivkova and A. Shavnya, *Org. Lett.*, 2014, **16**, 154-157.
27. S. N. Murthy, B. Madhav, V. P. Reddy, K. R. Rao and Y. V. D. Nageswar, *Tetrahedron Lett.*, 2009, **50**, 5009-5011.
28. K. Yang, M. L. Ke, Y. G. Lin and Q. L. Song, *Green Chem.*, 2015, **17**, 1395-1399.
29. S. Dayan, A. Cetin, N. B. Arslan, N. K. Ozpozan, N. Ozdemir and O. Dayan, *Polyhedron*, 2015, **85**, 748-753.
30. W. X. Zhang and M. M. Luo, *Chem. Commun.*, 2016, **52**, 2980-2983.
31. L. Tang, Y. Yang, L. X. Wen, X. K. Yang and Z. Y. Wang, *Green Chem.*, 2016, **18**, 1224-1228.

32. T. Okazaki, K. K. Laali, S. D. Bunge and S. K. Adas, *Eur. J. Org. Chem.*, 2014, **2014**, 1630-1644.

^{1}H NMR, ^{13}C NMR and 19F NMR spectra

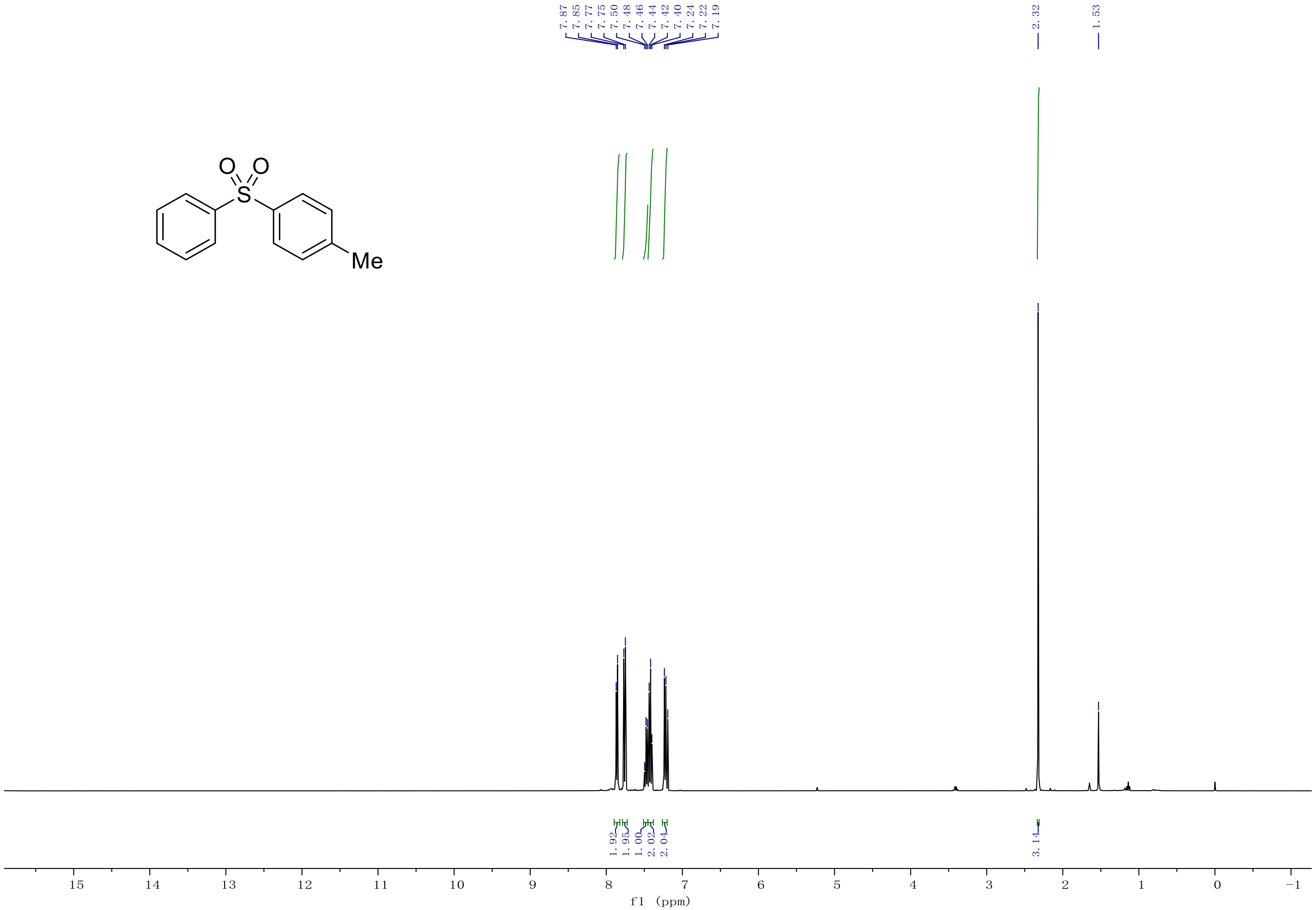

^{1}H NMR spectrum of **phenyl *p*-tolyl sulfone 3a**

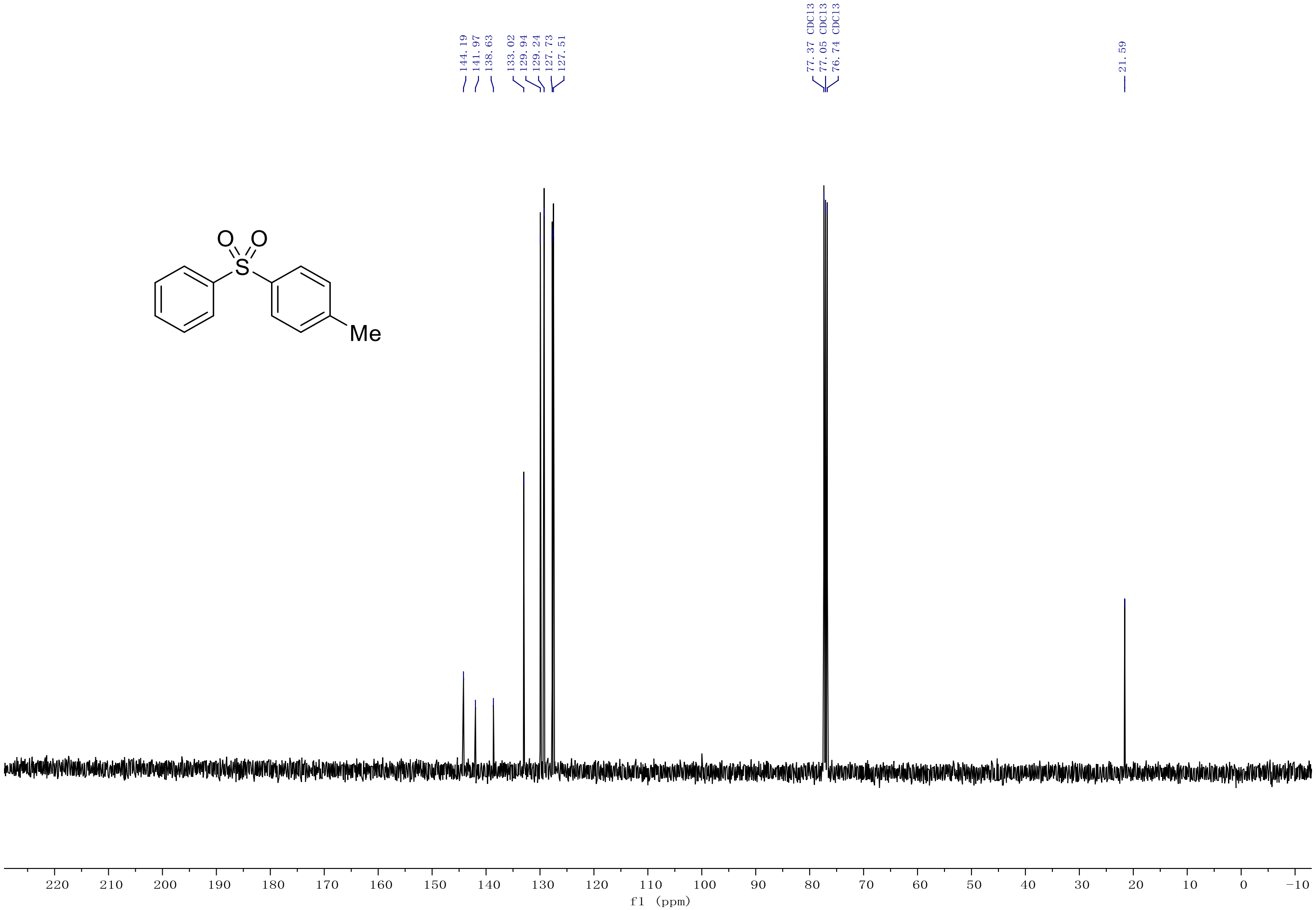

^{13}C NMR spectrum of **phenyl *p*-tolyl sulfone 3a**

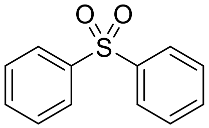

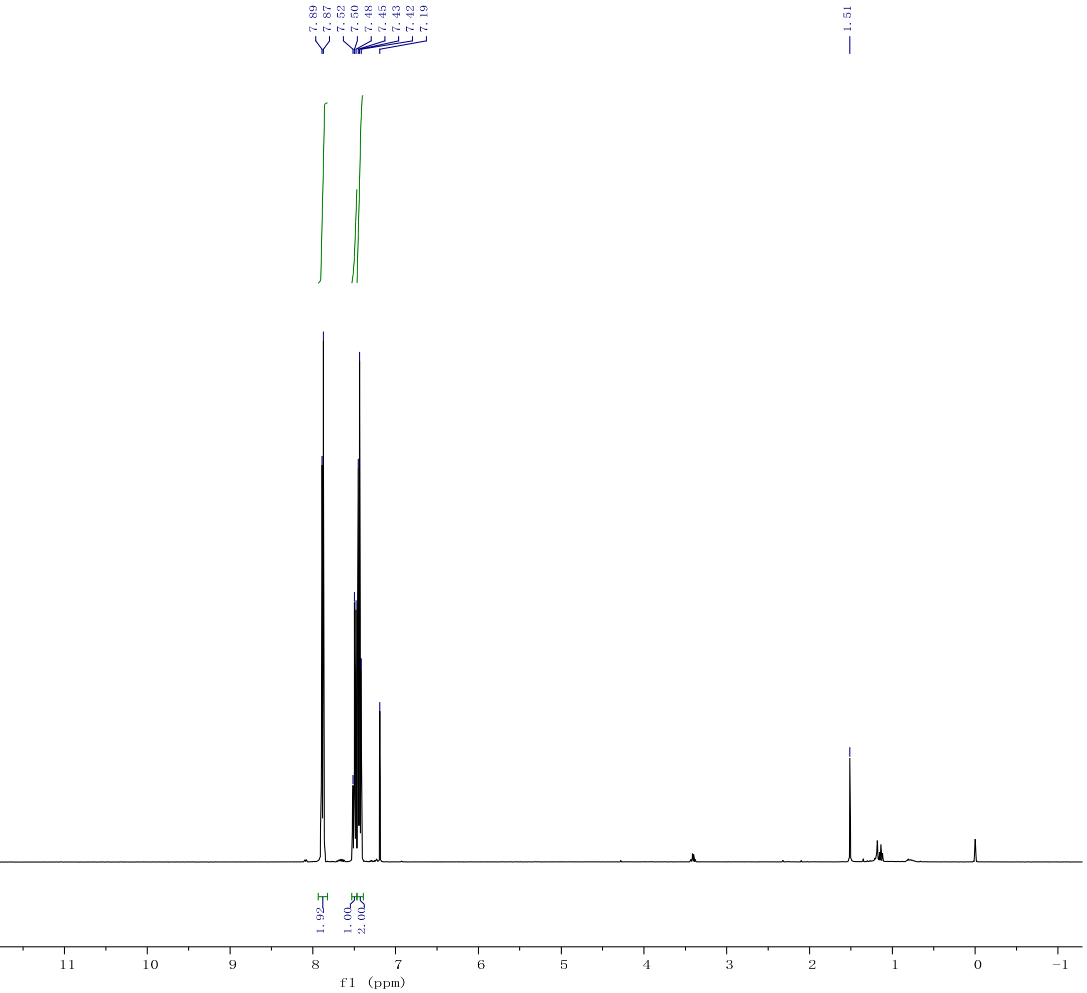

^{1}H NMR spectrum of **diphenyl sulfone 3b**

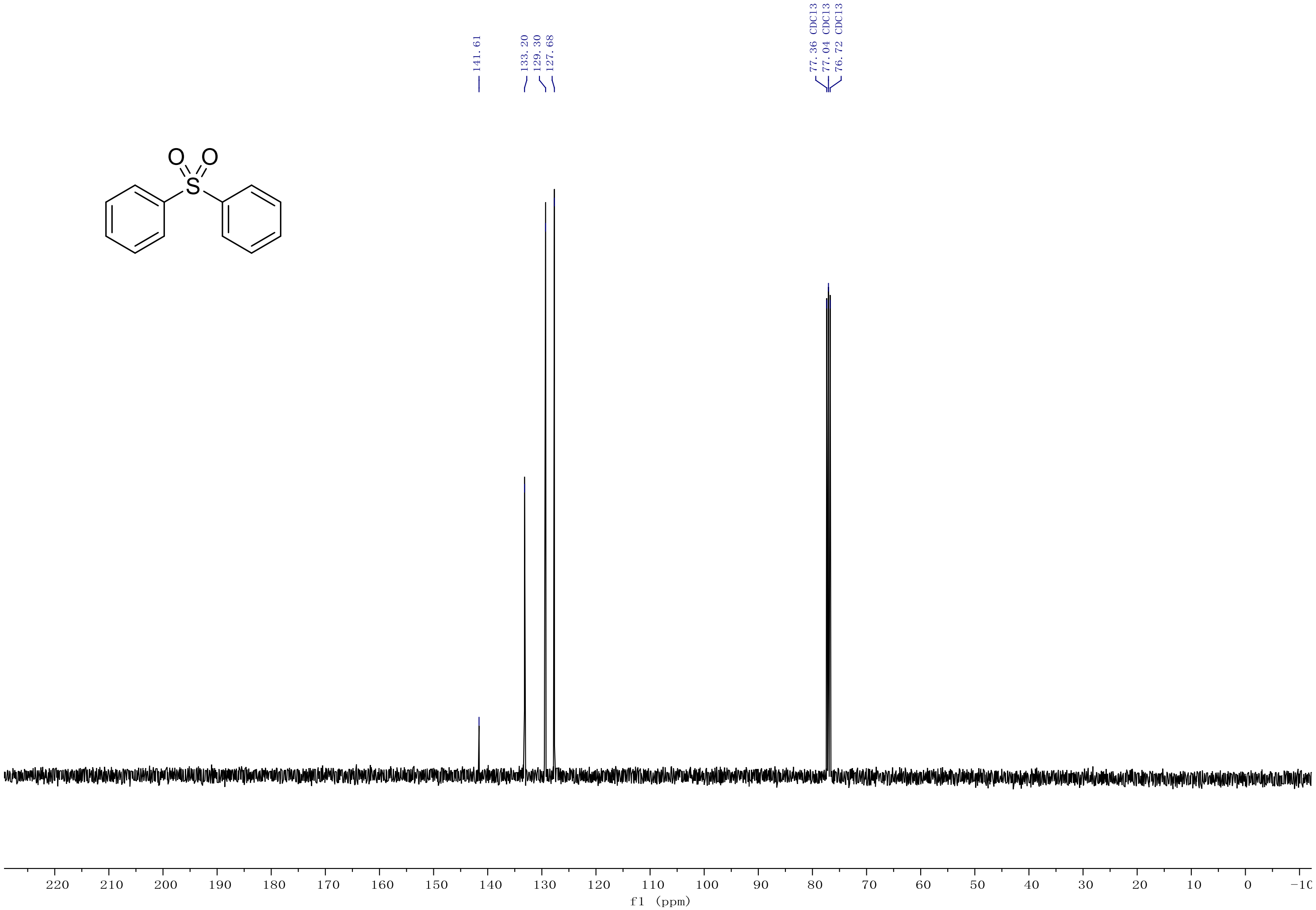

^{13}C NMR spectrum of **diphenyl sulfone 3b**

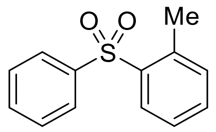

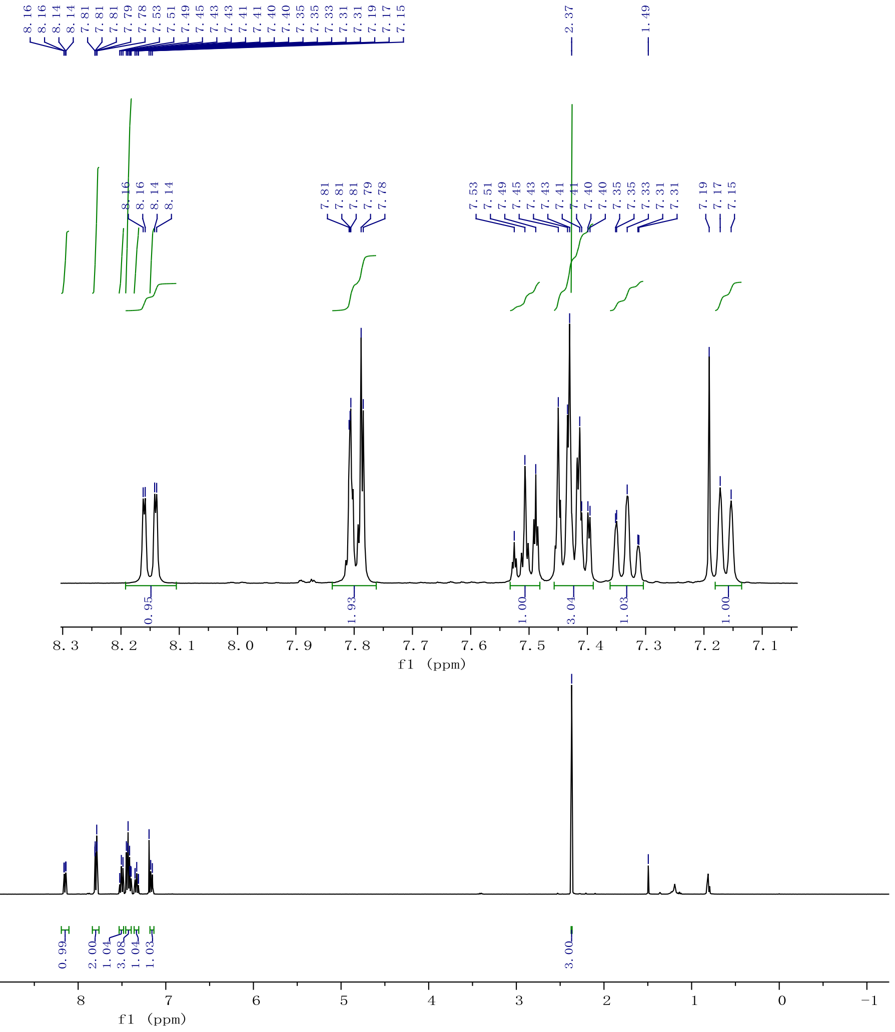

^{1}H NMR spectrum of **phenyl *o*-tolyl sulfone 3c**

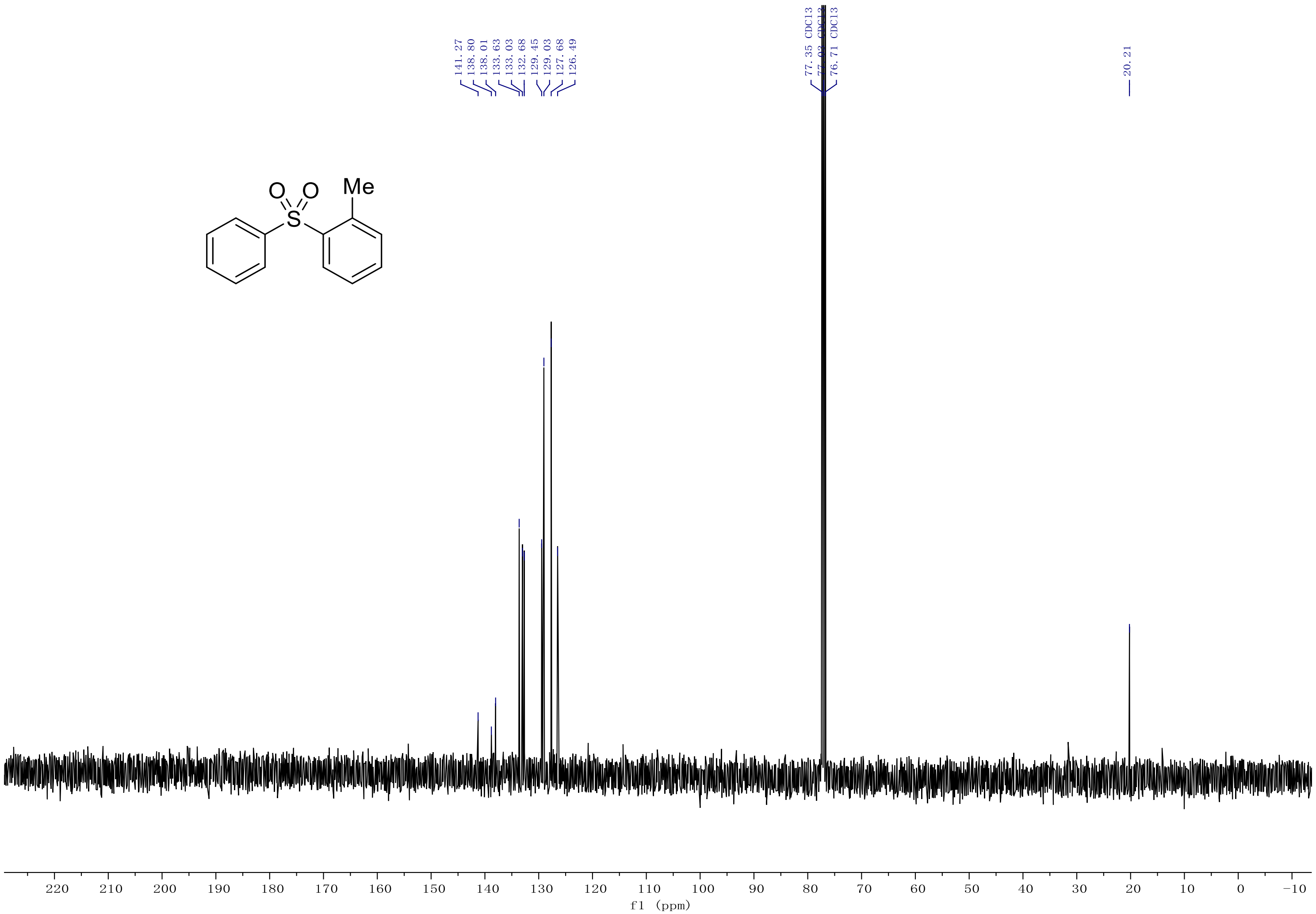

^{13}C NMR spectrum of **phenyl *o*-tolyl sulfone 3c**

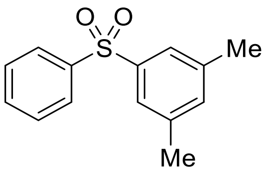

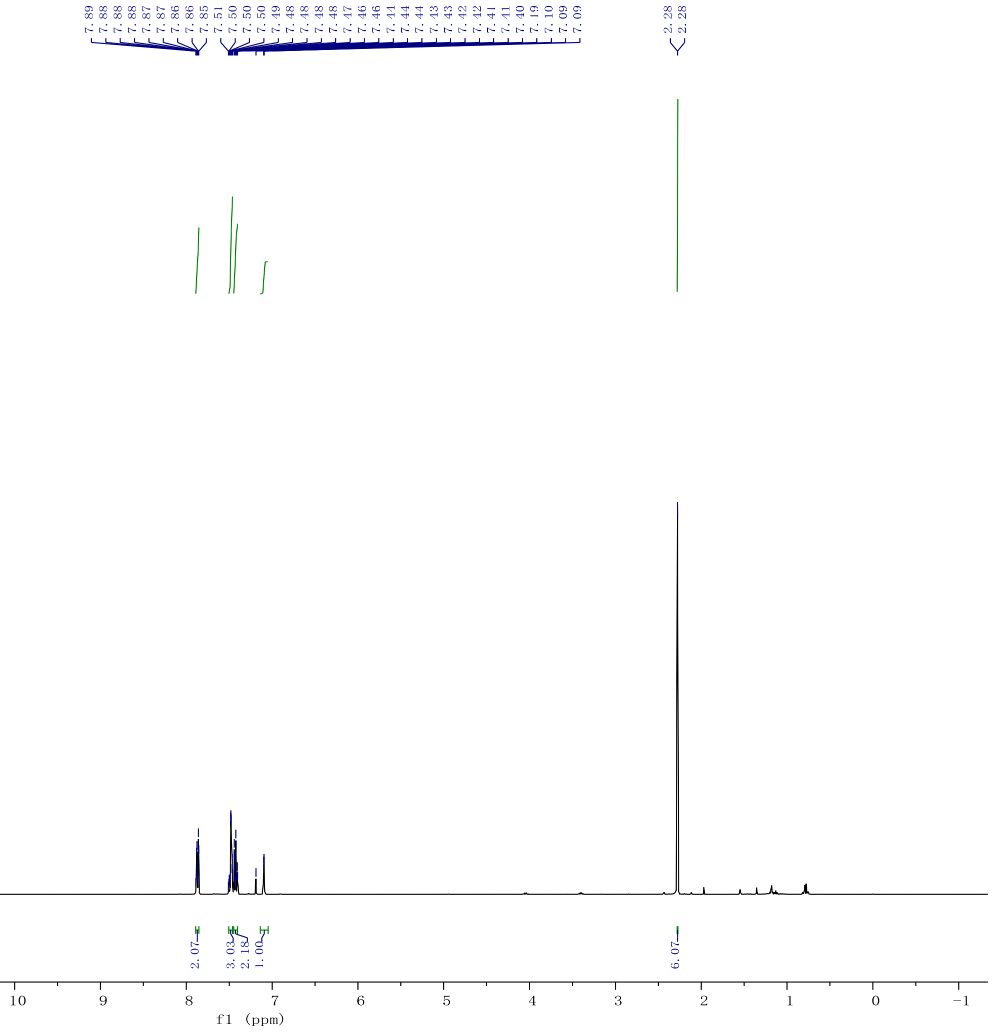

^{1}H NMR spectrum of **3,5-dimethyl-1-(phenylsulfonyl)benzene 3d**

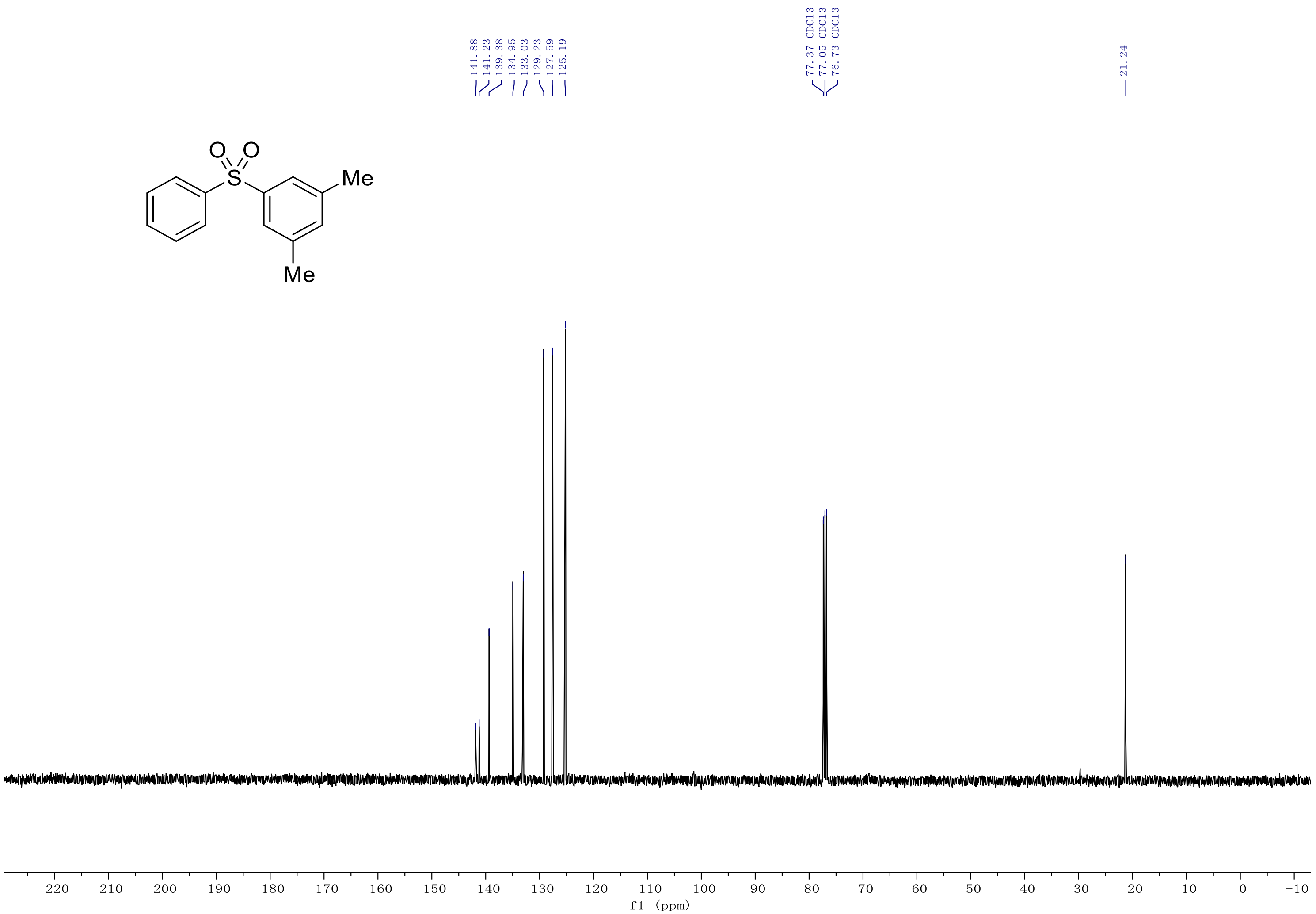

^{13}C NMR spectrum of **3,5-dimethyl-1-(phenylsulfonyl)benzene 3d**

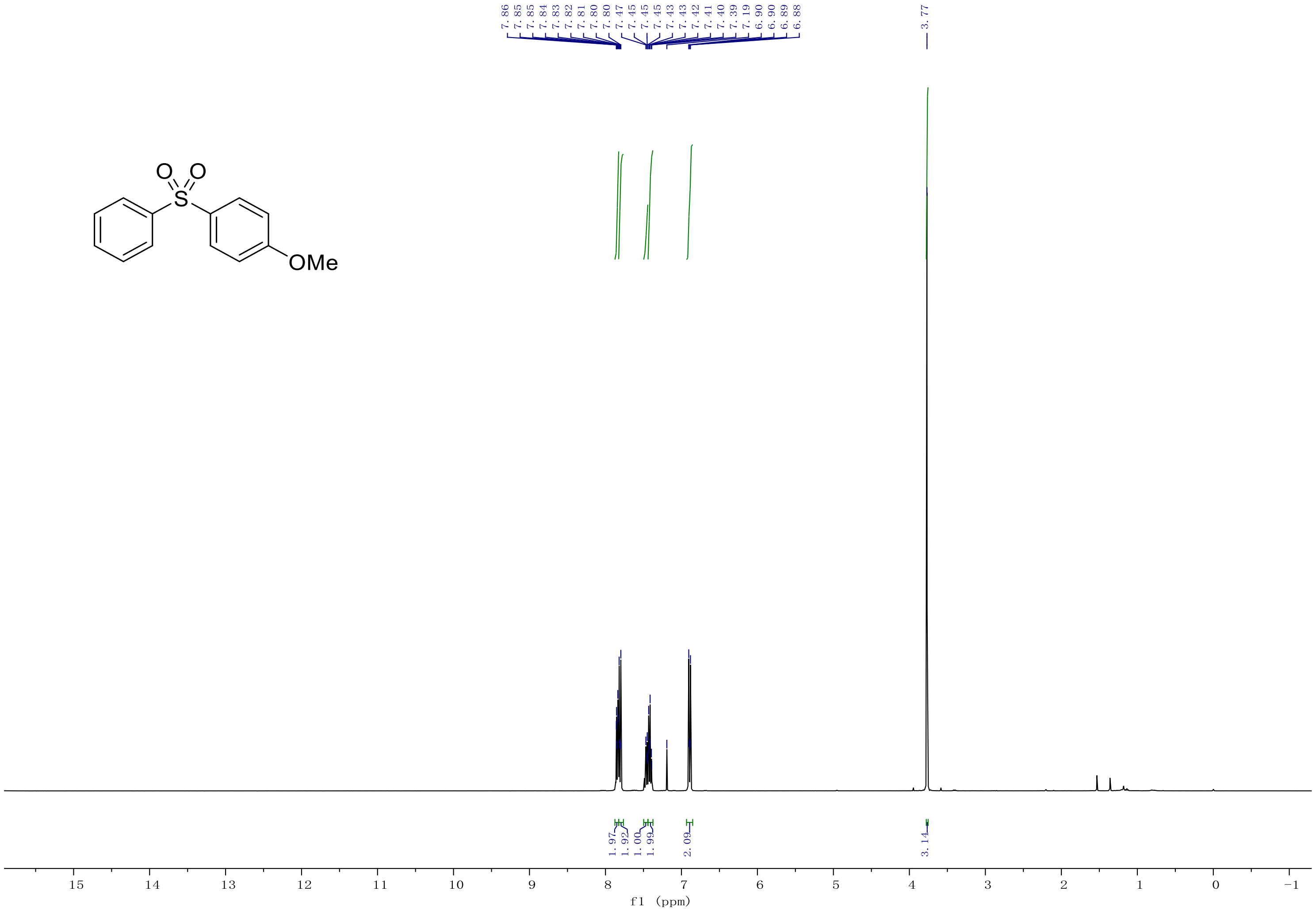

^1H NMR spectrum of **4-methoxyphenyl phenyl sulfone 3e**

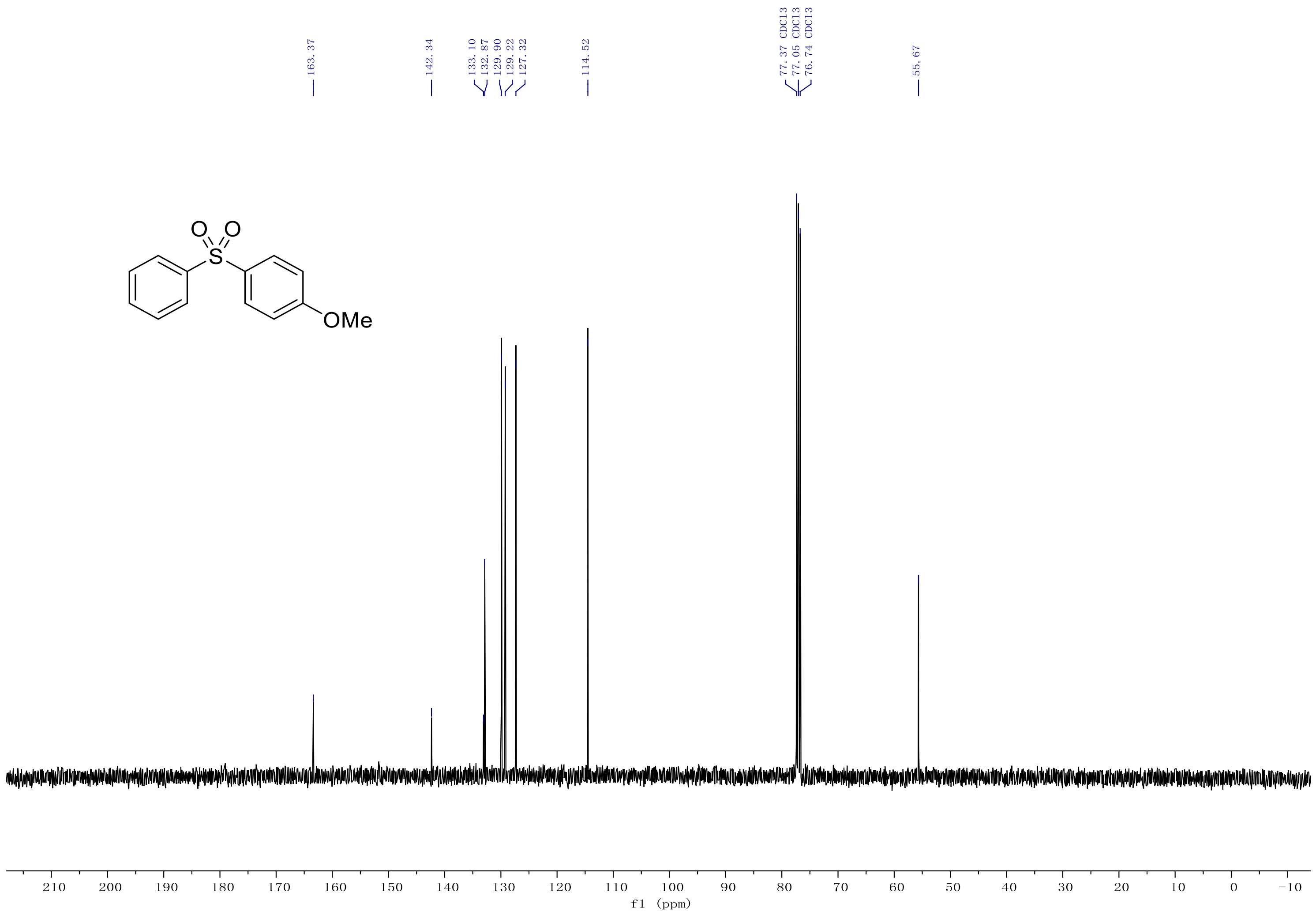

^{13}C NMR spectrum of **4-methoxyphenyl phenyl sulfone 3e**

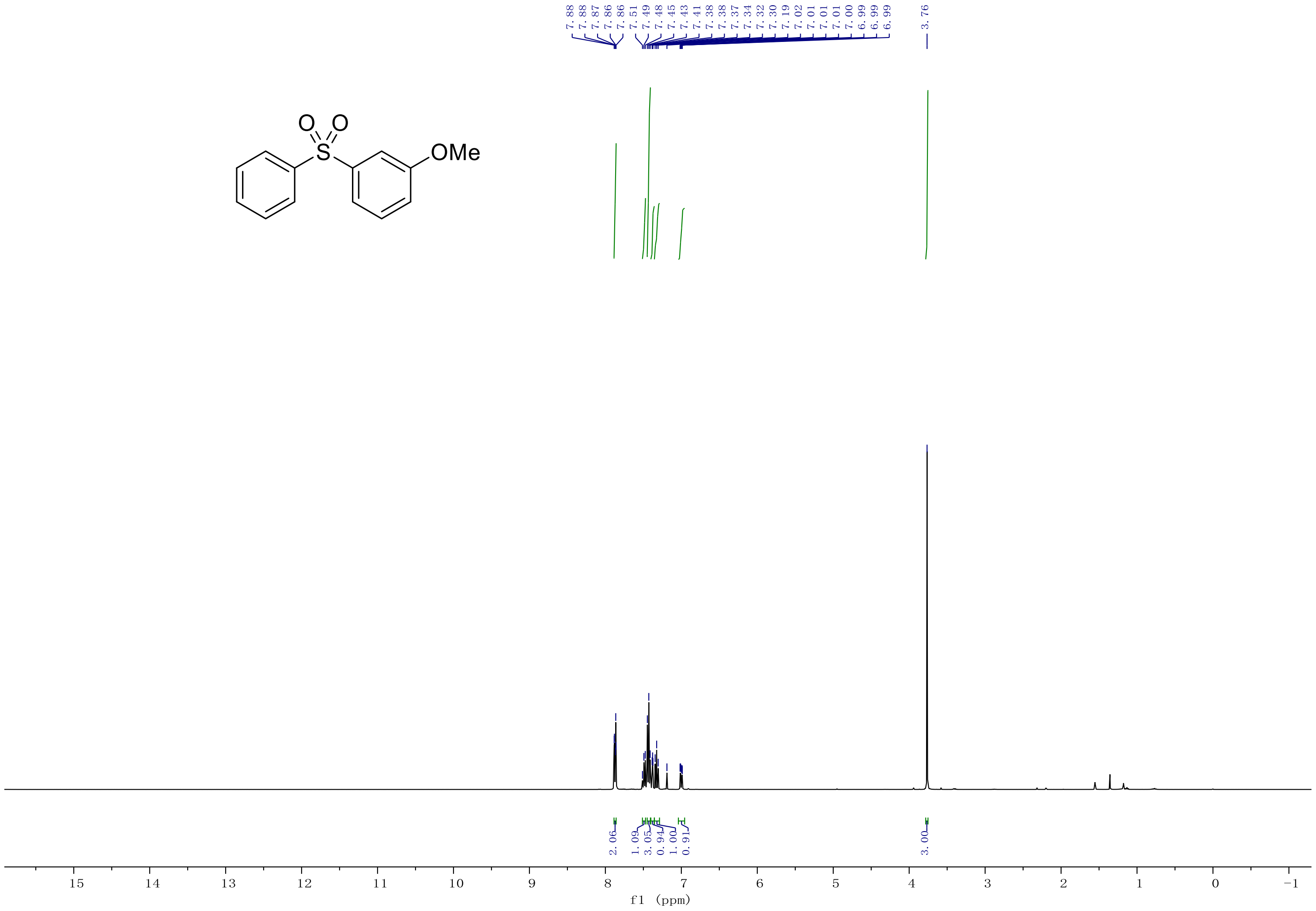

^{1}H NMR spectrum of **3-methoxyphenyl phenyl sulfone 3f**

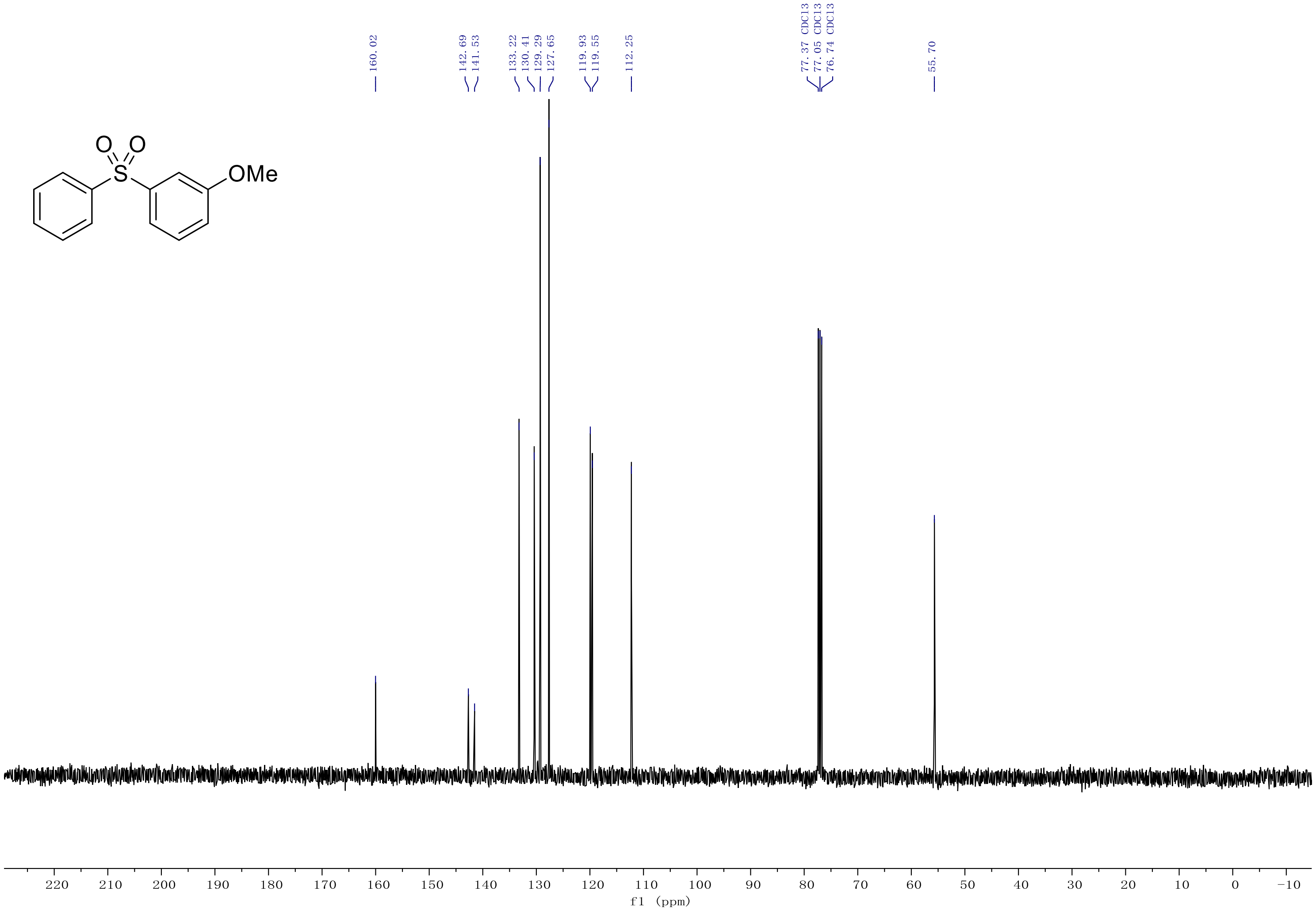

^{13}C NMR spectrum of **3-methoxyphenyl phenyl sulfone 3f**

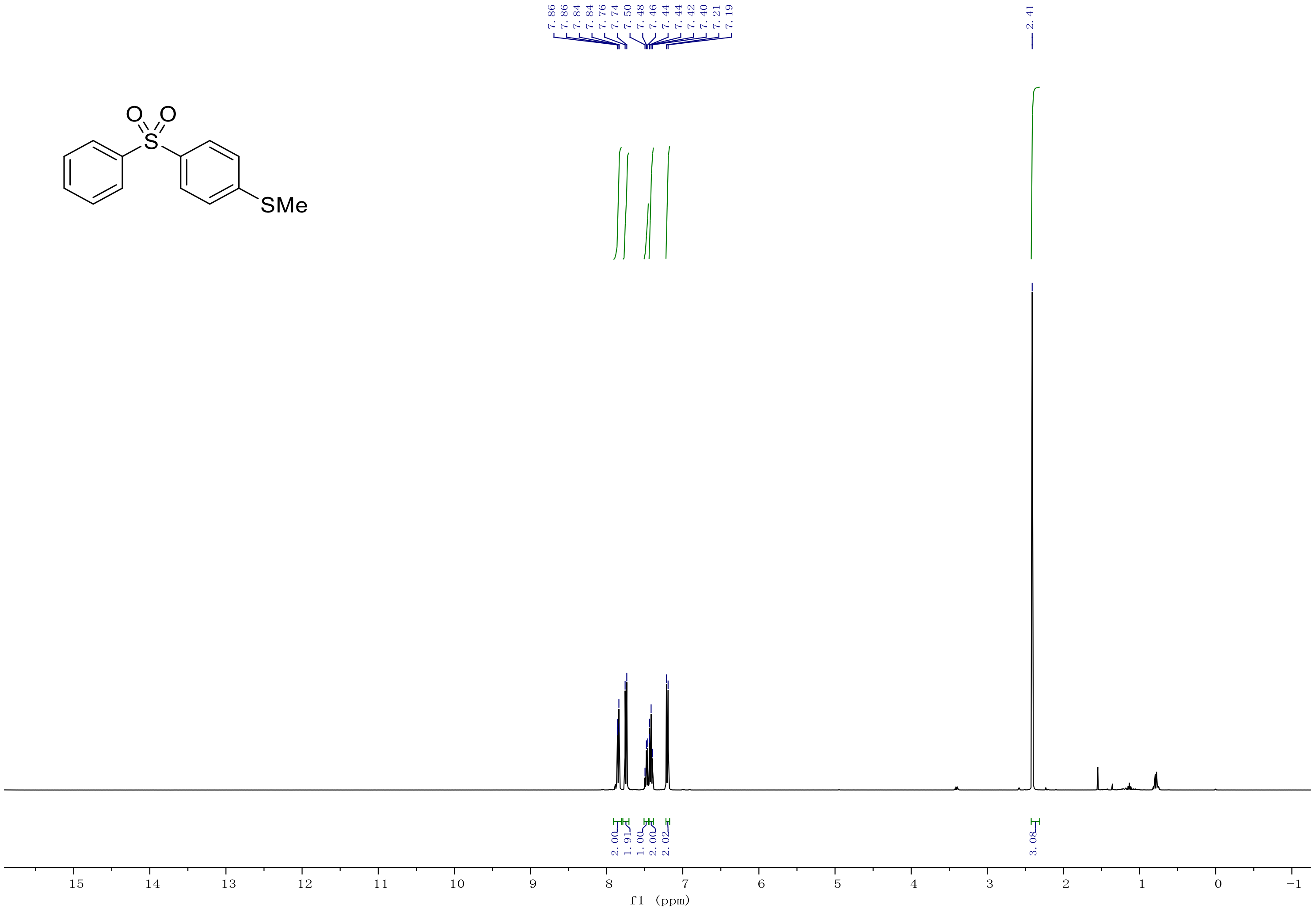

^{1}H NMR spectrum of **4-(benzenesulfonyl)phenyl methyl sulphide 3g**

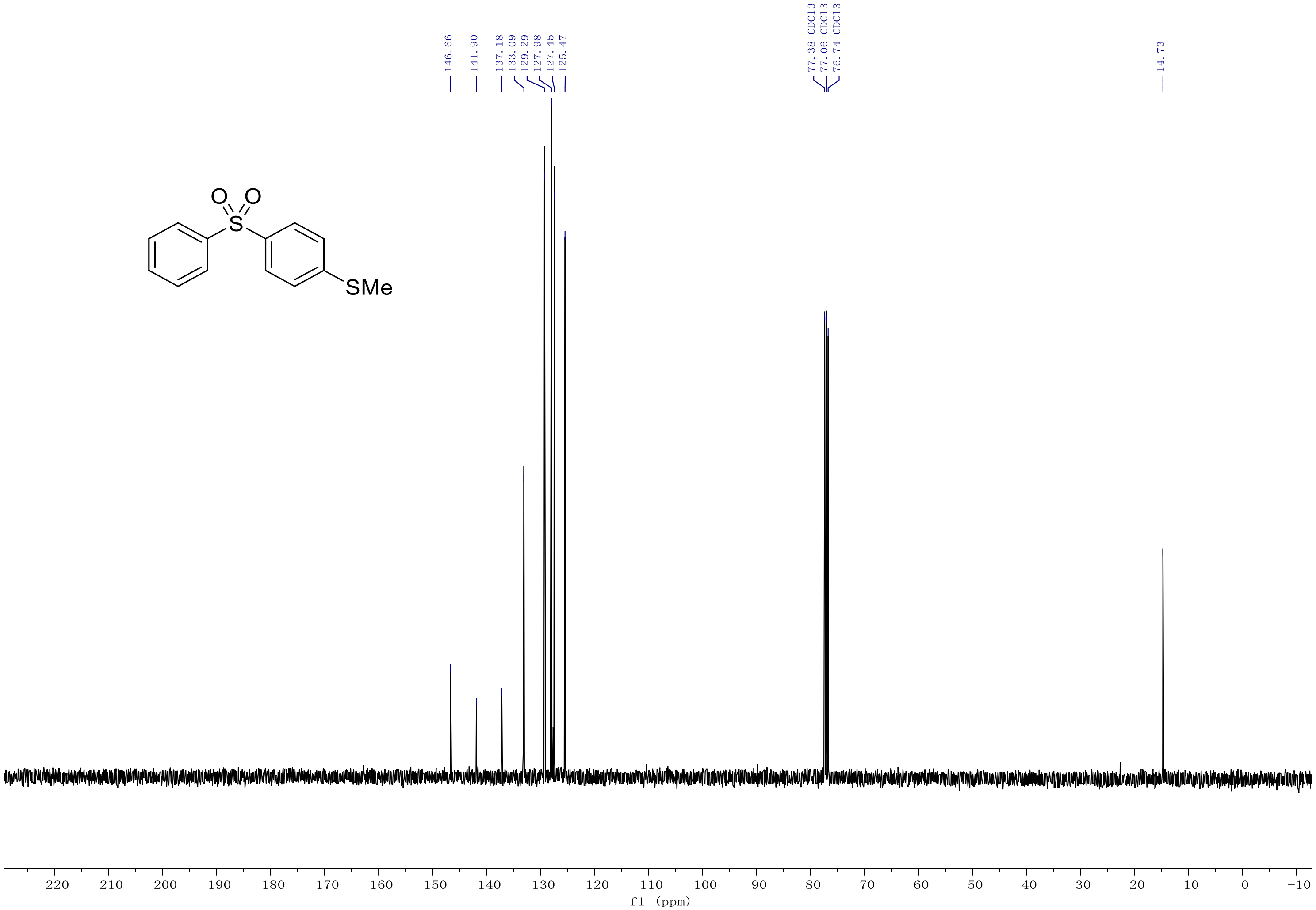

^{13}C NMR spectrum of **4-(benzenesulfonyl)phenyl methyl sulphide 3g**

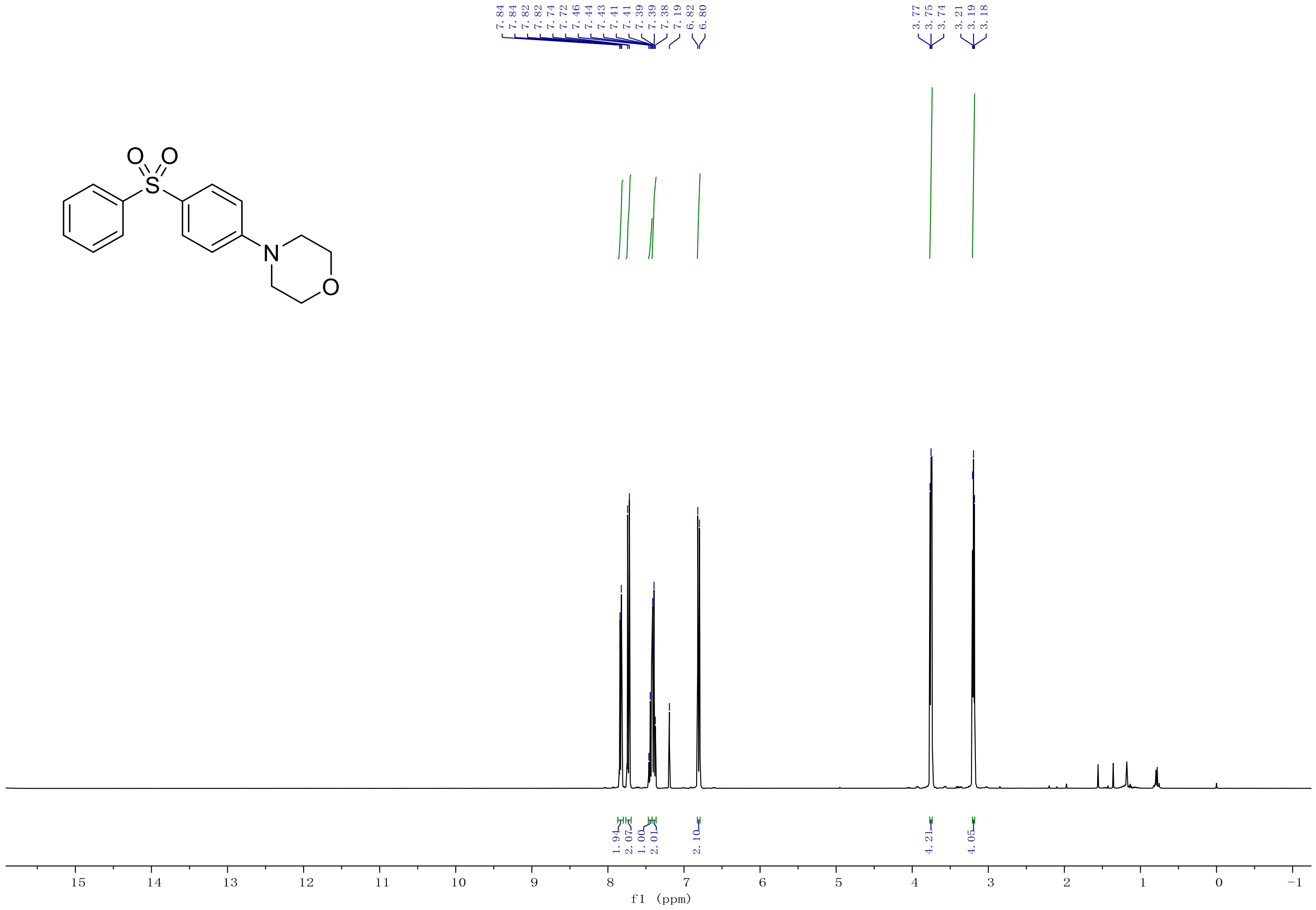

^{1}H NMR spectrum of **4-[4-(phenylsulfonyl)phenyl]morpholine 3h**

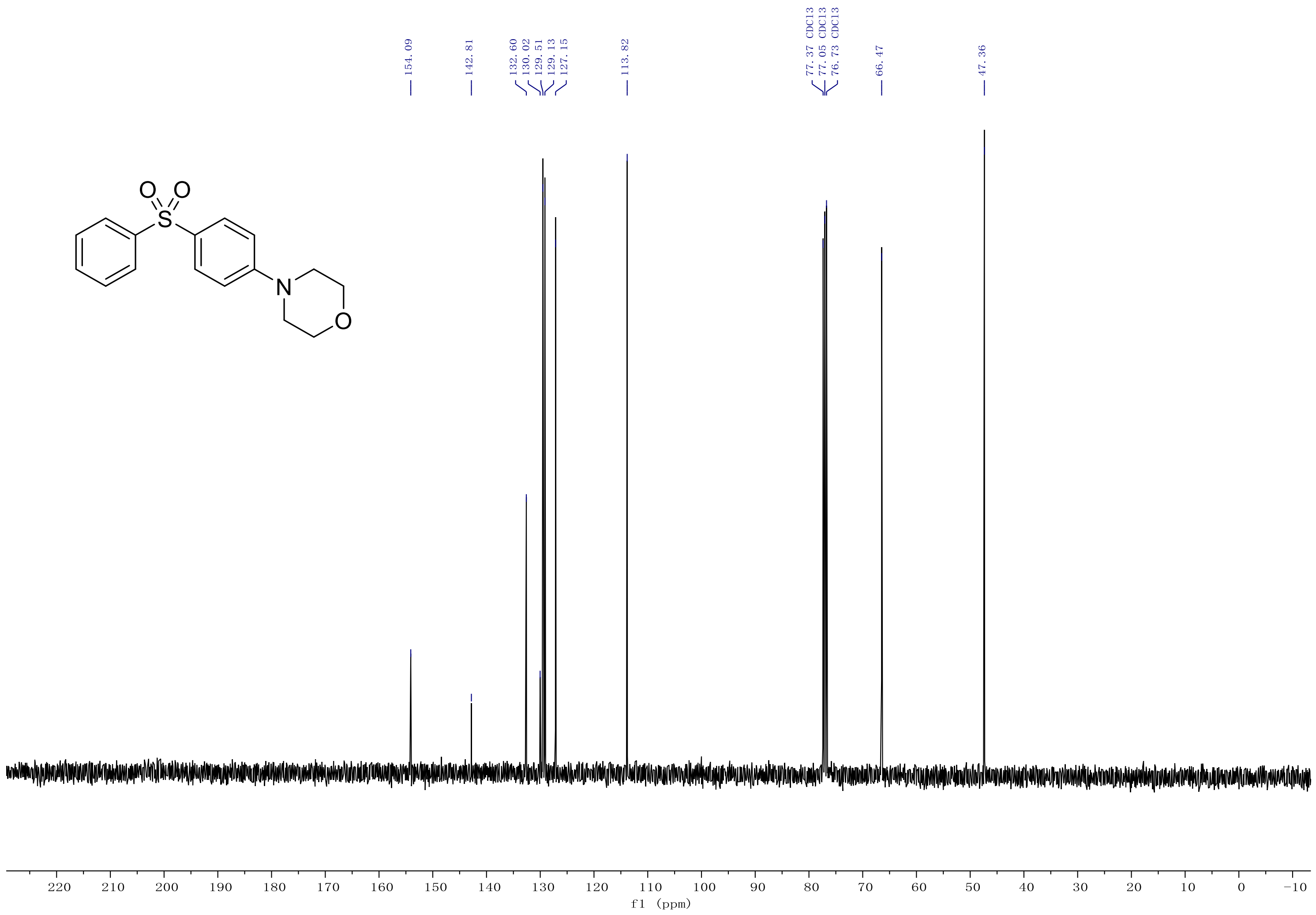

^{13}C NMR spectrum of **4-[4-(phenylsulfonyl)phenyl]morpholine 3h**

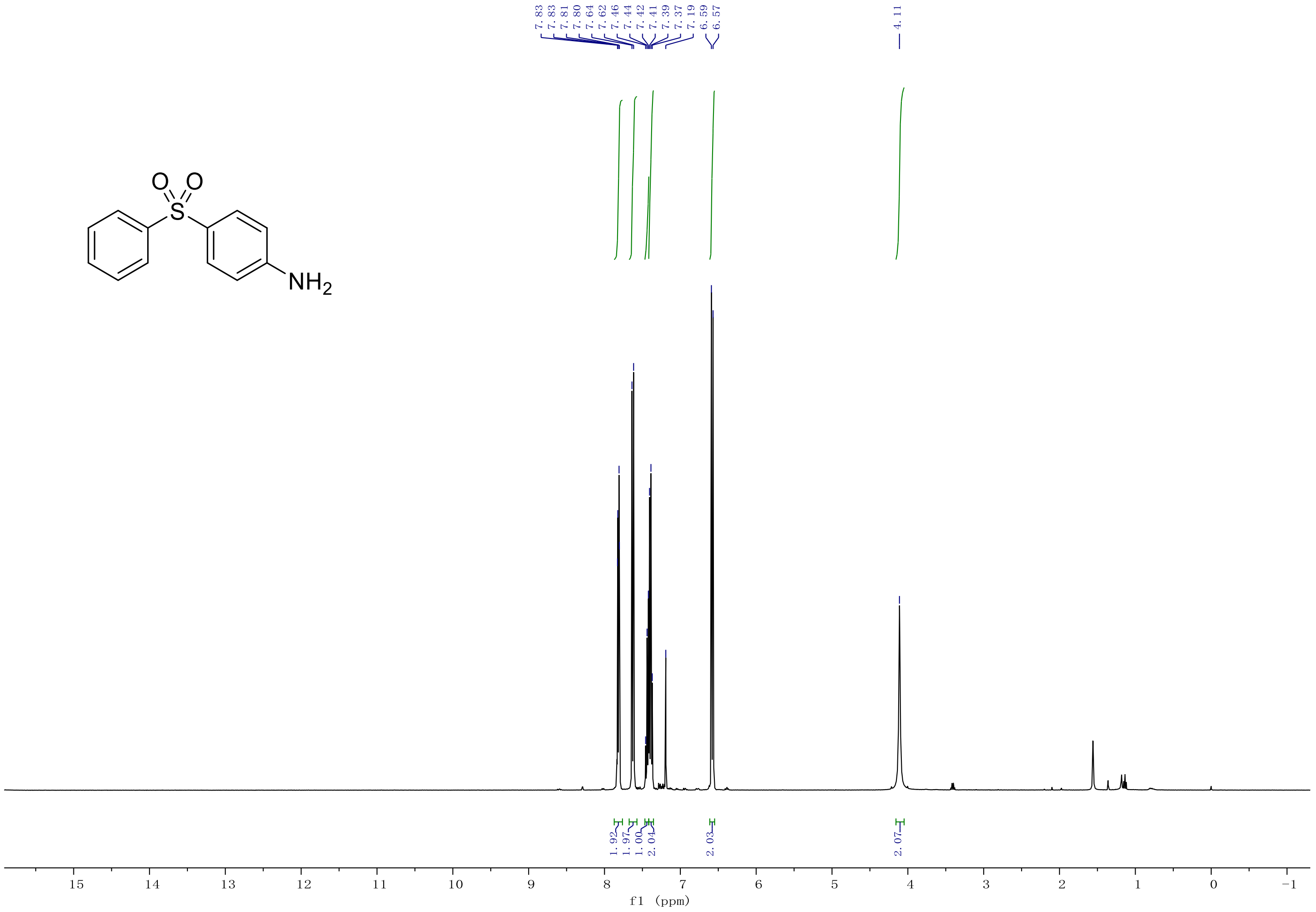

^{1}H NMR spectrum of **4-aminophenyl phenyl sulfone 3i**

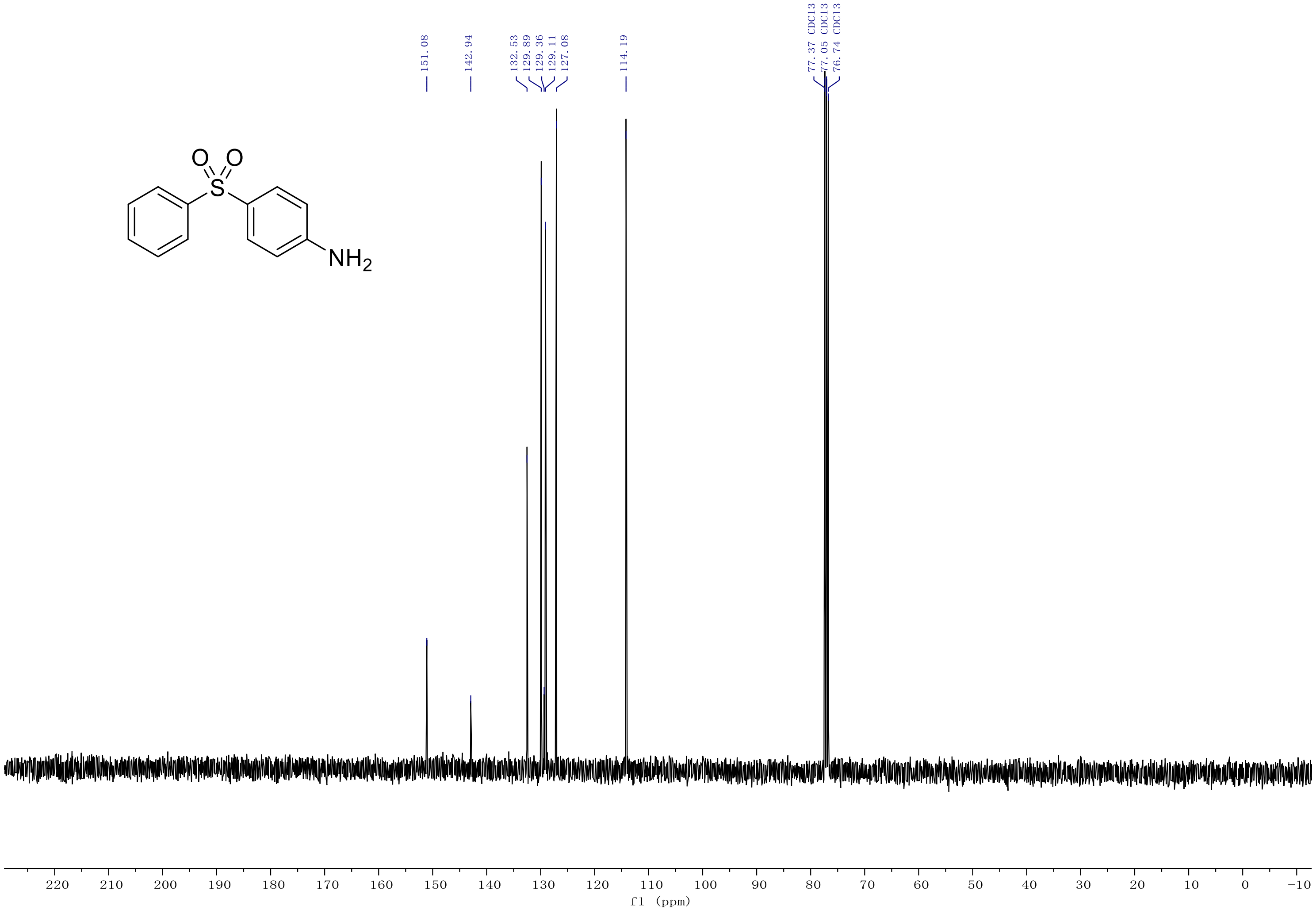

^{13}C NMR spectrum of **4-aminophenyl phenyl sulfone 3i**

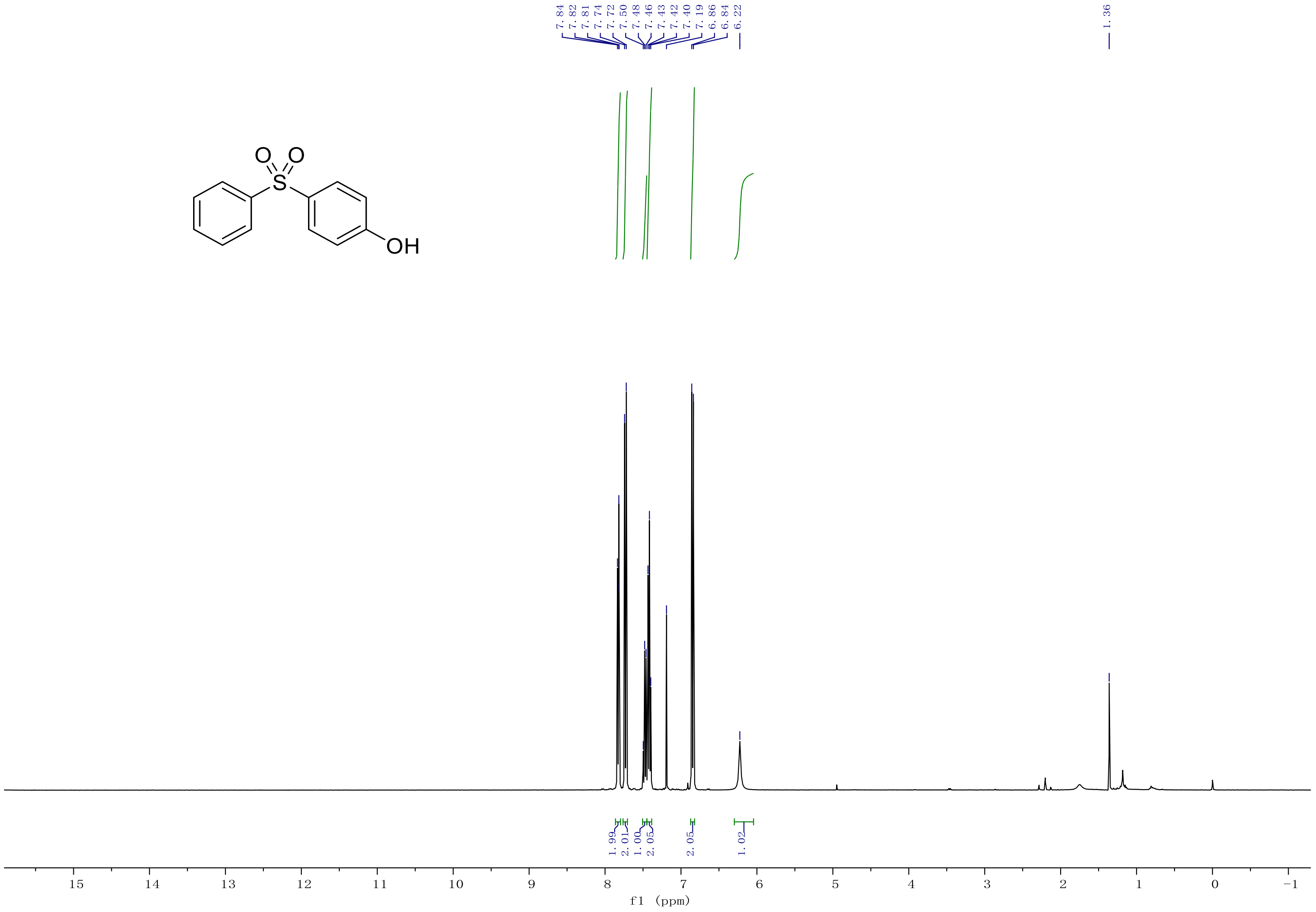

^{1}H NMR spectrum of **4-hydroxyphenyl p-tolyl sulfone 3j**

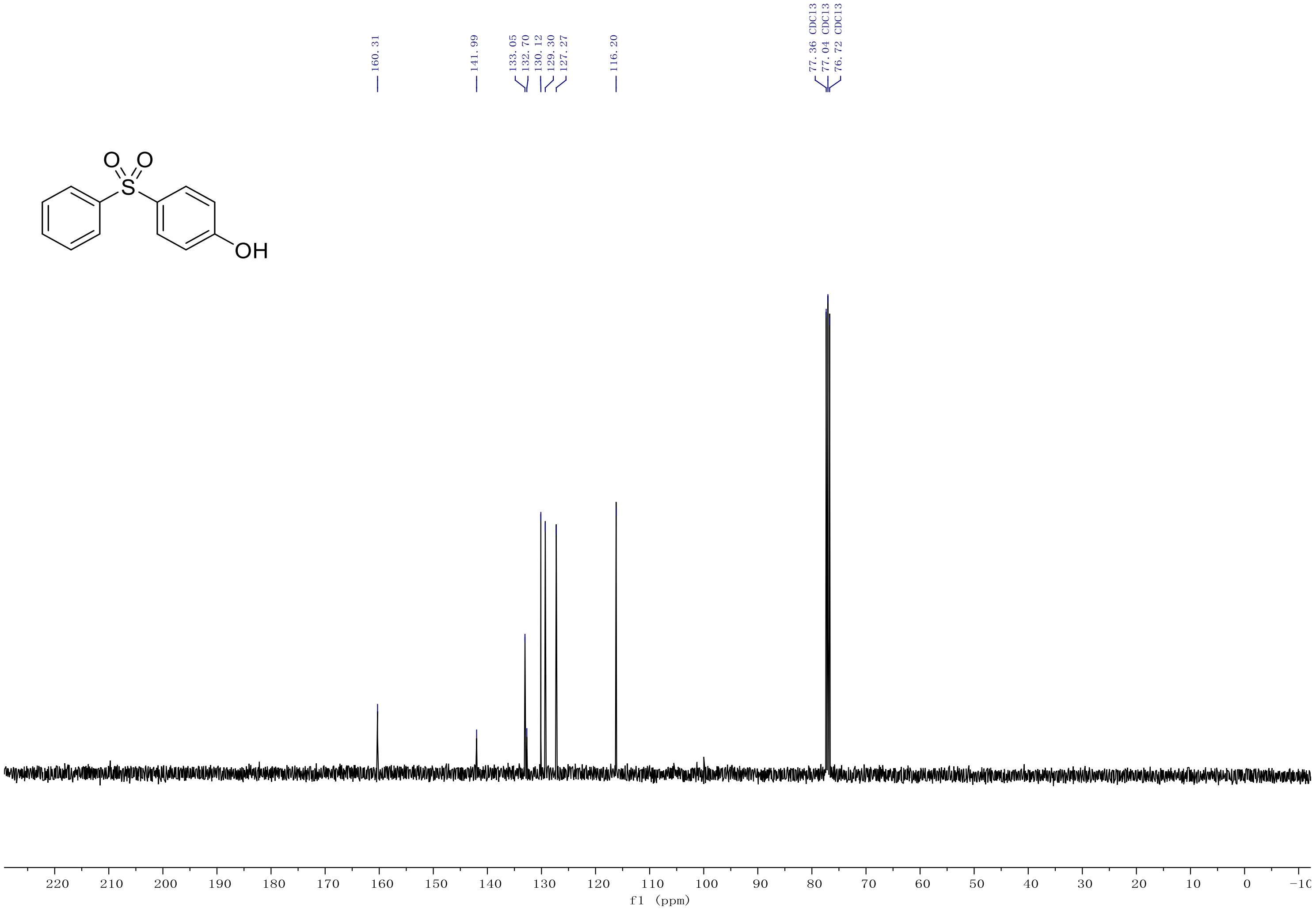

^{13}C NMR spectrum of **4-hydroxyphenyl p-tolyl sulfone 3j**

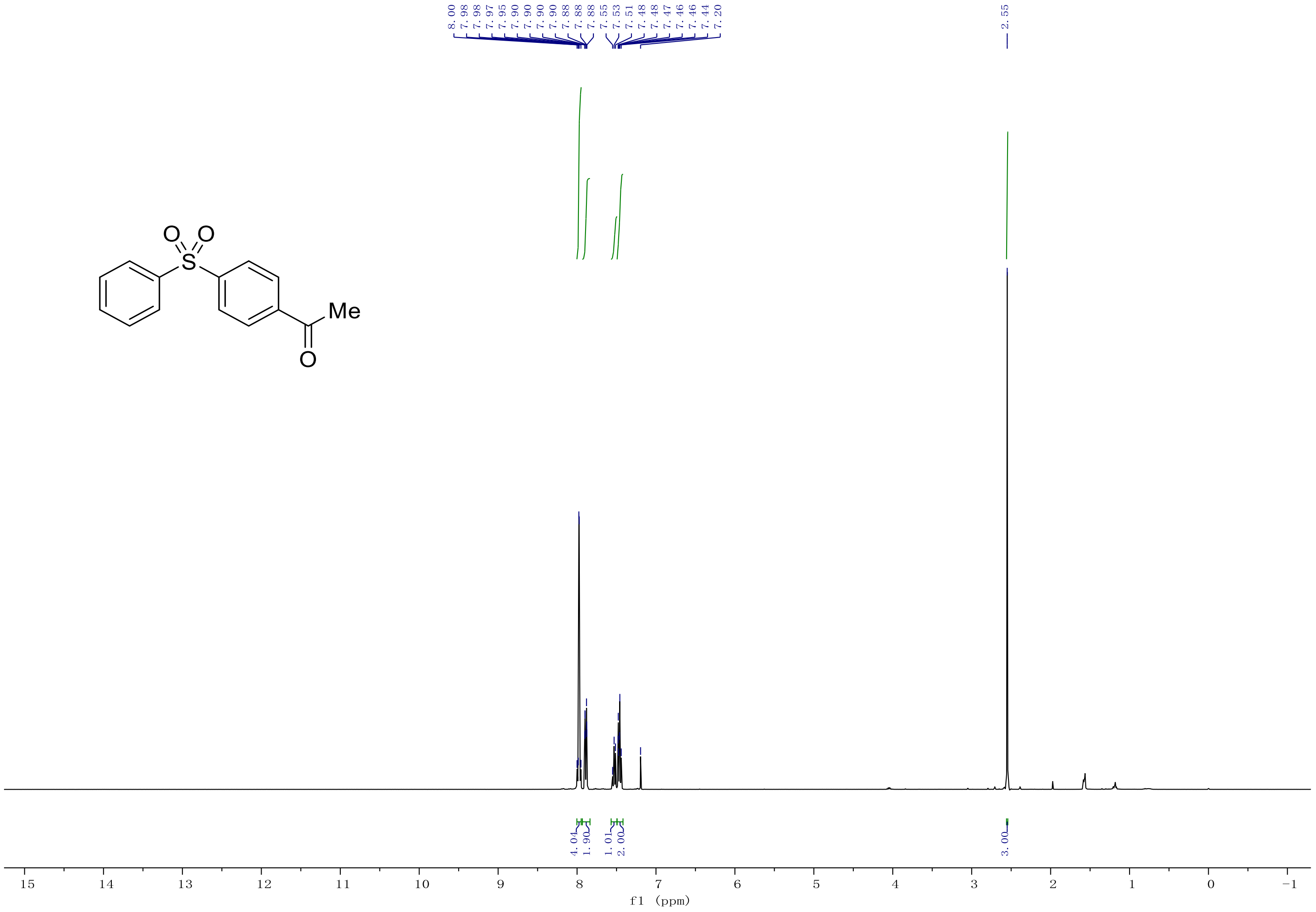

^{1}H NMR spectrum of **4'-(phenylsulfonyl)acetophenone 3k**

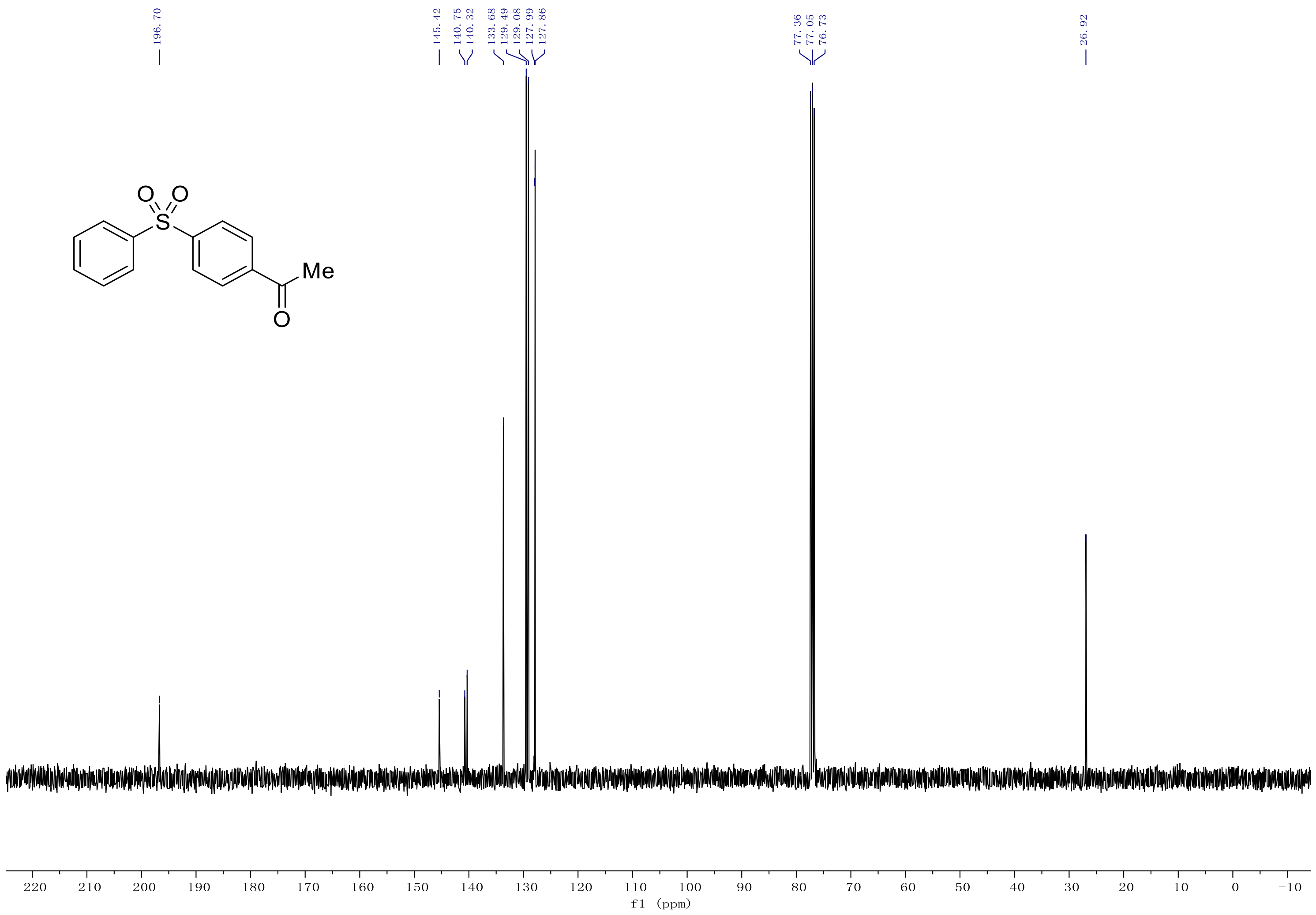

^{13}C NMR spectrum of **4'-(phenylsulfonyl)acetophenone 3k**

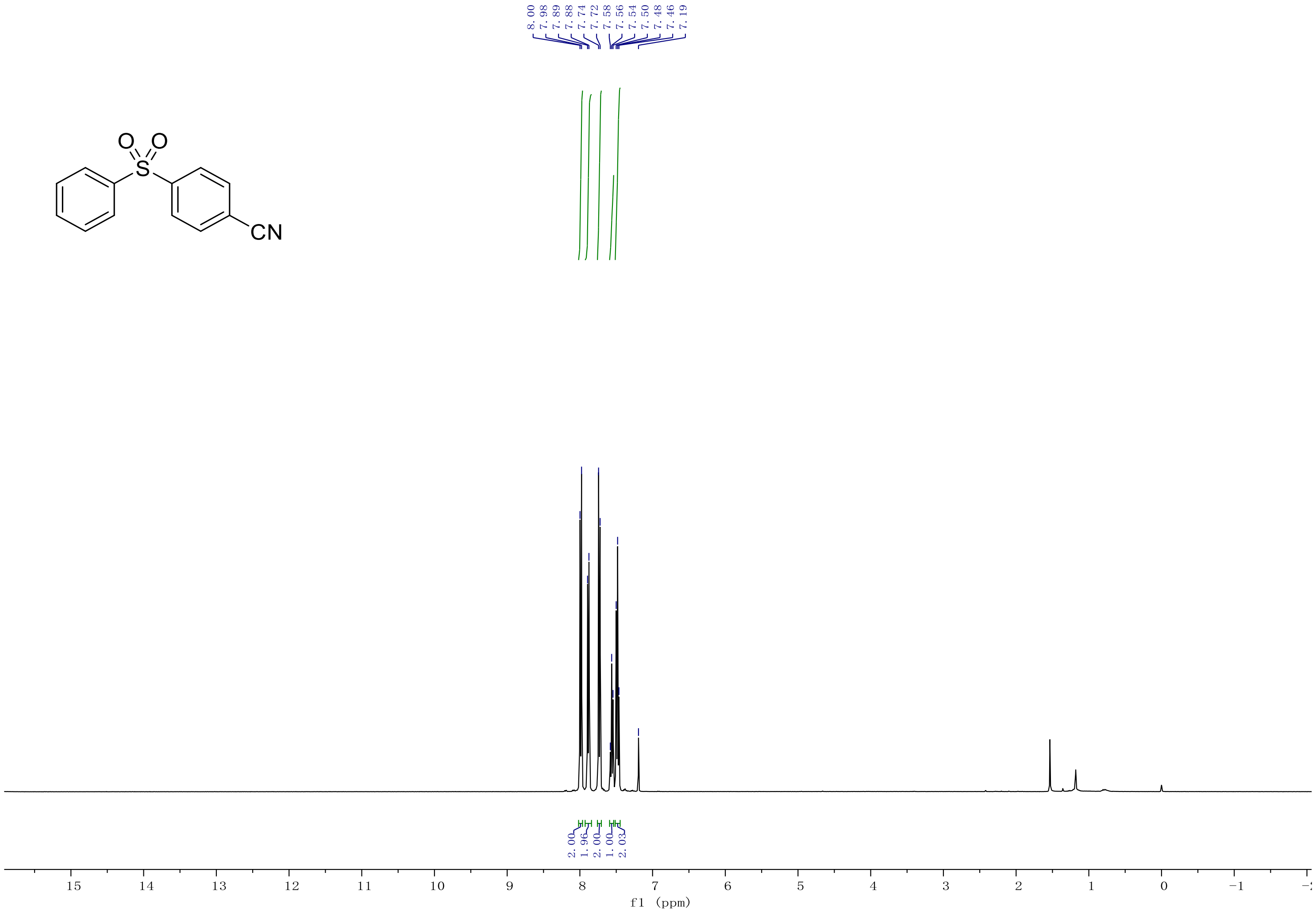

^{1}H NMR spectrum of **4-(phenylsulfonyl)benzonitrile 3l**

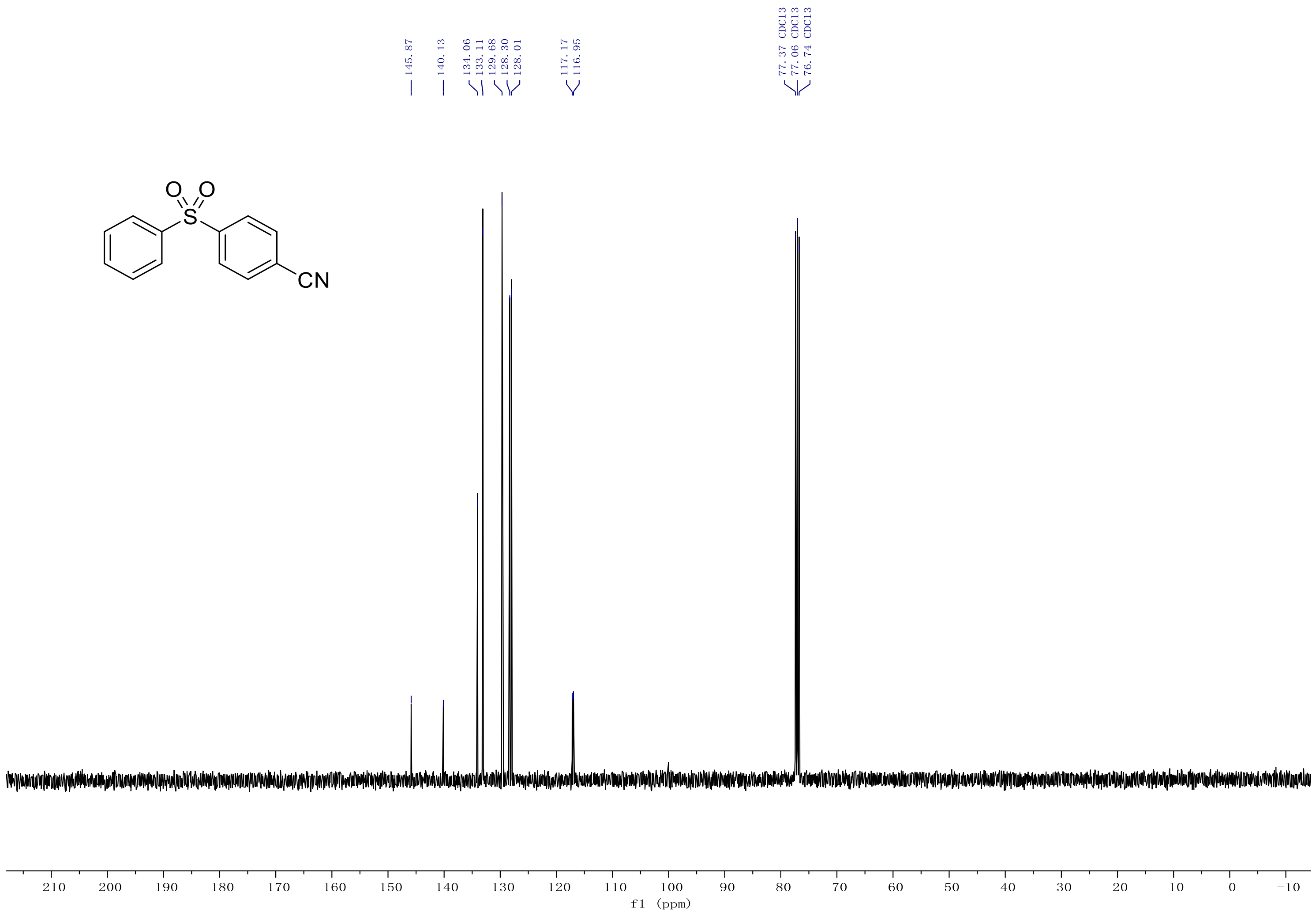

^{13}C NMR spectrum of **4-(phenylsulfonyl)benzonitrile 3l**

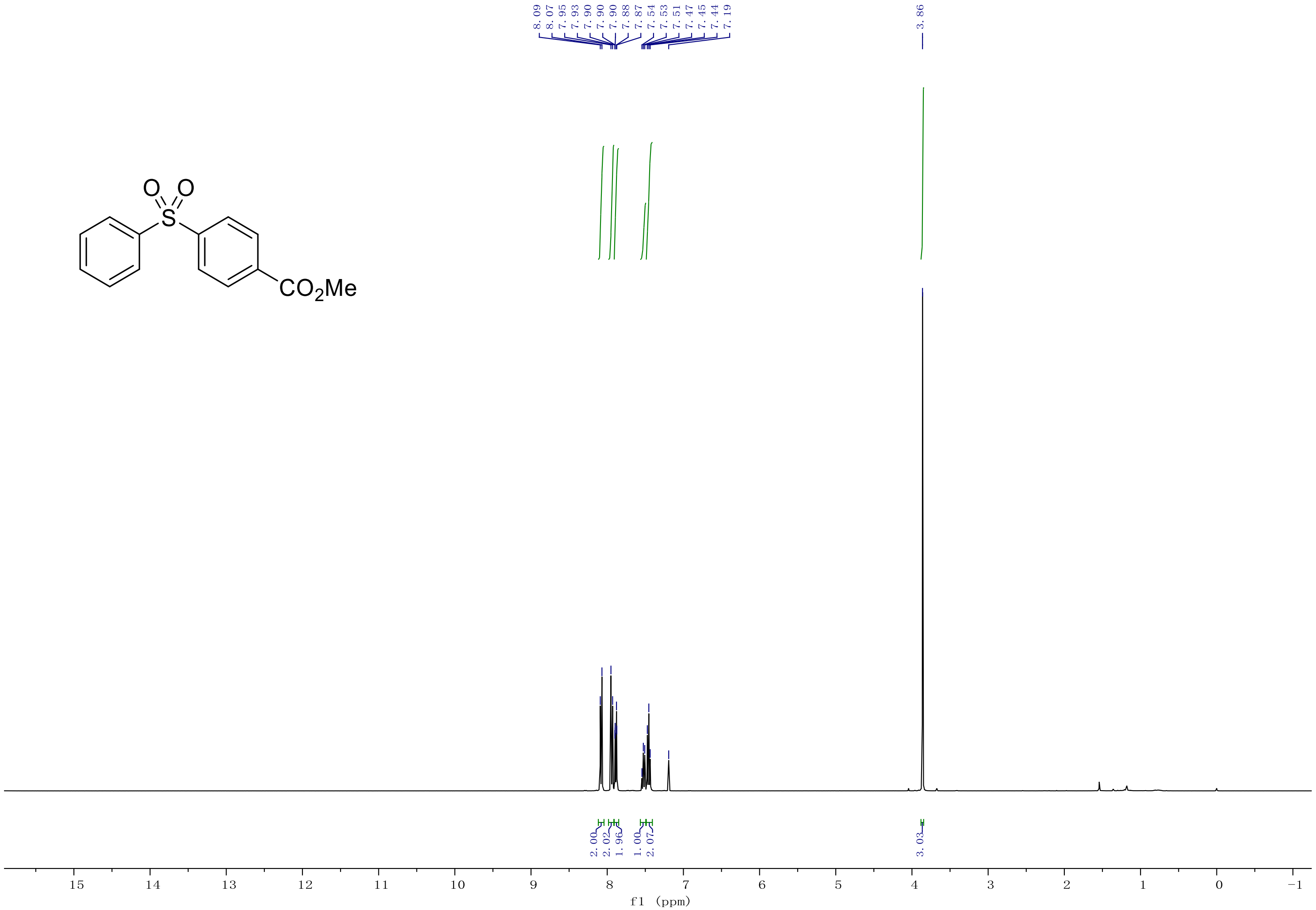

^{1}H NMR spectrum of **methyl 4-(phenylsulfonyl)benzoate 3m**

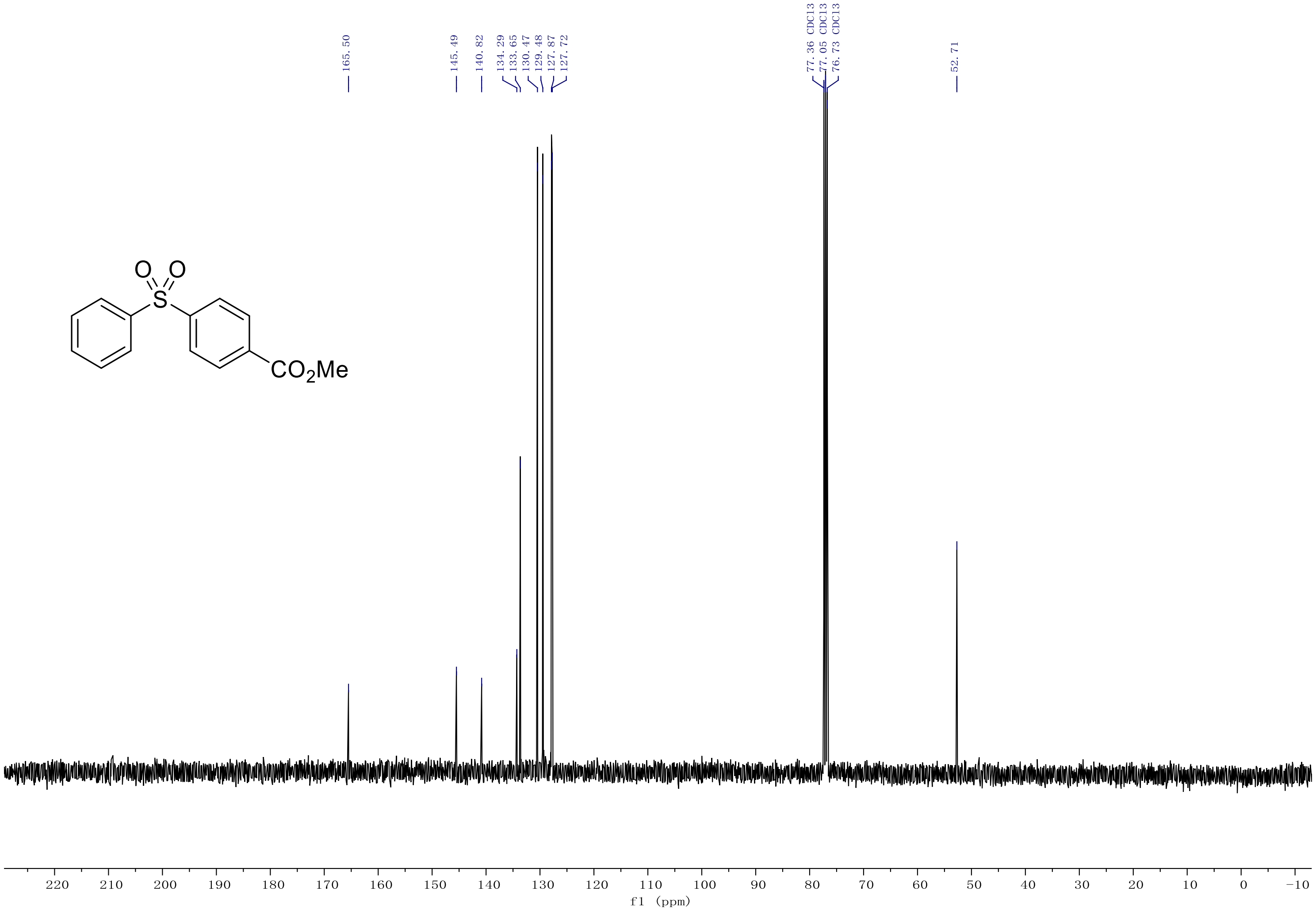

^{13}C NMR spectrum of **methyl 4-(phenylsulfonyl)benzoate 3m**

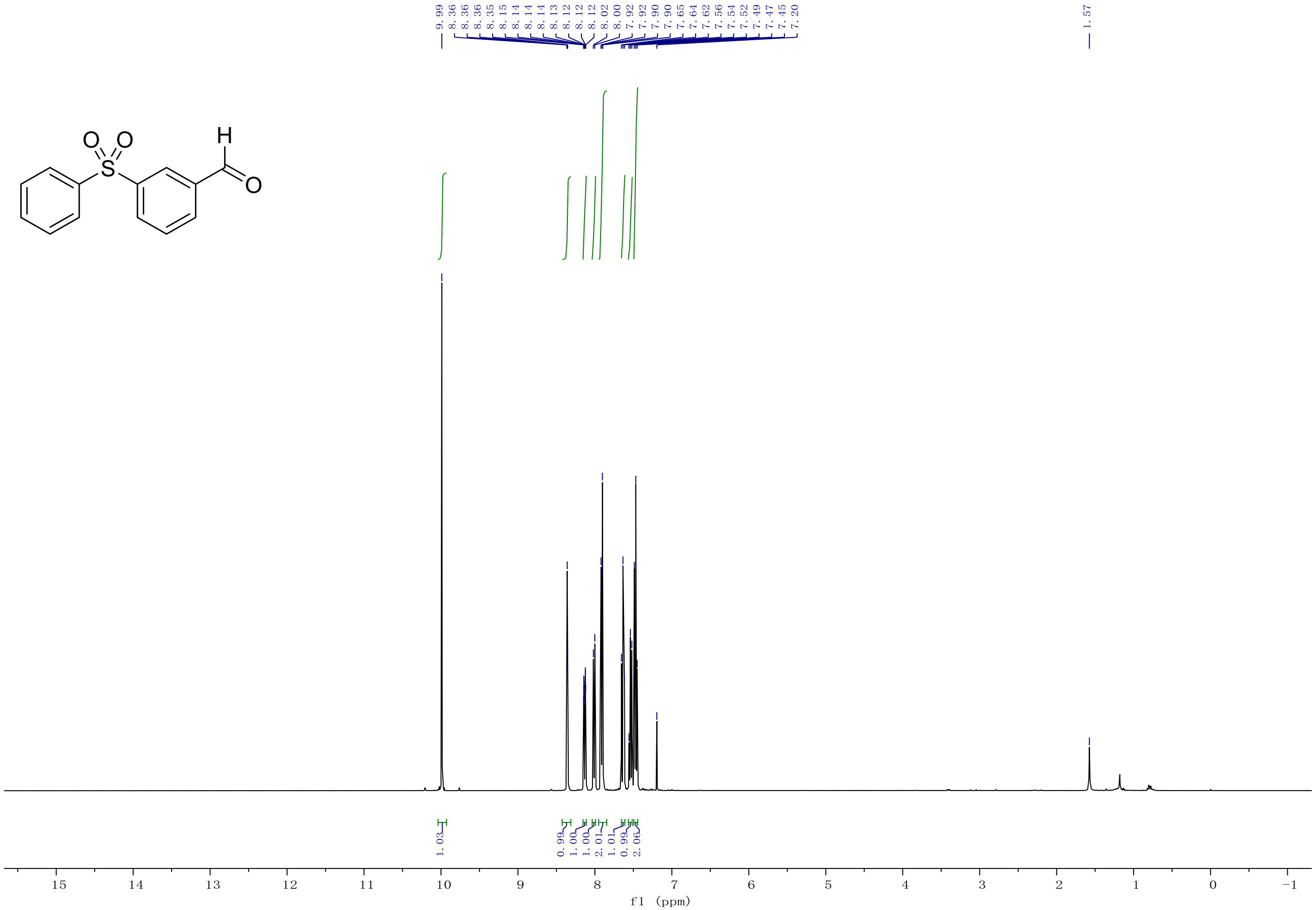

^{1}H NMR spectrum of **3-(phenylsulfonyl)benzaldehyde 3n**

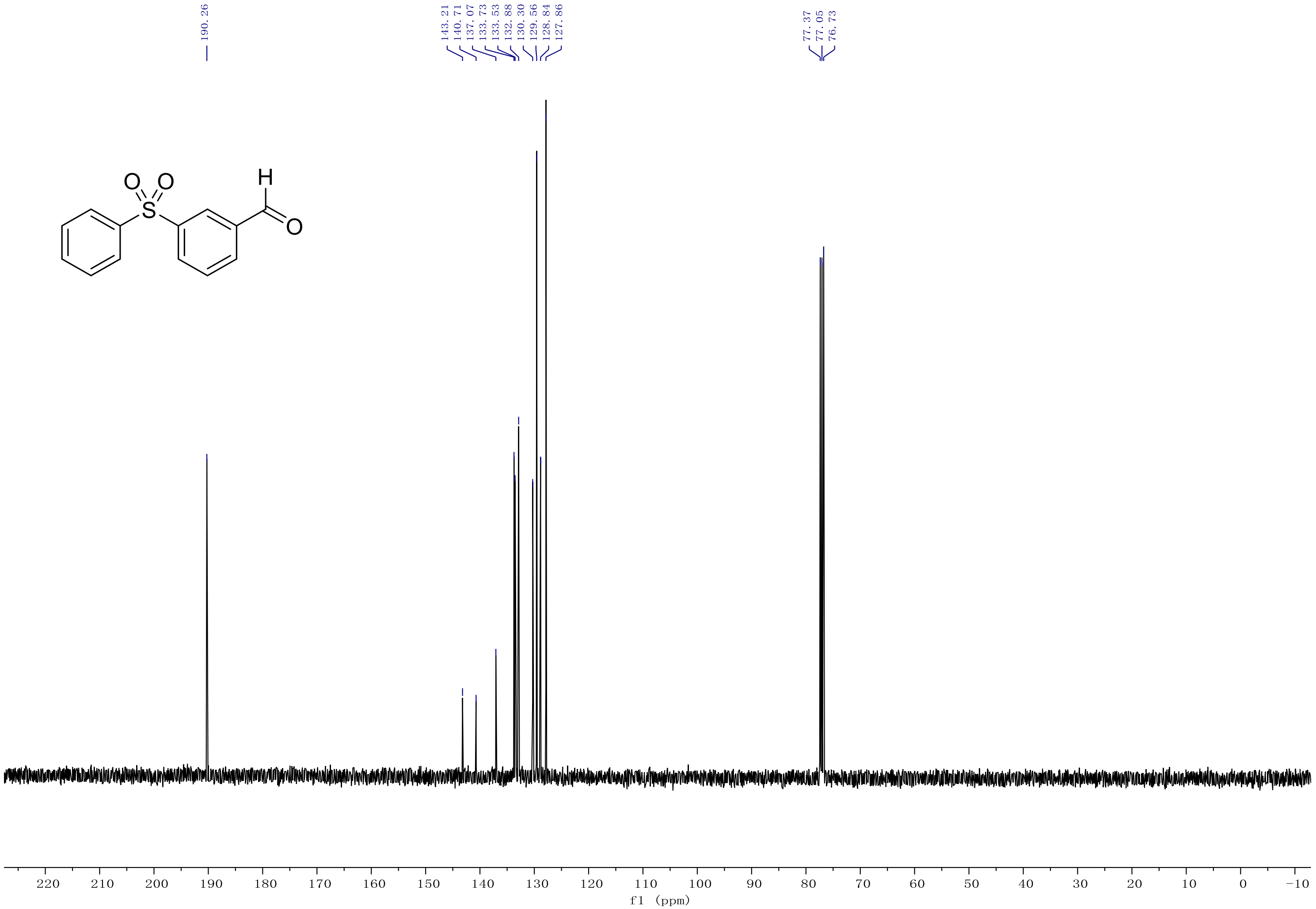

^{13}C NMR spectrum of **3-(phenylsulfonyl)benzaldehyde 3n**

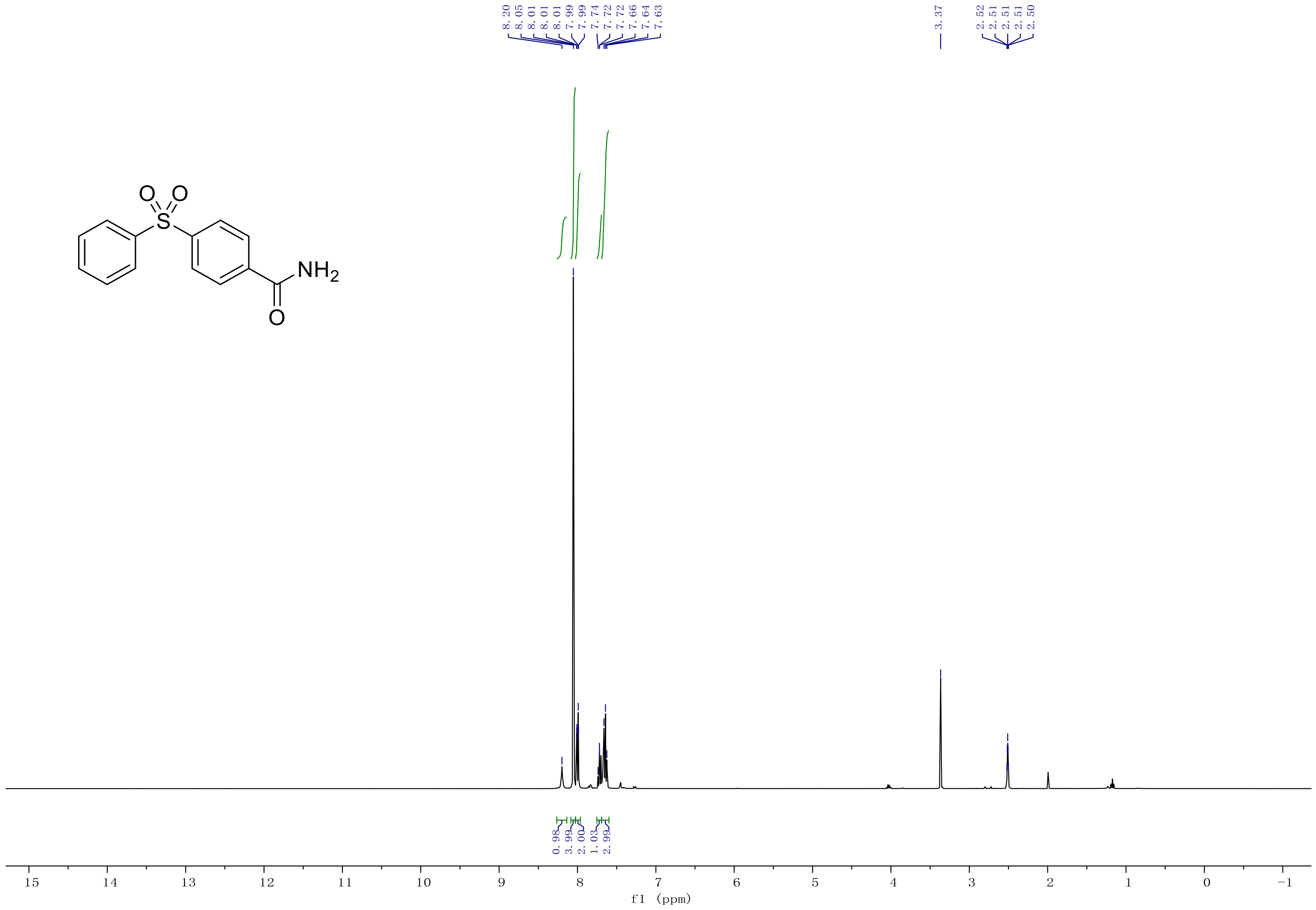

^{1}H NMR spectrum of **4-benzenesulfonyl-benzoic acid amide 3o**

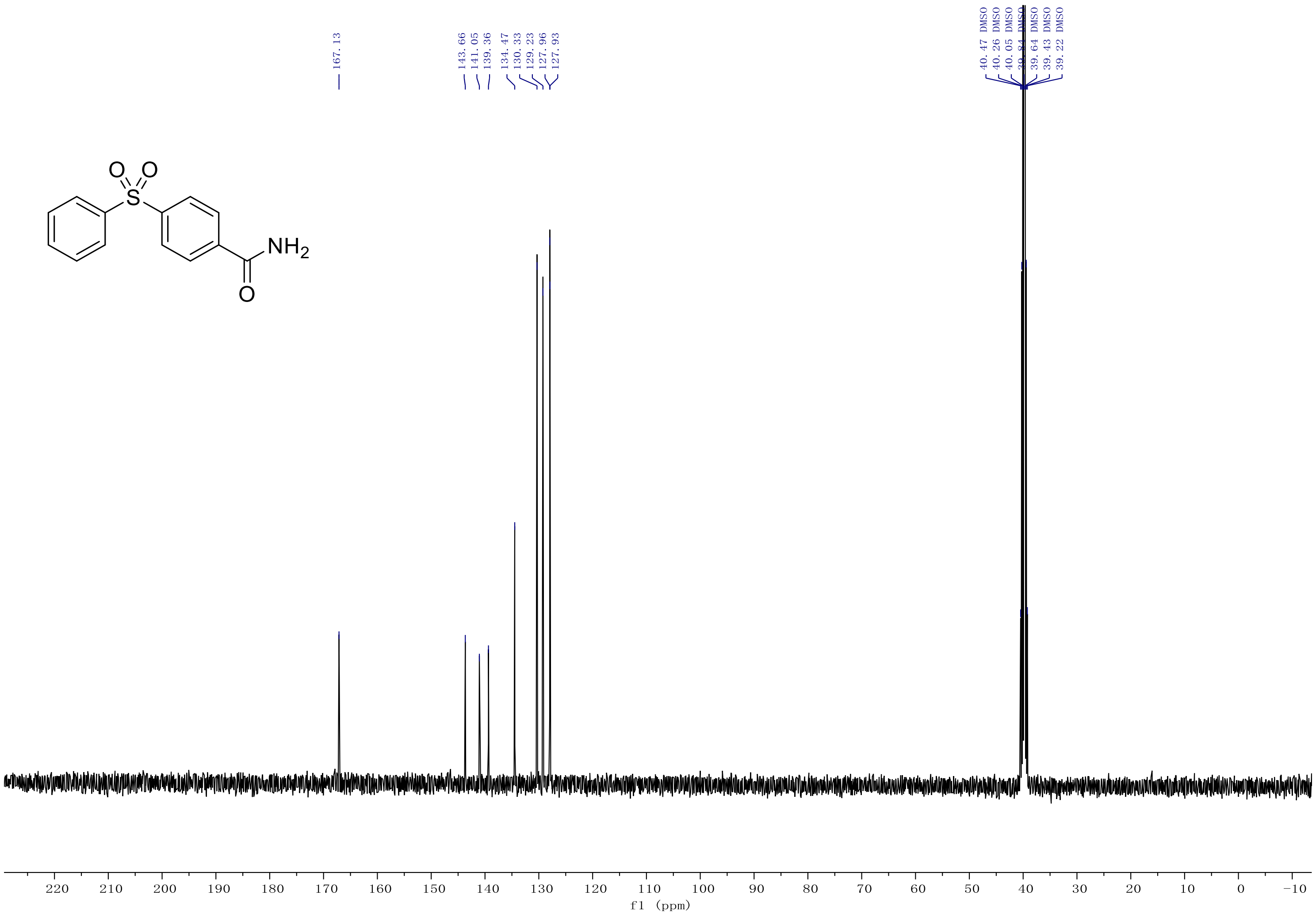

^{13}C NMR spectrum of **4-benzenesulfonyl-benzoic acid amide 3o**

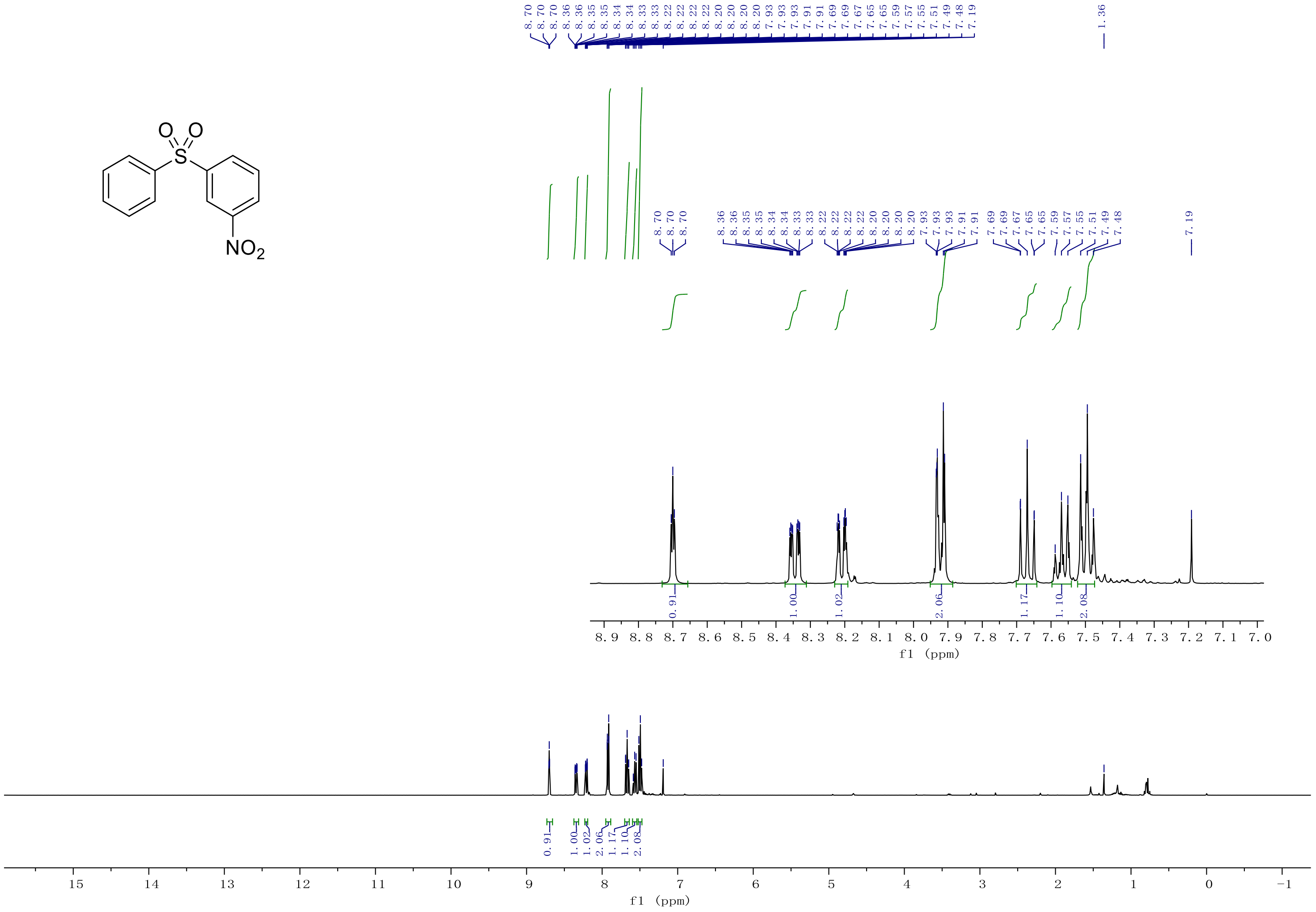

^{1}H NMR spectrum of **3-nitrodiphenyl sulfone 3p**

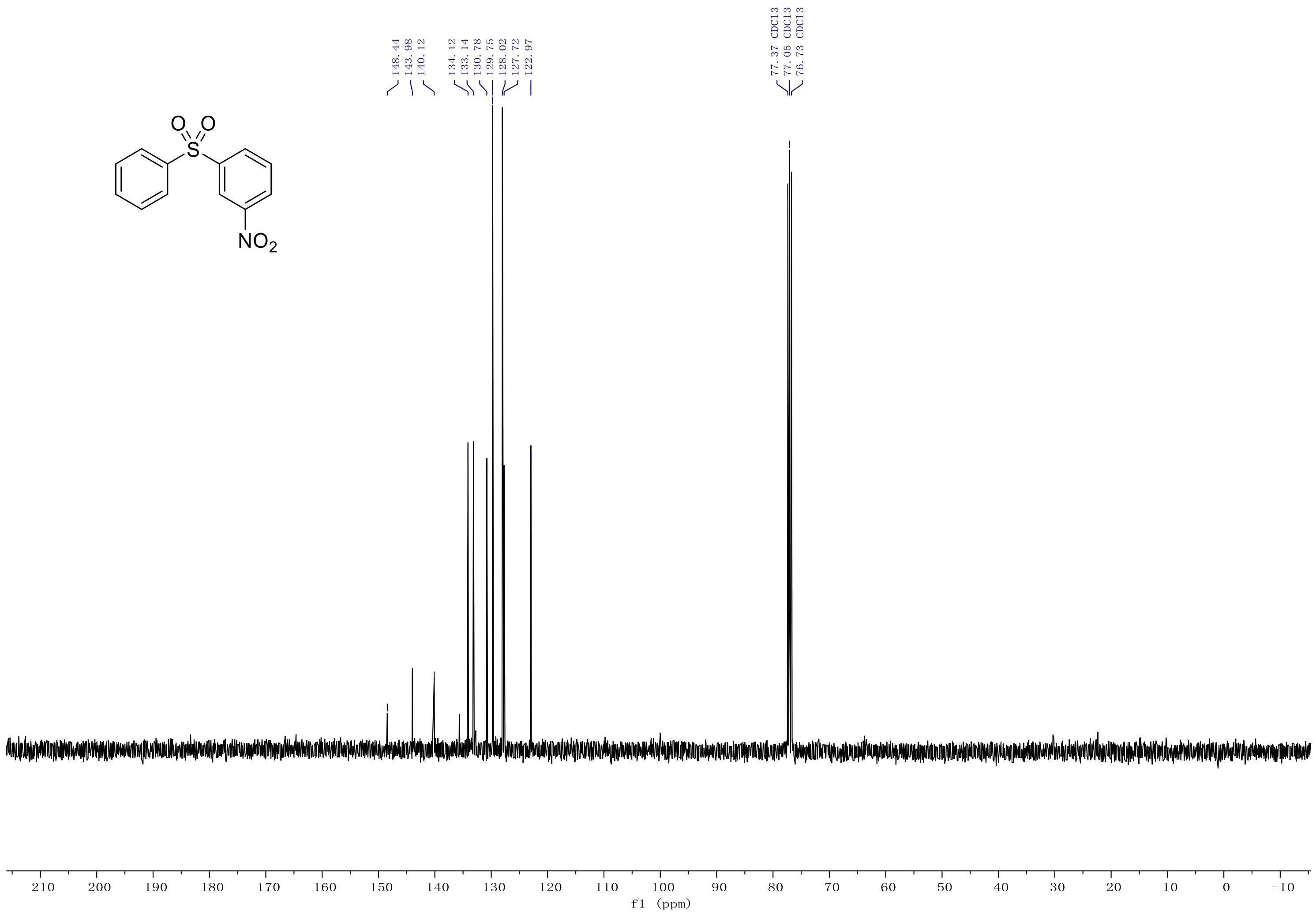

^{13}C NMR spectrum of **3-nitrodiphenyl sulfone 3p**

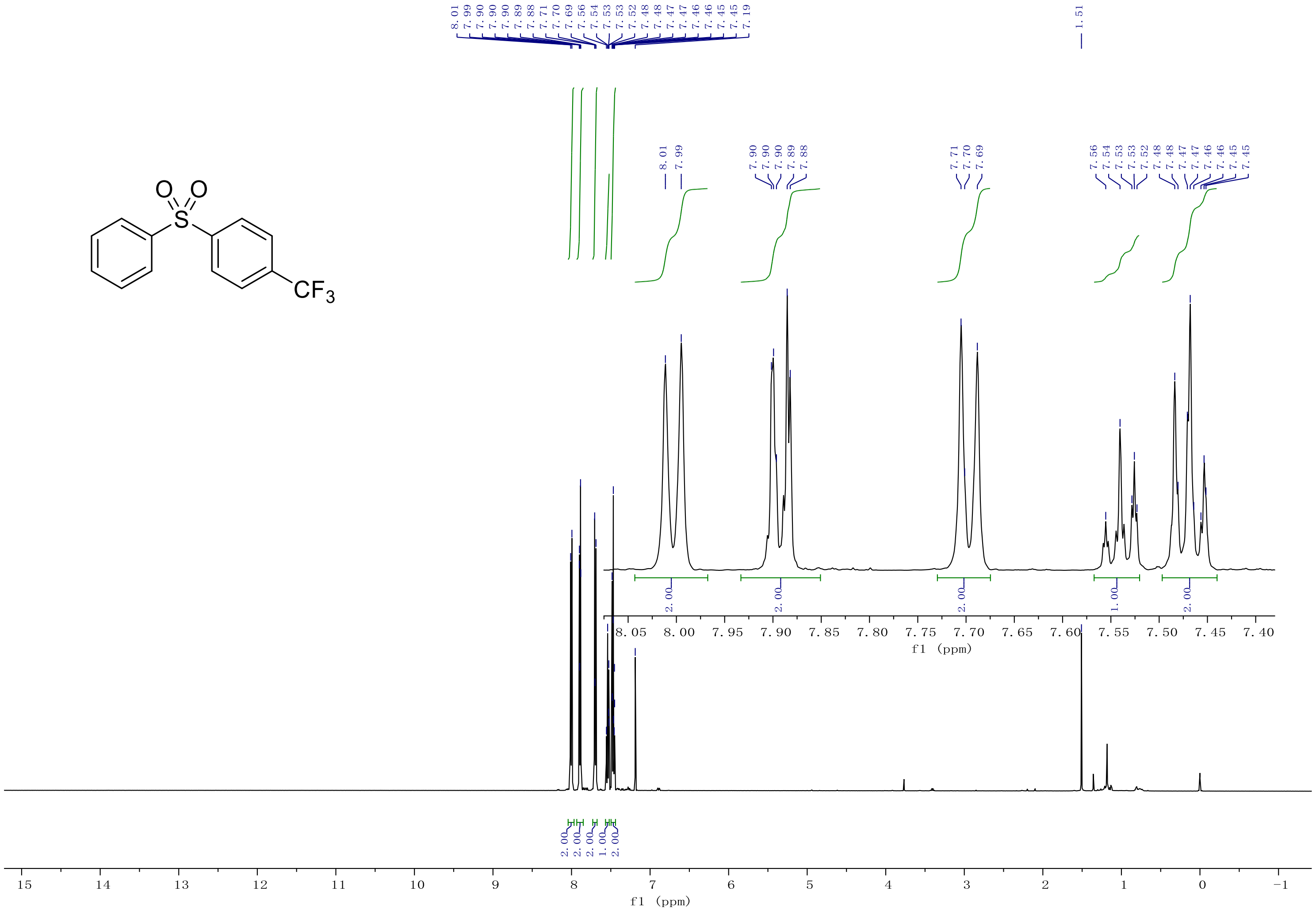

^{1}H NMR spectrum of **1-(phenylsulfonyl)-4-(trifluoromethyl)benzene 3q**

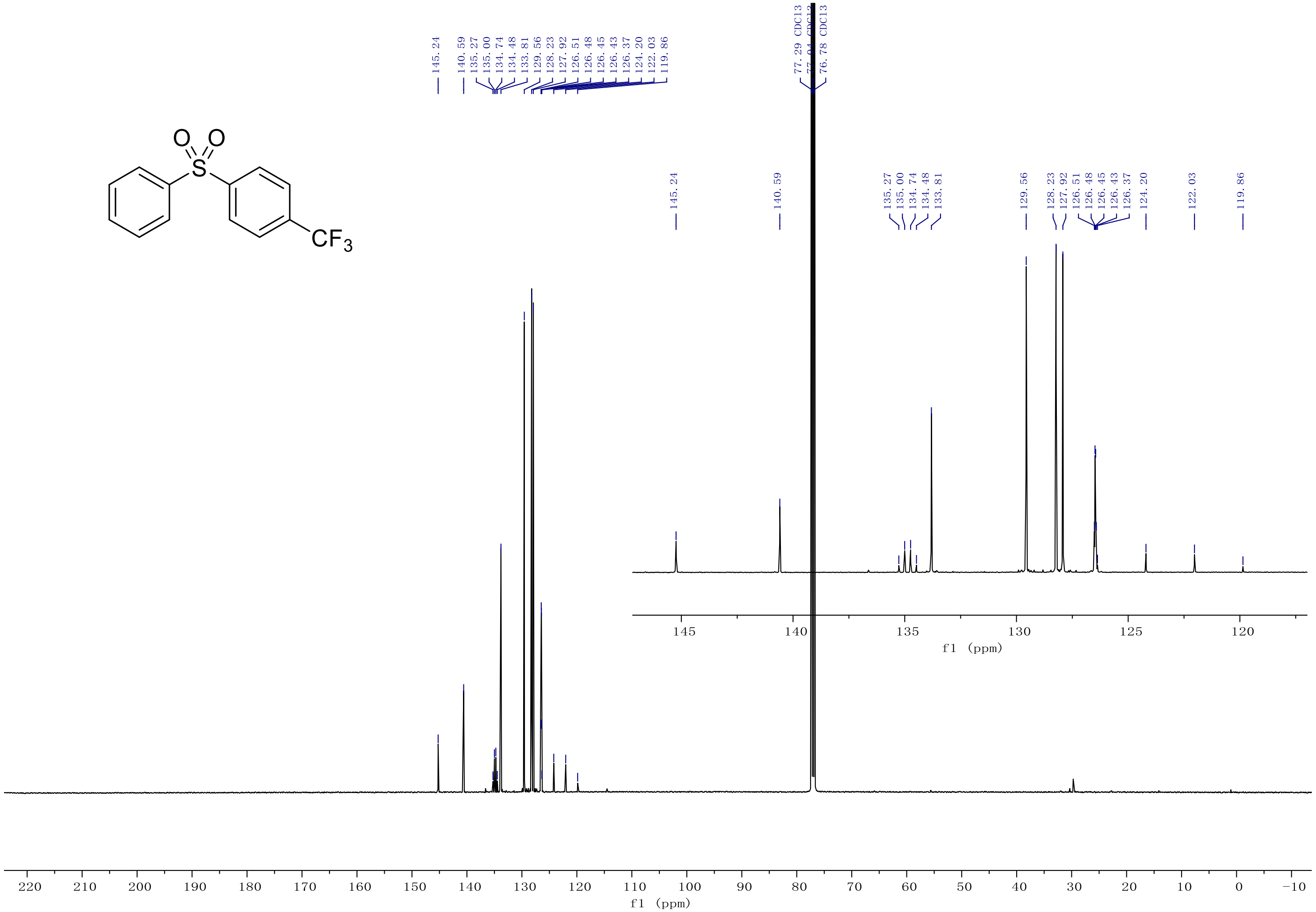

^{13}C NMR spectrum of **1-(phenylsulfonyl)-4-(trifluoromethyl)benzene 3q**

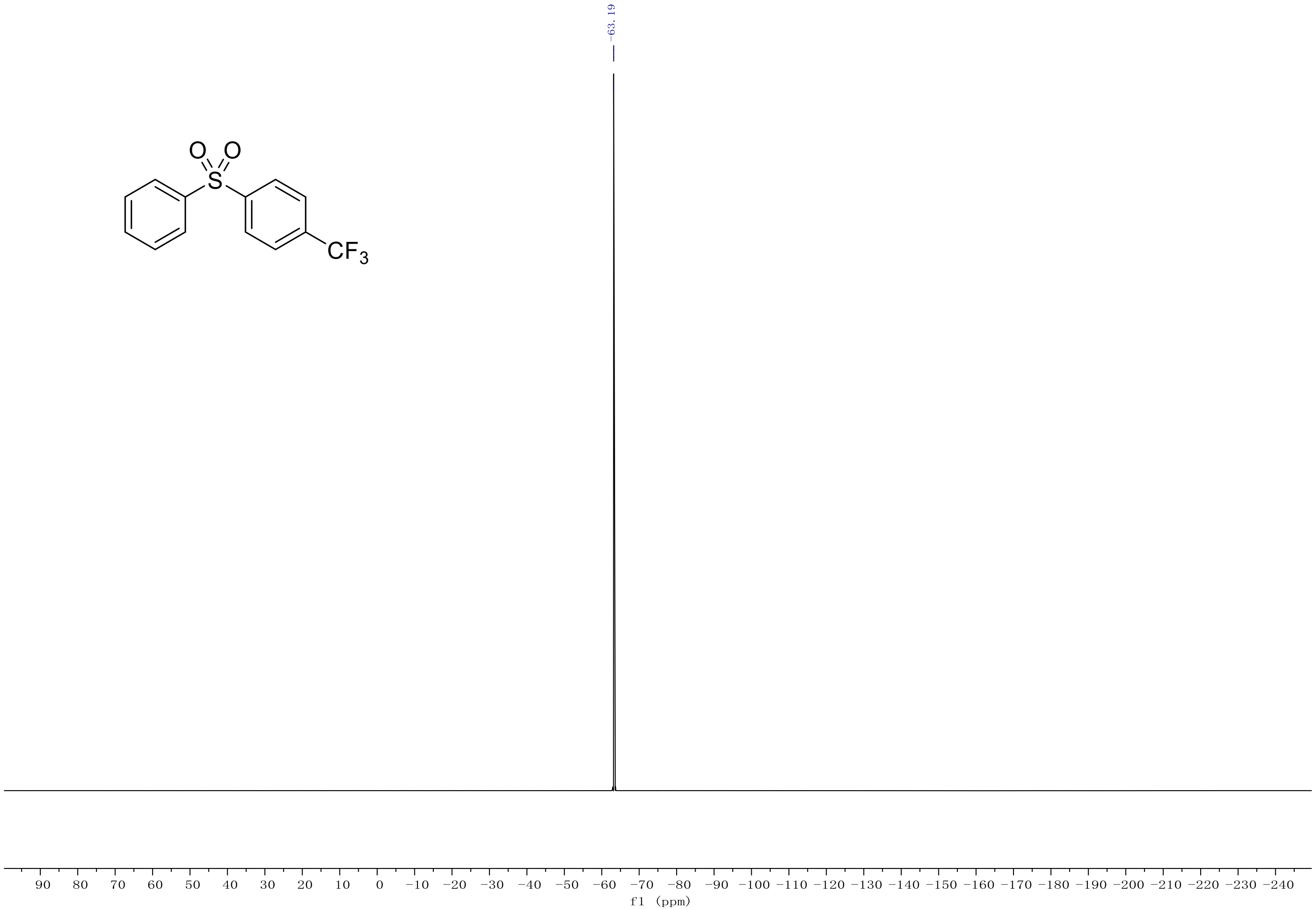

^{19}F NMR spectrum of **1-(phenylsulfonyl)-4-(trifluoromethyl)benzene 3q**

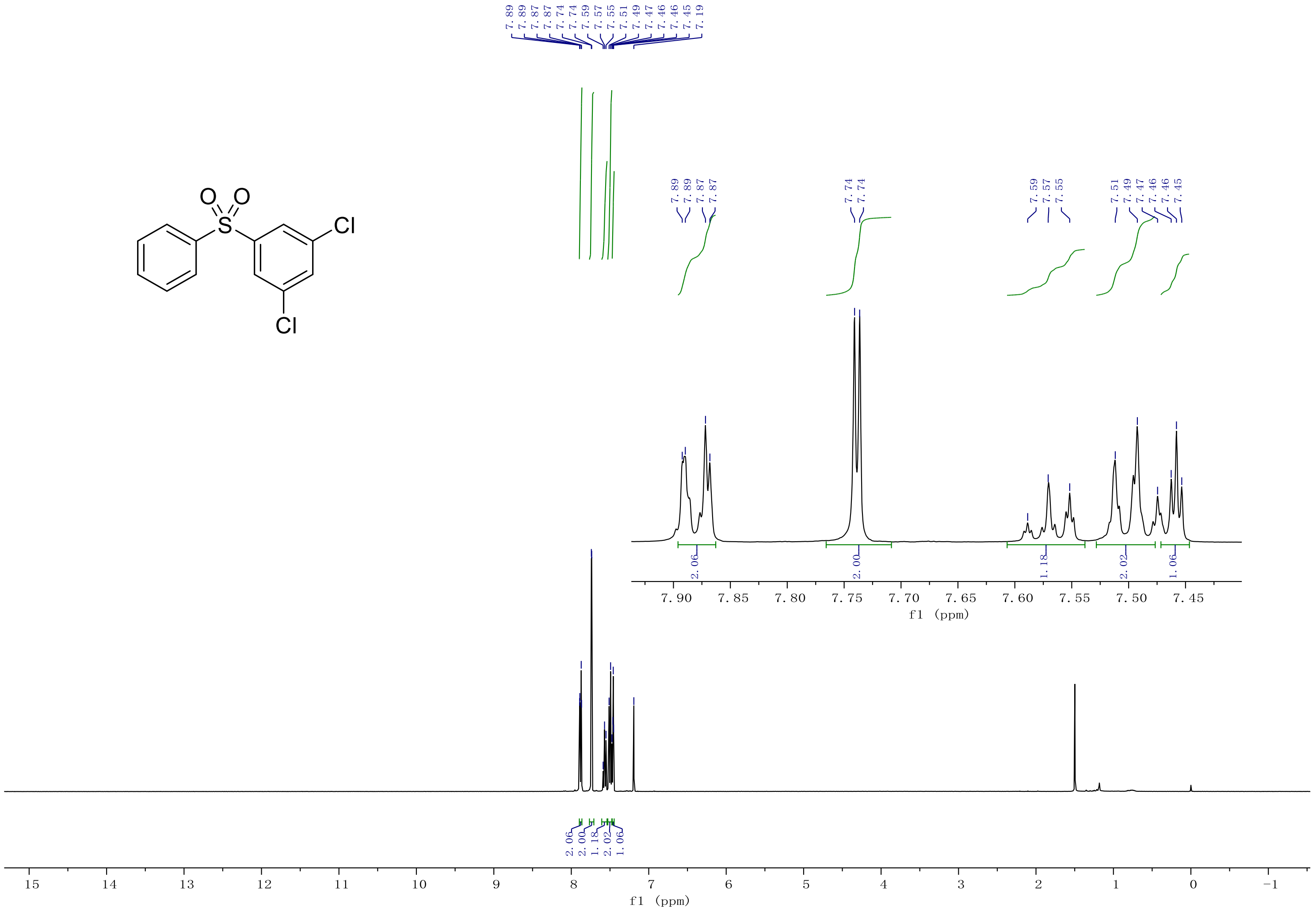

^{1}H NMR spectrum of **3,5-dichloro-1-(phenylsulfonyl)benzene 3r**

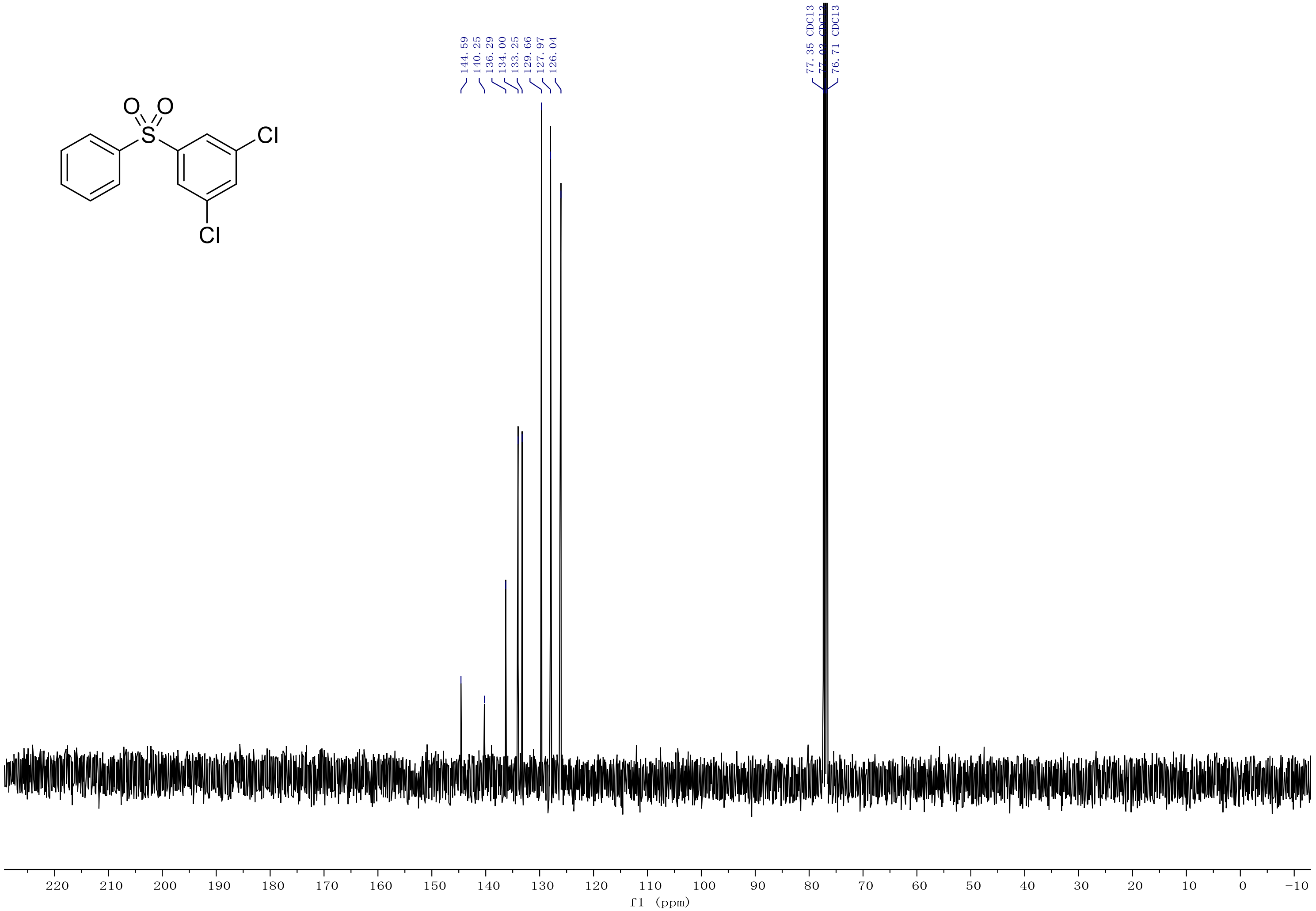

^{1}H NMR and ^{13}C NMR spectrum of **3,5-dichloro-1-(phenylsulfonyl)benzene 3r**

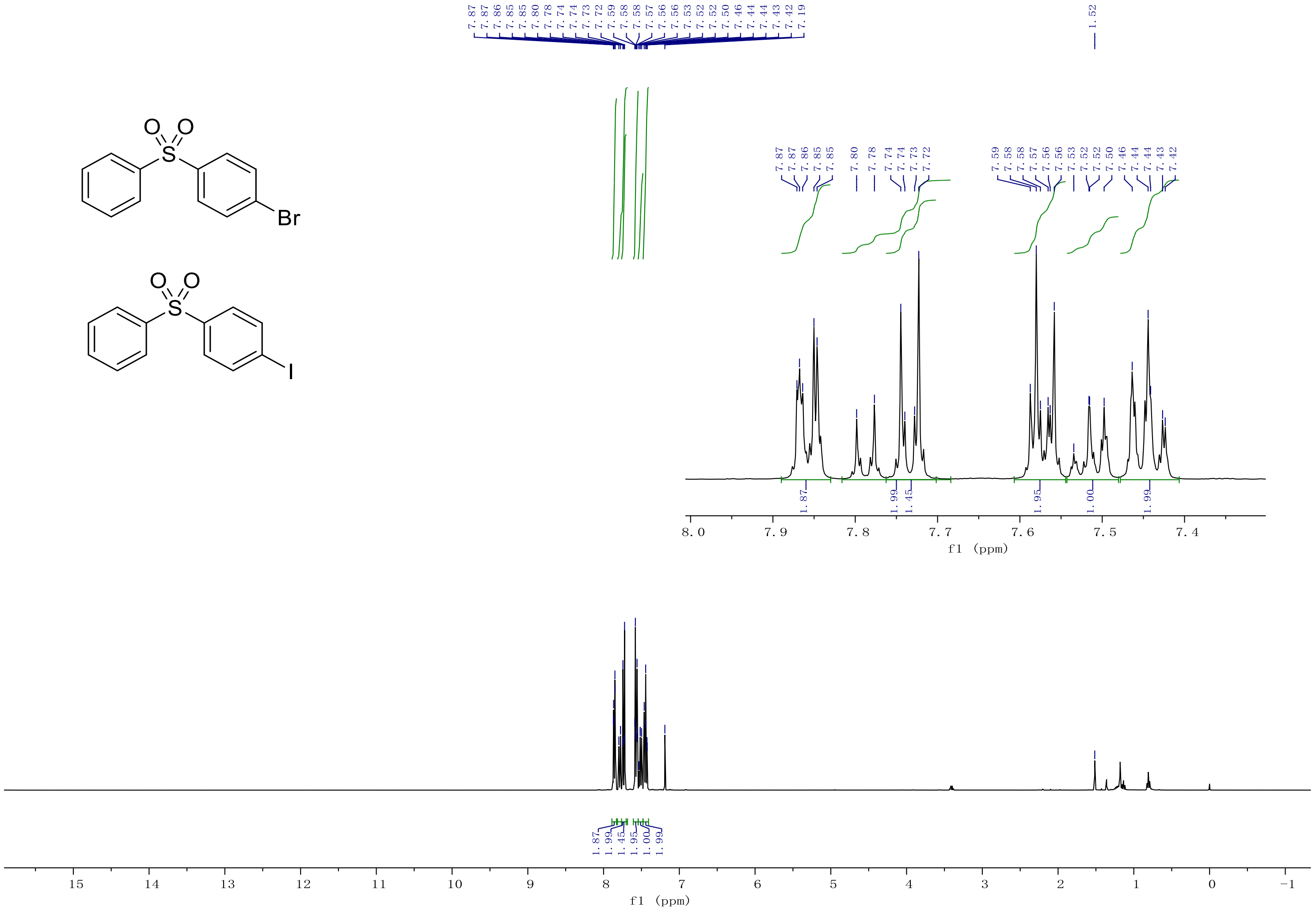

^{1}H NMR spectrum of **4-phenylsulfonylbromobenzene and 4-phenylsulfonyliodobenzene 3s/3s'**

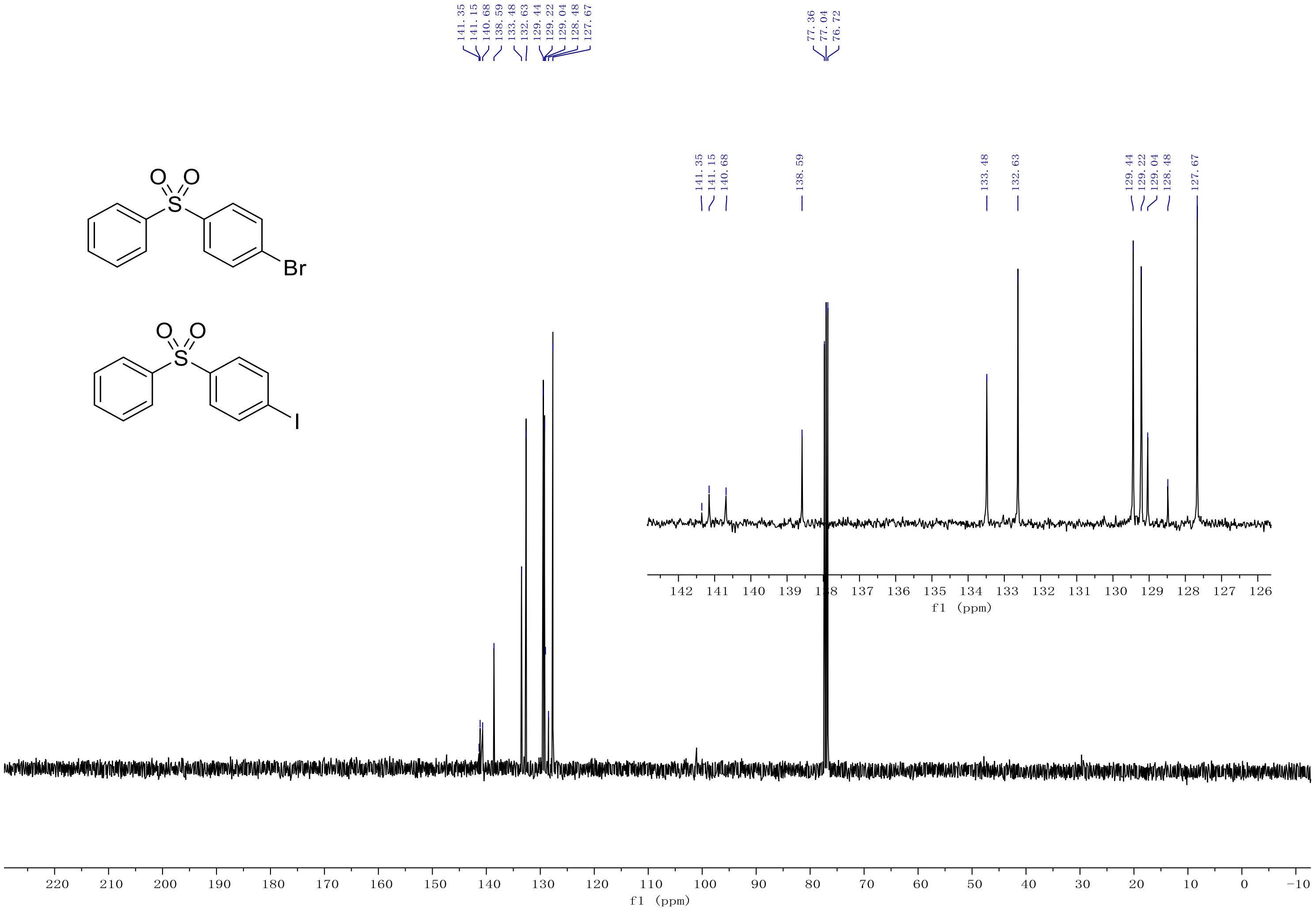

^{13}C NMR spectrum of **4-phenylsulfonylbromobenzene and 4-phenylsulfonyliodobenzene 3s/3s’**

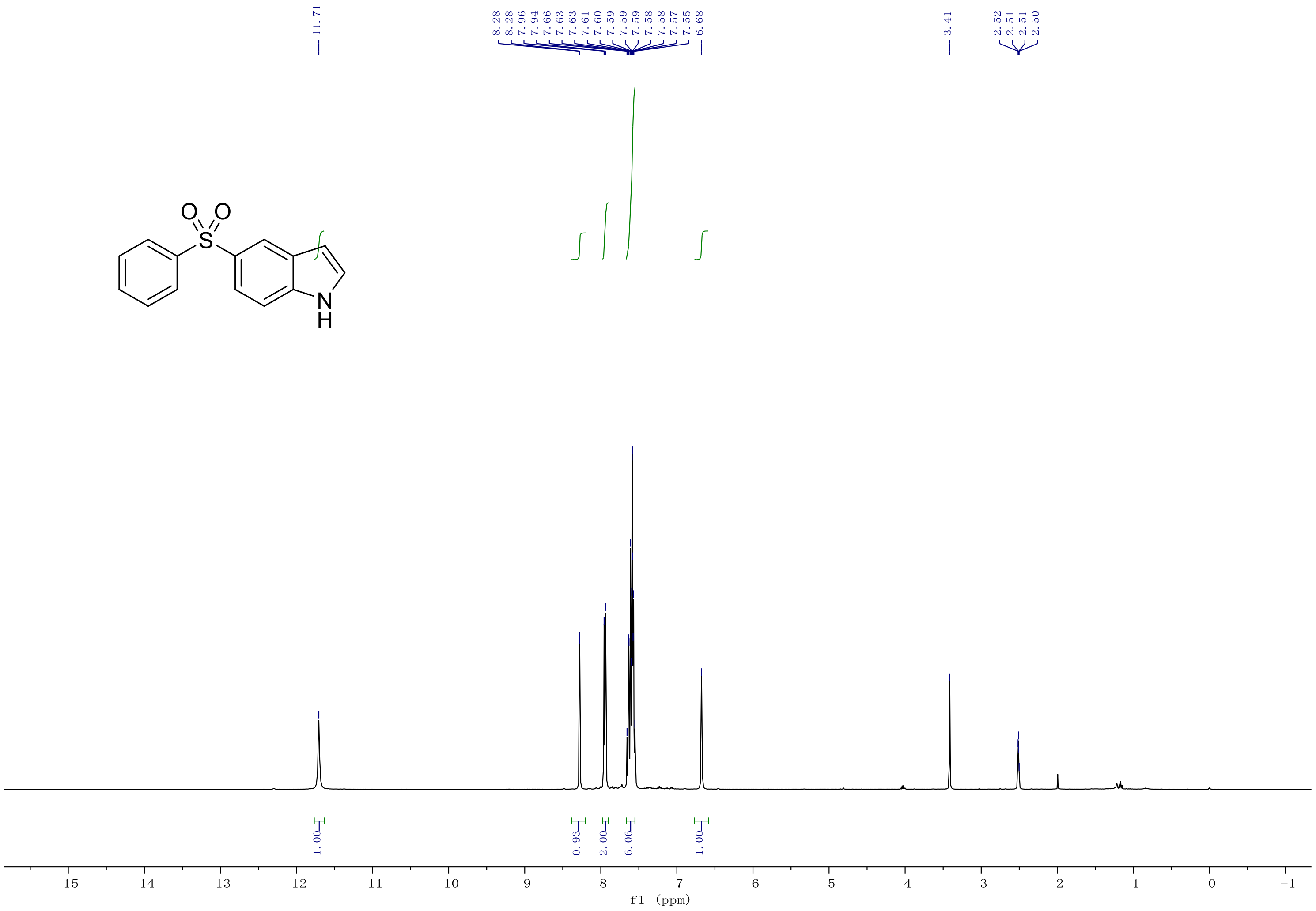

^{1}H NMR spectrum of **5-(phenylsulfonyl)indole 3t**

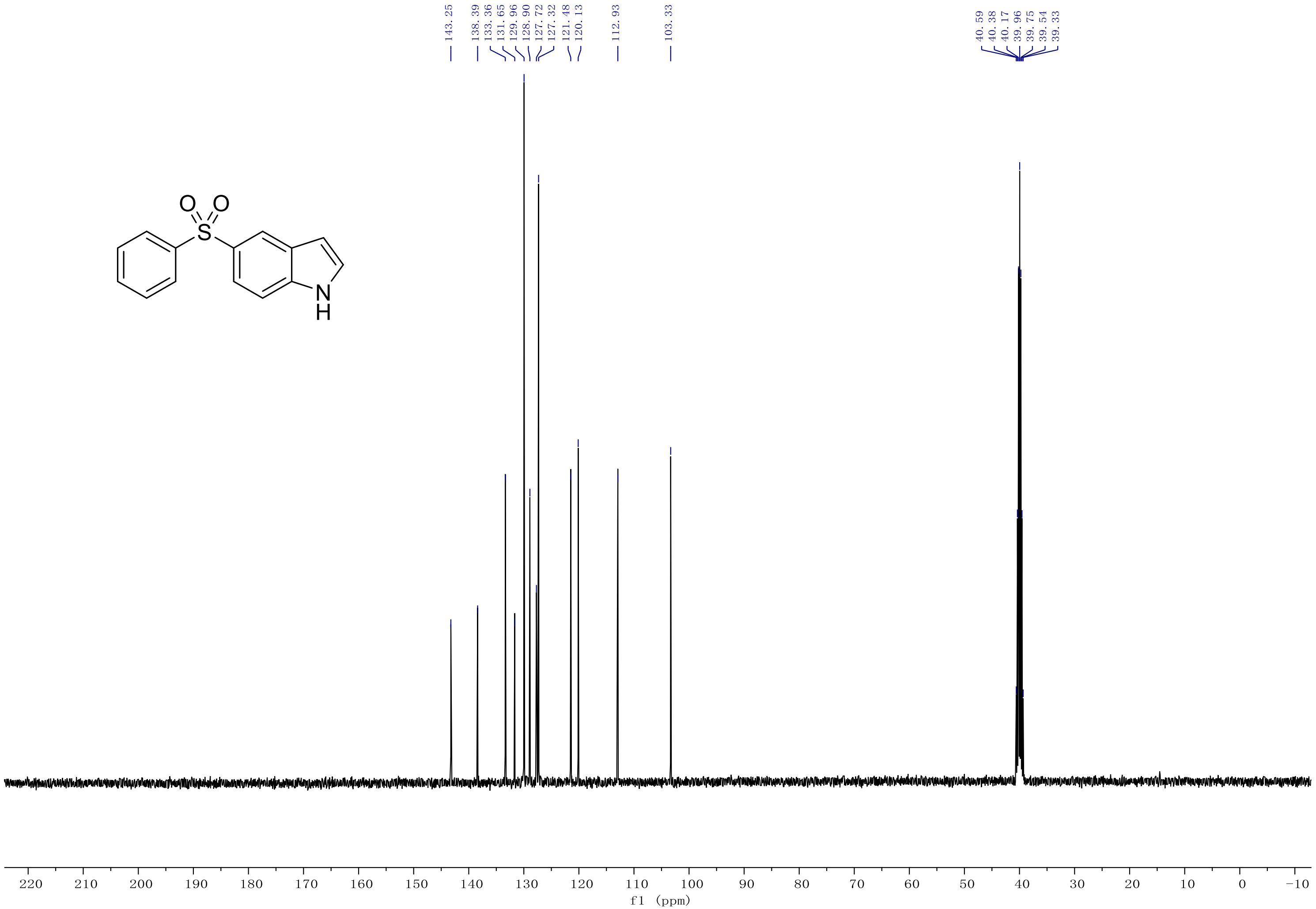

^{13}C NMR spectrum of **5-(phenylsulfonyl)indole 3t**

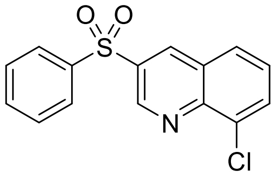

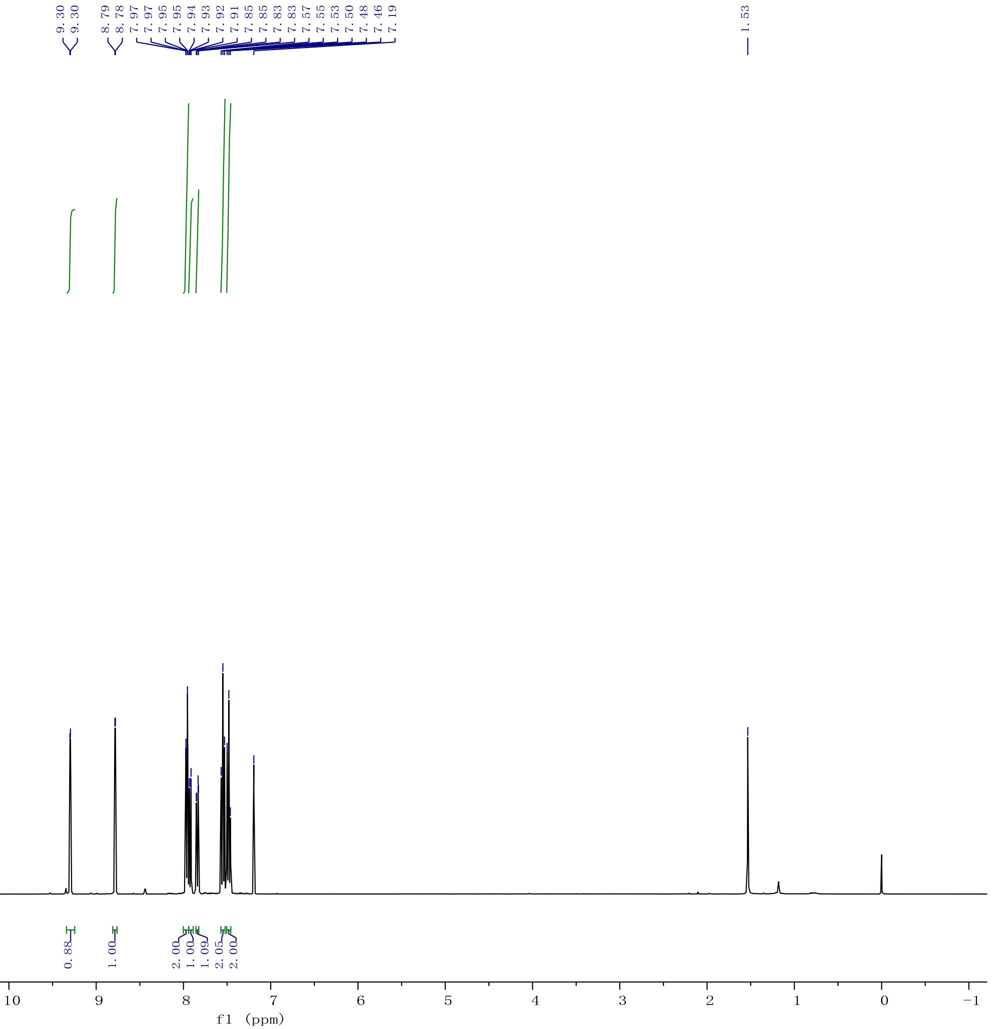

^{1}H NMR spectrum of **8-chloro-3-(phenylsulfonyl)quinoline 3u**

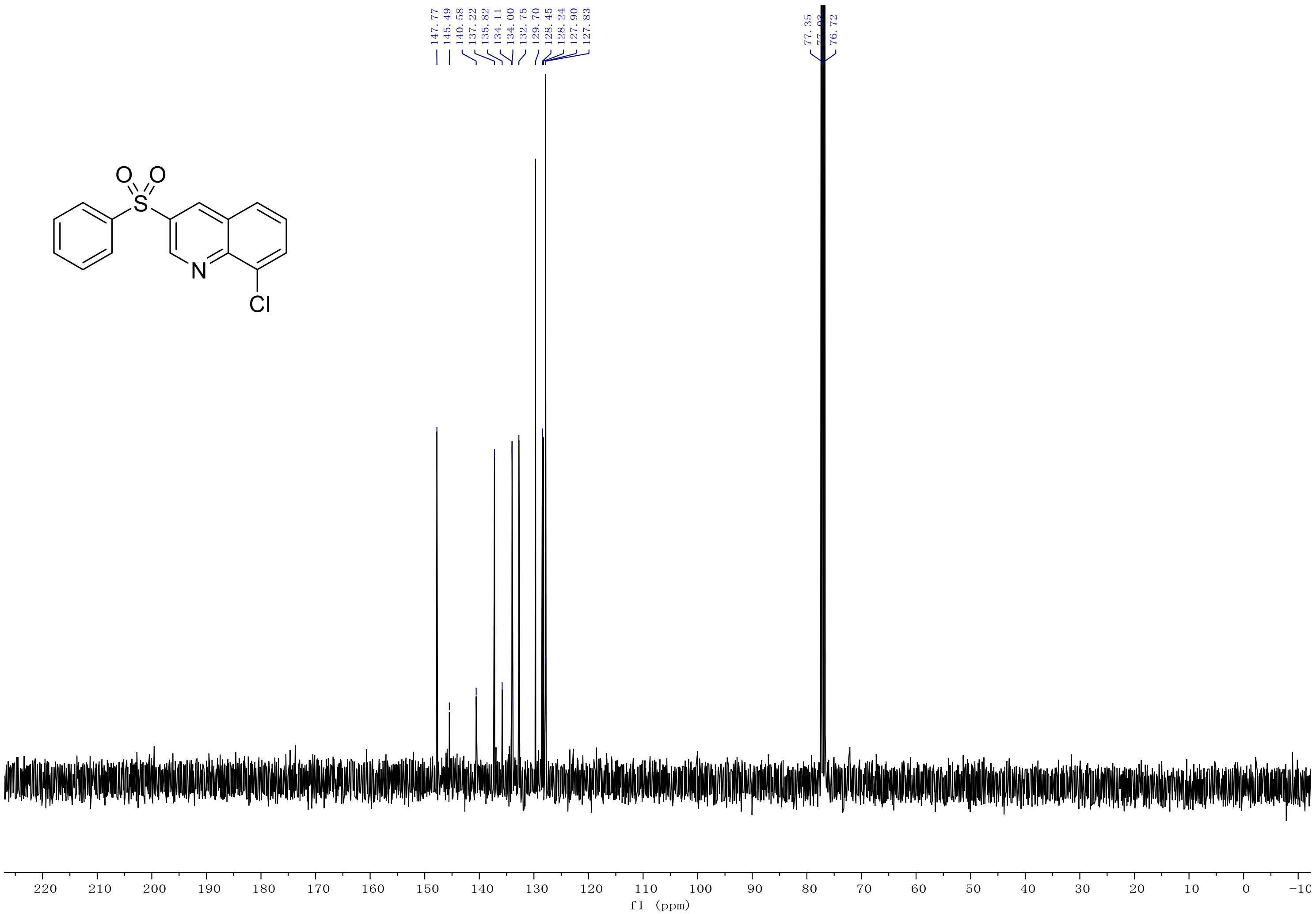

^{13}C NMR spectrum of **8-chloro-3-(phenylsulfonyl)quinoline 3u**

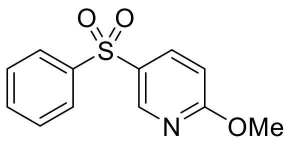

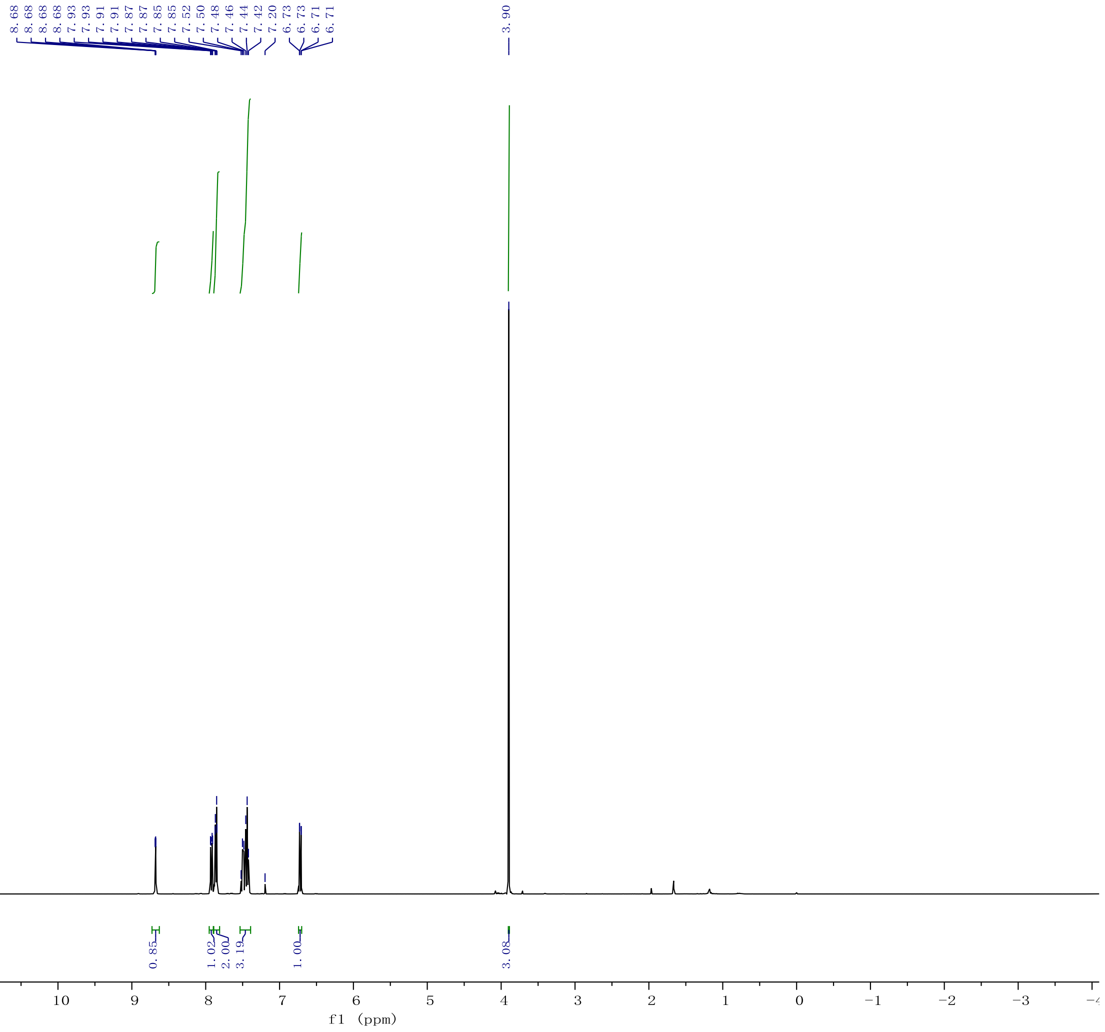

^{1}H NMR spectrum of **2-methoxy-5-(phenylsulfonyl)pyridine 3v**

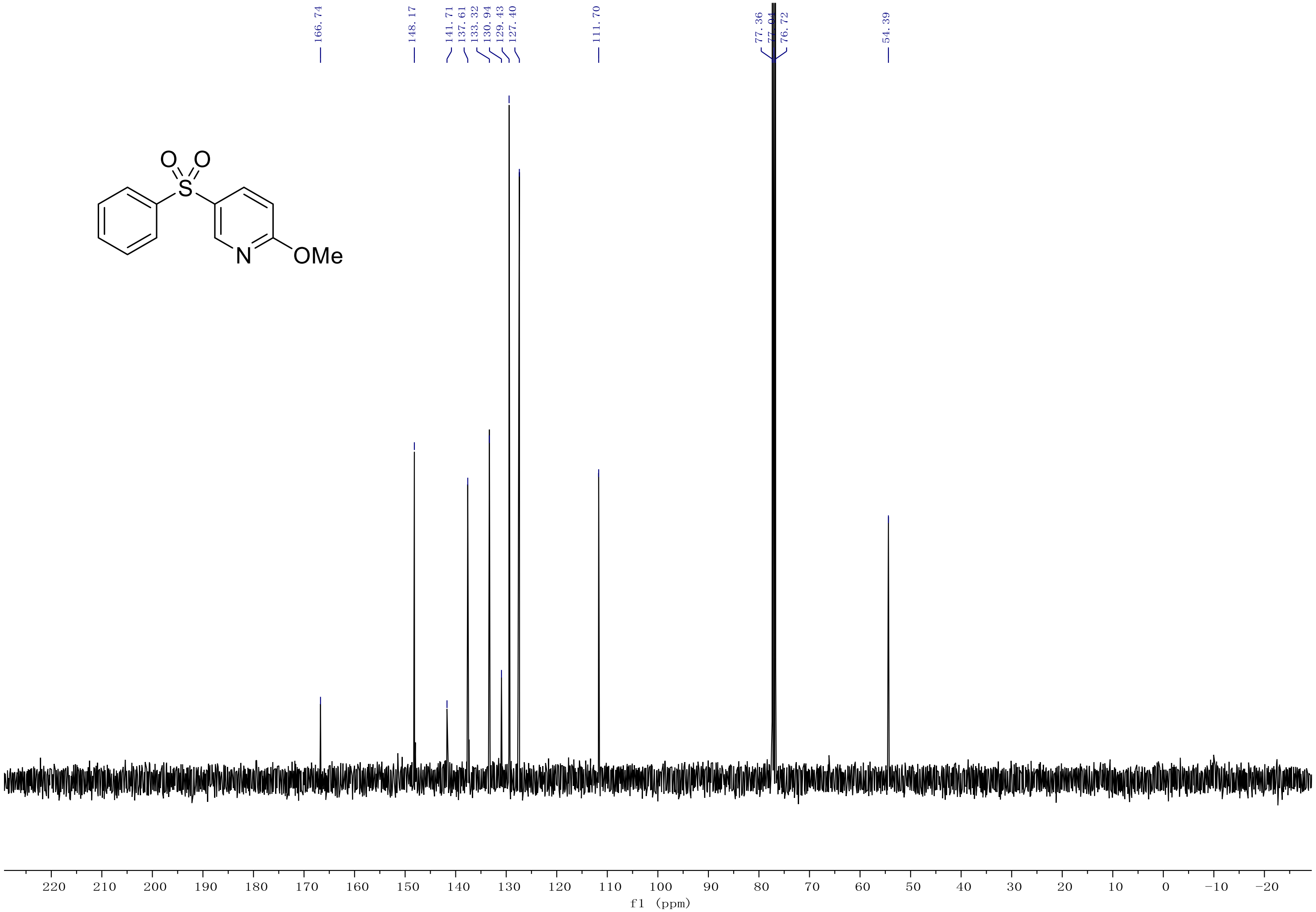

^{13}C NMR spectrum of **2-methoxy-5-(phenylsulfonyl)pyridine 3v**

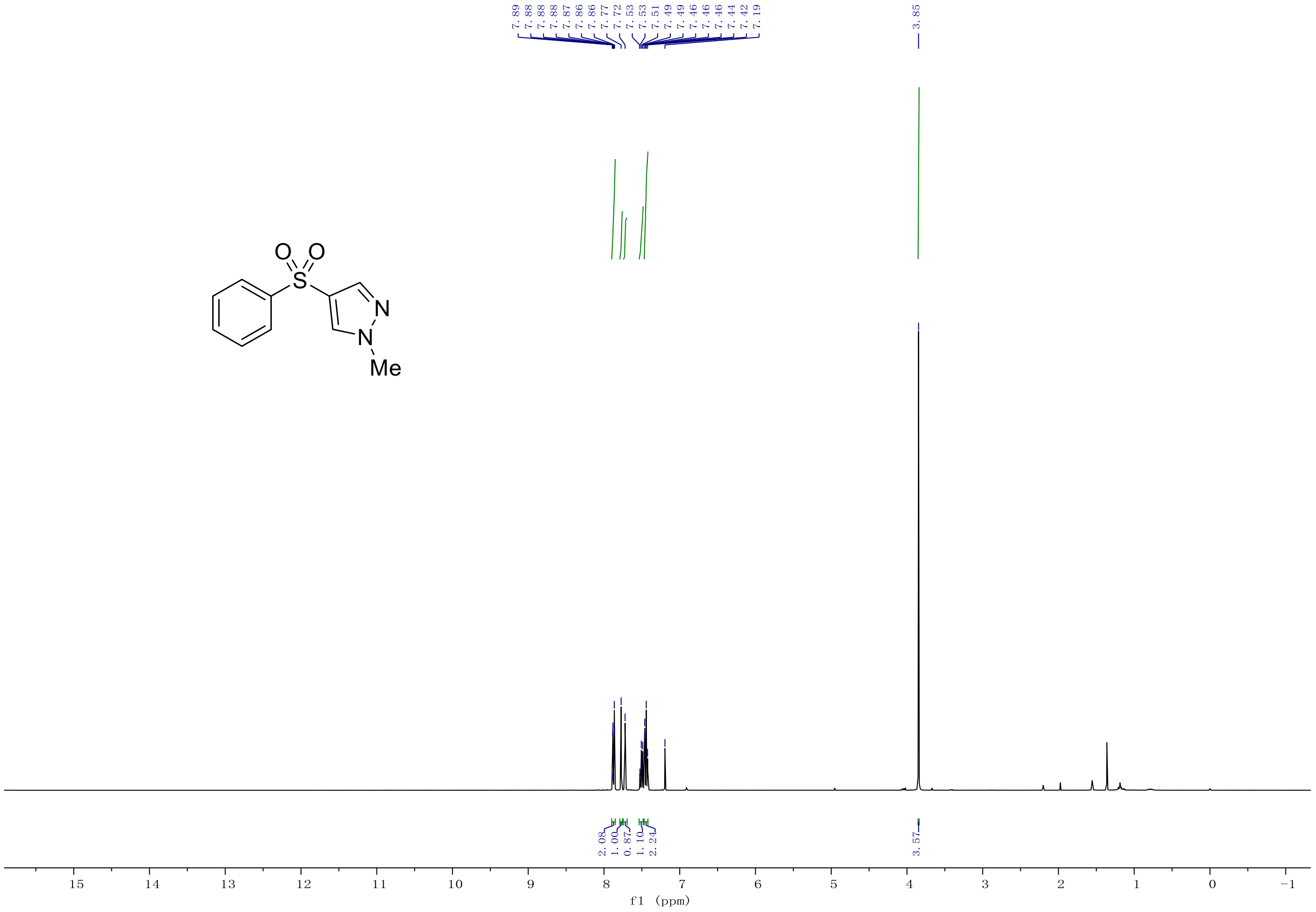

^{1}H NMR spectrum of **1-methyl-4-(phenylsulfonyl)pyrazole 3w**

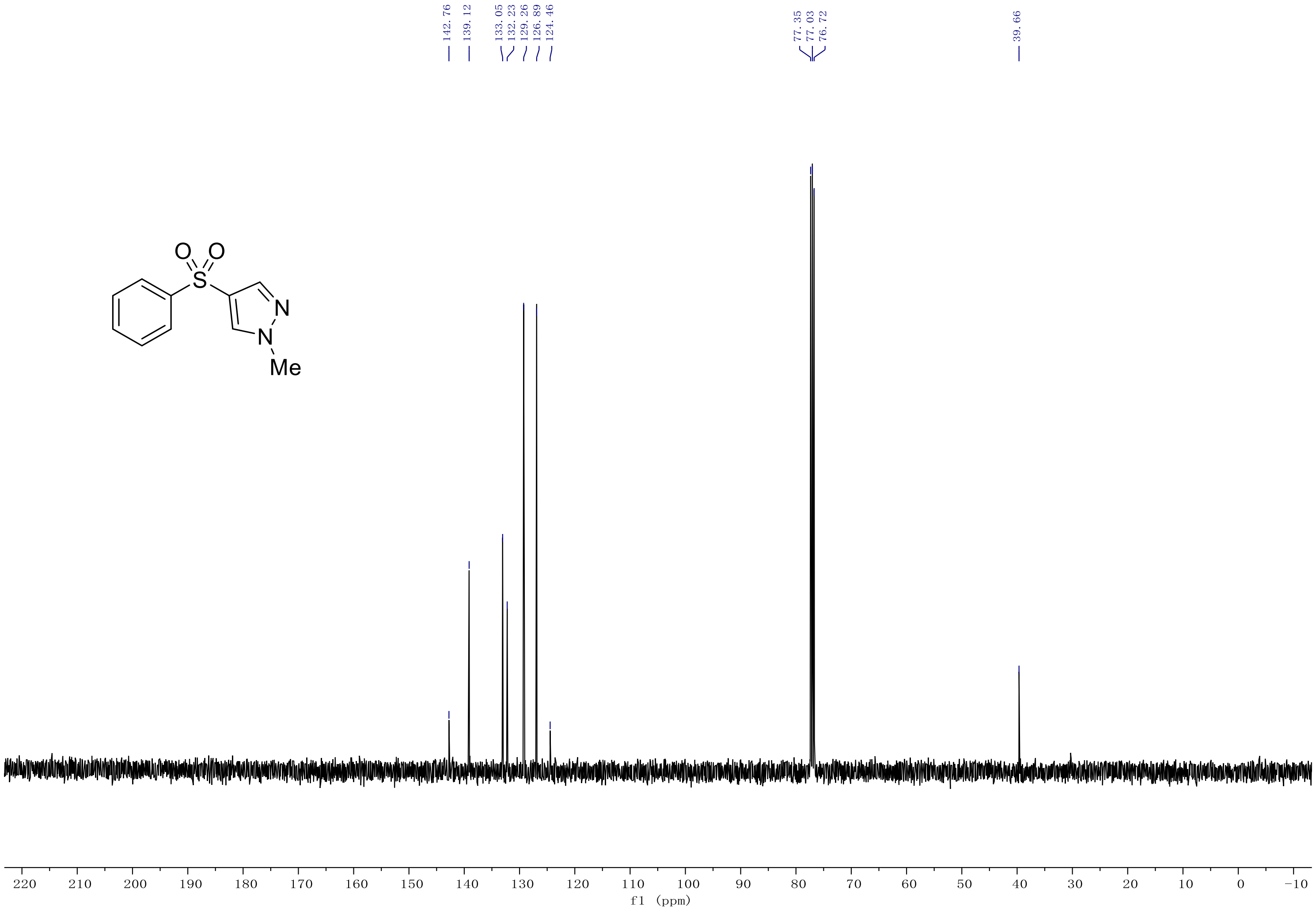

^{13}C NMR spectrum of **1-methyl-4-(phenylsulfonyl)pyrazole 3w**

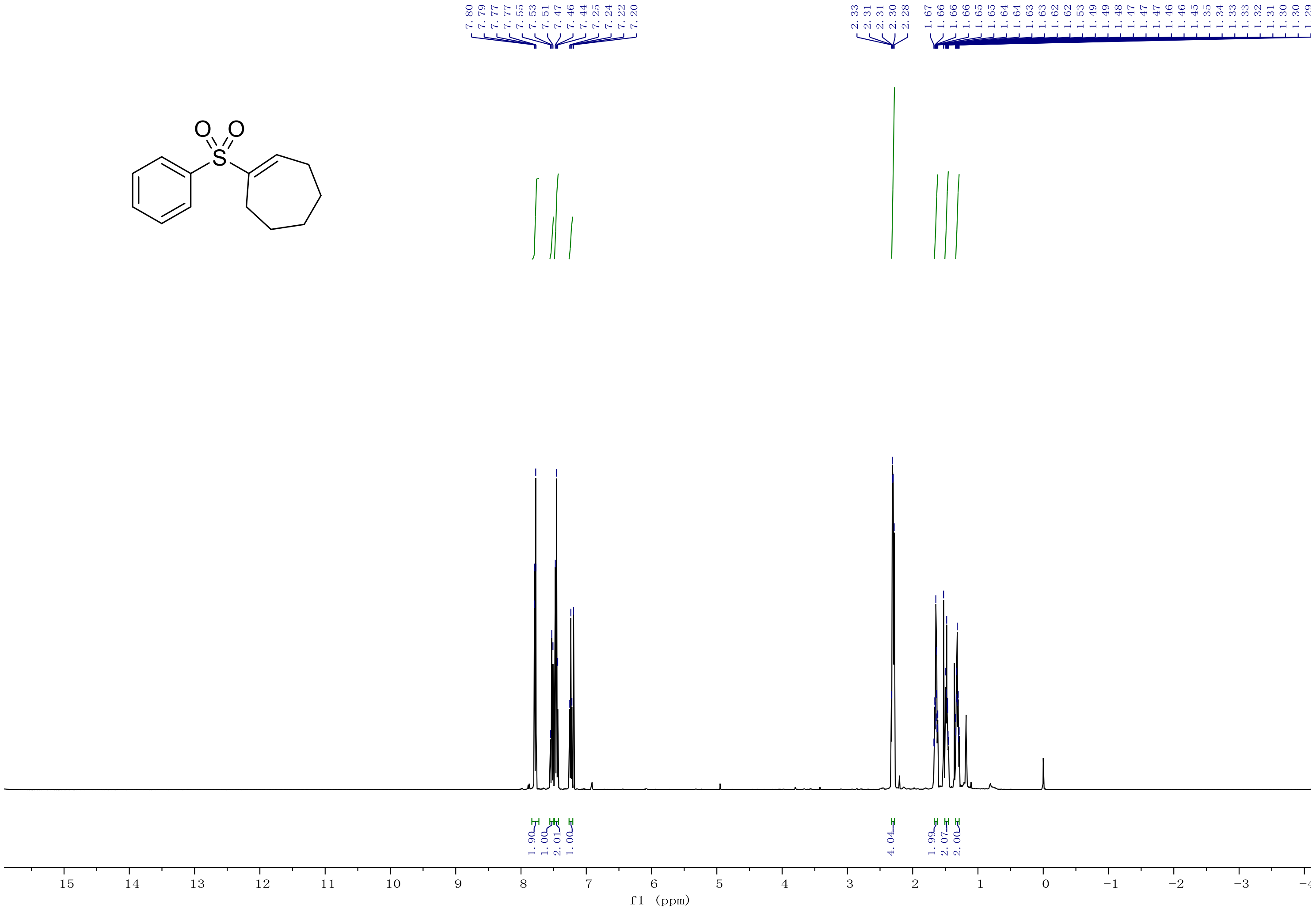

^{1}H NMR spectrum of **1-cyclohepten-1-yl-phenylsulfon 3x**

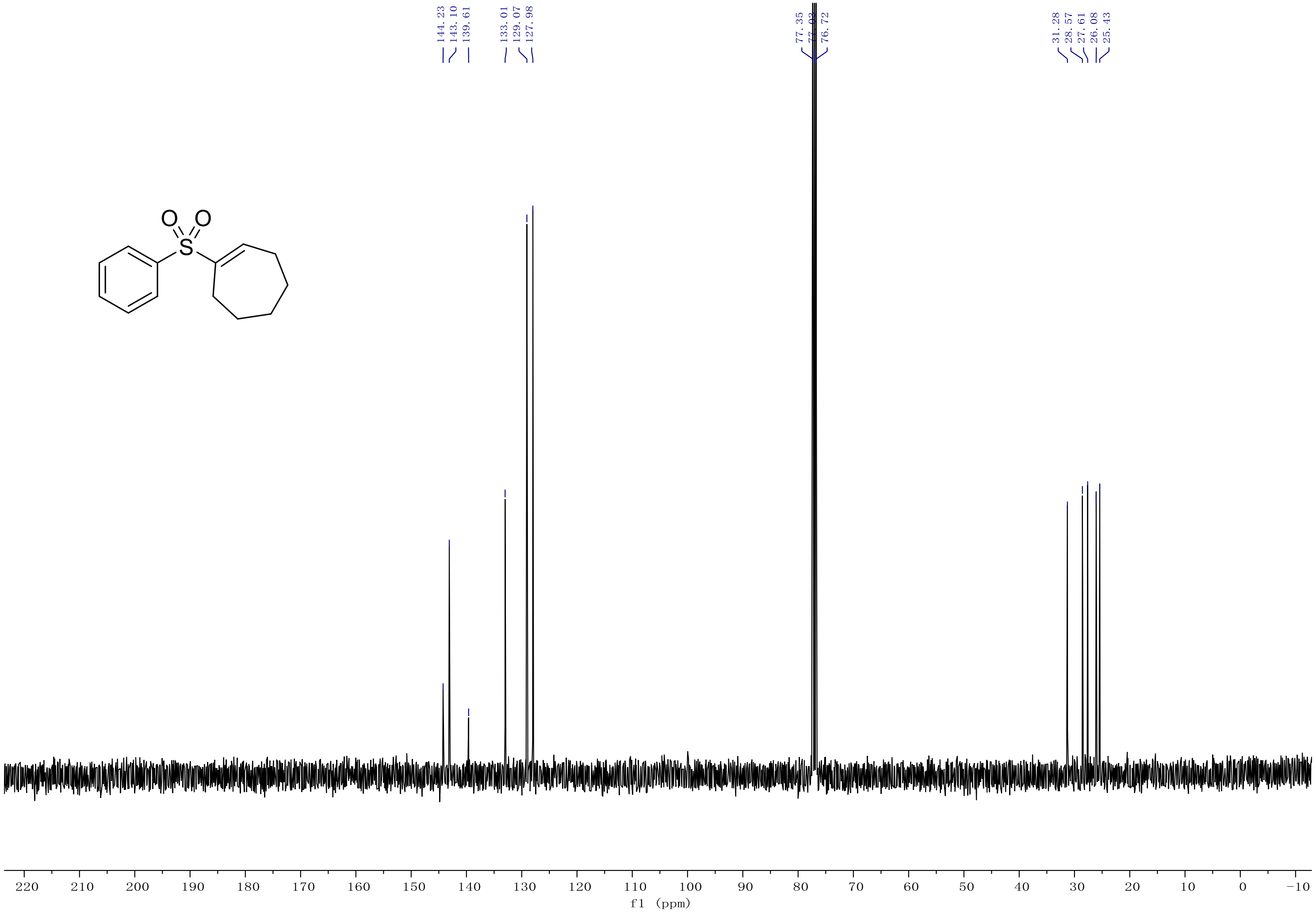

^{13}C NMR spectrum of **1-cyclohepten-1-yl-phenylsulfon 3x**

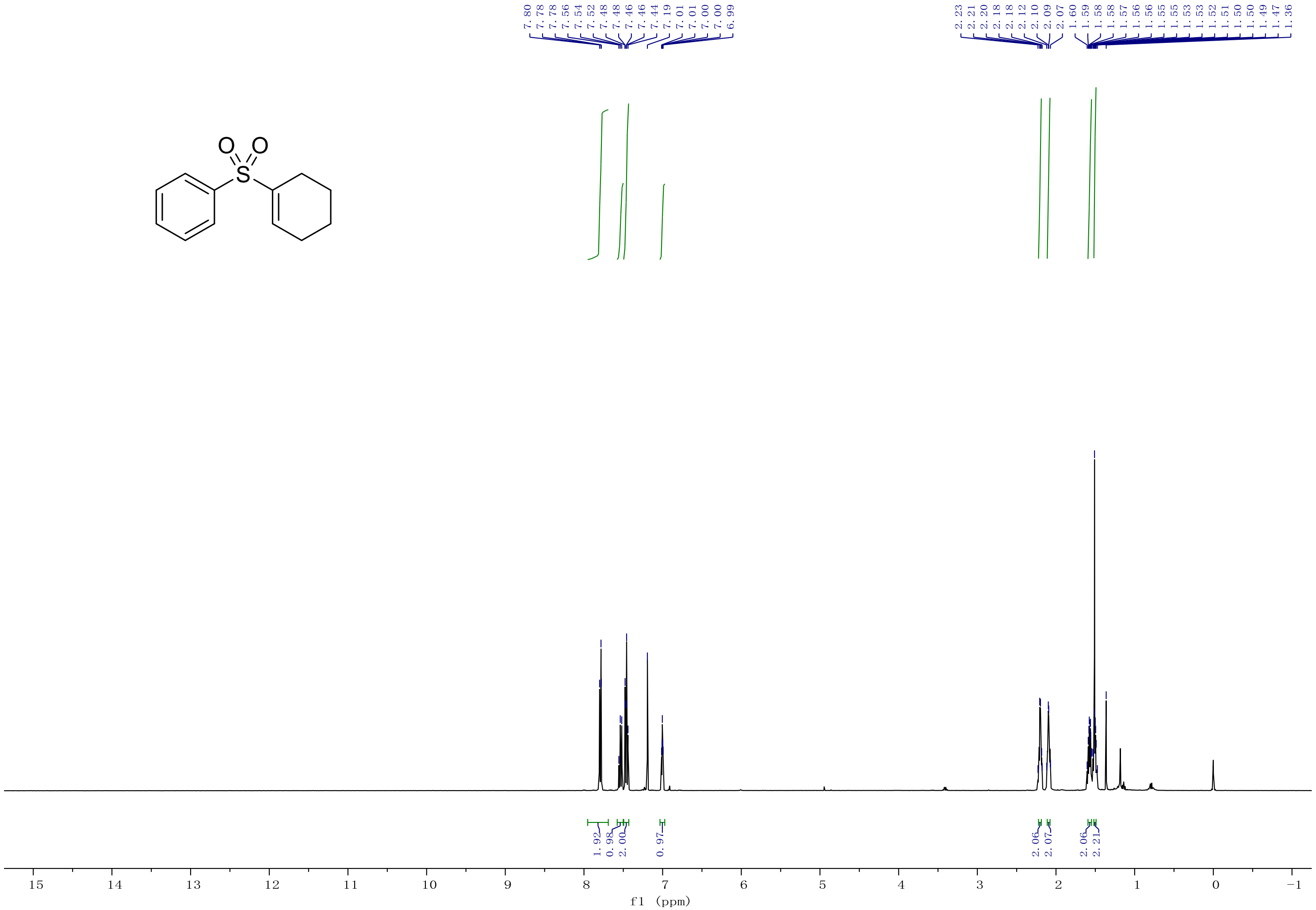

^{1}H NMR spectrum of **1-cyclohexen-1-yl-phenylsulfon 3y**

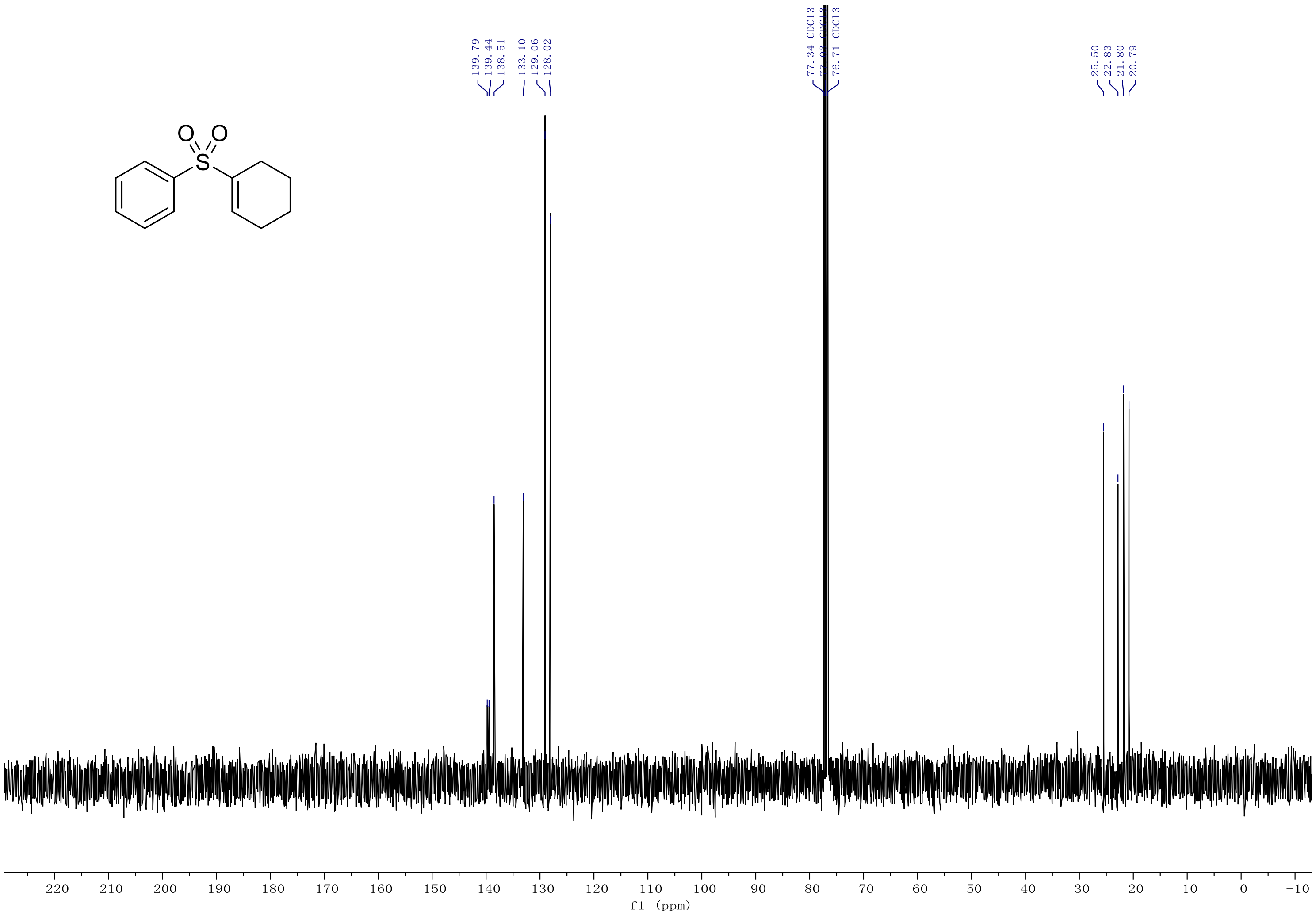

^{1}H NMR and ^{13}C NMR spectra of **1-cyclohexen-1-yl-phenylsulfon 3y**

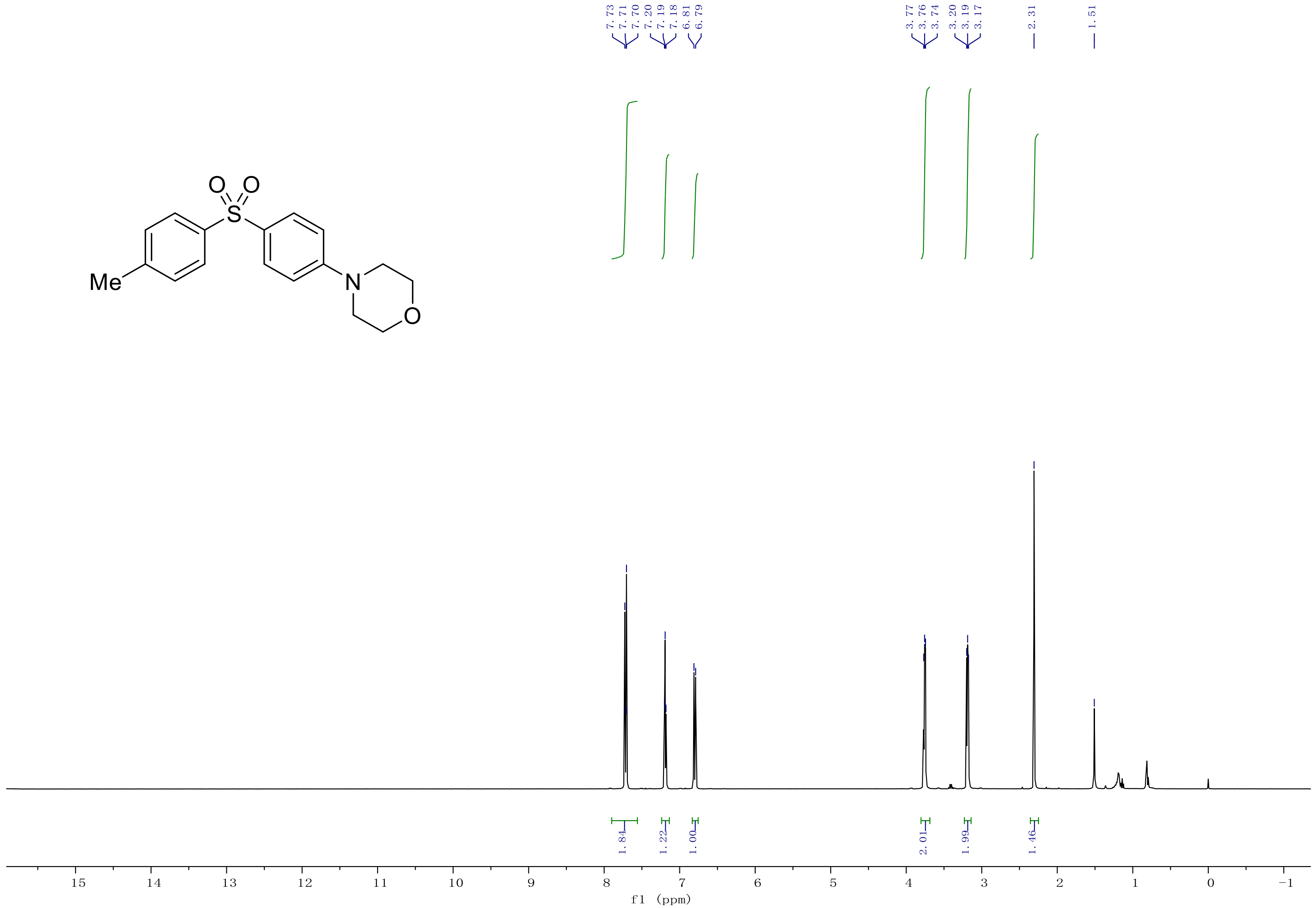

^{1}H NMR spectrum of **4-(4-tosylphenyl)morpholine 4a**

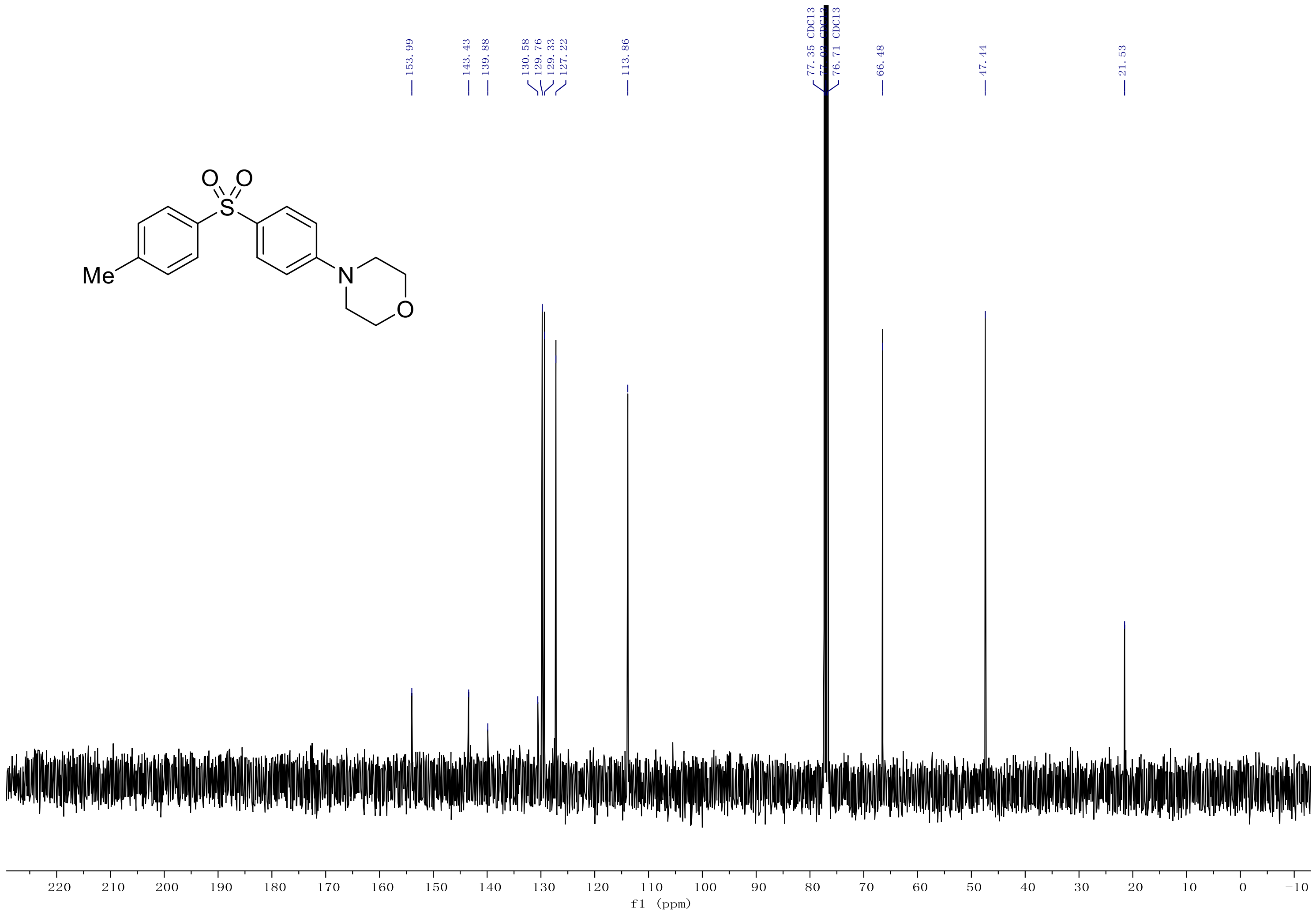

^{13}C NMR spectrum of **4-(4-tosylphenyl)morpholine 4a**

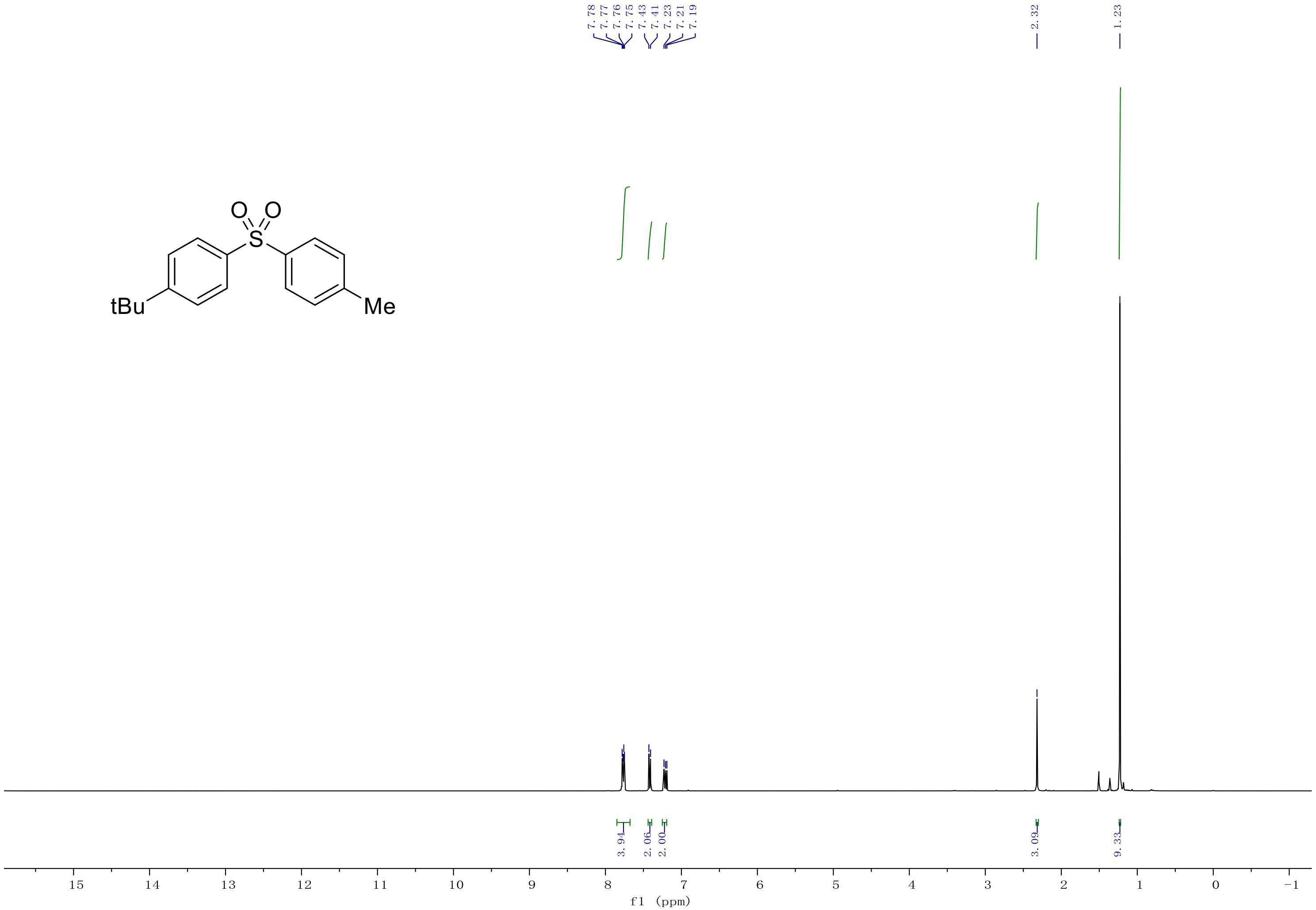

^{1}H NMR spectrum of **(4-*t*-butylphenyl)-*p*-tolyl sulfone 4b**

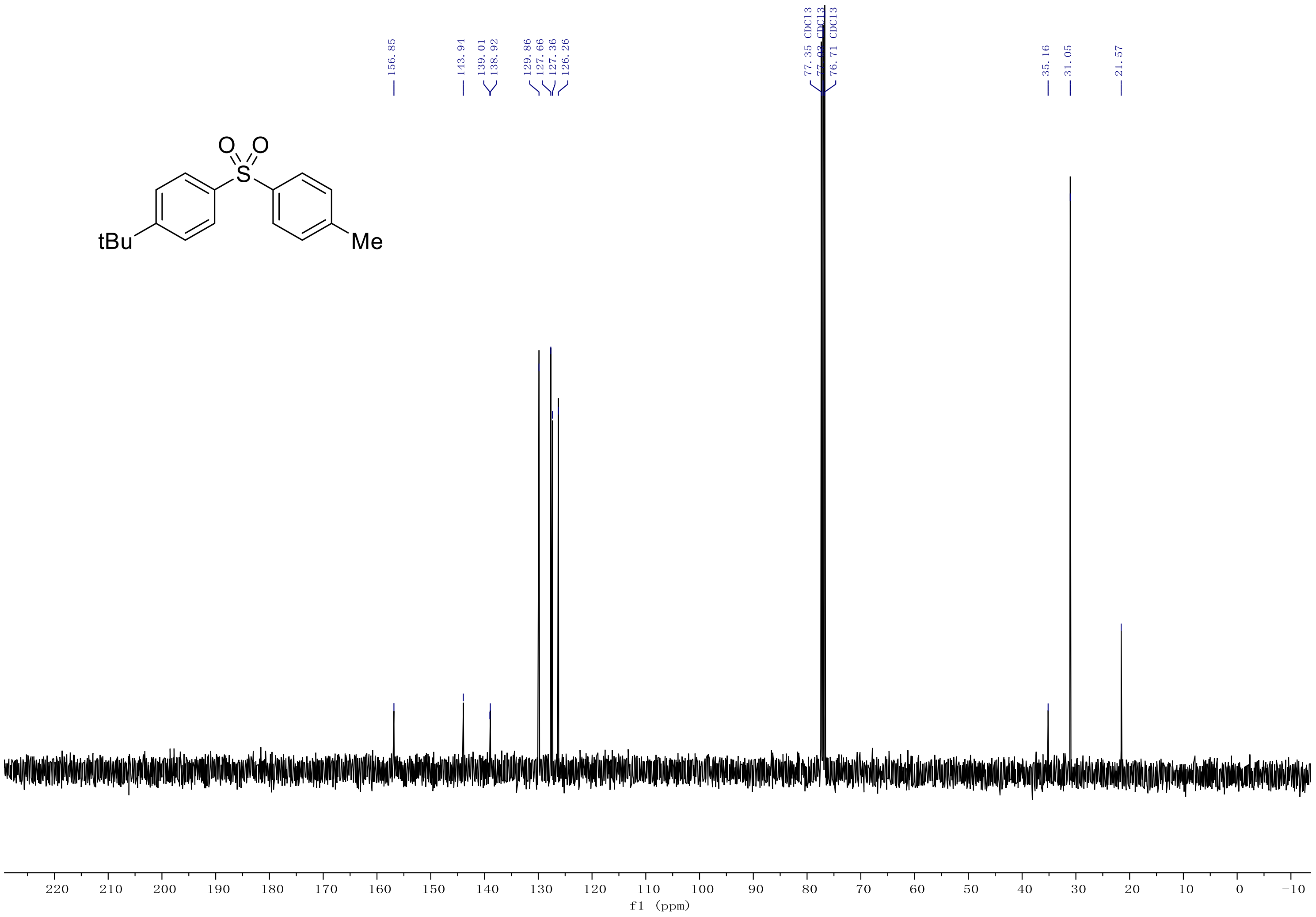

^{13}C NMR spectrum of **(4-*t*-butylphenyl)-*p*-tolyl sulfone 4b**

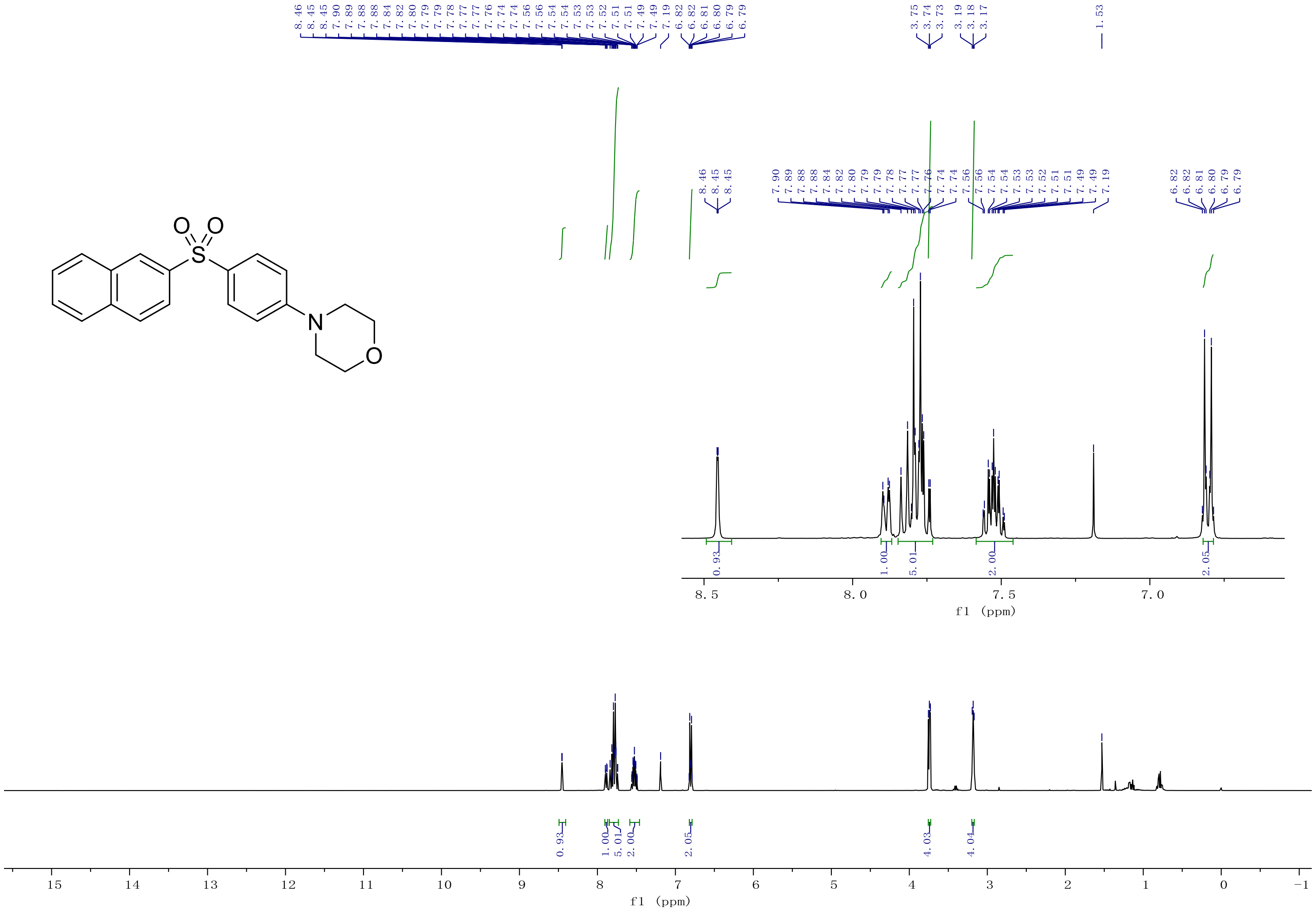

^{1}H NMR spectrum of **4-(4-(naphthalen-2-ylsulfonyl)phenyl)morpholine 4c**

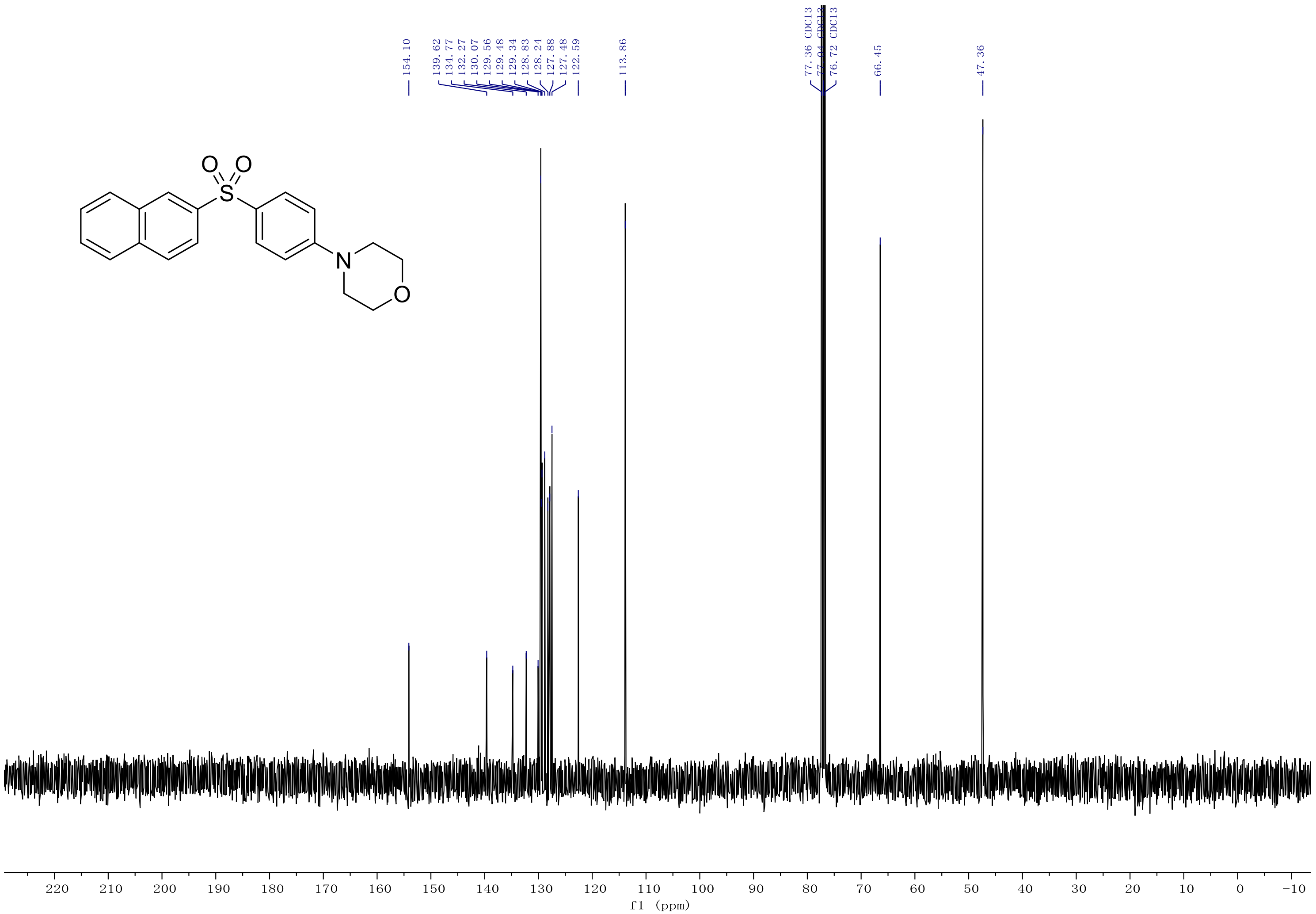

^{13}C NMR spectrum of **4-(4-(naphthalen-2-ylsulfonyl)phenyl)morpholine 4c**

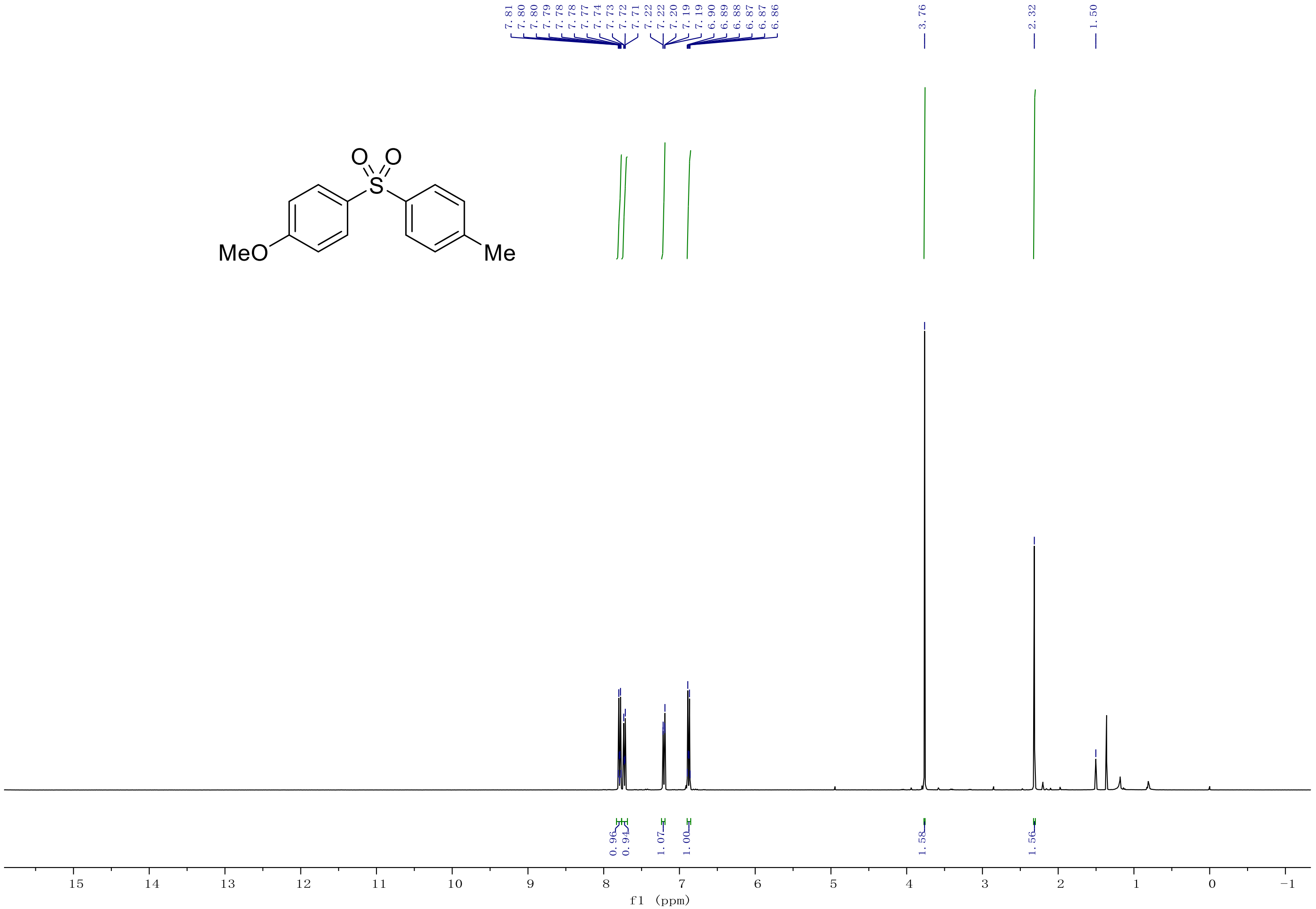

^{1}H NMR spectrum of **4-tolyl 4-methoxyphenyl sulfone 4d**

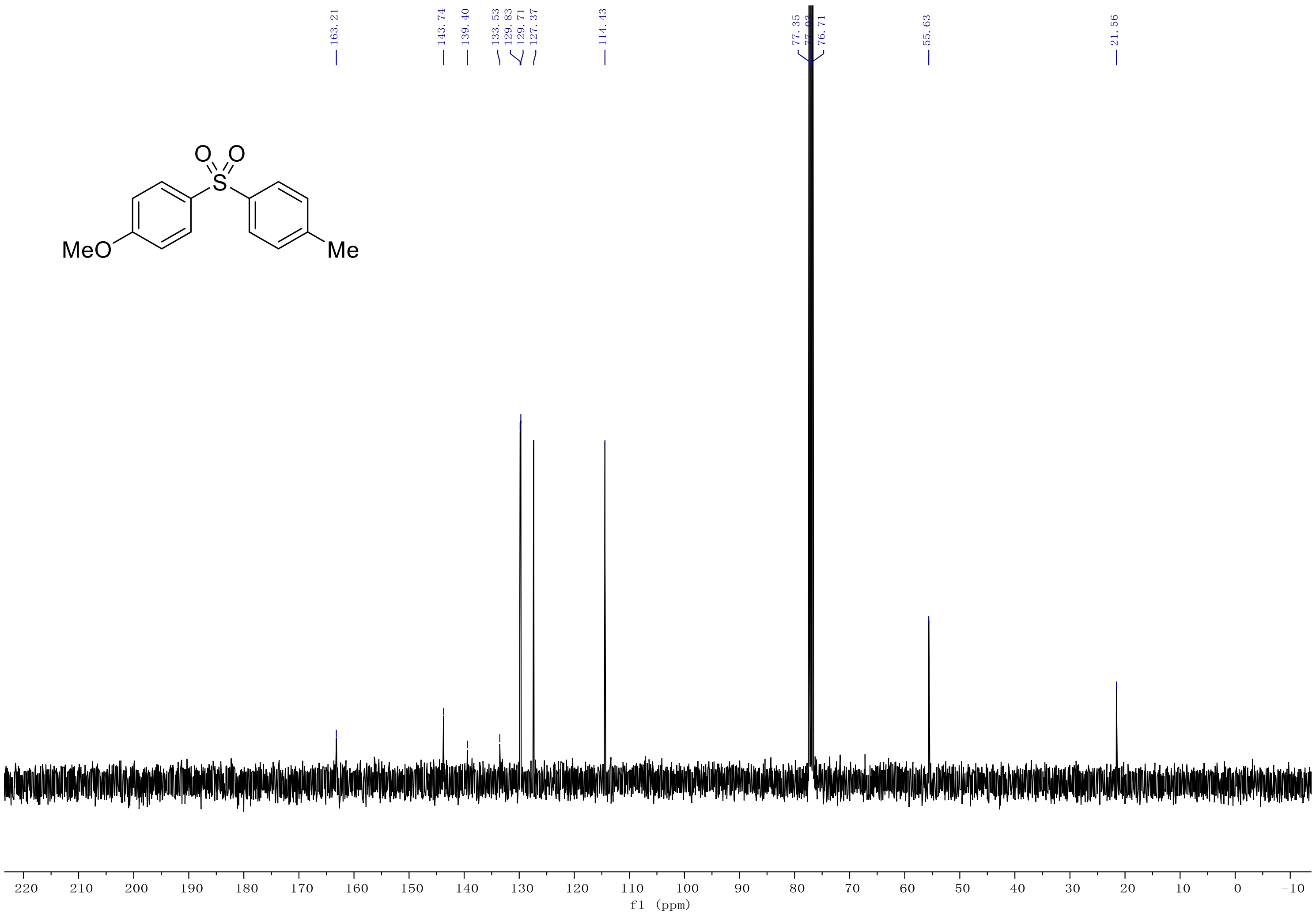

^{13}C NMR spectrum of **4-tolyl 4-methoxyphenyl sulfone 4d**

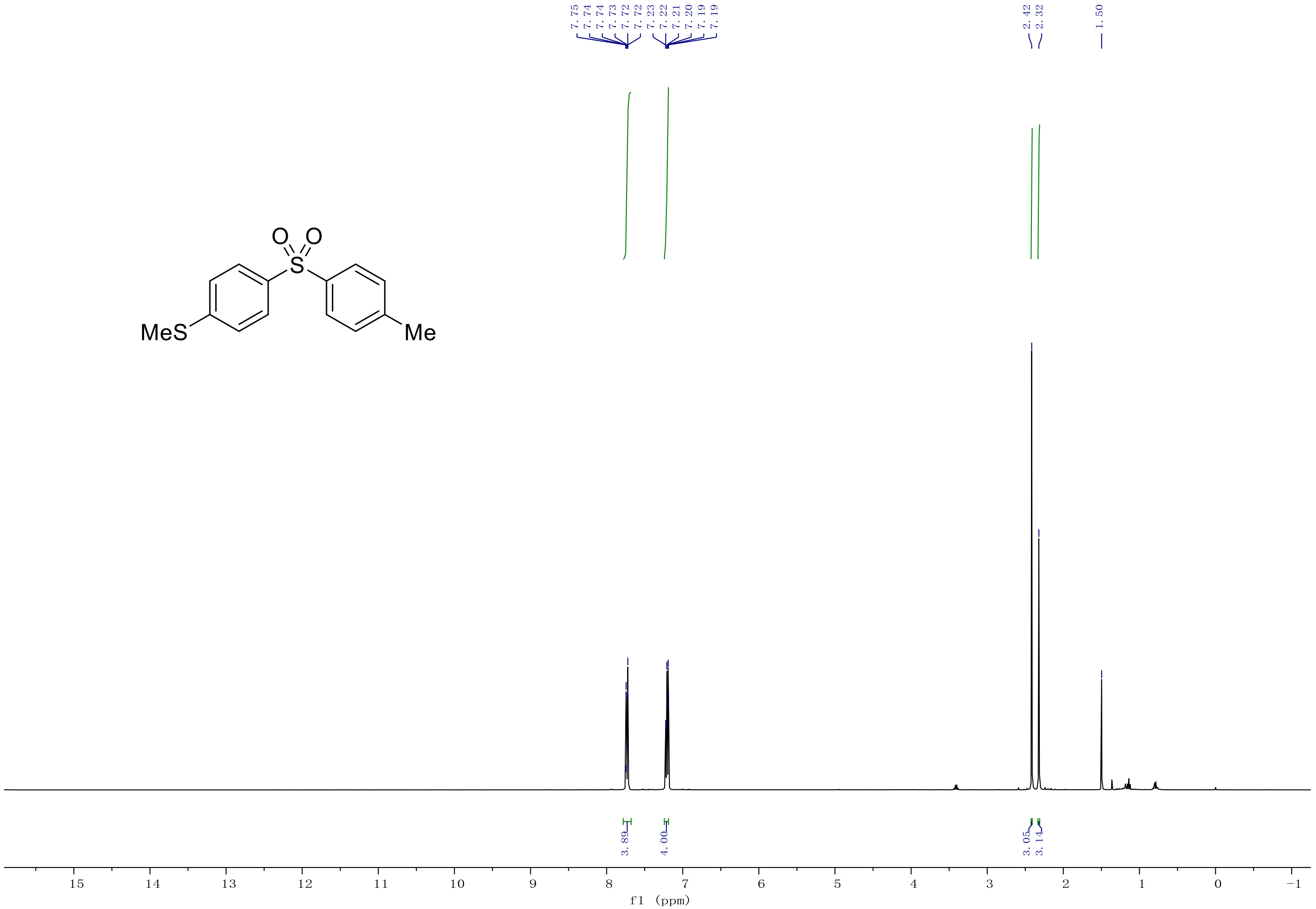

^{1}H NMR spectrum of **4-tolyl 4-methylthiophenyl sulfone 4e**

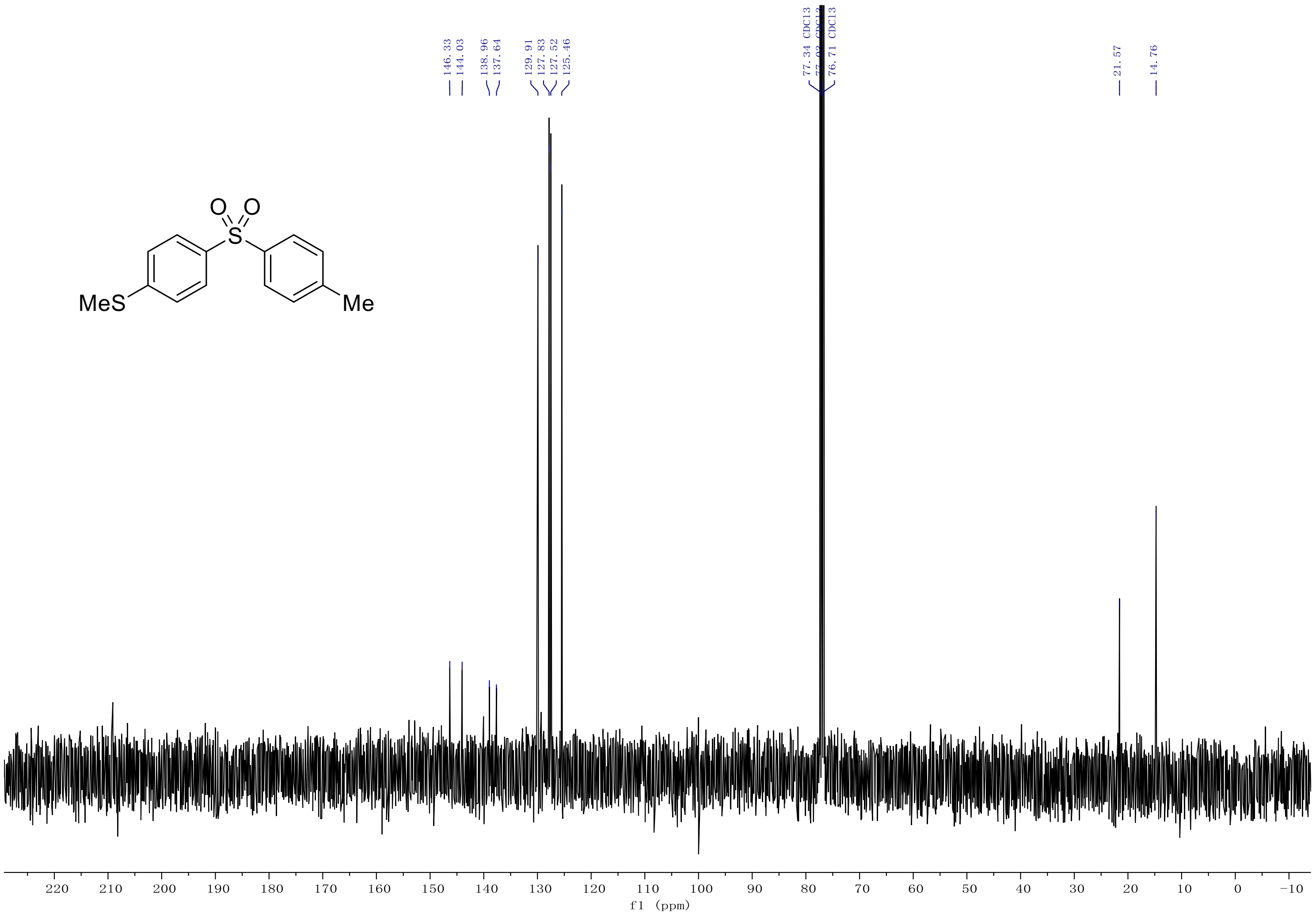

^{13}C NMR spectrum of **4-tolyl 4-methylthiophenyl sulfone 4e**

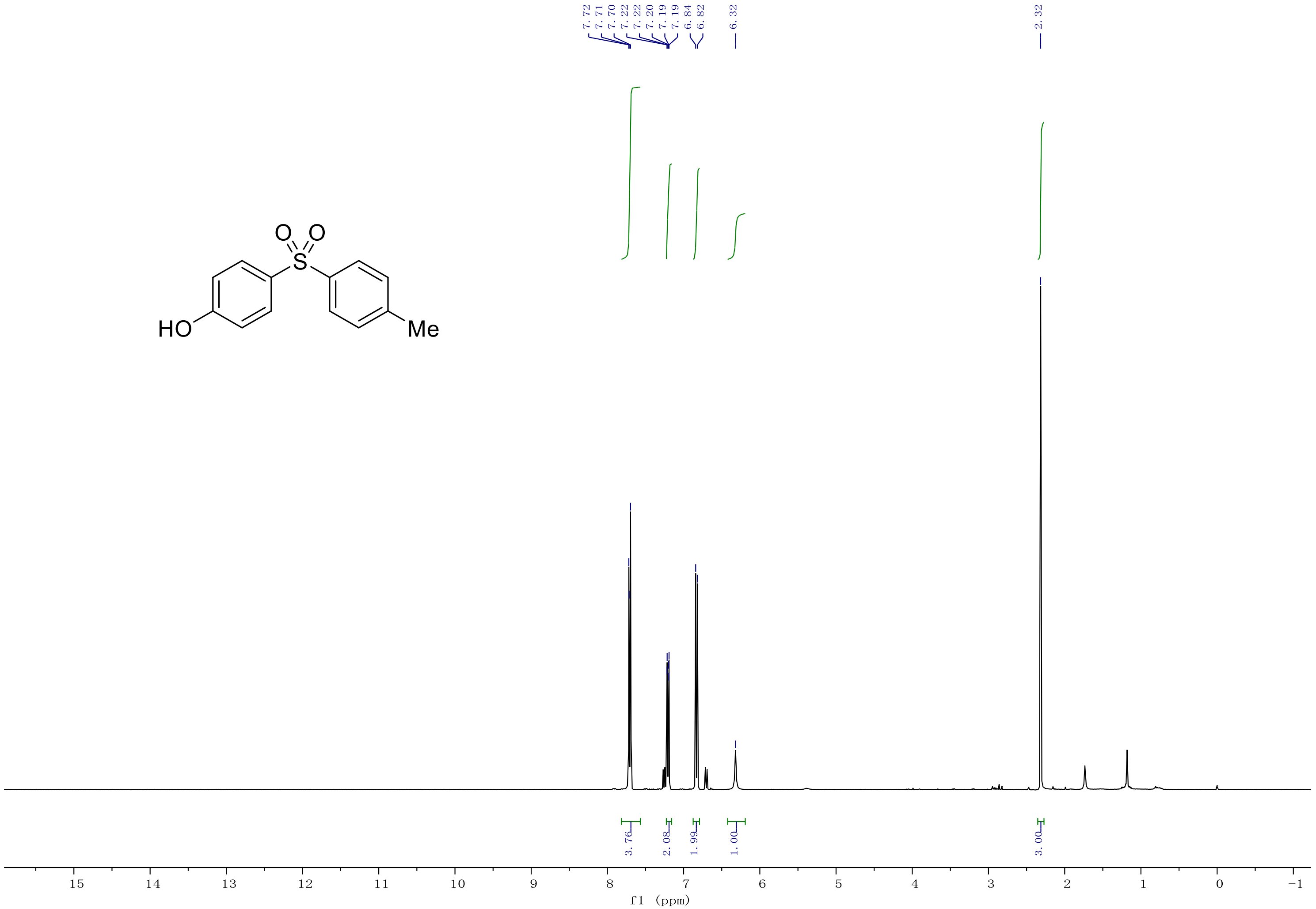

^{1}H NMR and ^{13}C NMR spectra of **4-tosylphenol 4f**

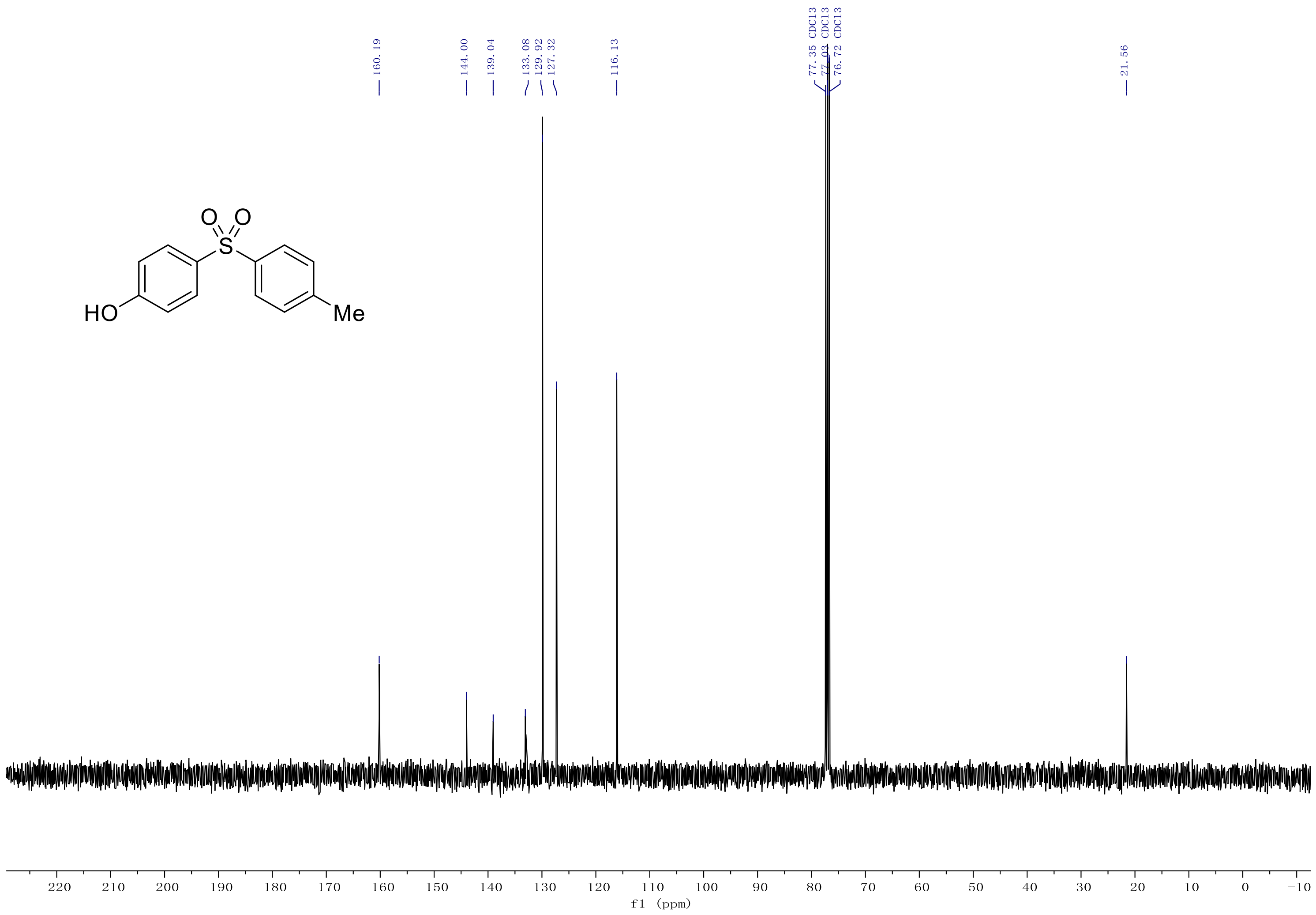

^{13}C NMR spectrum of **4-tosylphenol 4f**

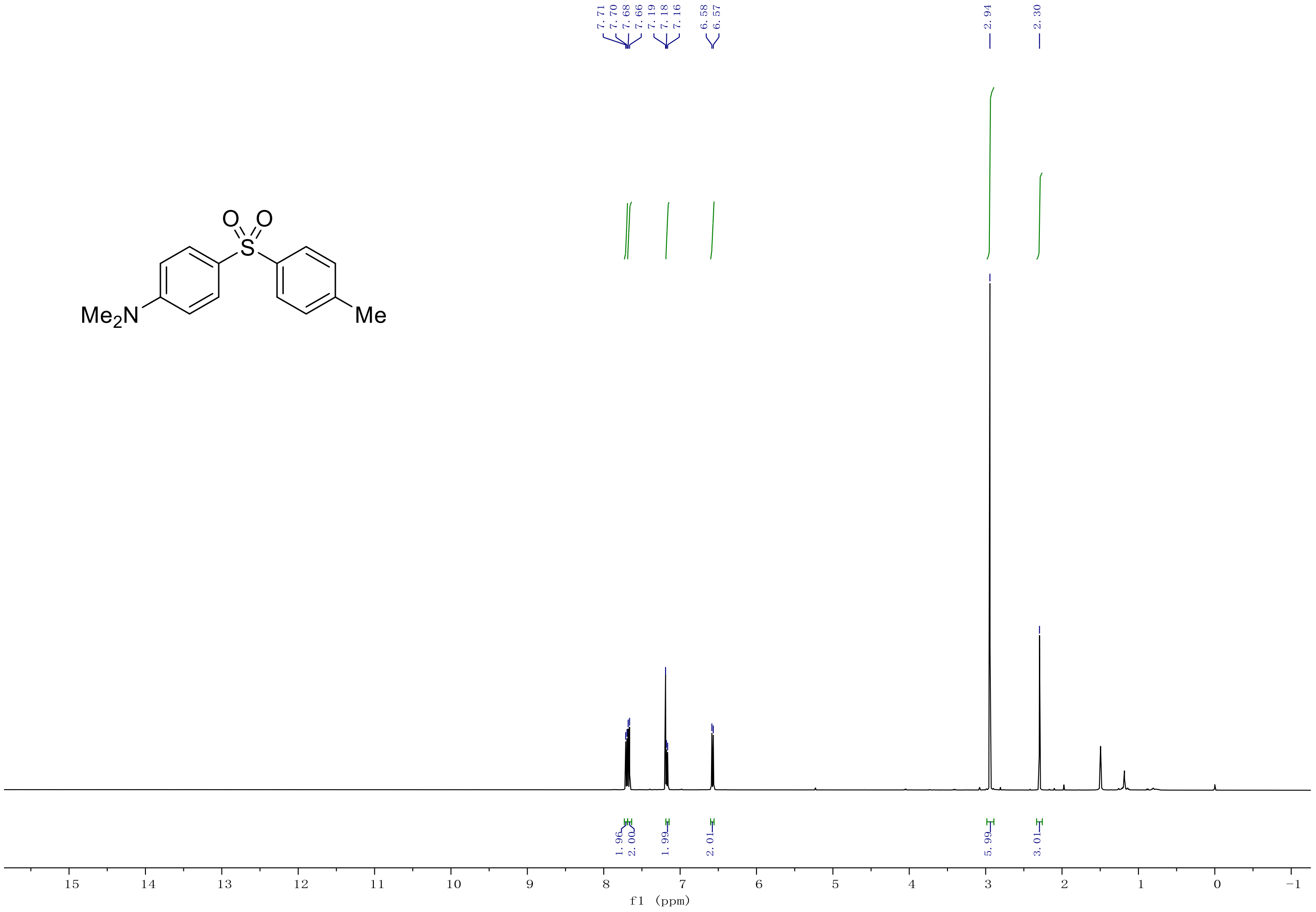

^{1}H NMR spectrum of ***N,N*-dimethyl-4-tosylaniline 4g**

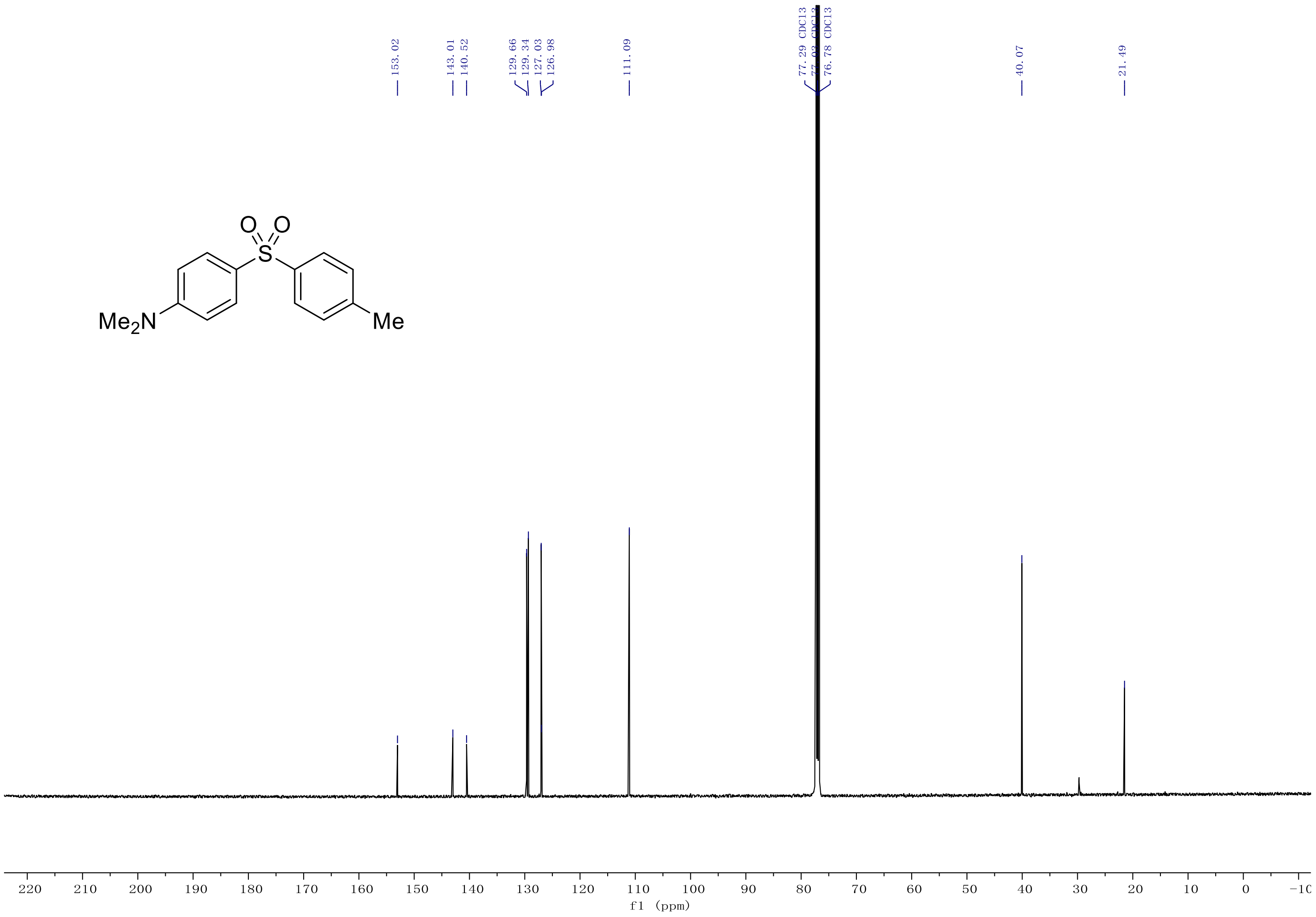

^{13}C NMR spectrum of ***N*,*N*-dimethyl-4-tosylaniline 4g**

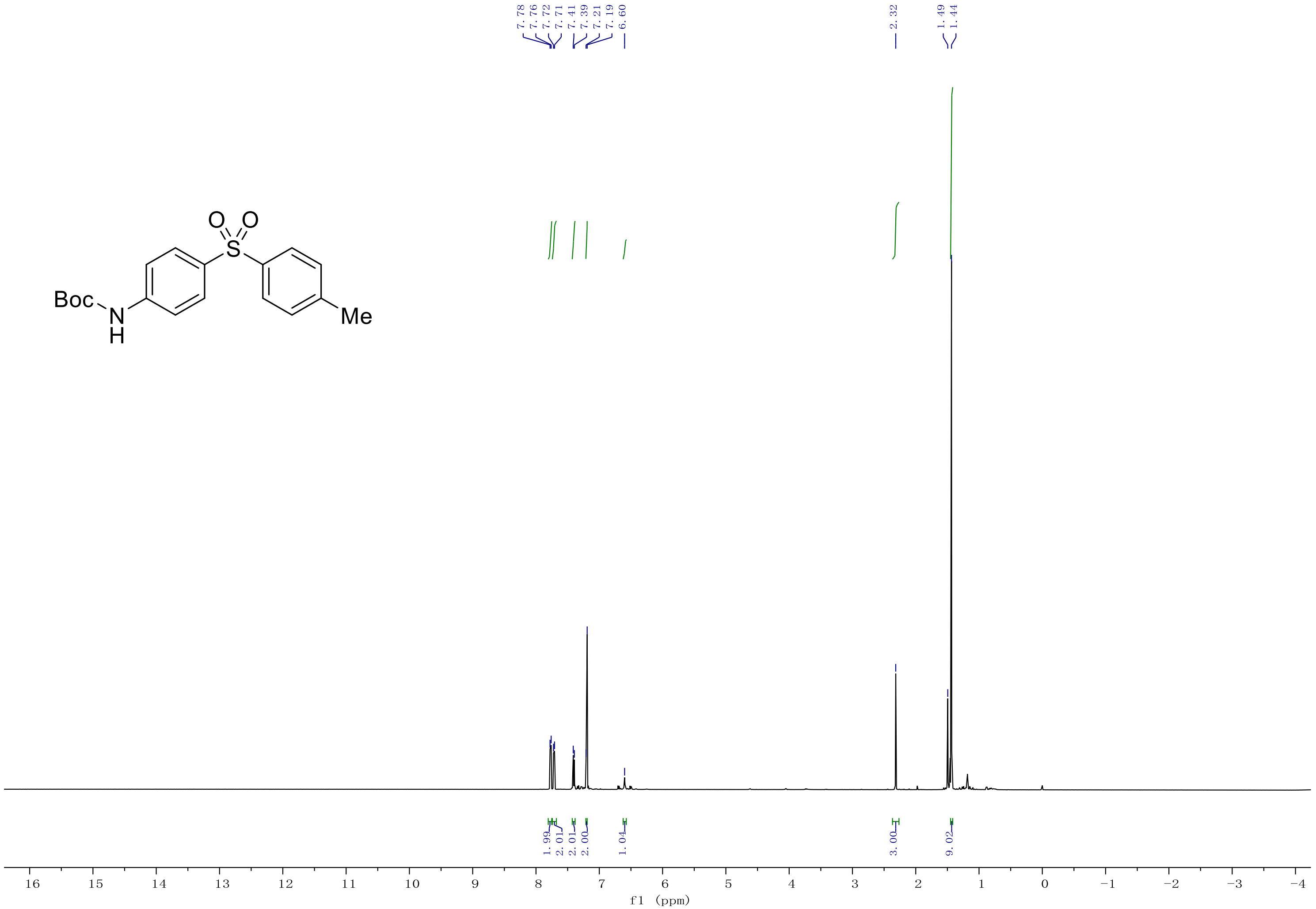

^{1}H NMR spectrum of ***tert*-butyl (4-tosylphenyl)carbamate 4h**

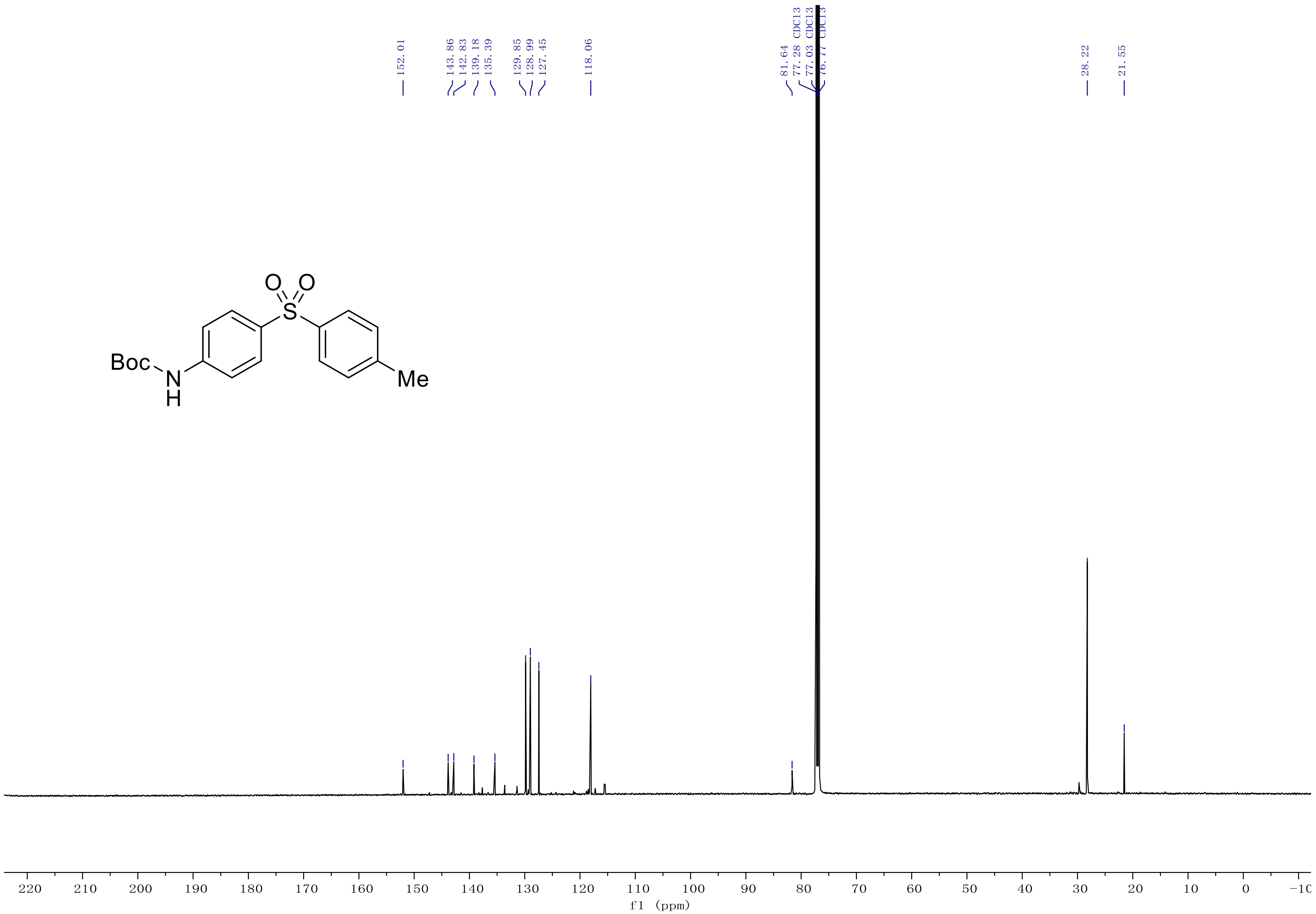

^{13}C NMR spectrum of ***tert*-butyl (4-tosylphenyl)carbamate 4h**

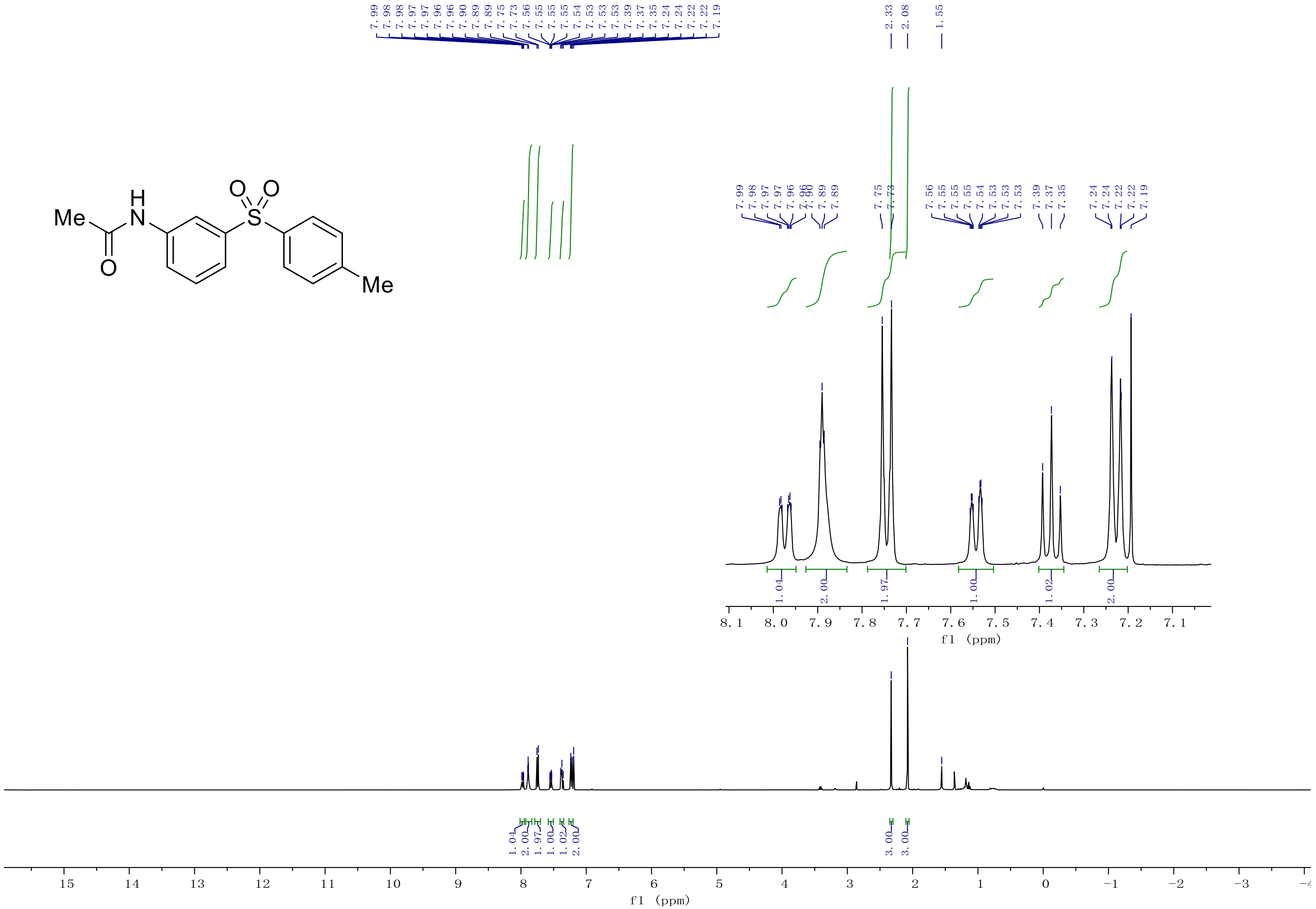

^{1}H NMR spectrum of ***N*-(3-Tosylphenyl)acetamide 4i**

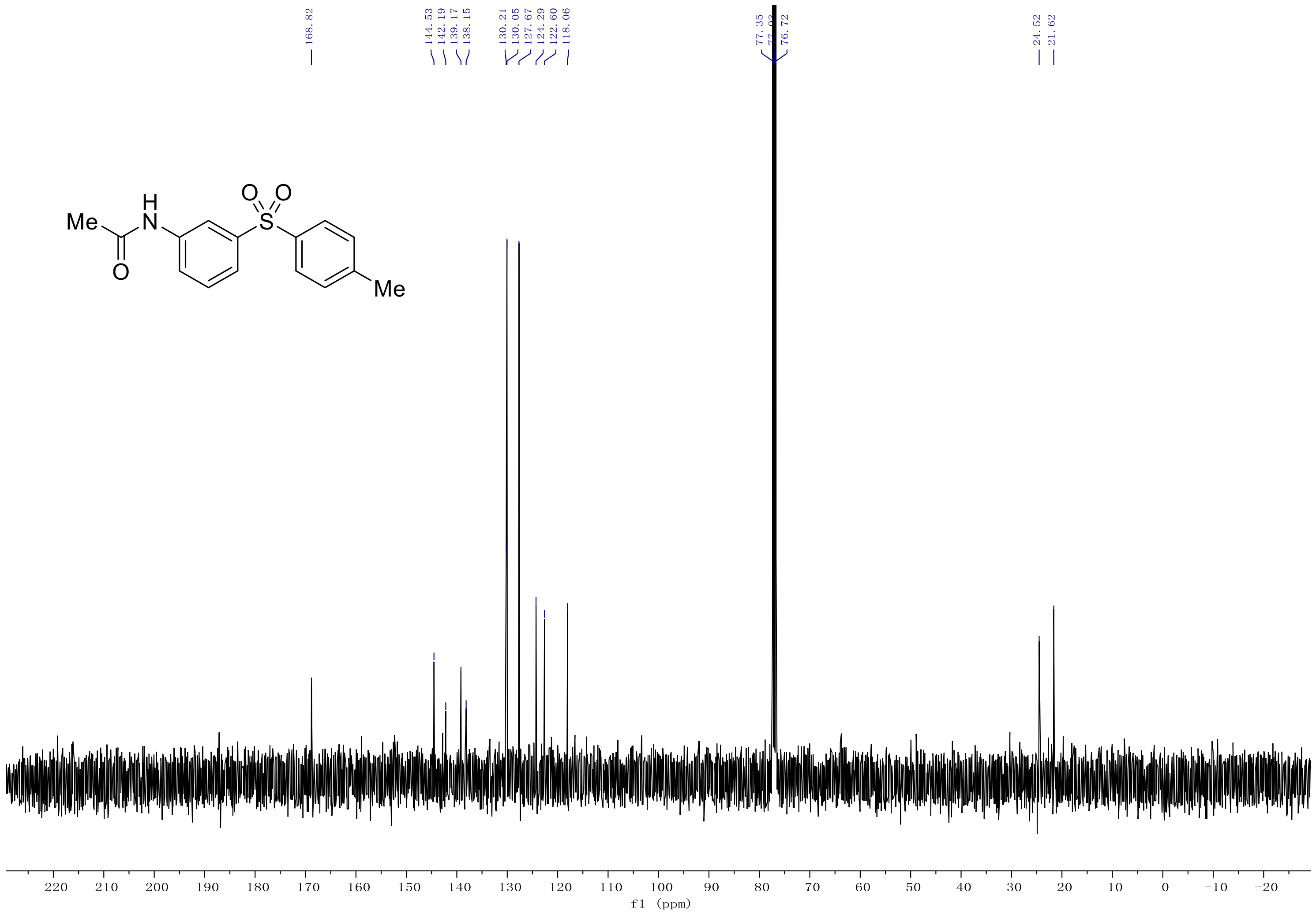

^{1}H NMR spectrum of ***N*-(3-tosylphenyl)acetamide 4i**

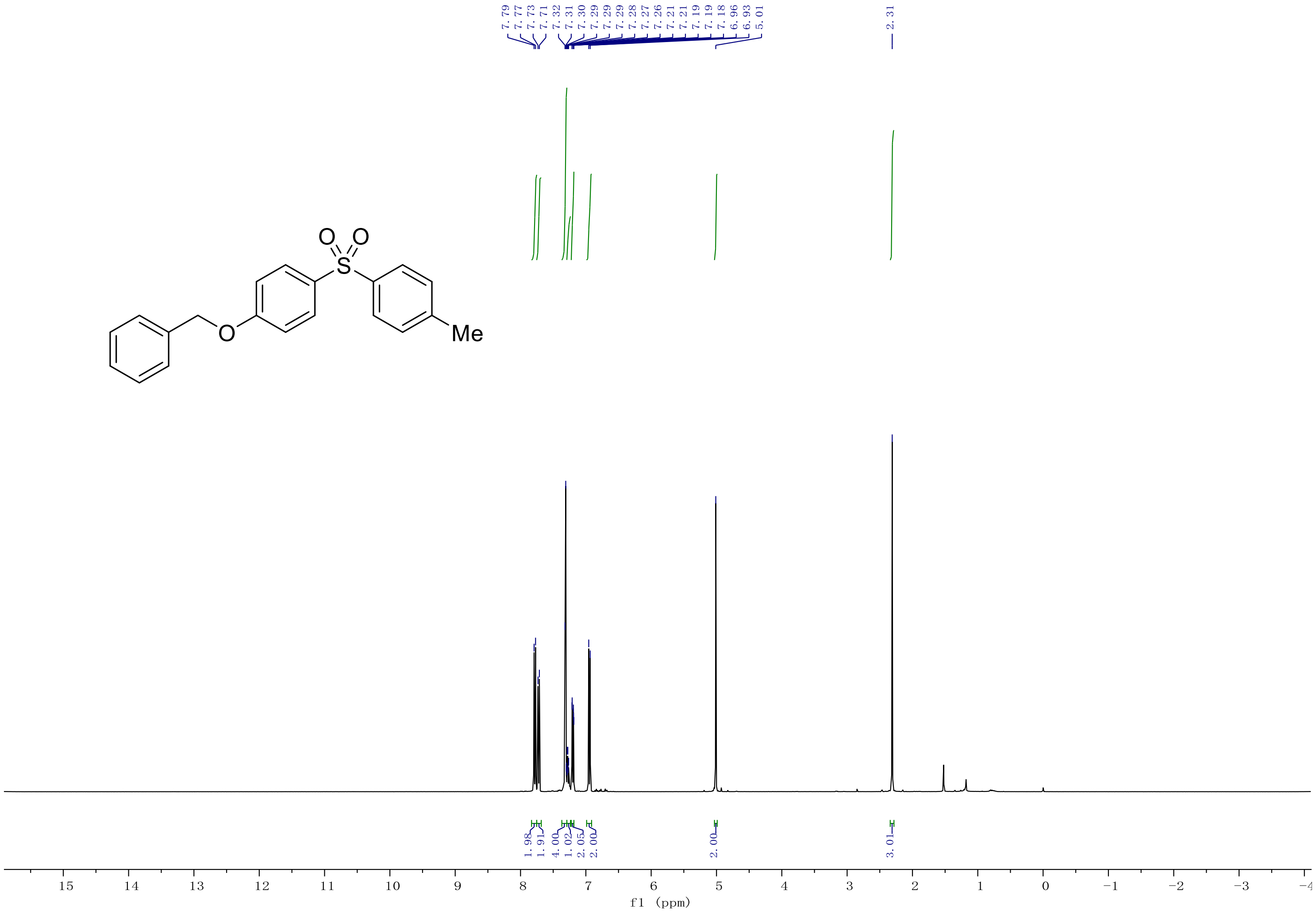

^{1}H NMR spectrum of **1-(benzyloxy)-4-tosylbenzene 4j**

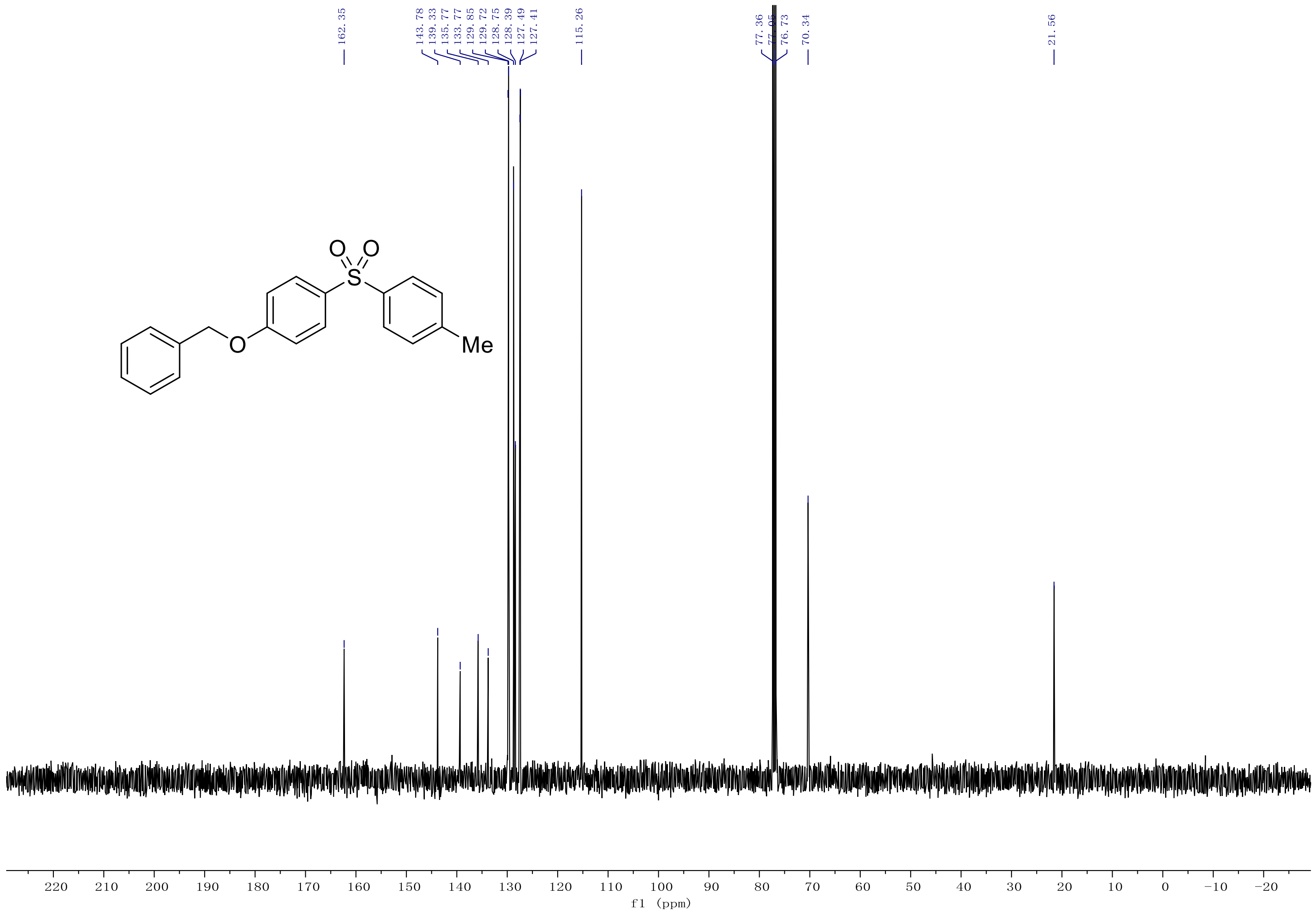

^{13}C NMR spectrum of **1-(benzyloxy)-4-tosylbenzene 4j**

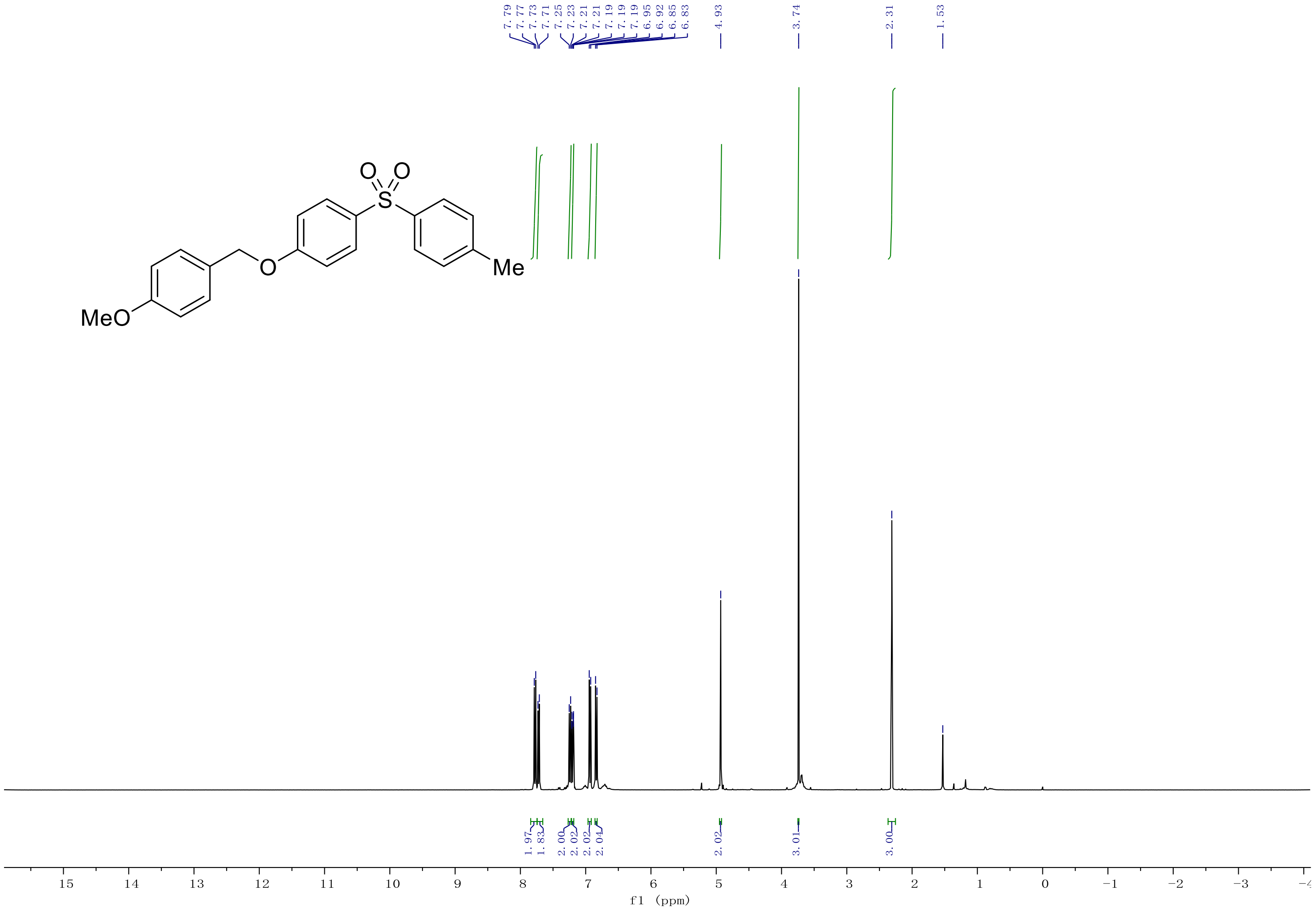

^{1}H NMR spectrum of **1-methoxy-4-((4-tosylphenoxy)methyl)benzene 4k**

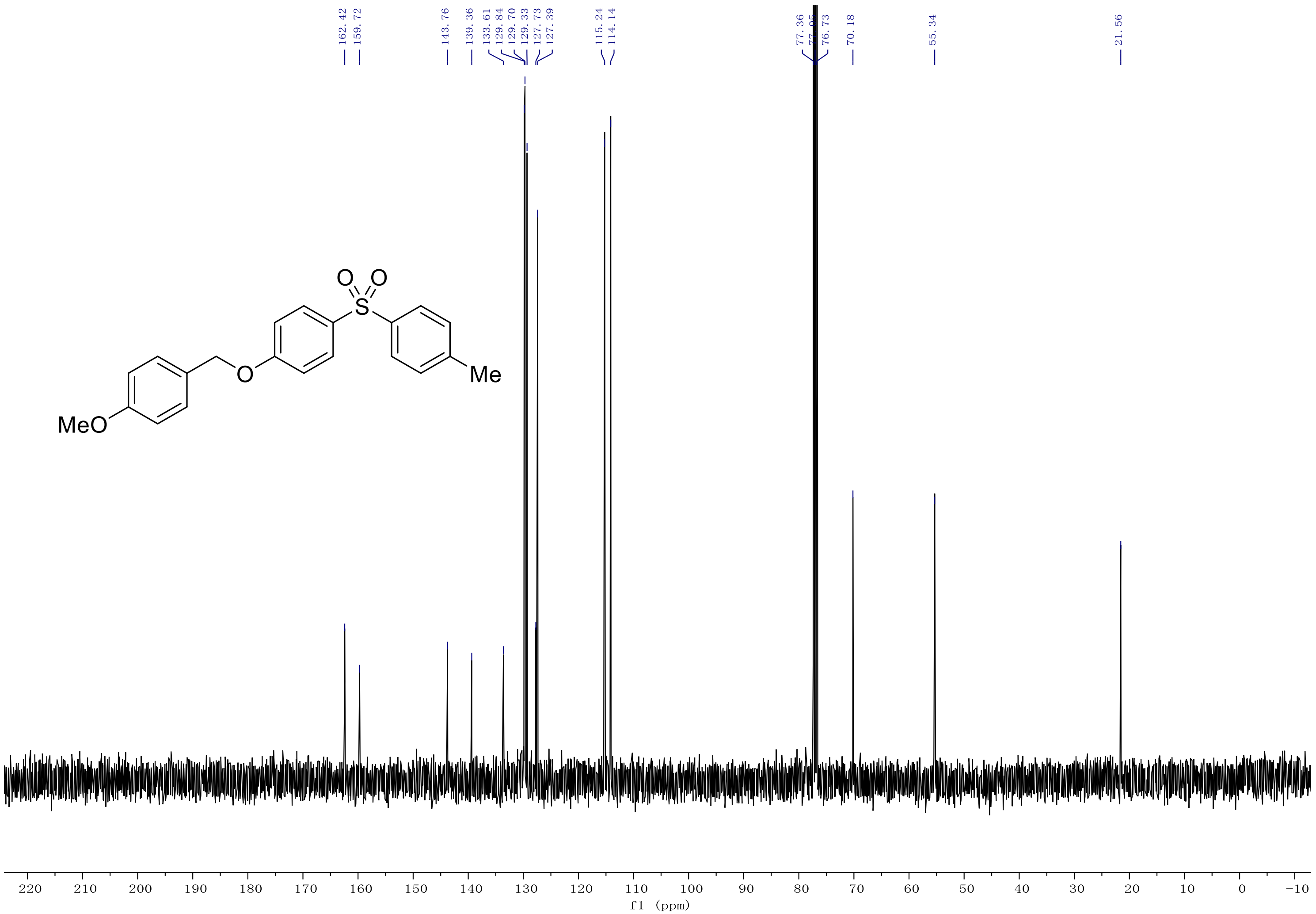

^{13}C NMR spectrum of **1-methoxy-4-((4-tosylphenoxy)methyl)benzene 4k**

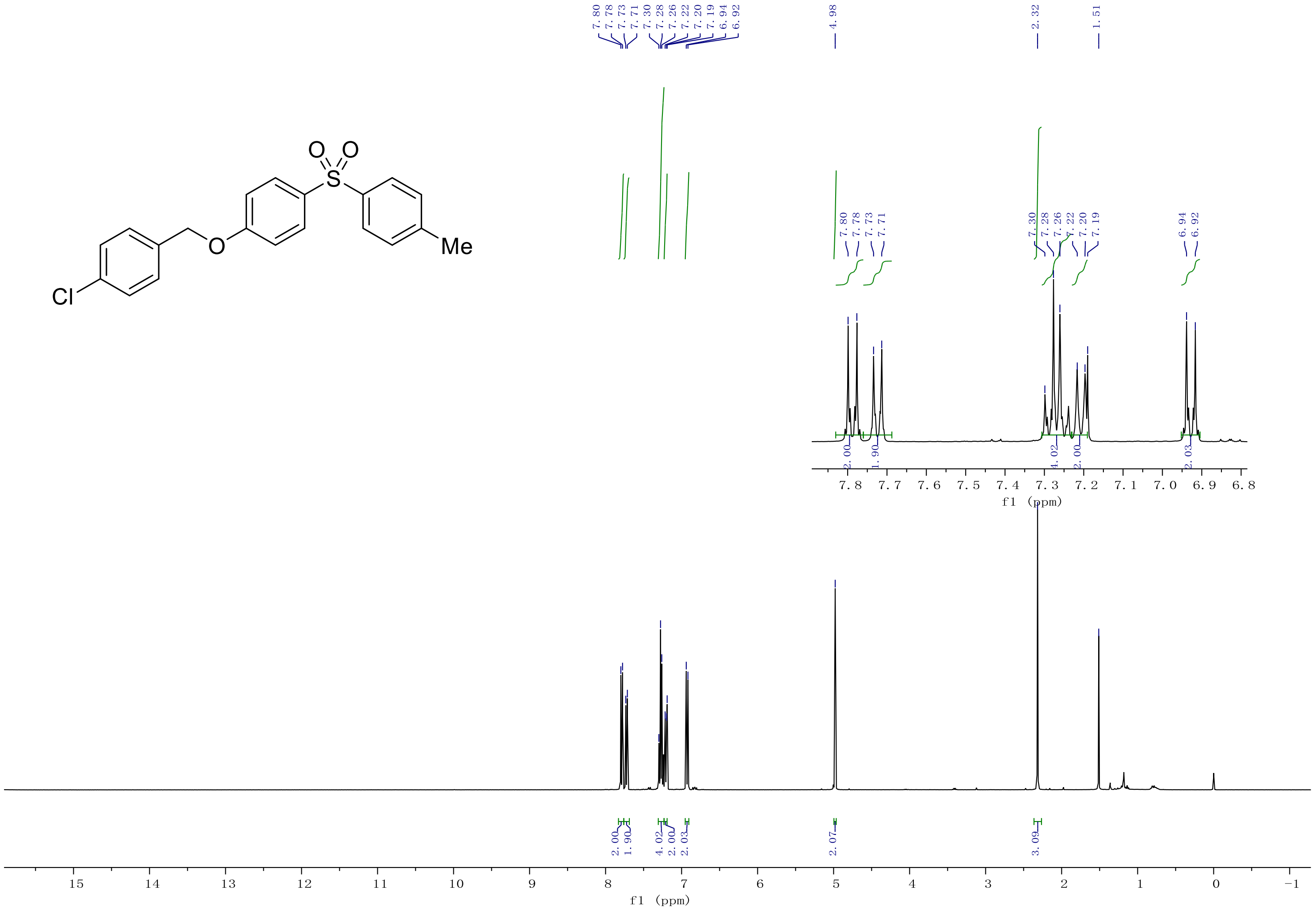

^{1}H NMR spectrum of **1-chloro-4-((4-tosylphenoxy)methyl)benzene 4l**

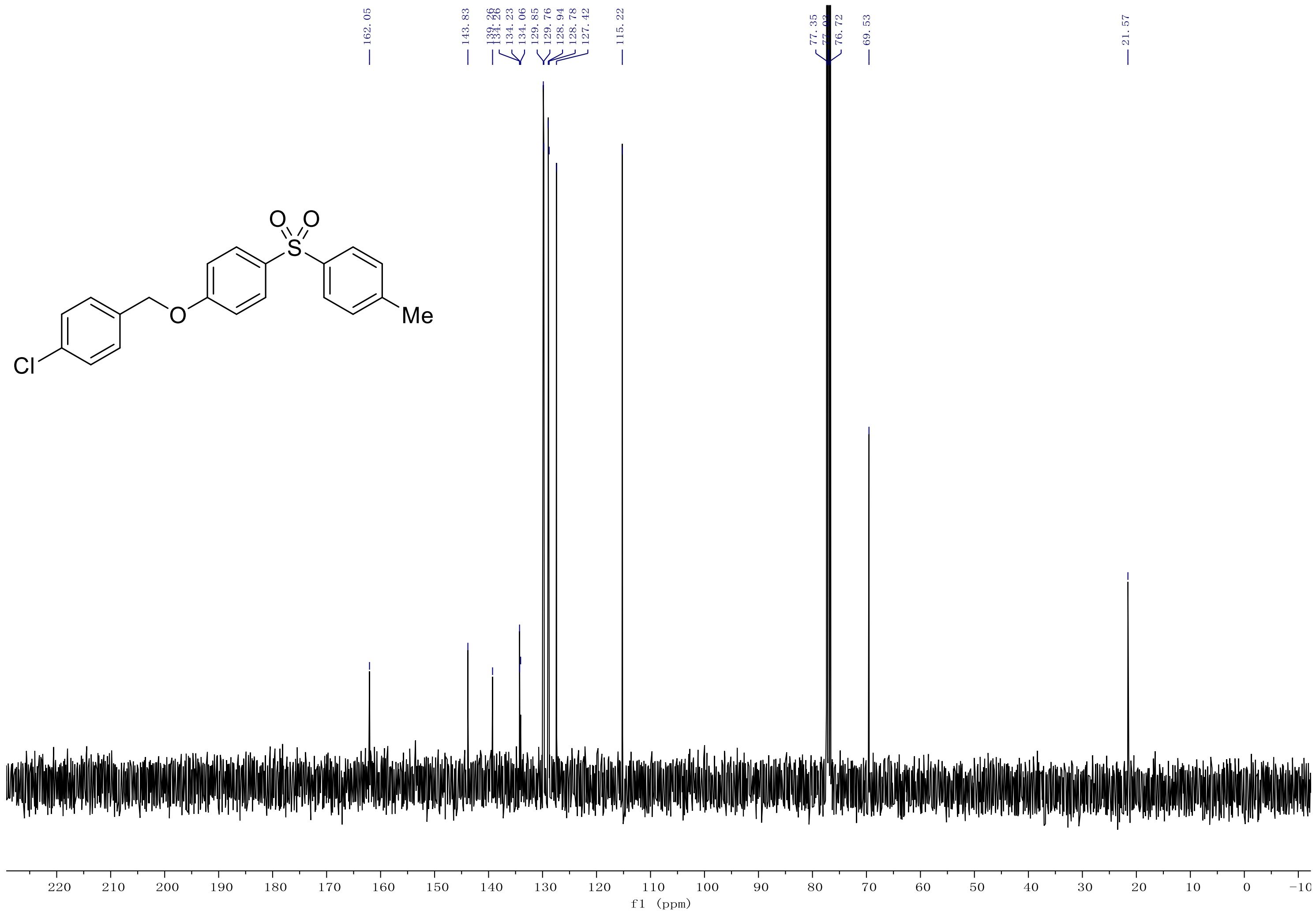

^{13}C NMR spectrum of **1-chloro-4-((4-tosylphenoxy)methyl)benzene 4l**

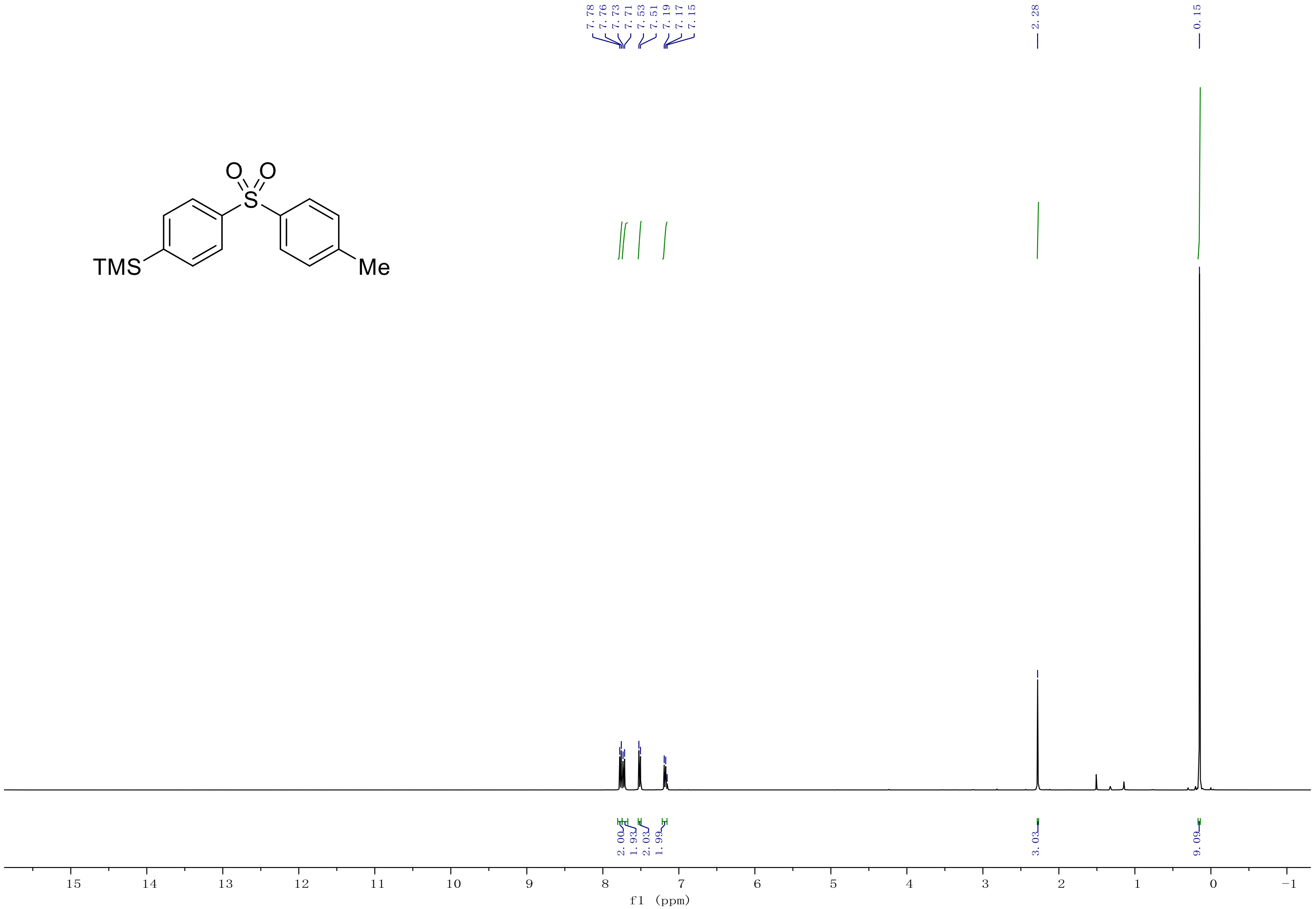

^{1}H NMR spectrum of **trimethyl(4-tosylphenyl)silane 4m**

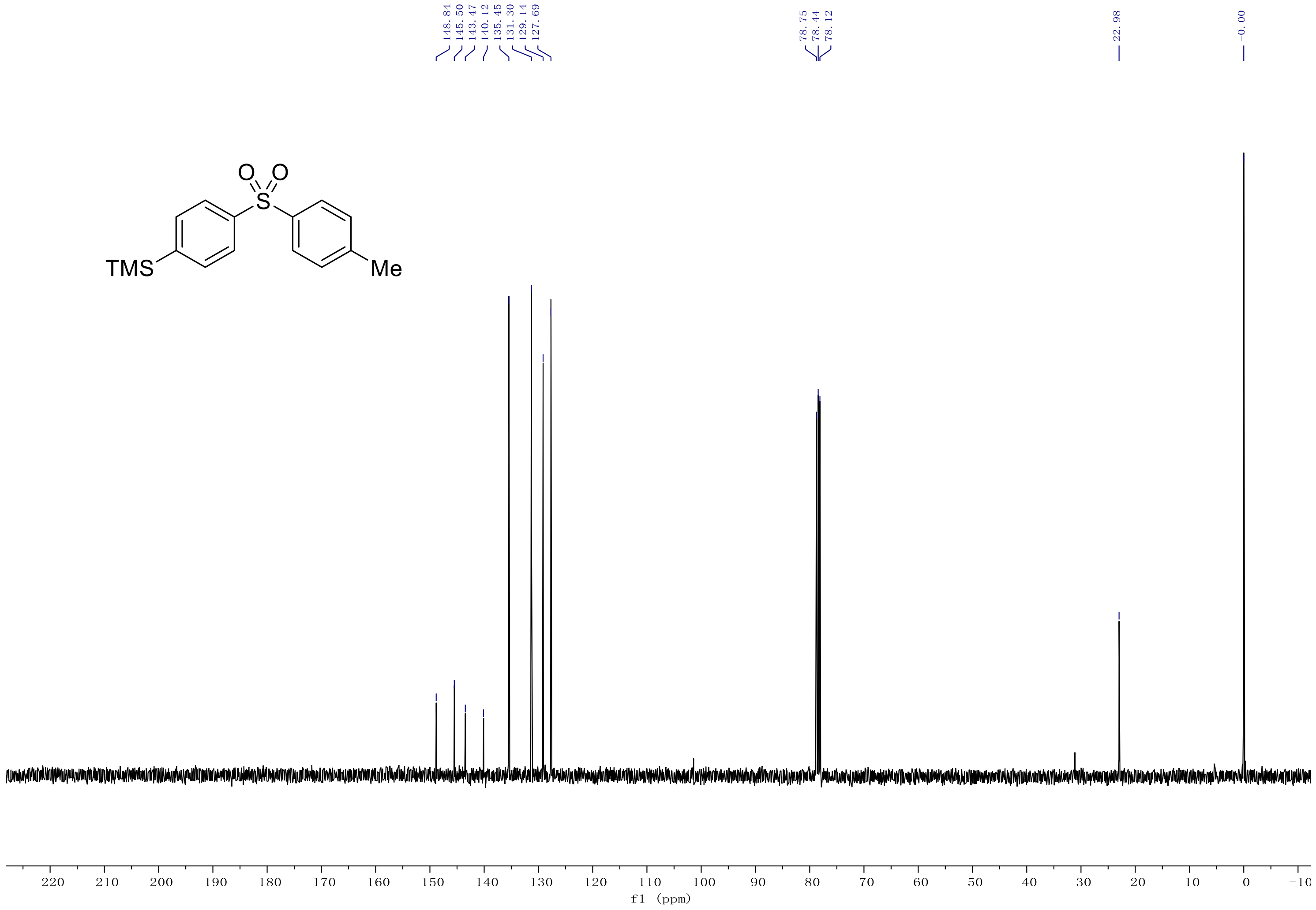

^{13}C NMR spectrum of **trimethyl(4-tosylphenyl)silane 4m**

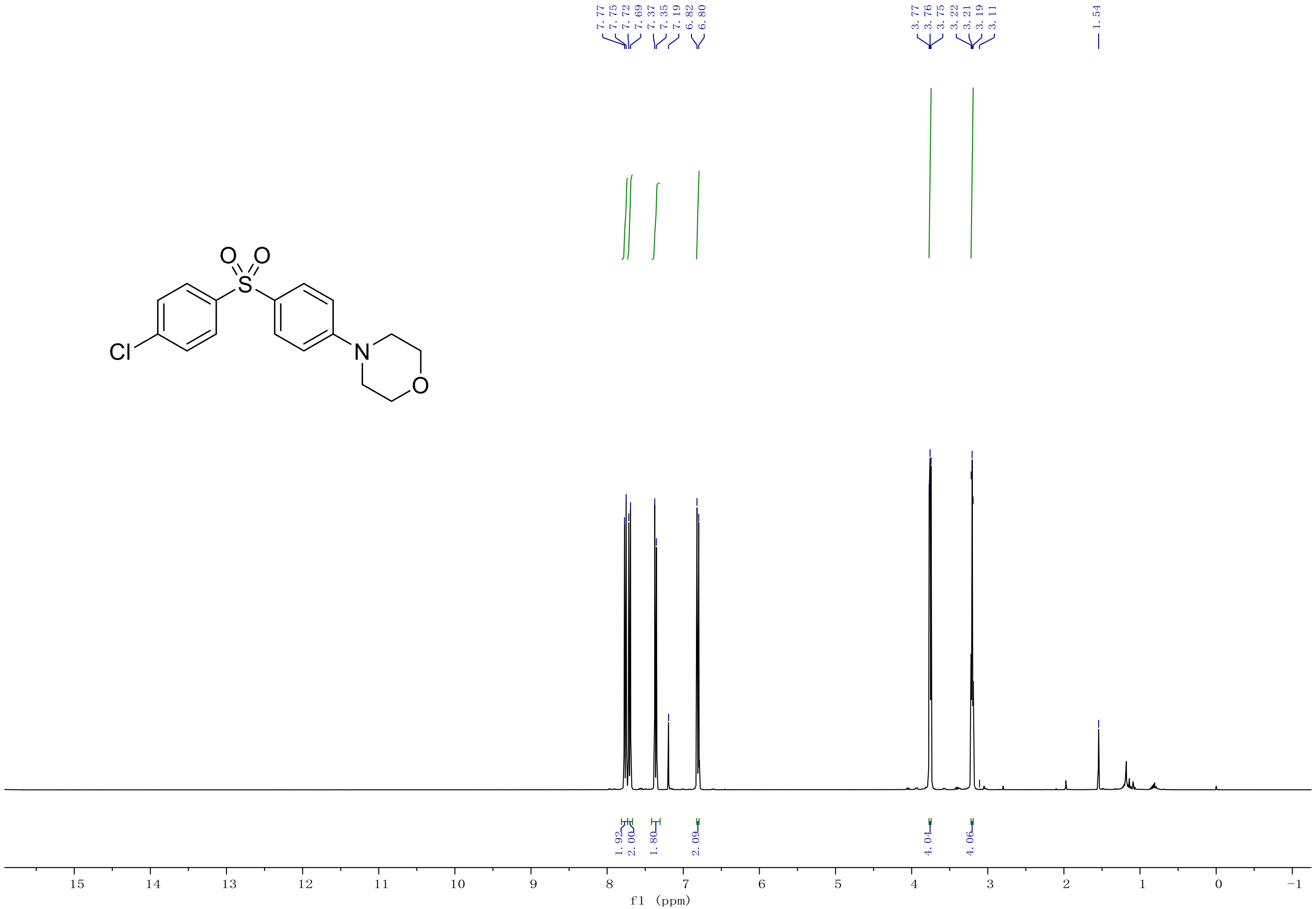

^{1}H NMR spectrum of **4-{4-[(4-chlorophenyl)sulfonyl]phenyl}morpholine 4n**

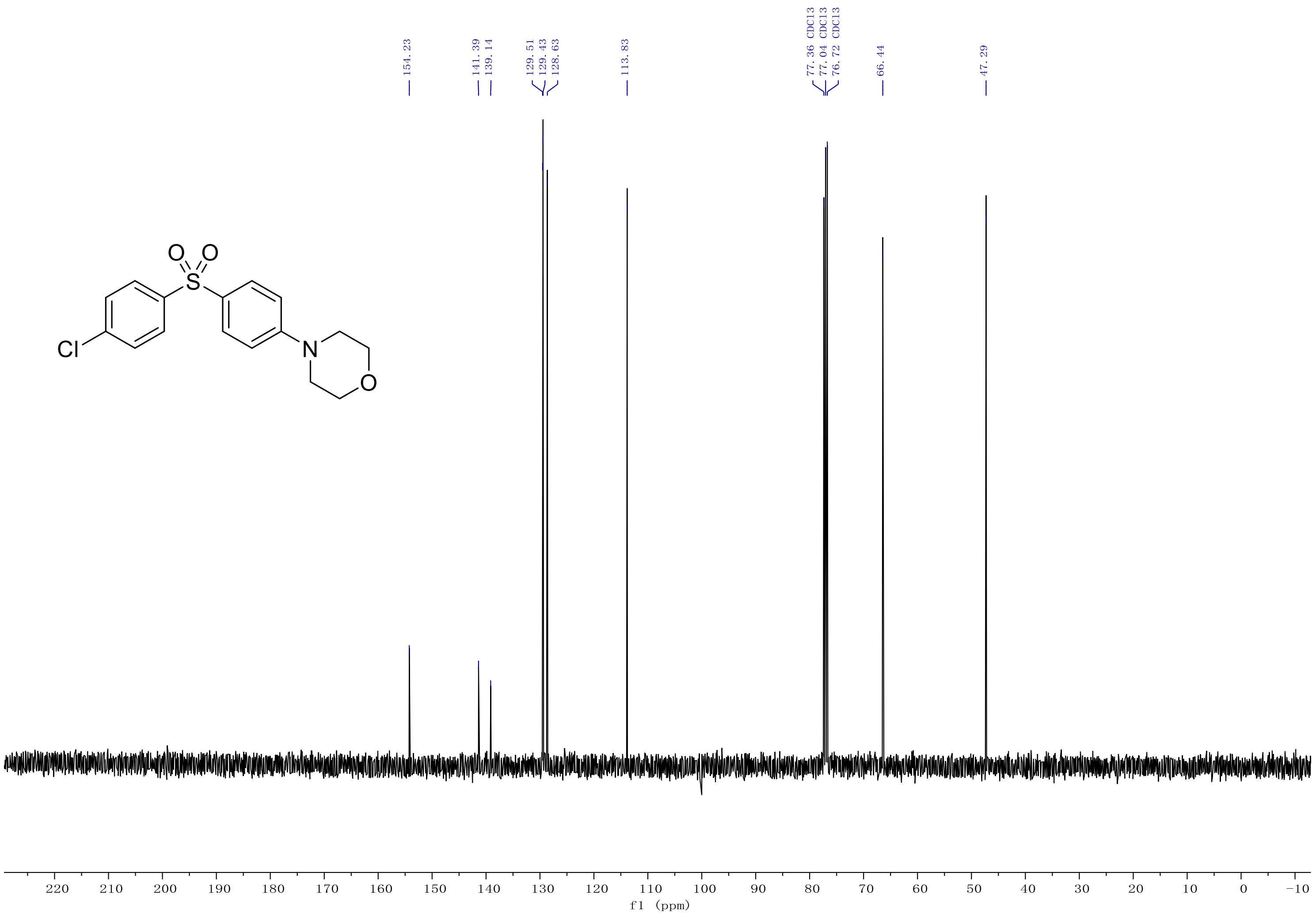

^{13}C NMR spectrum of **4-{4-[(4-chlorophenyl)sulfonyl]phenyl}morpholine 4n**

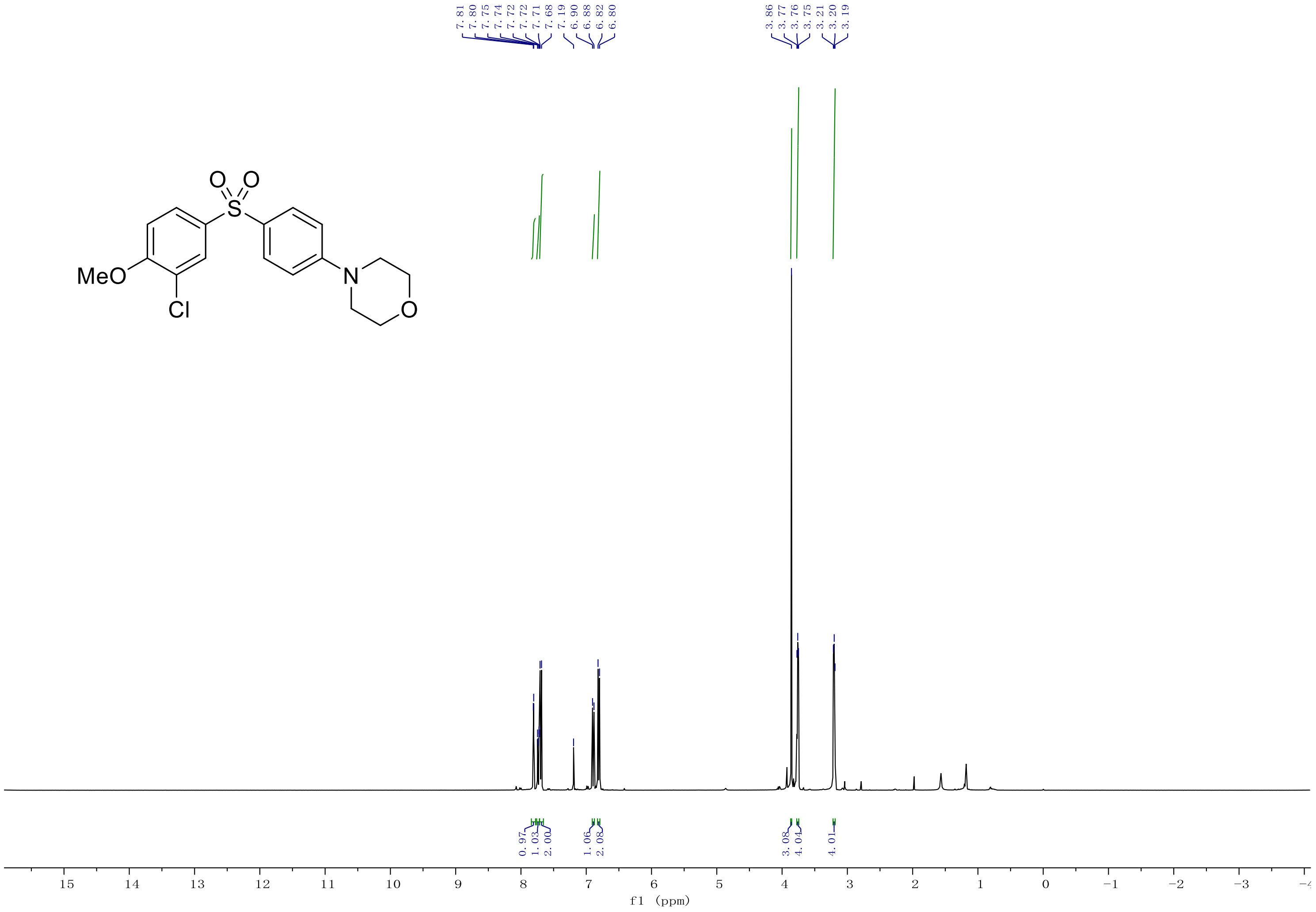

^{1}H NMR spectrum of **4-(4-((3-chloro-4-methoxyphenyl)sulfonyl)phenyl)morpholine 4o**

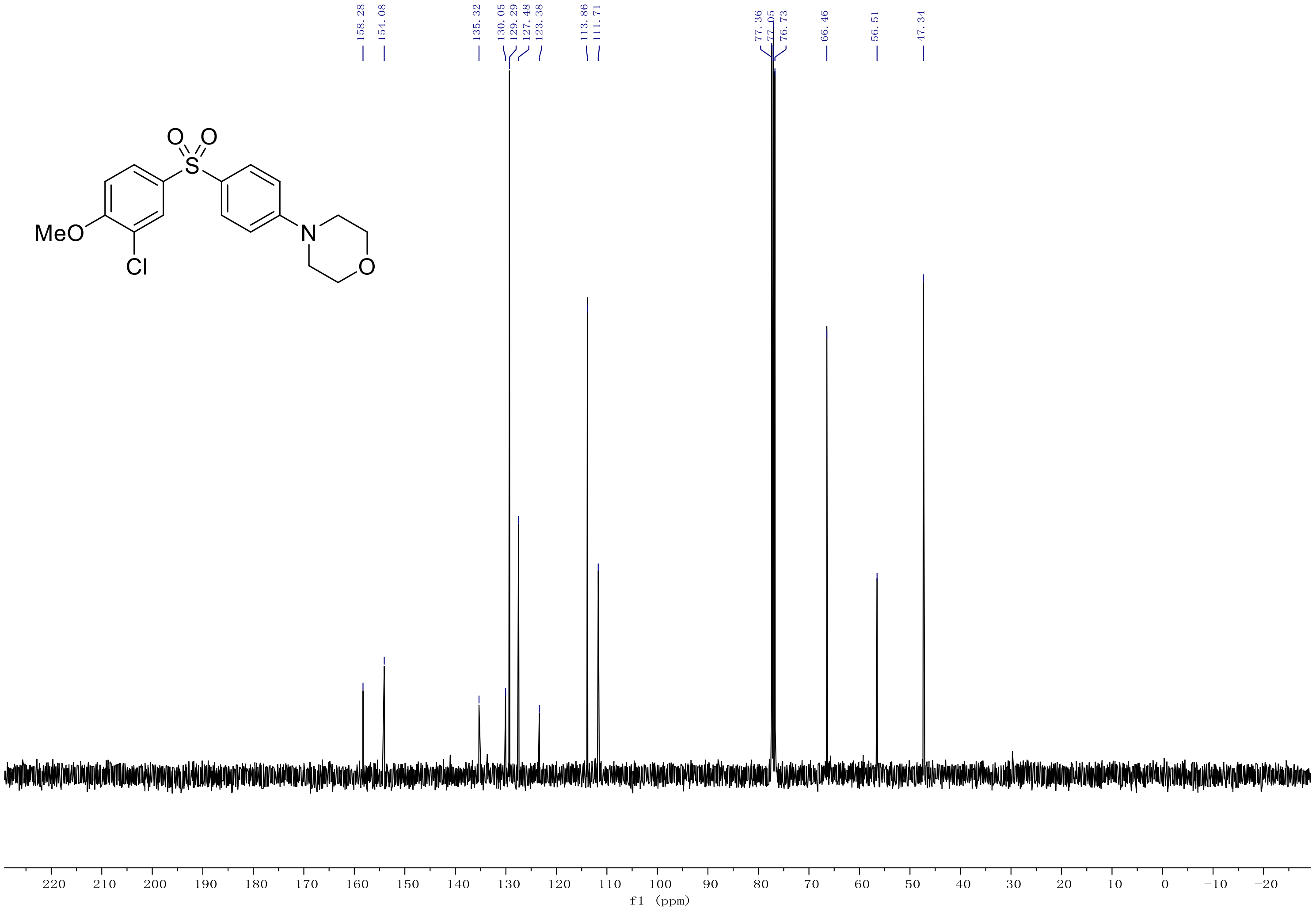

^{13}C NMR spectra of **4-(4-((3-chloro-4-methoxyphenyl)sulfonyl)phenyl)morpholine 4o**

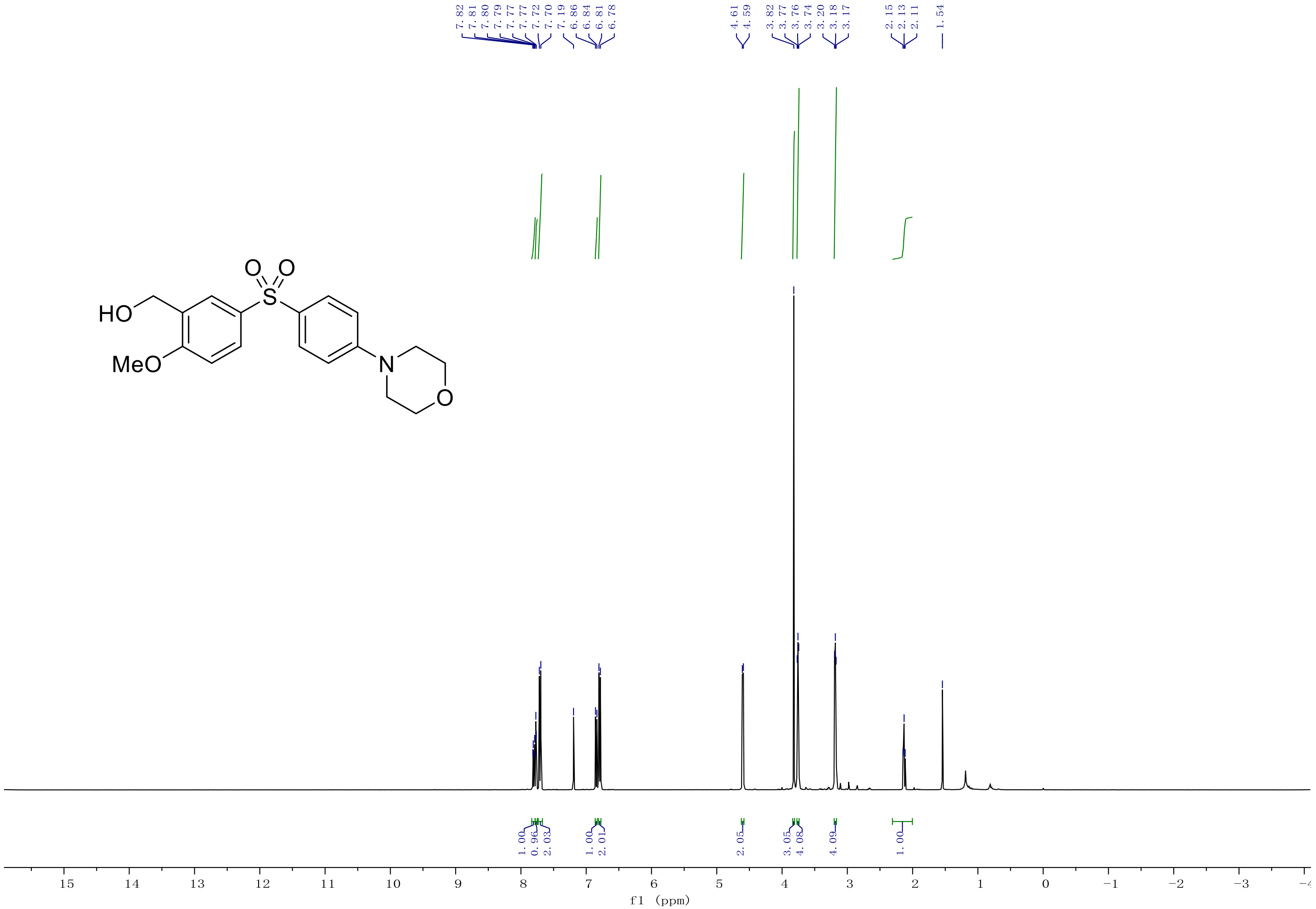

^{1}H NMR and ^{13}C NMR spectra of **{2-Methoxy-5-[(4-morpholinophenyl)sulfonyl]phenyl}methanol 4p**

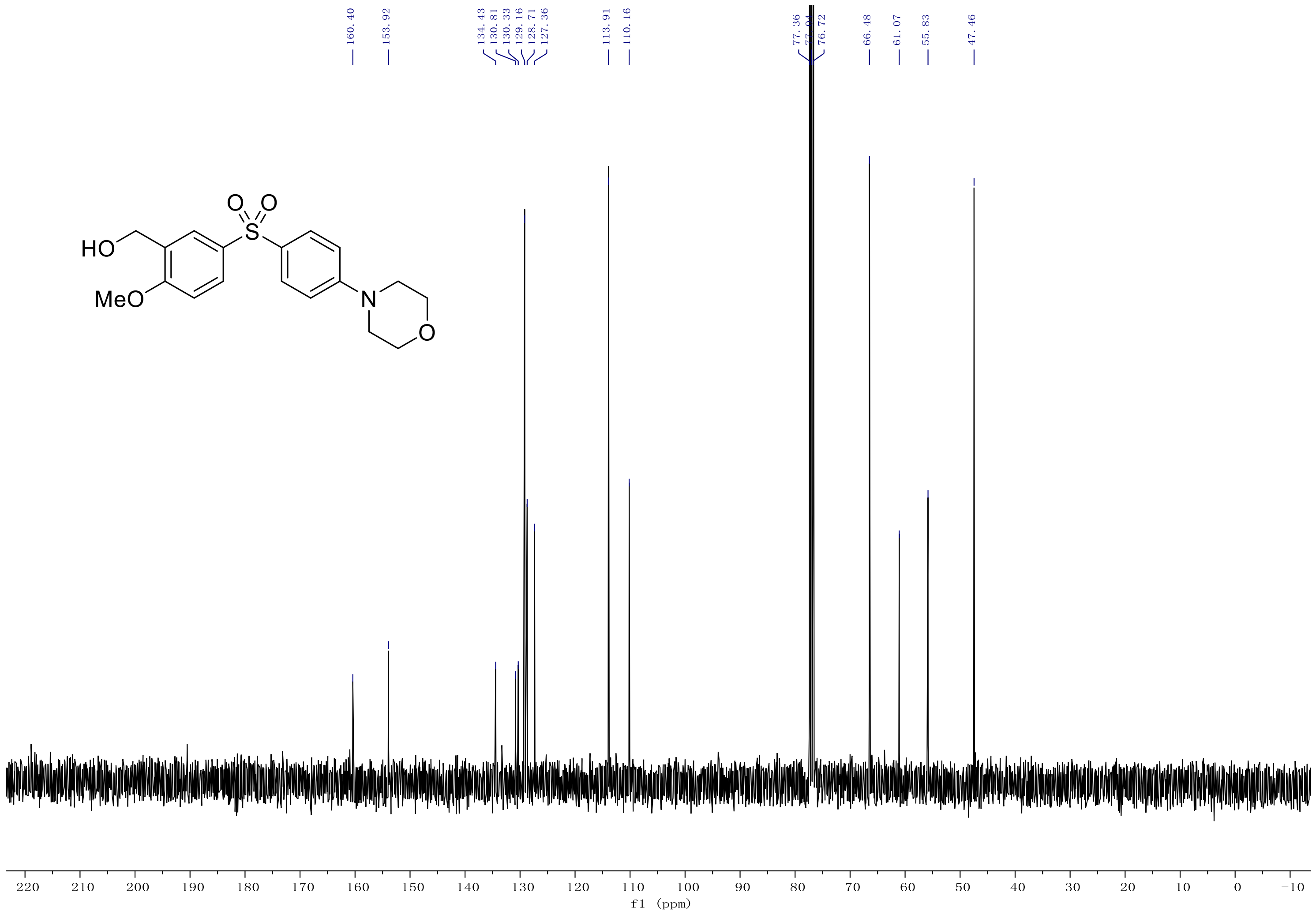

^{1}H NMR and ^{13}C NMR spectra of **{2-Methoxy-5-[(4-morpholinophenyl)sulfonyl]phenyl}methanol 4p**

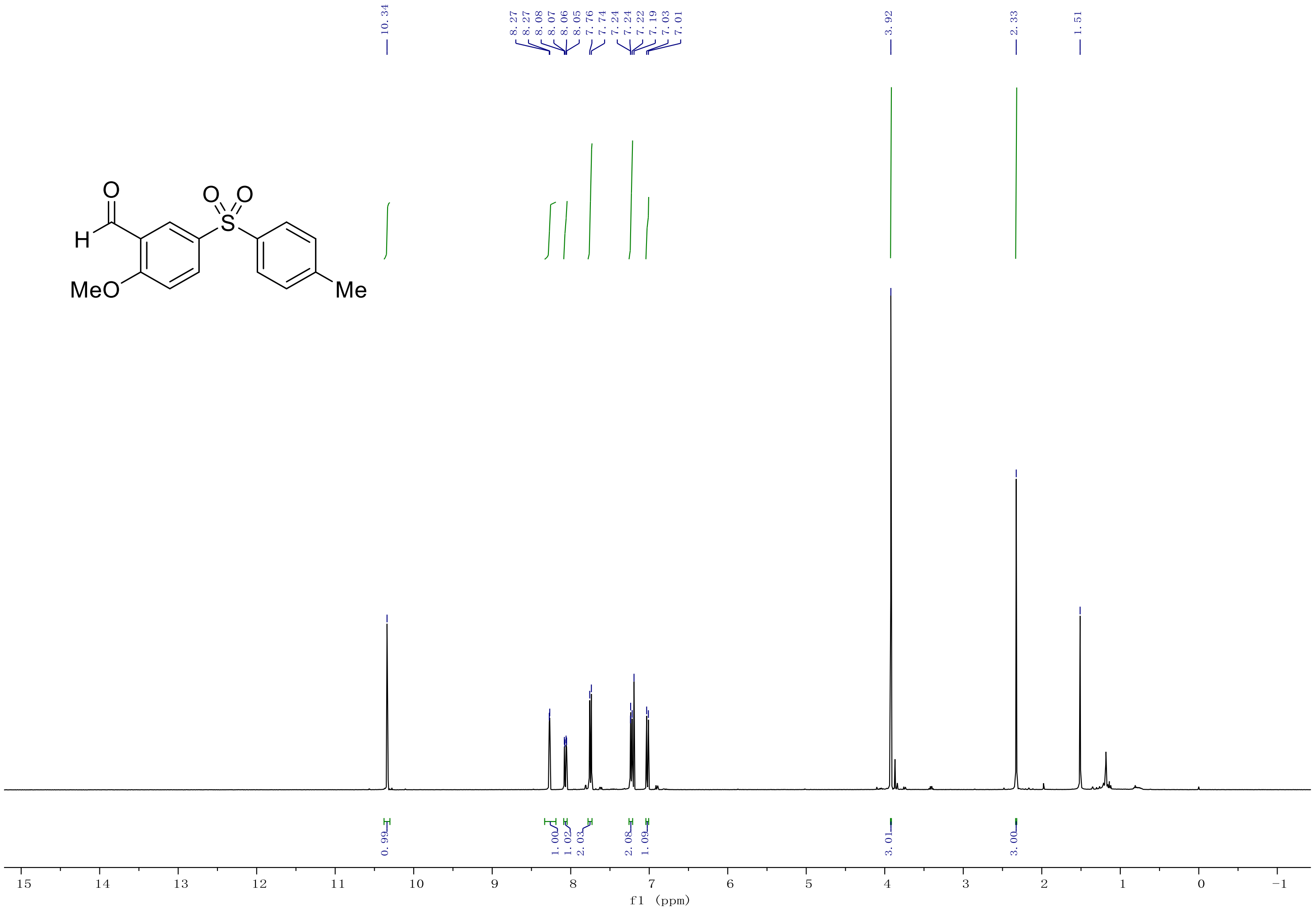

^{1}H NMR spectrum of **2-methoxy-5-tosylbenzaldehyde 4q**

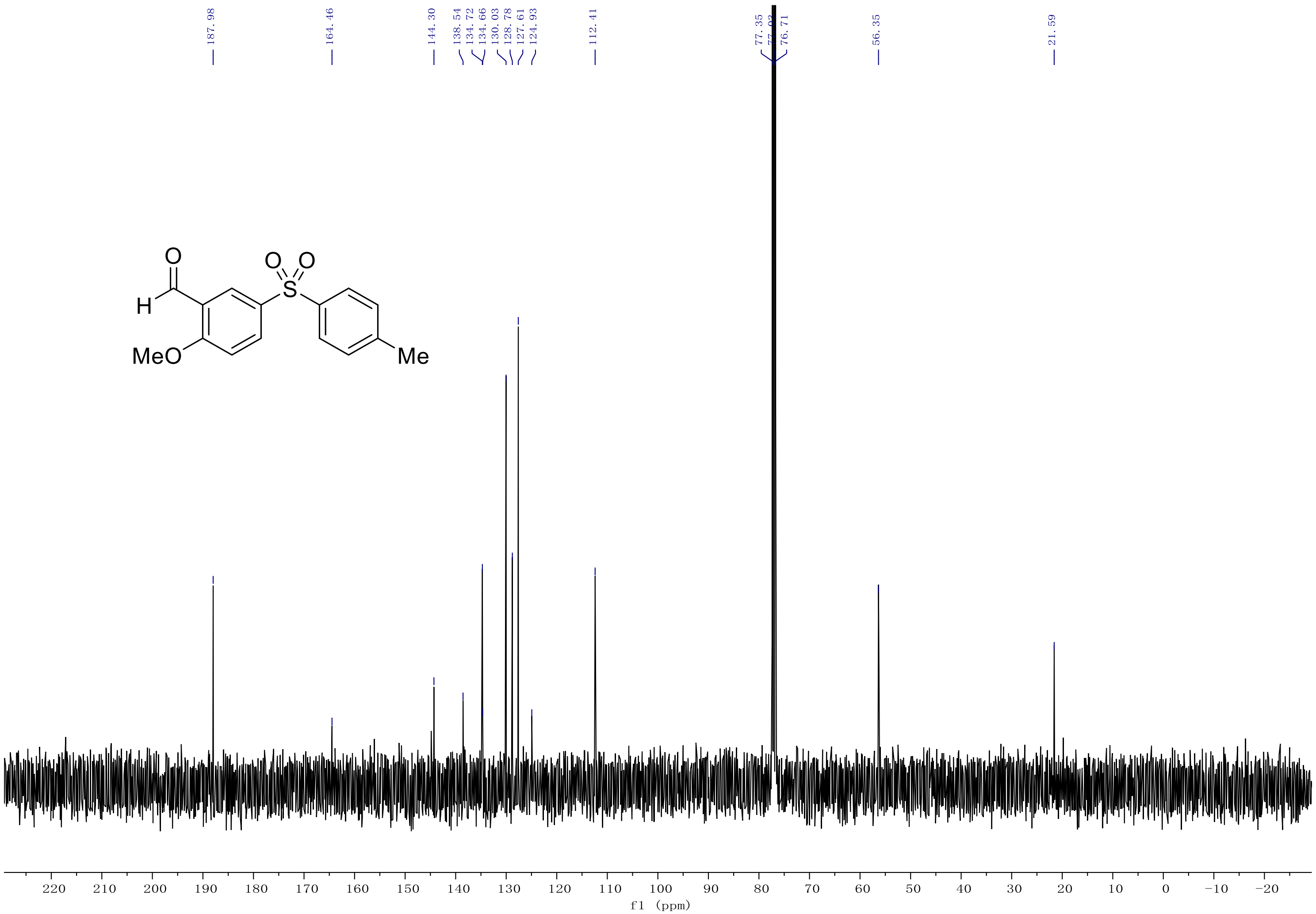

^{13}C NMR spectrum of **2-methoxy-5-tosylbenzaldehyde 4q**

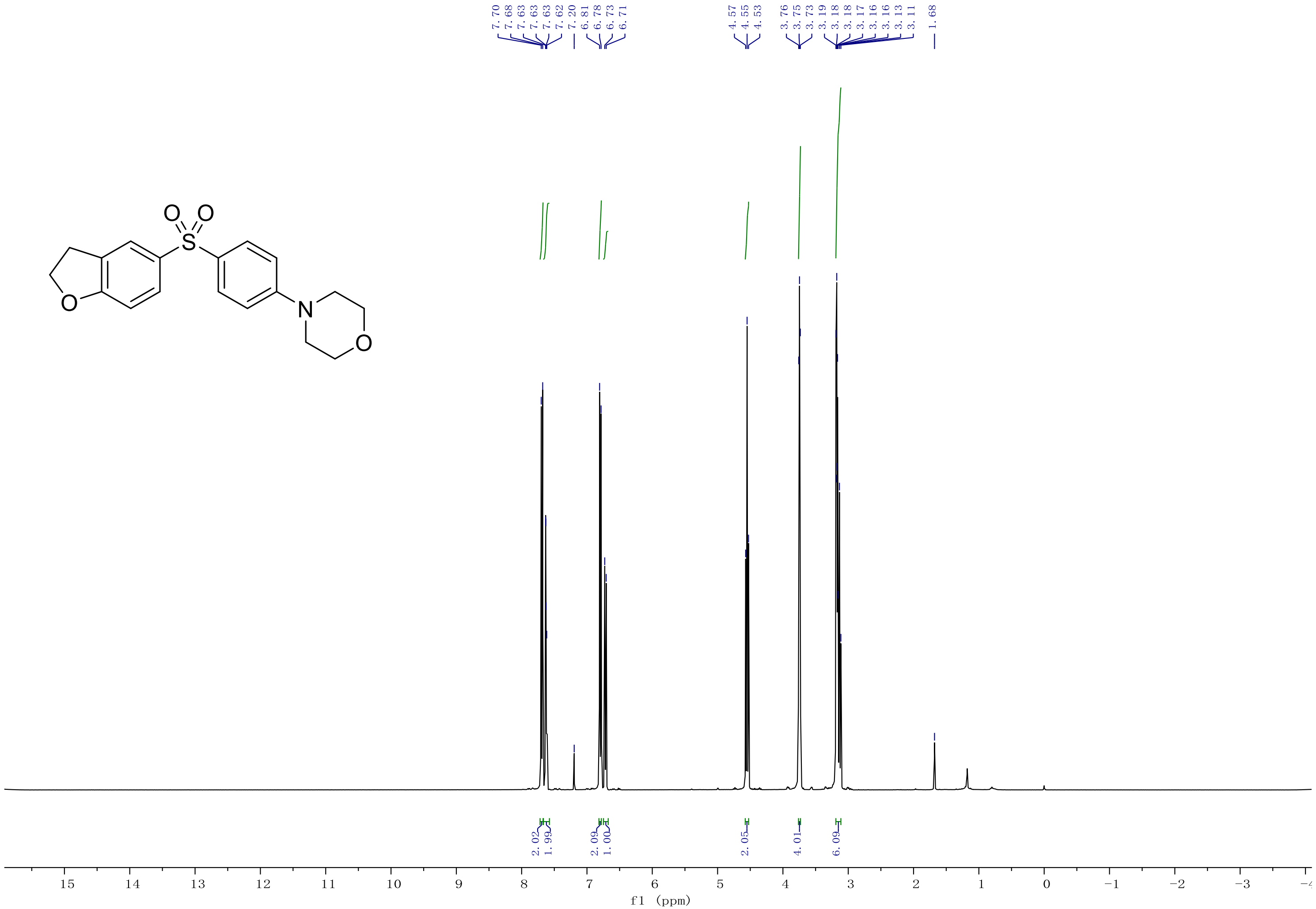

^{1}H NMR spectrum of **4-{4-[(2,3-Dihydrobenzofuran-5-yl)sulfonyl]phenyl}morpholine 4r**

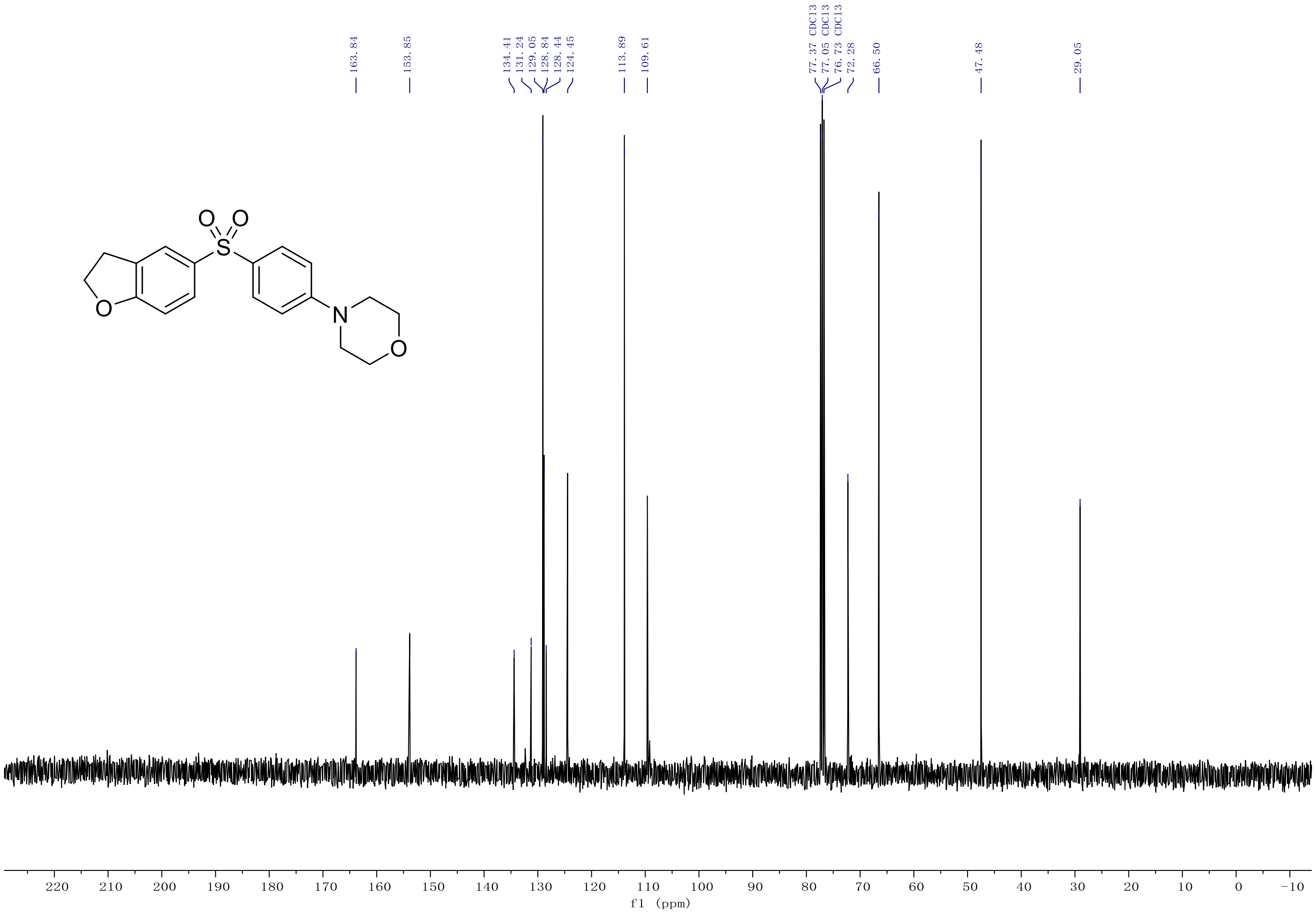

^{1}H NMR and ^{13}C NMR spectra of **4-{4-[(2,3-Dihydrobenzofuran-5-yl)sulfonyl]phenyl}morpholine 4r**

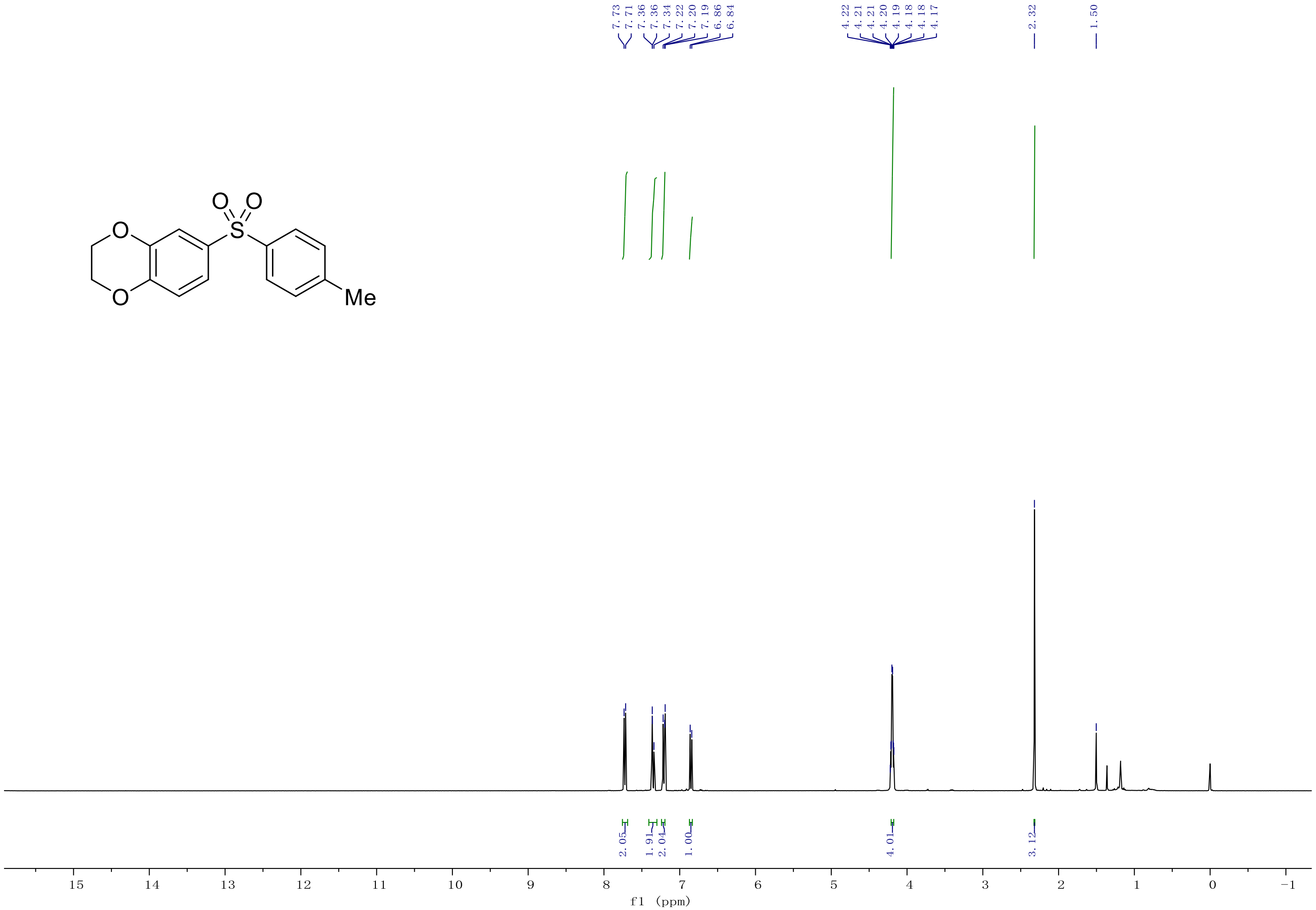

^{1}H NMR spectrum of **6-tosyl-2,3-dihydrobenzo-1,4-dioxine 4s**

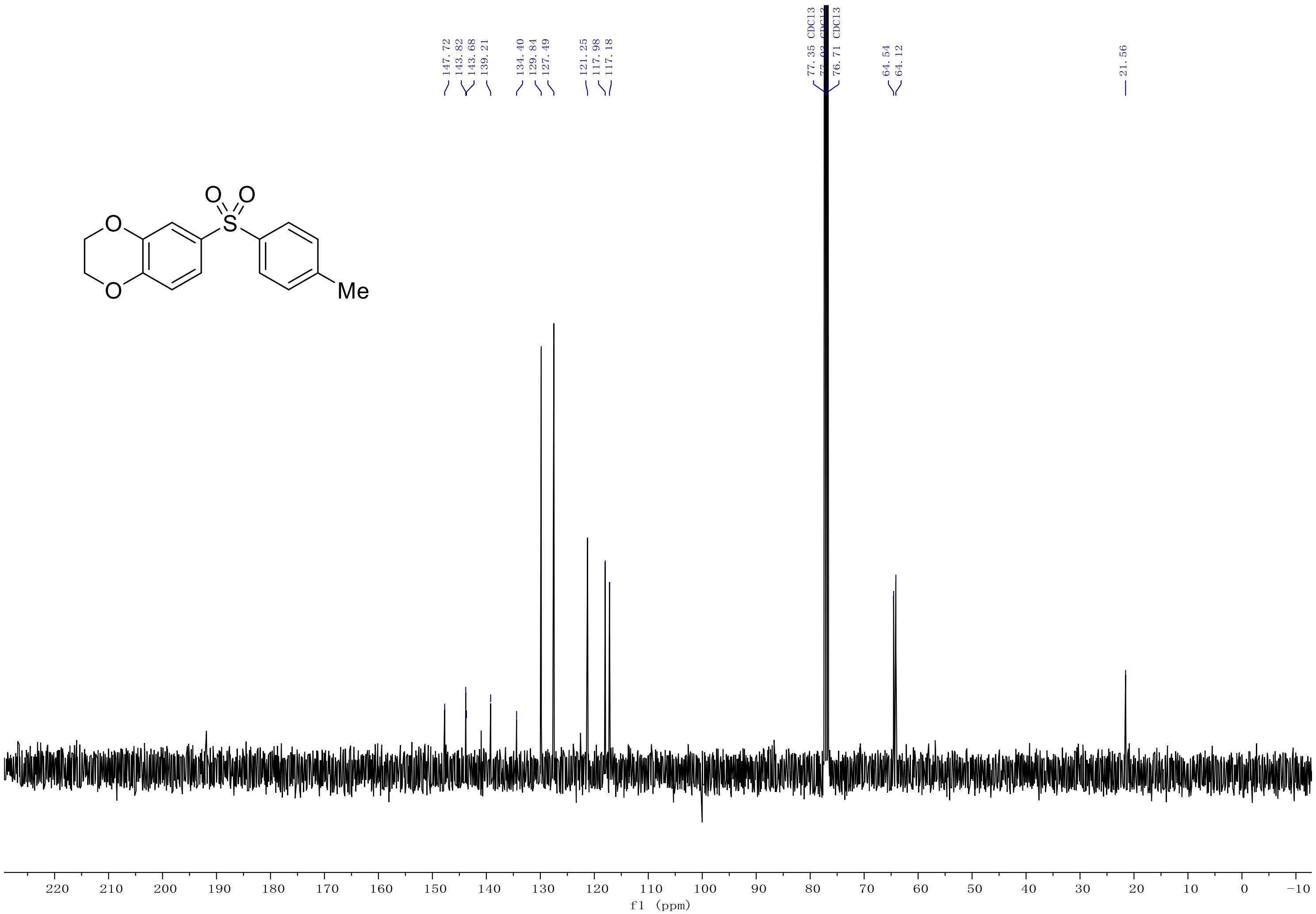

^{13}C NMR spectrum of **6-tosyl-2,3-dihydrobenzo-1,4-dioxine 4s**

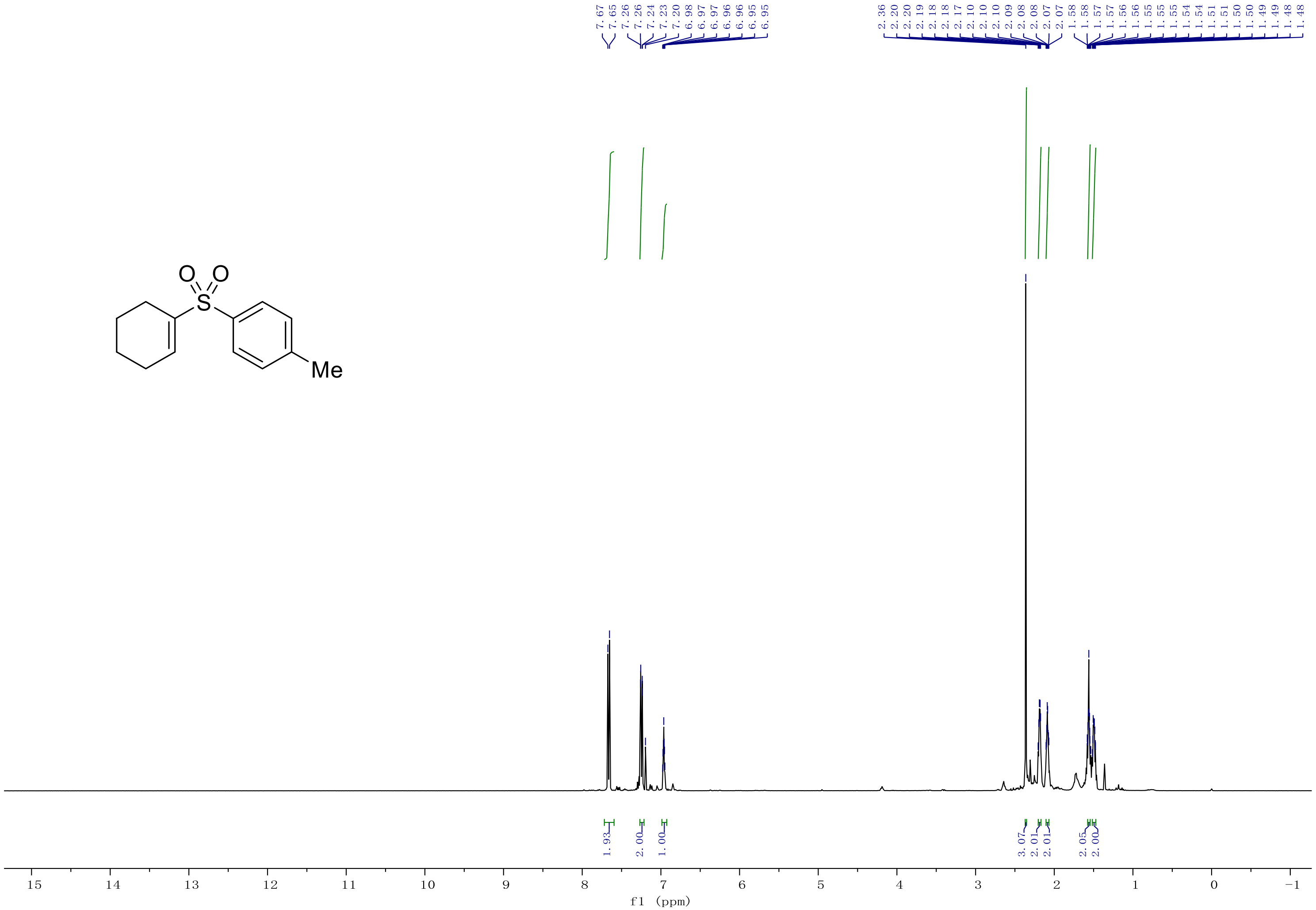

^{1}H NMR and ^{13}C NMR spectra of **1-(cyclohex-1-en-1-ylsulfonyl)-4-methylbenzene 4t**

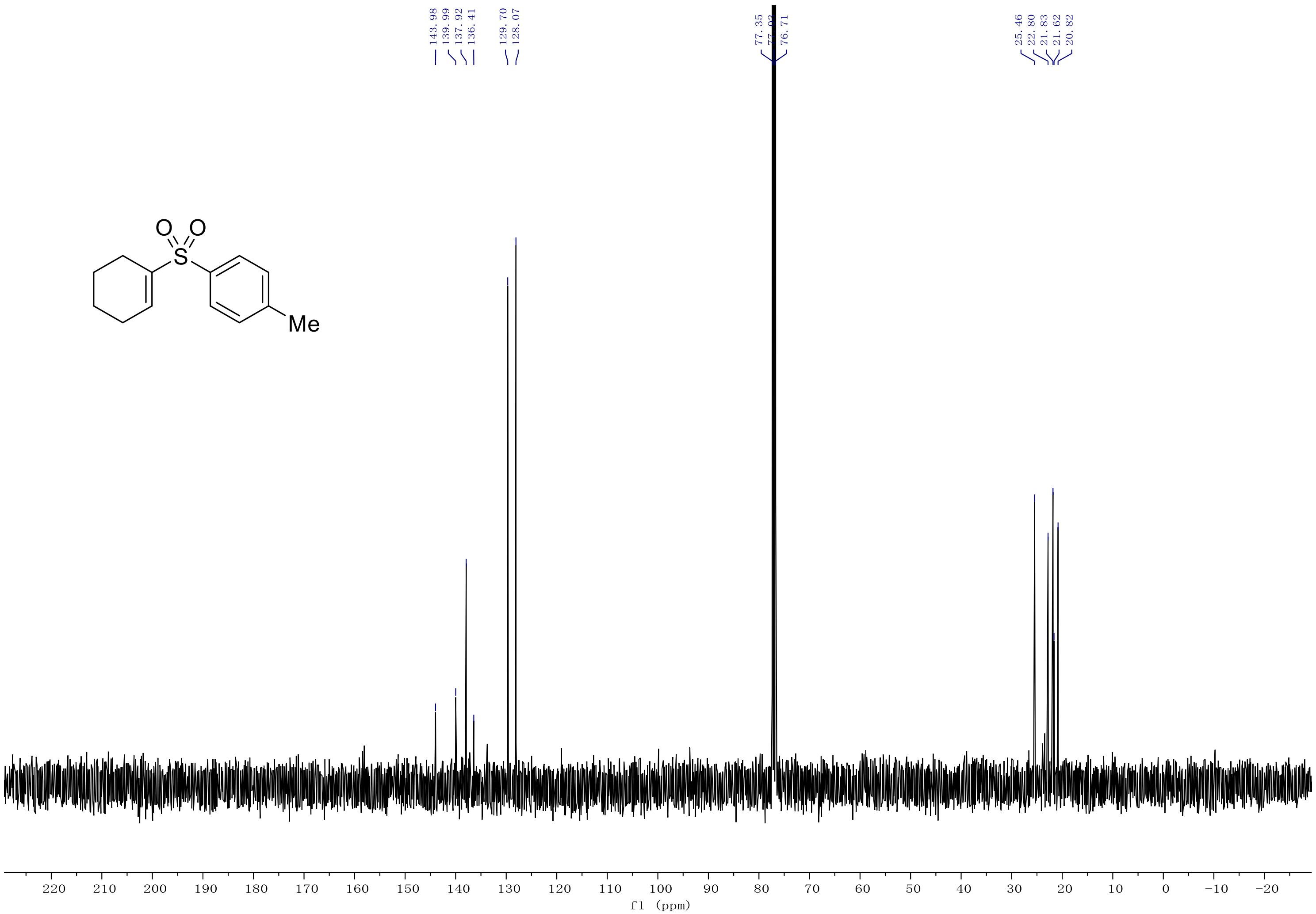

^{1}H NMR and ^{13}C NMR spectra of **1-(cyclohex-1-en-1-ylsulfonyl)-4-methylbenzene 4t**

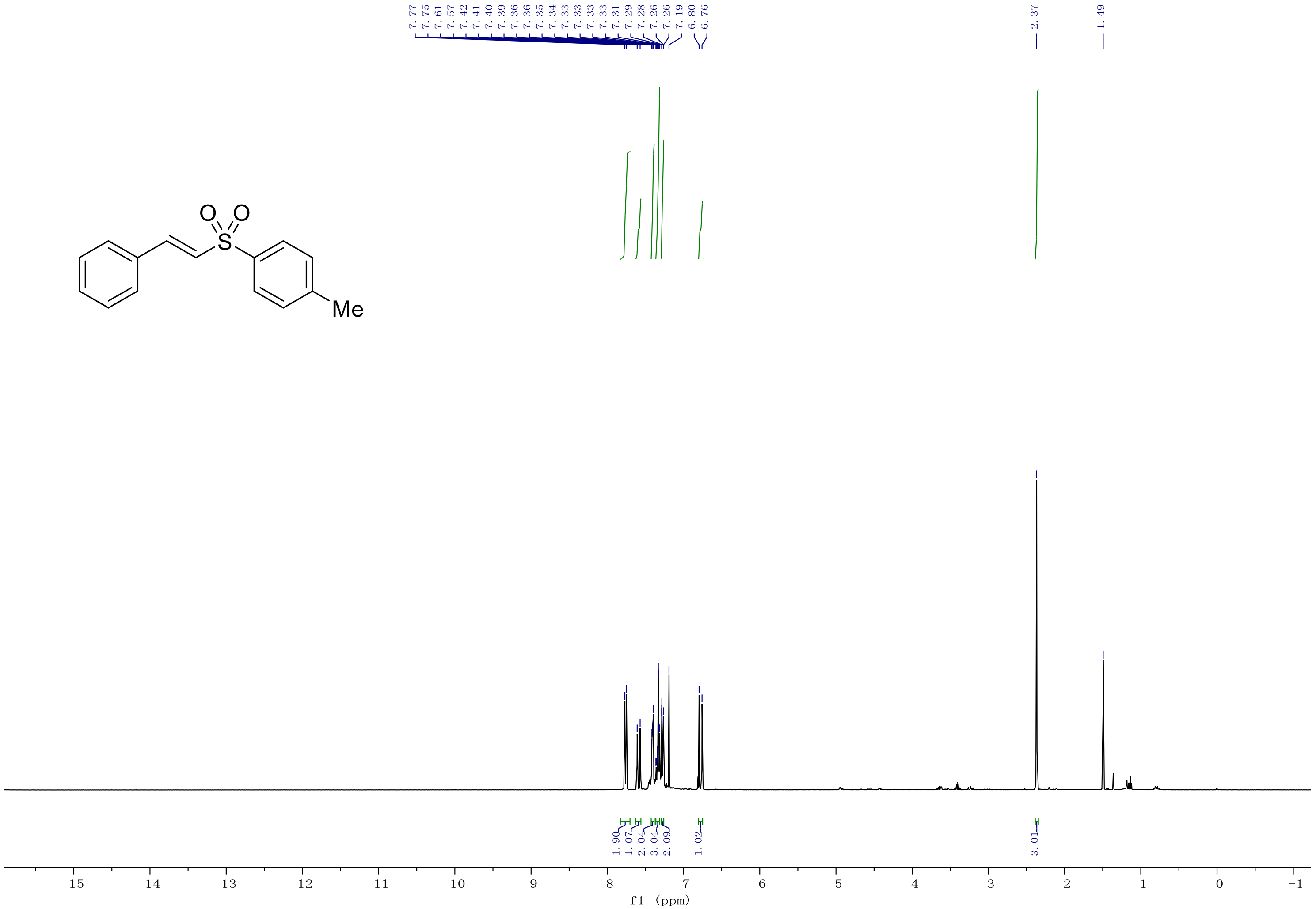

^{1}H NMR spectrum of ***tans*-1-methyl-4-(styrylsulfonyl)benzene 4u**

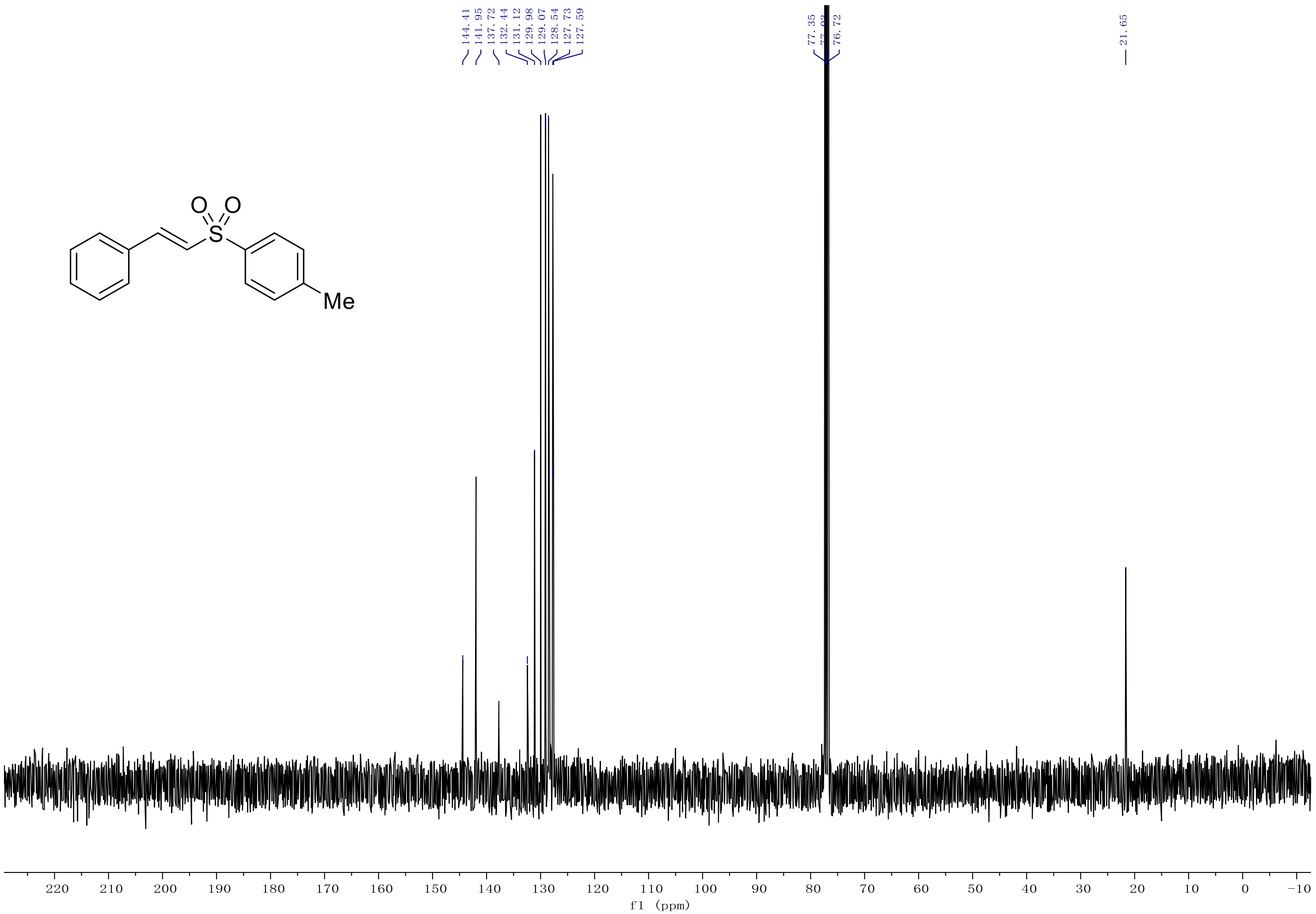

^{13}C NMR spectrum of ***tans*-1-methyl-4-(styrylsulfonyl)benzene 4u**

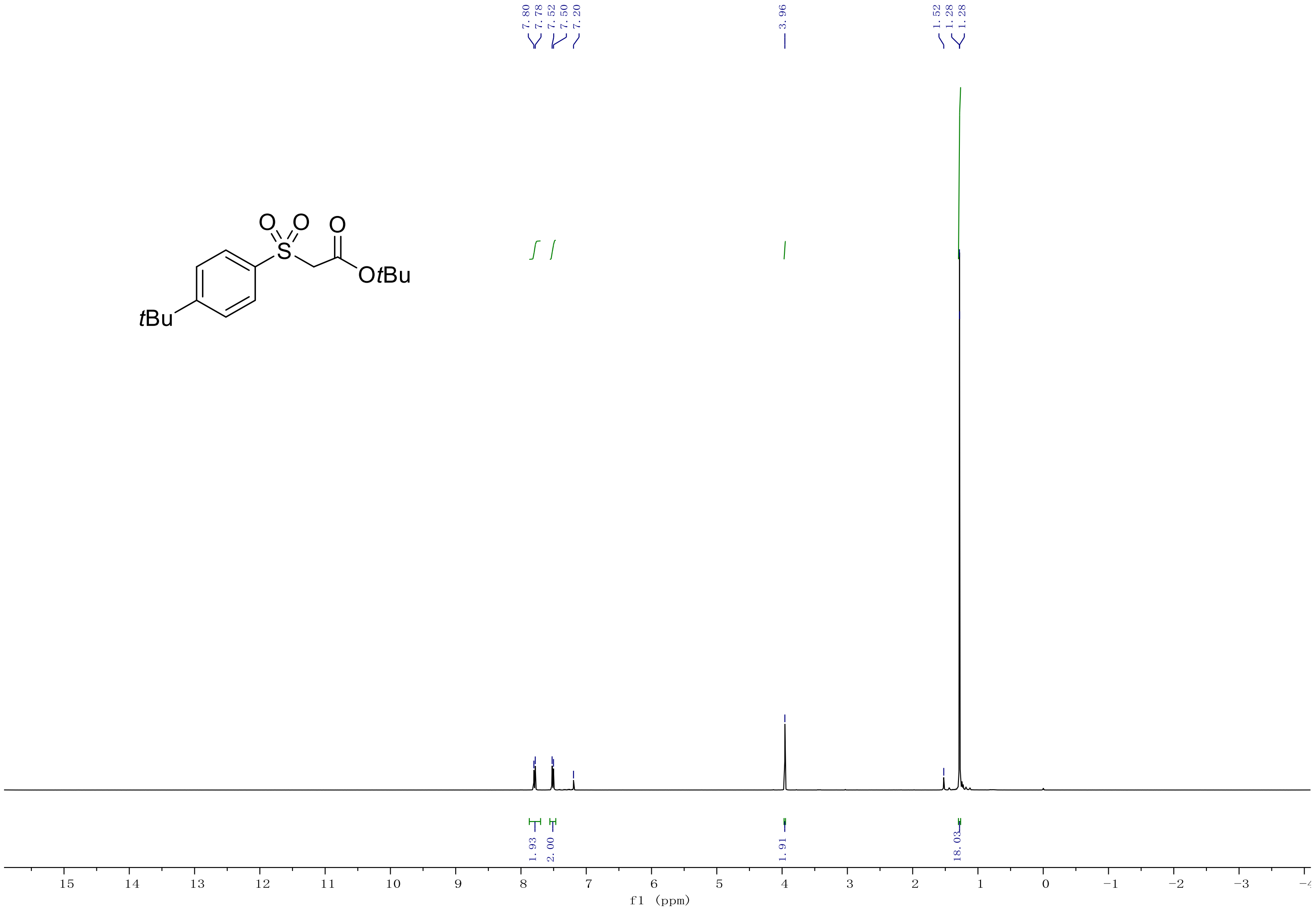

^{1}H NMR spectrum of **4-*tert*-butyl 2-{[4-(*tert*-butyl)phenyl]sulfonyl}acetate 6a**

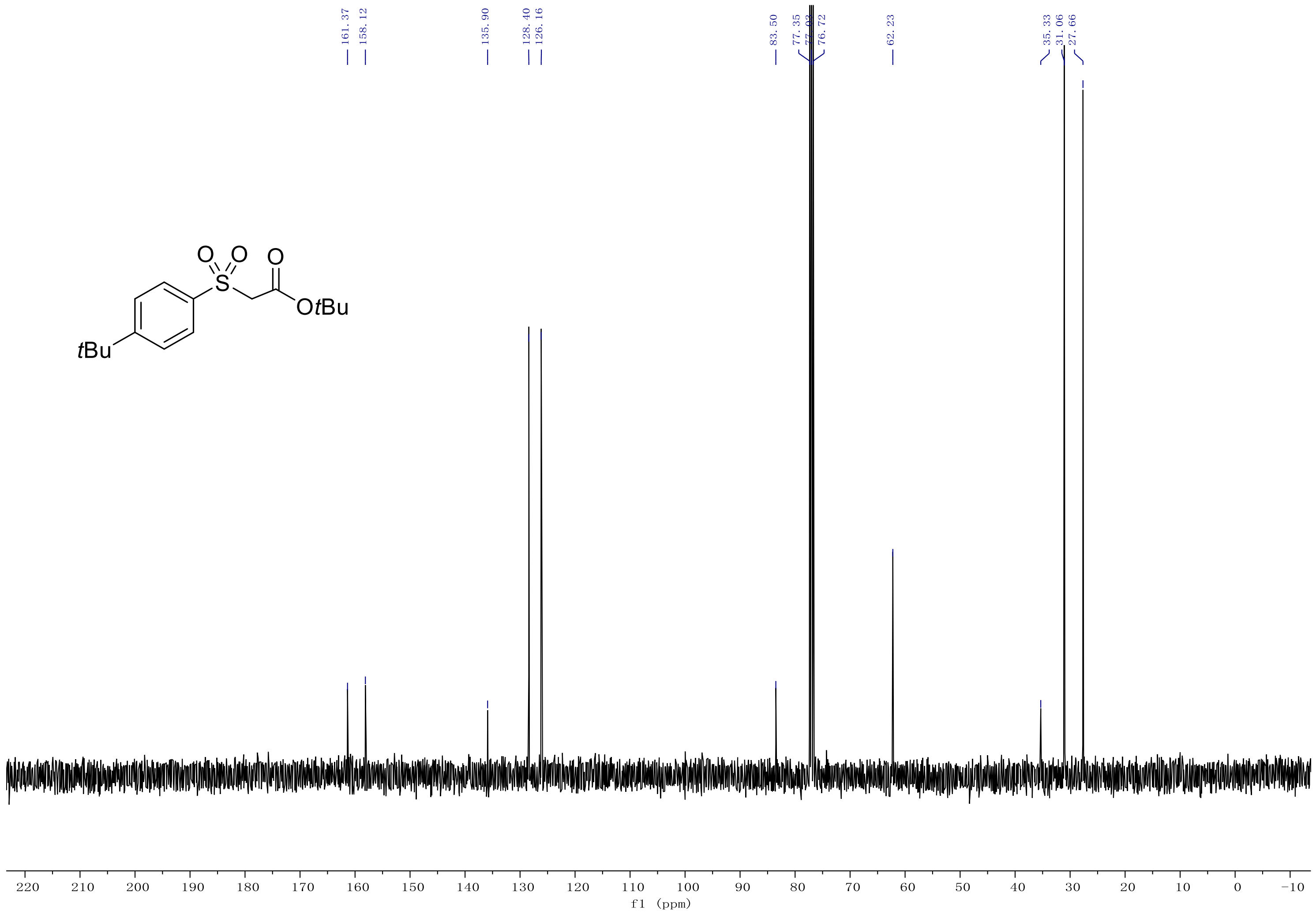

^{13}C NMR spectrum of **4-*tert*-butyl 2-{[4-(*tert*-butyl)phenyl]sulfonyl}acetate 6a**

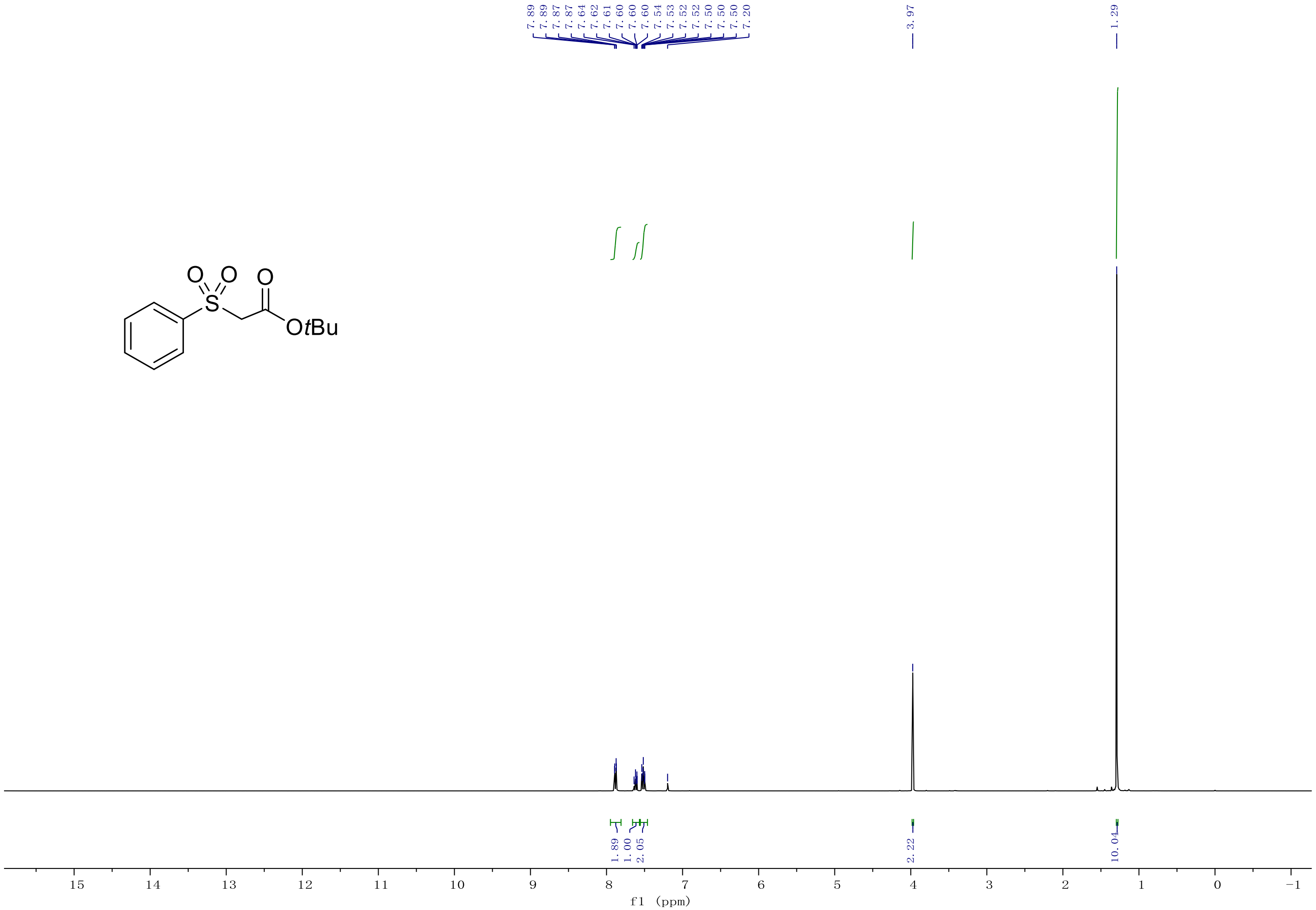

^{1}H NMR spectrum of ***tert*-butyl 2-(phenylsulfonyl)acetate 6b**

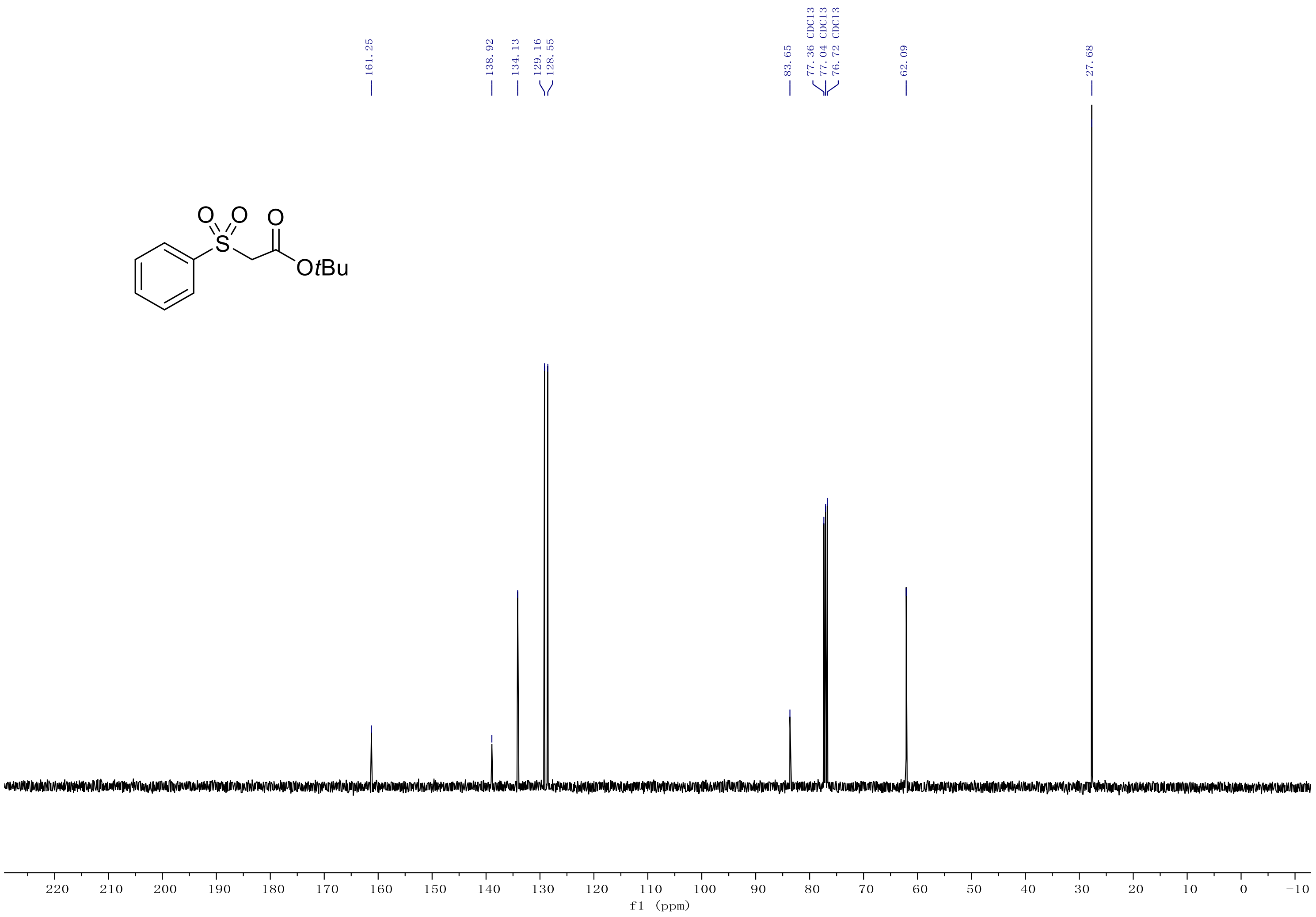

^{13}C NMR spectrum of ***tert*-butyl 2-(phenylsulfonyl)acetate 6b**

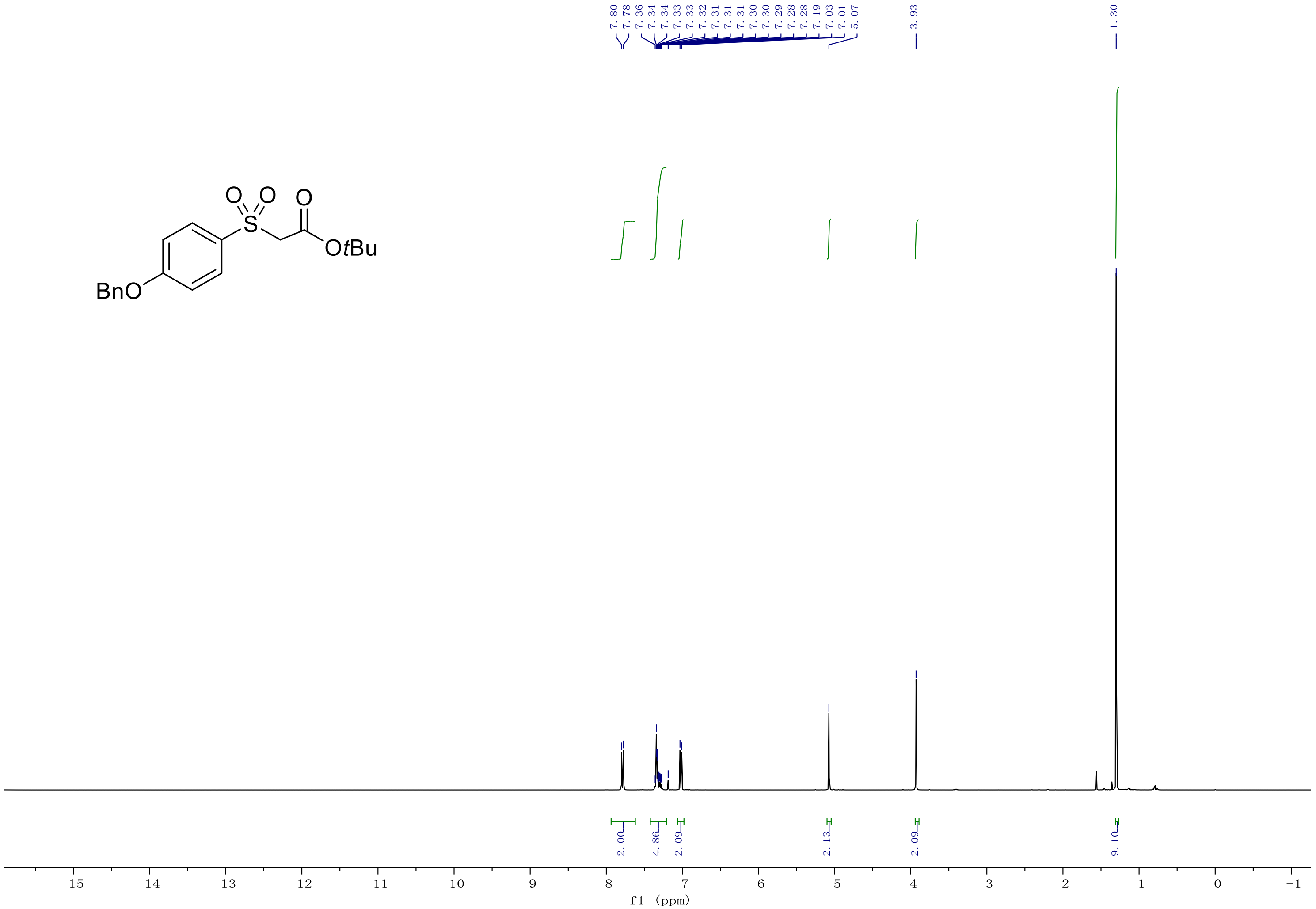

^{1}H NMR spectrum of ***tert*-butyl 2-((4-(benzyloxy)phenyl)sulfonyl)acetate 6c**

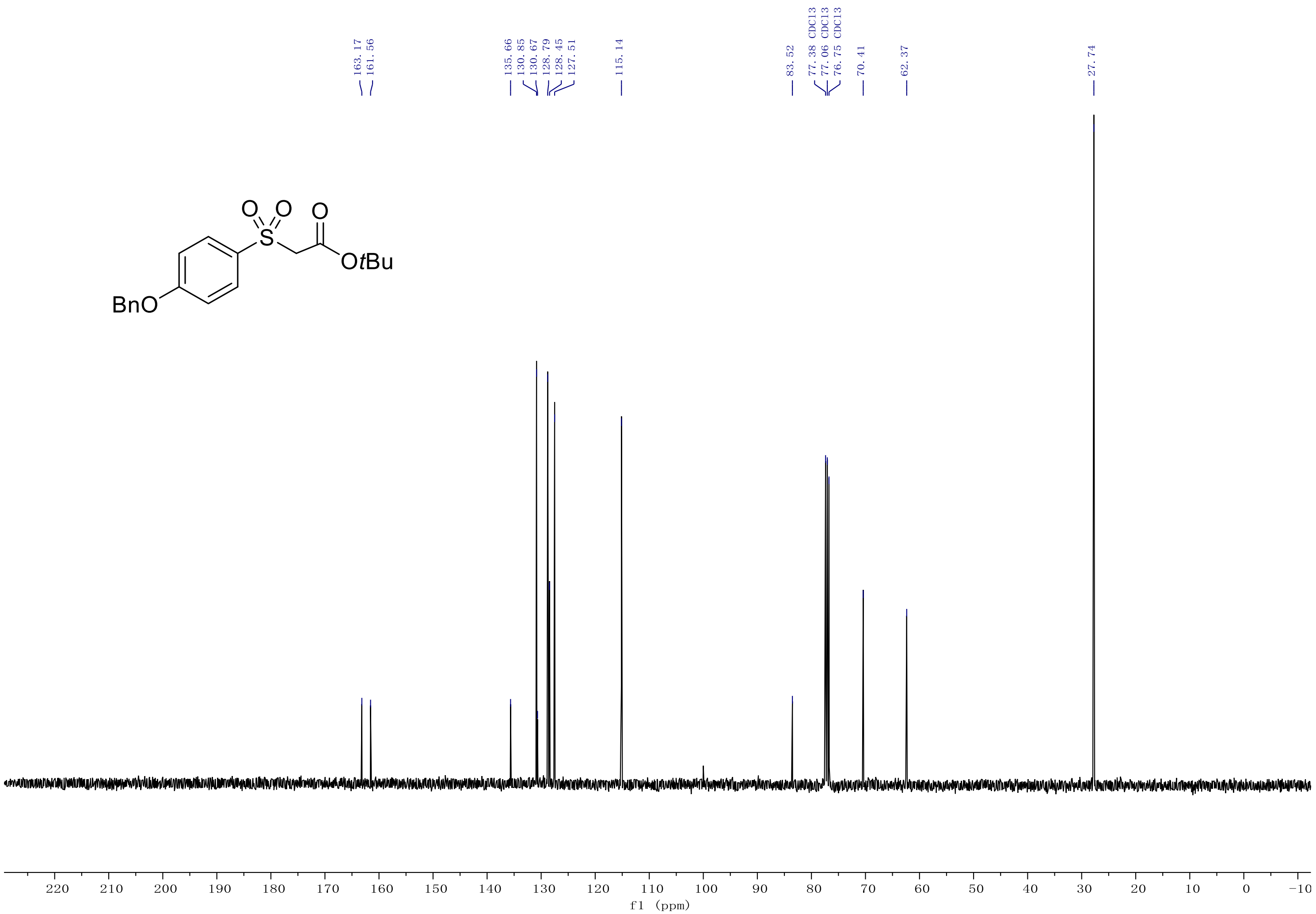

^{13}C NMR spectrum of ***tert*-butyl 2-((4-(benzyloxy)phenyl)sulfonyl)acetate 6c**

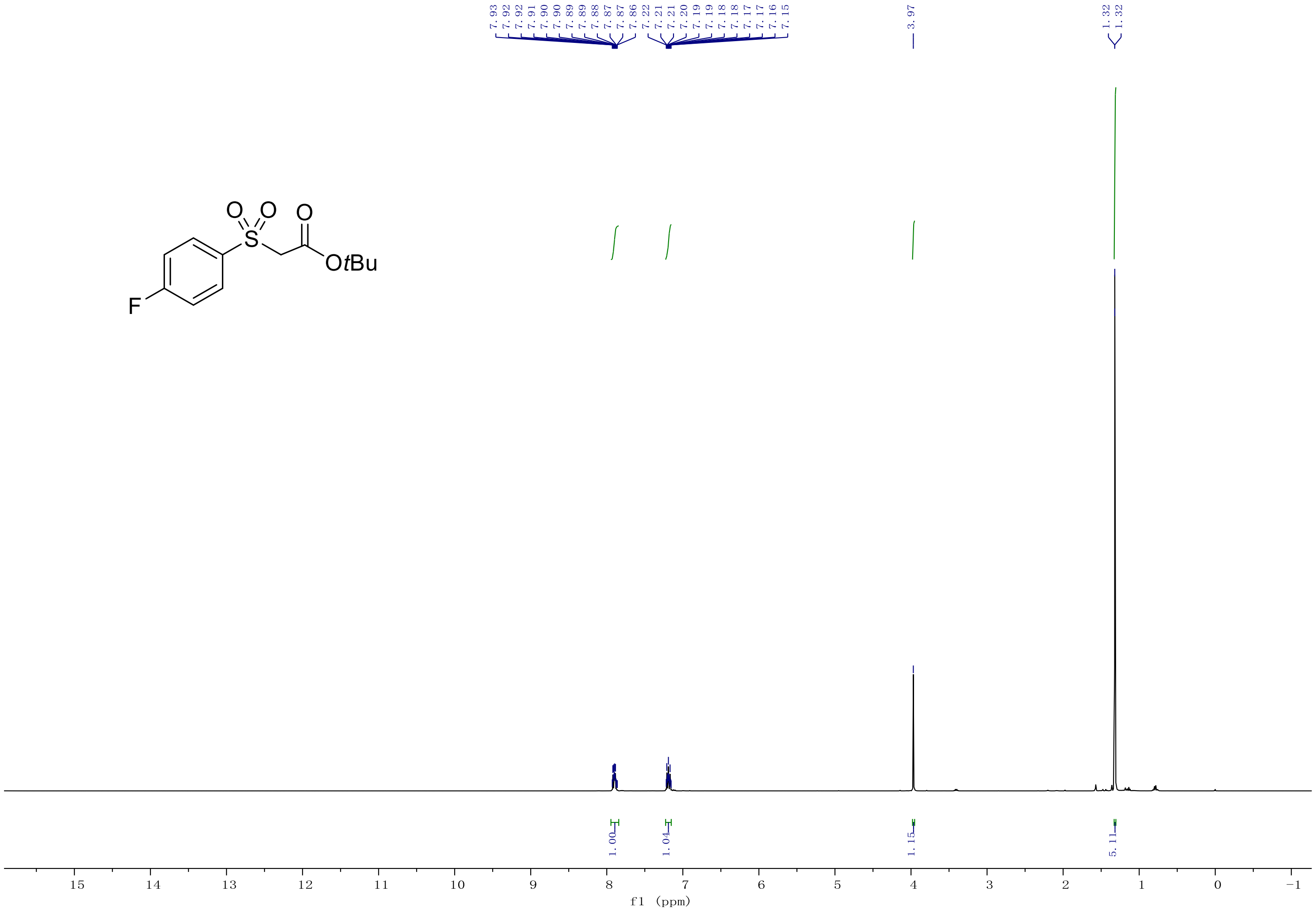

^{1}H NMR spectrum of ***tert*-butyl 2-((4-fluorophenyl)sulfonyl)acetate 6d**

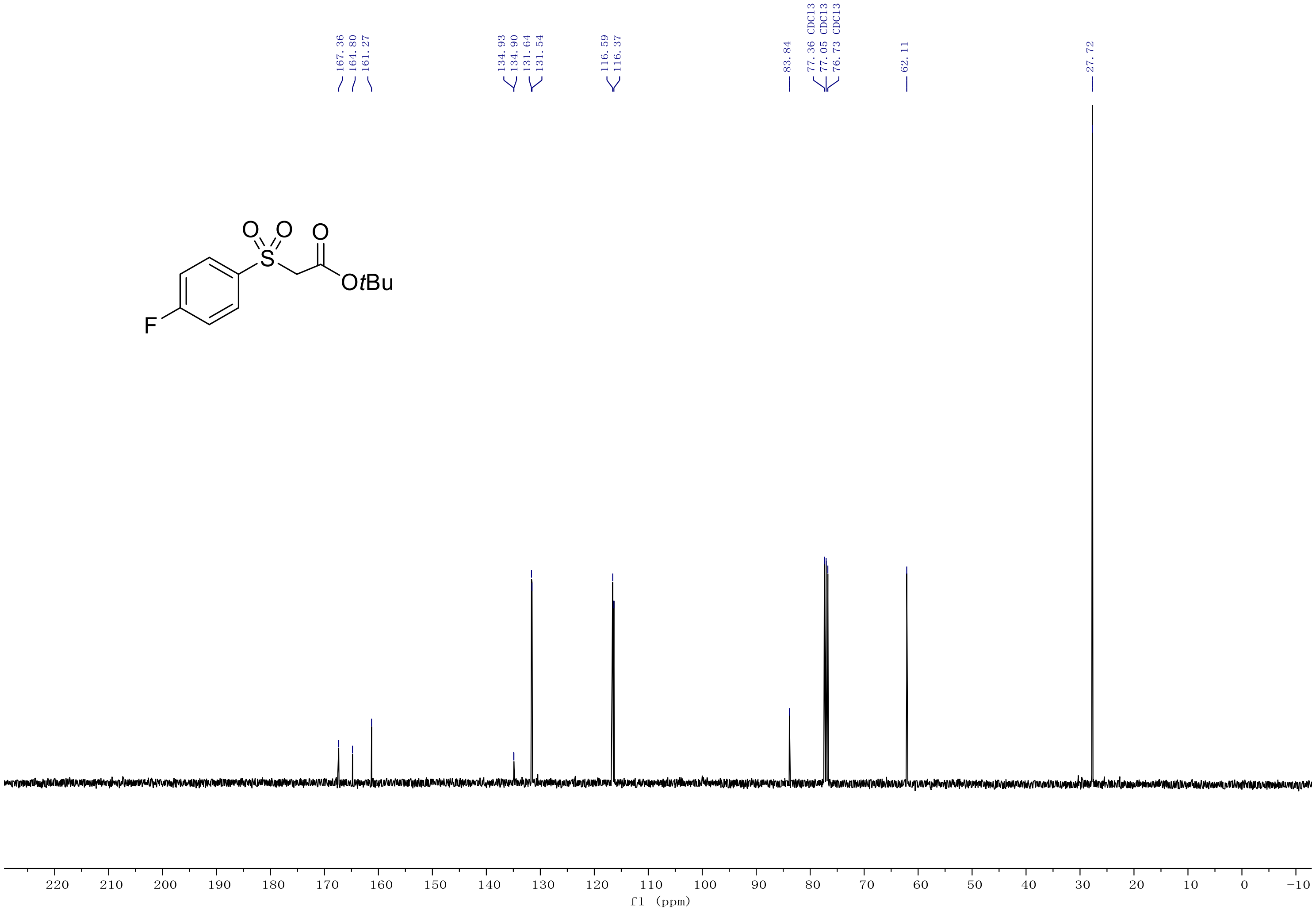

^{13}C NMR spectrum of ***tert*-butyl 2-((4-fluorophenyl)sulfonyl)acetate 6d**

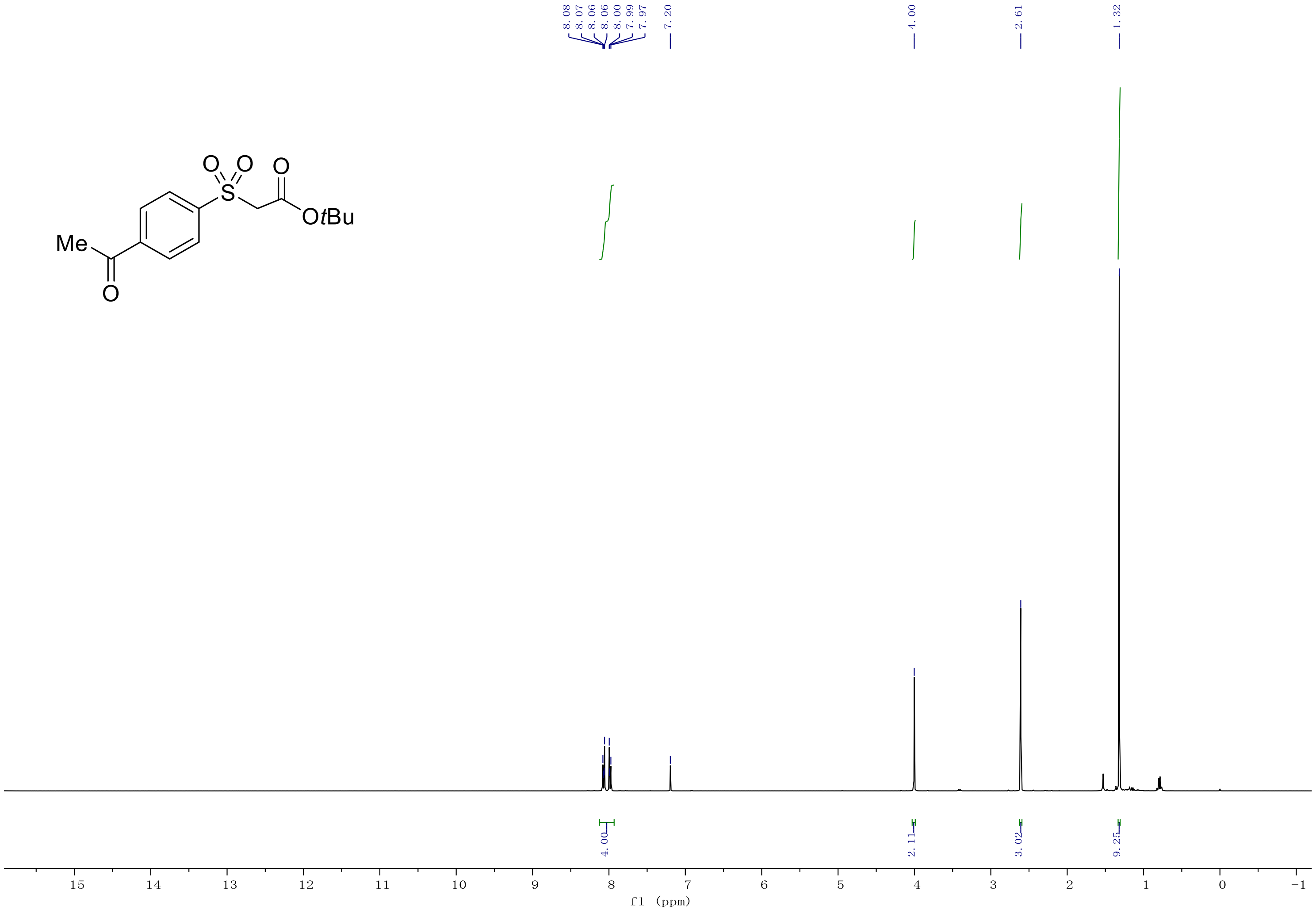

^{1}H NMR spectrum of ***tert*-butyl 2-((4-(benzyloxy)phenyl)sulfonyl)acetate 6e**

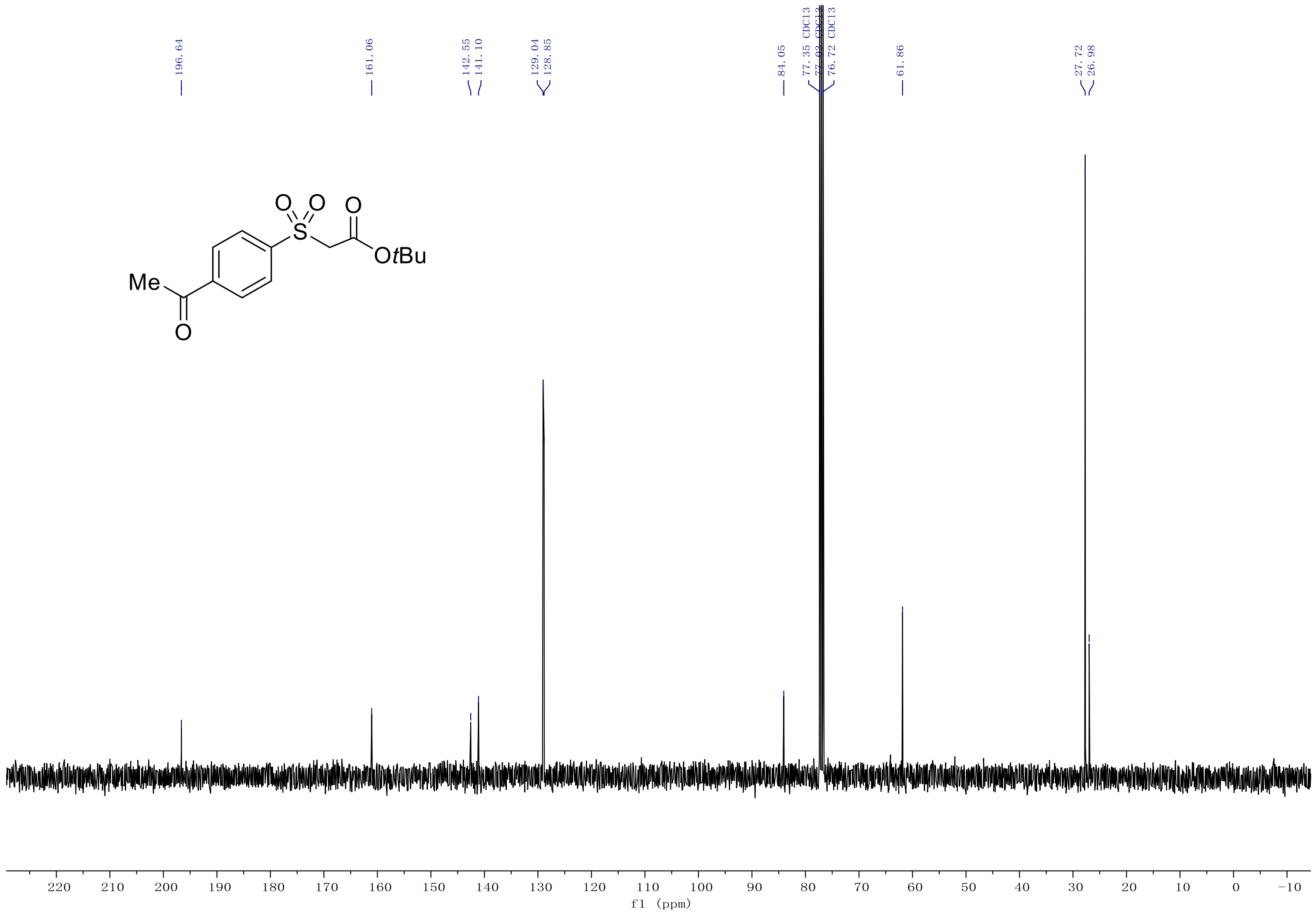

^{1}H NMR and ^{13}C NMR spectra of ***tert*-butyl 2-((4-(benzyloxy)phenyl)sulfonyl)acetate 6e**

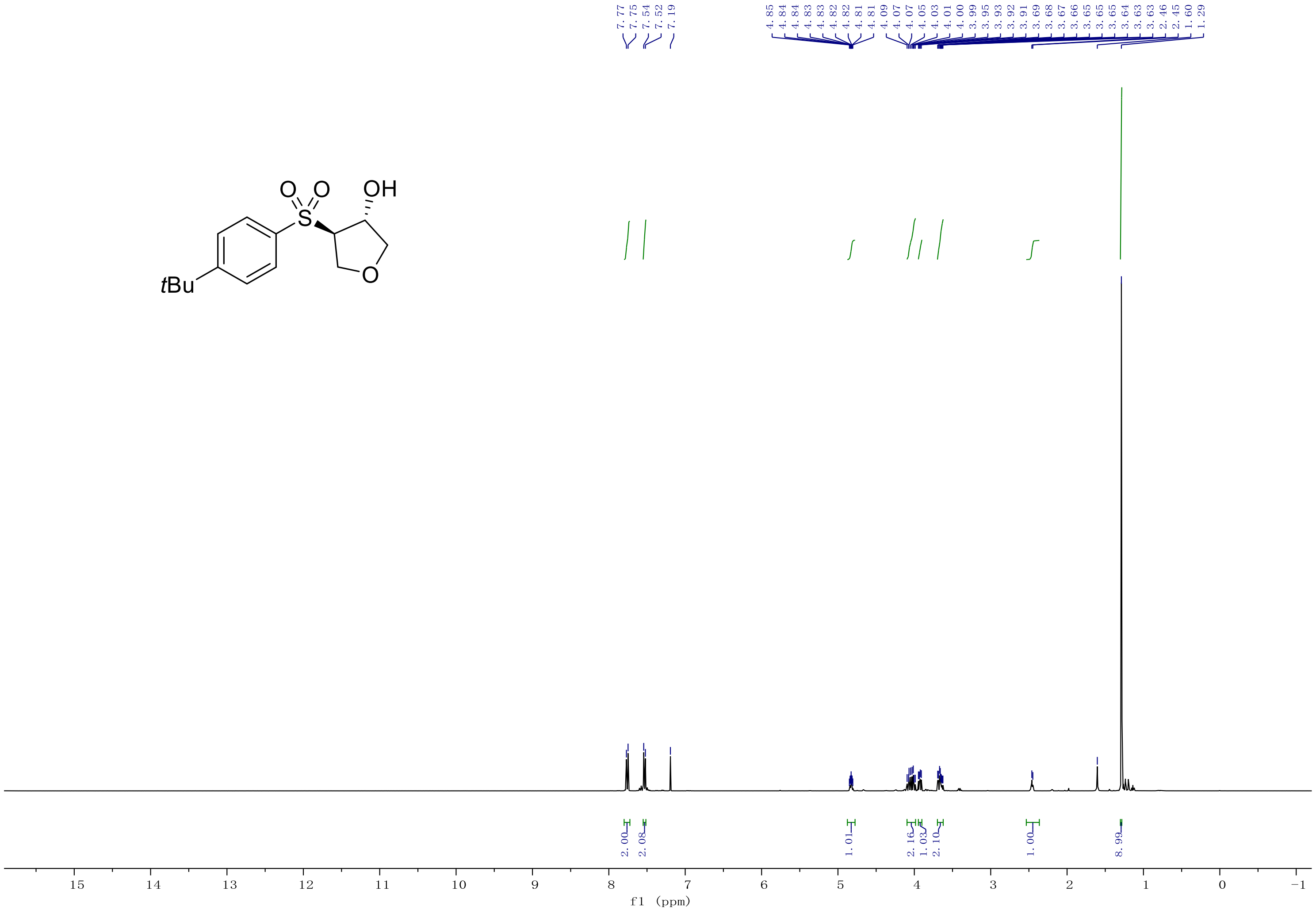

^{1}H NMR and ^{13}C NMR spectra of **4-(4-(*tert*-butyl)phenyl)sulfonyl)tetrahydrofuran-3-ol 6f**

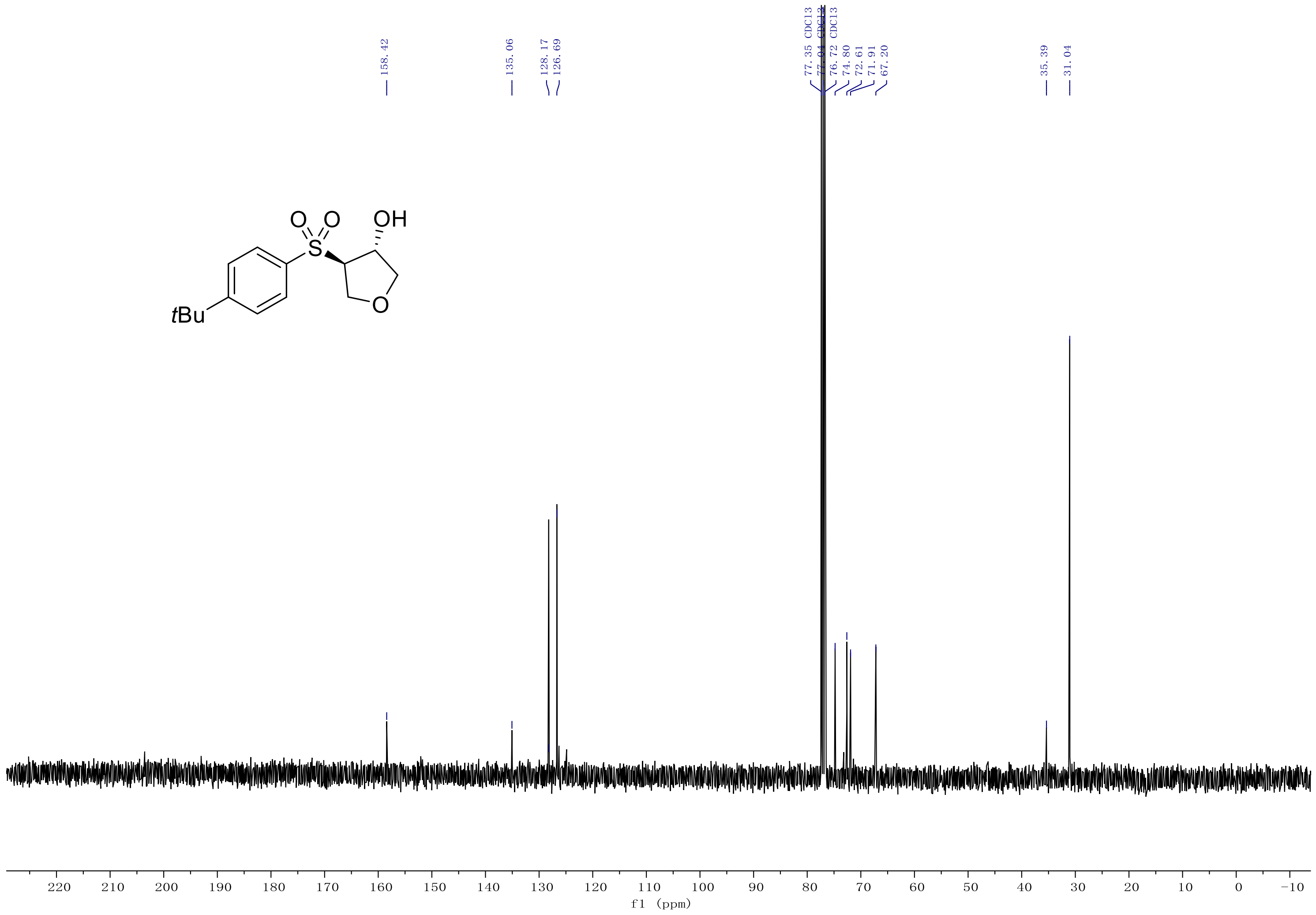

^{1}H NMR and ^{13}C NMR spectra of **4-(4-(*tert*-butyl)phenyl)sulfonyl)tetrahydrofuran-3-ol 6f**

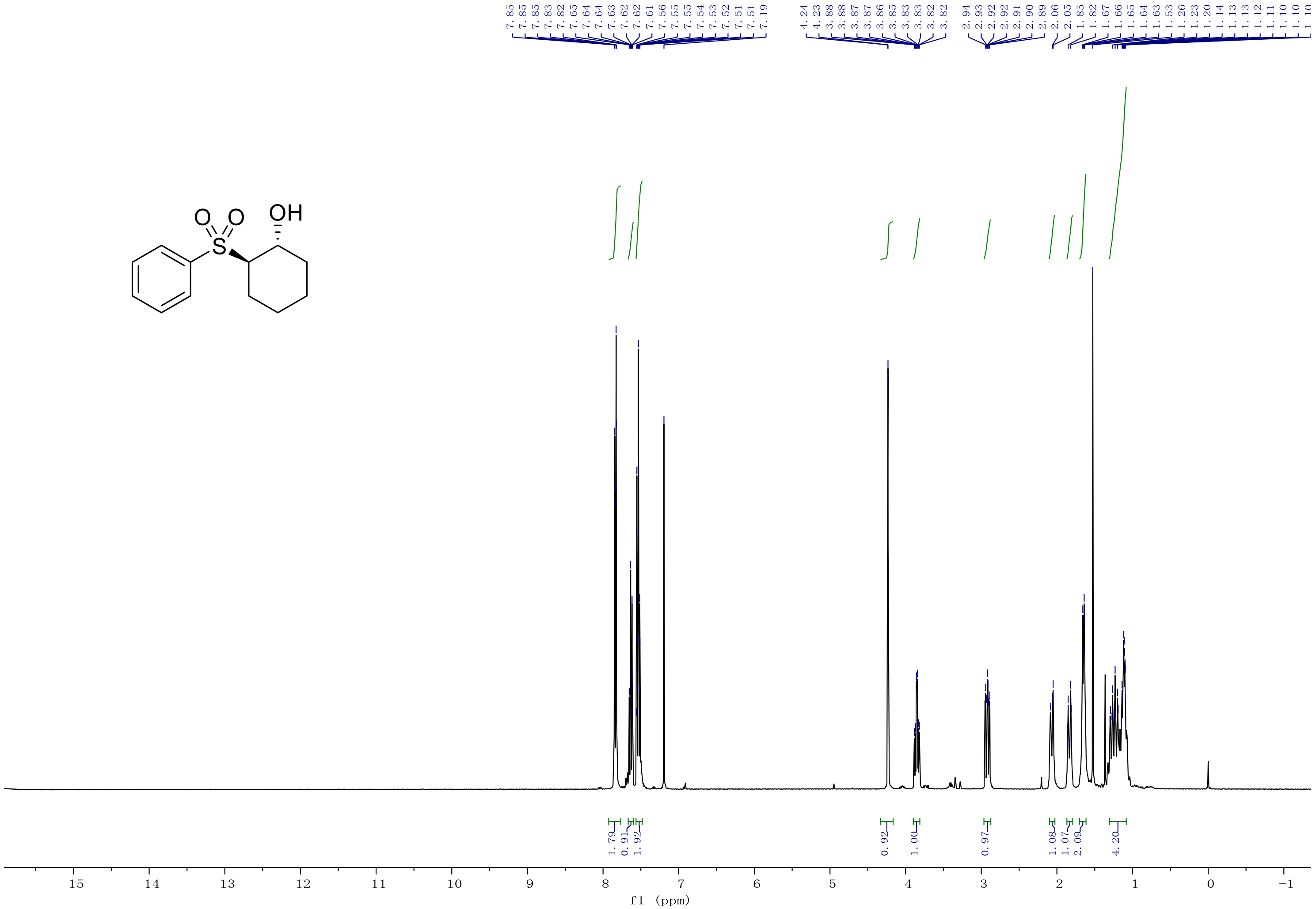

^{1}H NMR spectrum of **2-(phenylsulfonyl)cyclohexan-1-ol 6g**

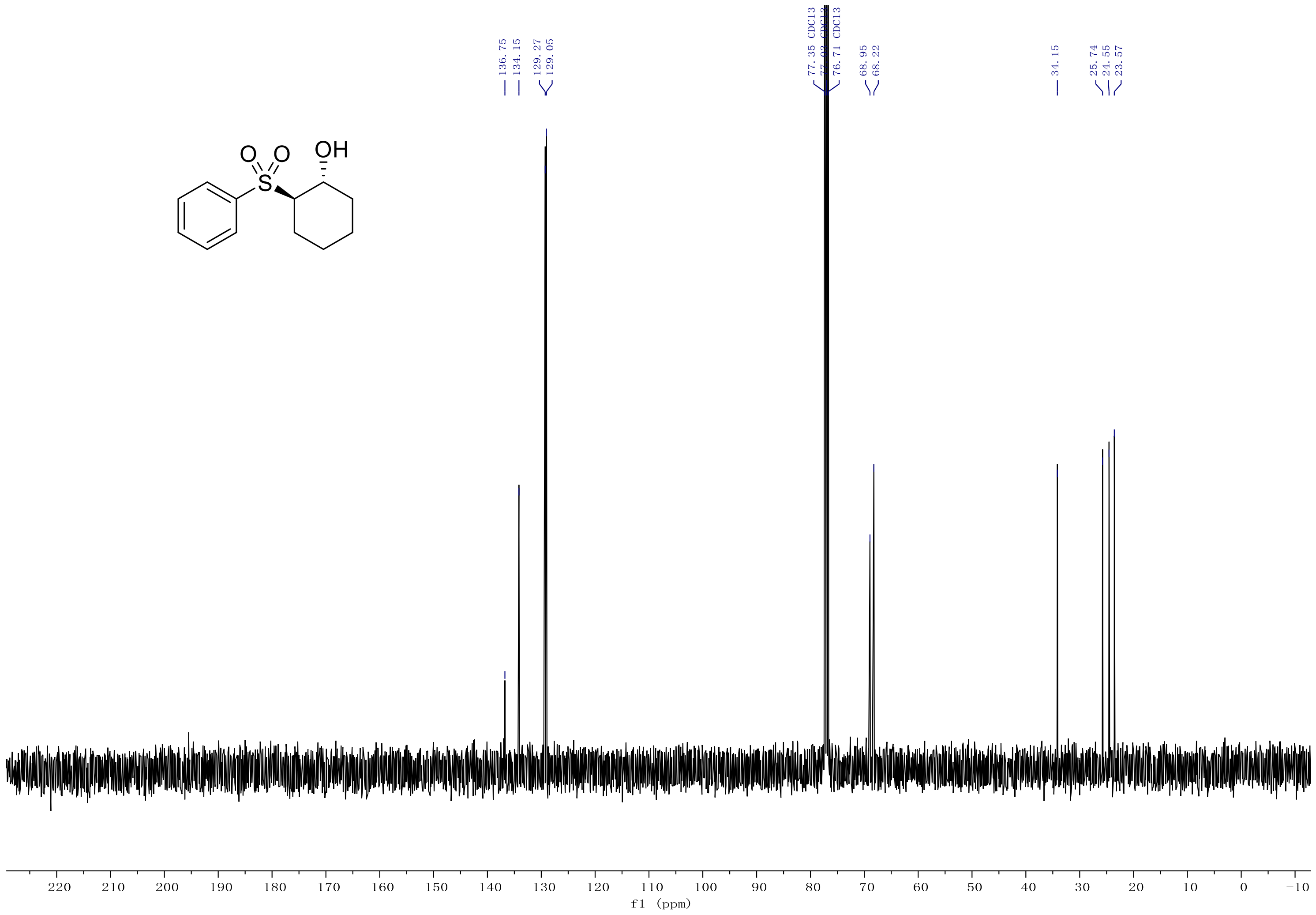

^{1}H NMR and ^{13}C NMR spectra of **2-(phenylsulfonyl)cyclohexan-1-ol 6g**

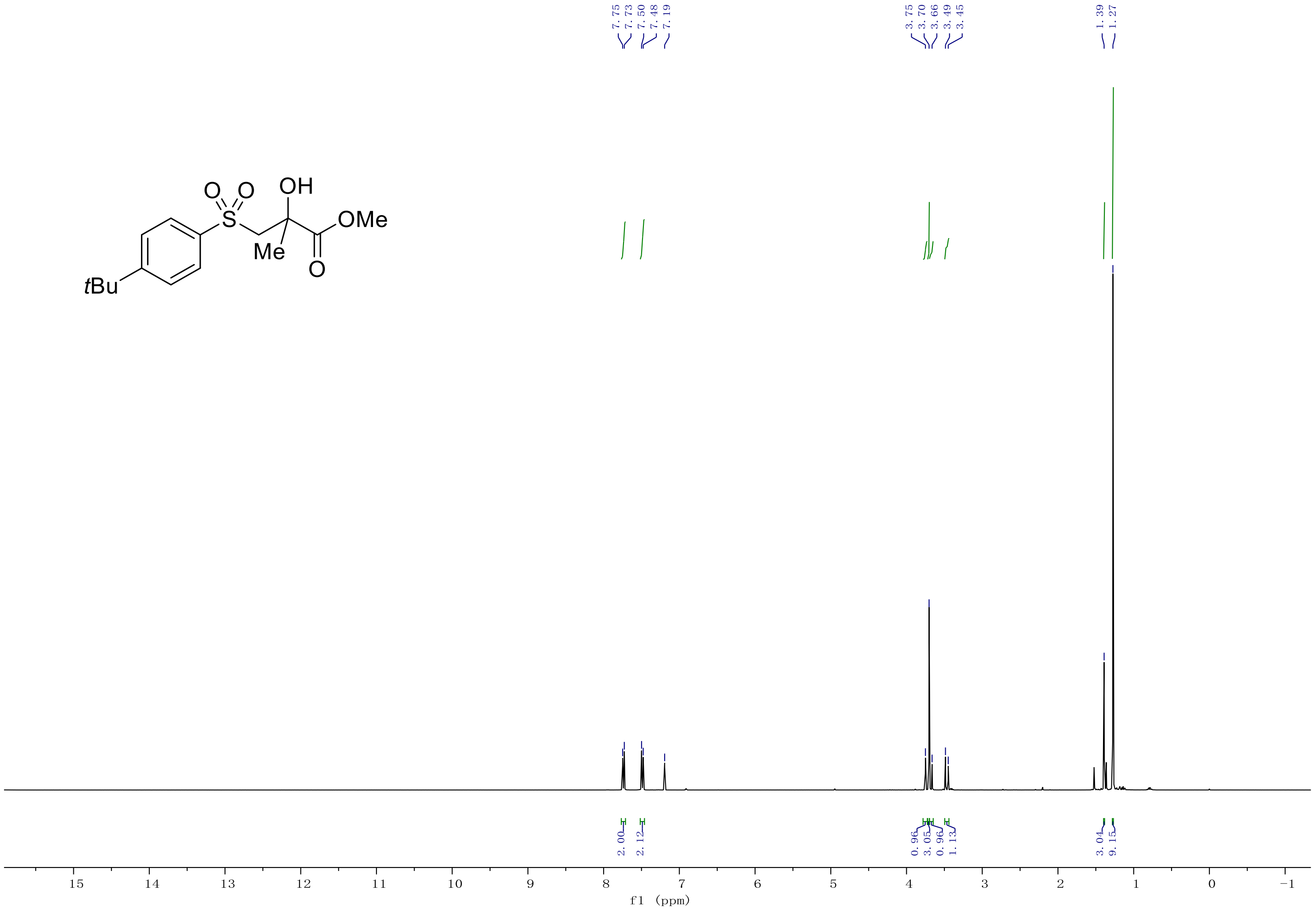

^{1}H NMR spectrum of **2-methyl 3-((4-(*tert*-butyl)phenyl)sulfonyl)-2-hydroxy-2-methylpropanoate 6h**

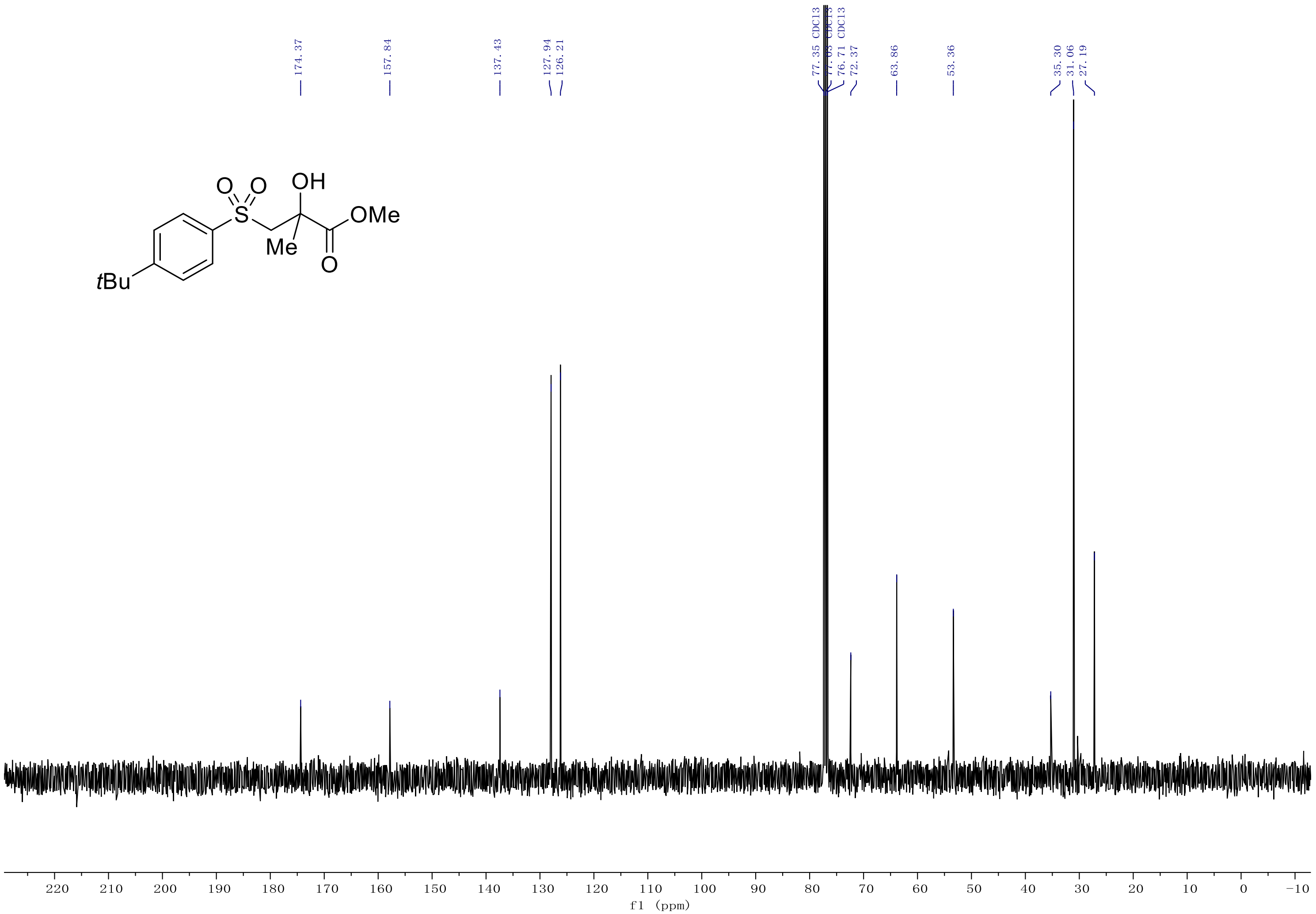

^{13}C NMR spectrum of **2-methyl 3-((4-(*tert*-butyl)phenyl)sulfonyl)-2-hydroxy-2-methylpropanoate 6h**

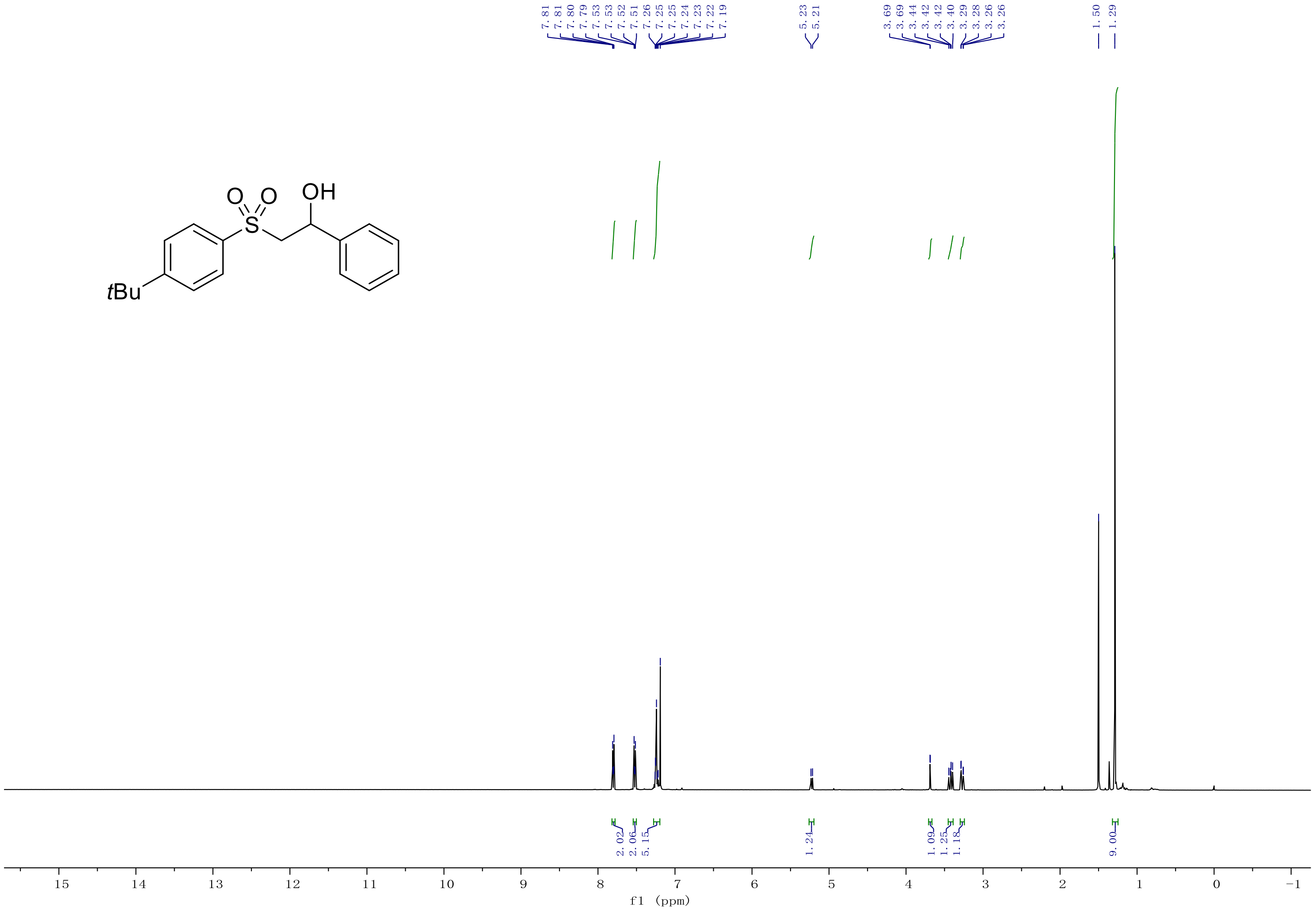

^{1}H NMR spectrum of **2-((4-(*tert*-butyl)phenyl)sulfonyl)-1-phenylethan-1-ol 6i**

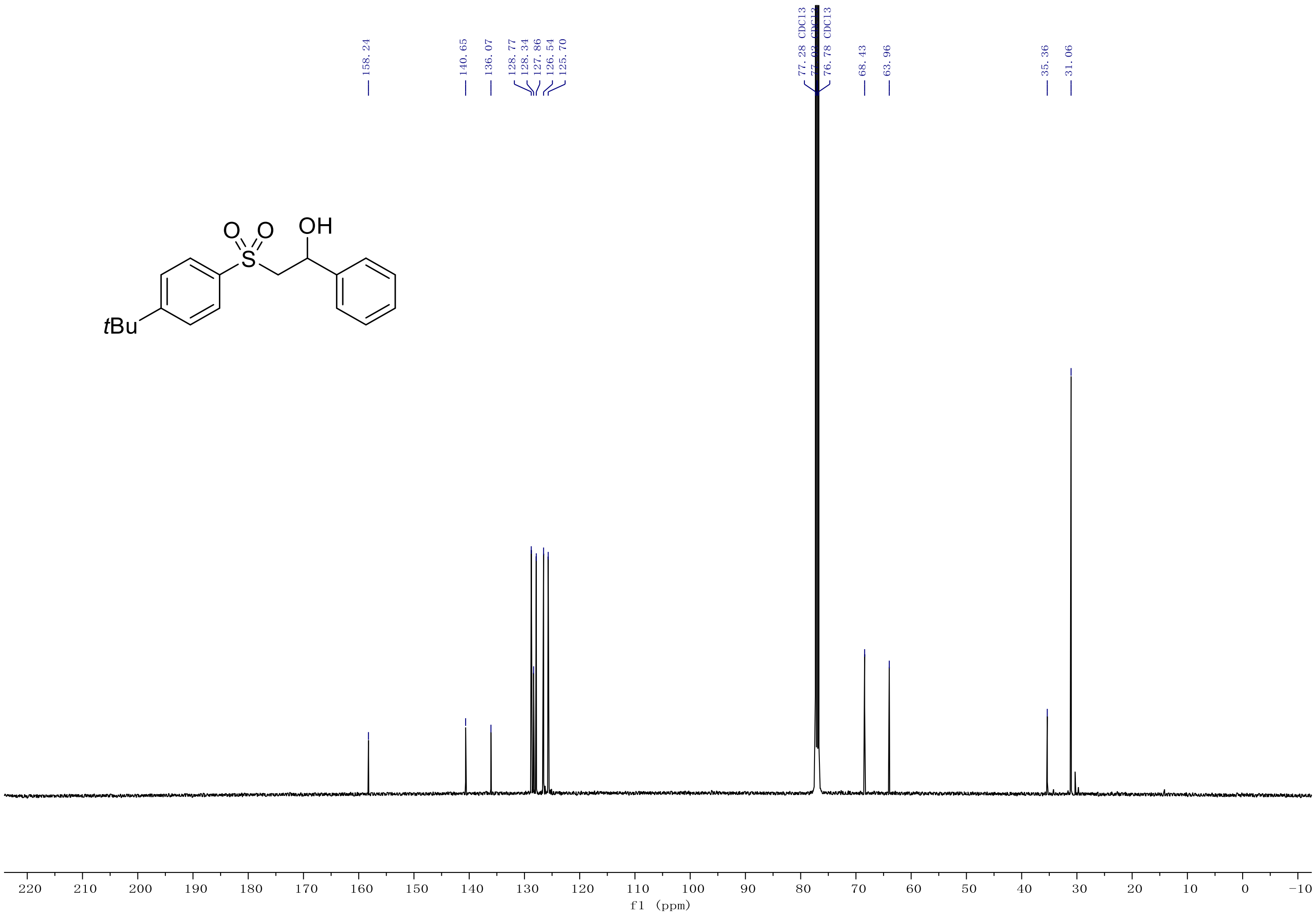

^{13}C NMR spectrum of **2-((4-(*tert*-butyl)phenyl)sulfonyl)-1-phenylethan-1-ol 6i**

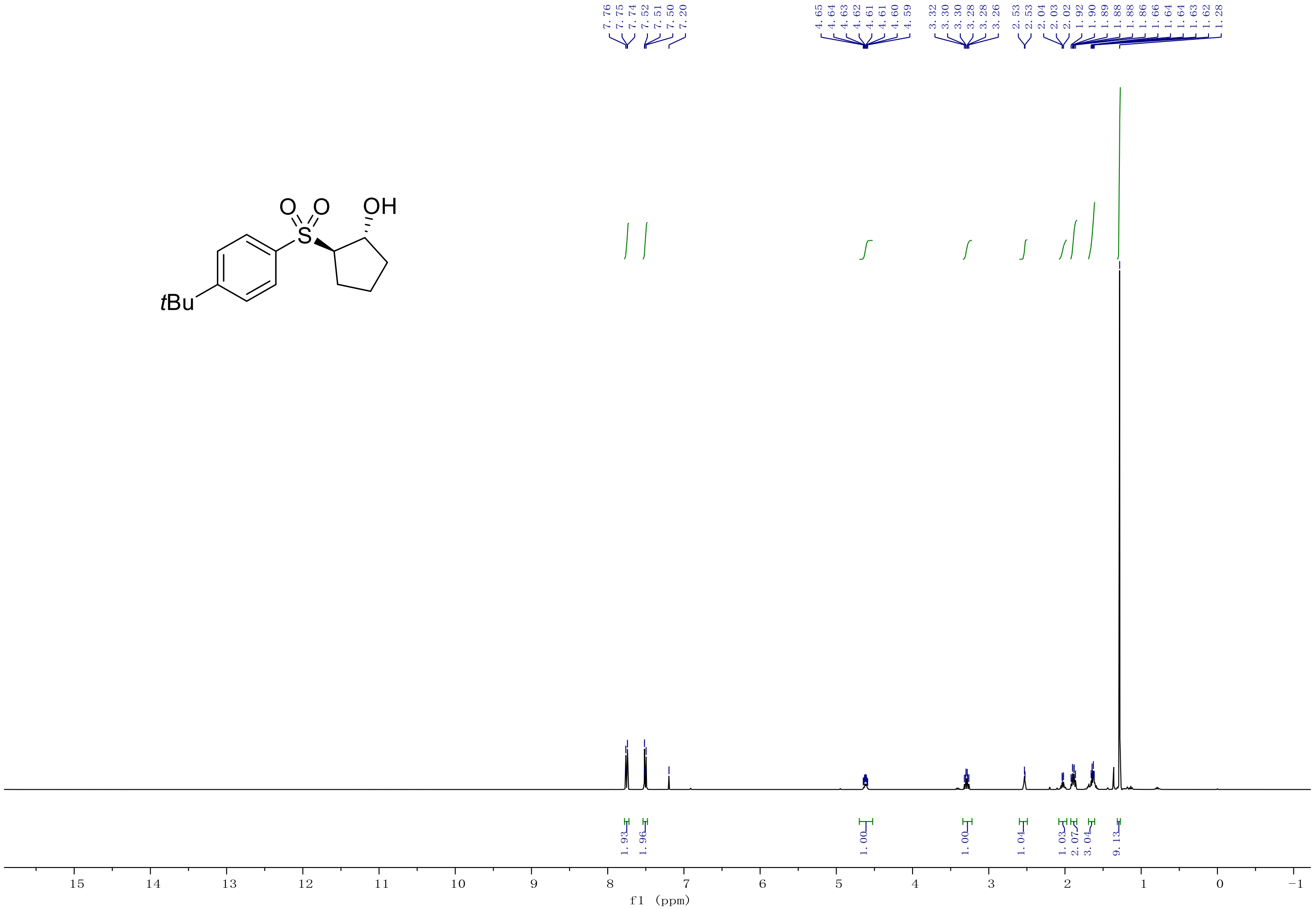

^{1}H NMR spectrum of **2-((4-(*tert*-butyl)phenyl)sulfonyl)cyclopentan-1-ol 6j**

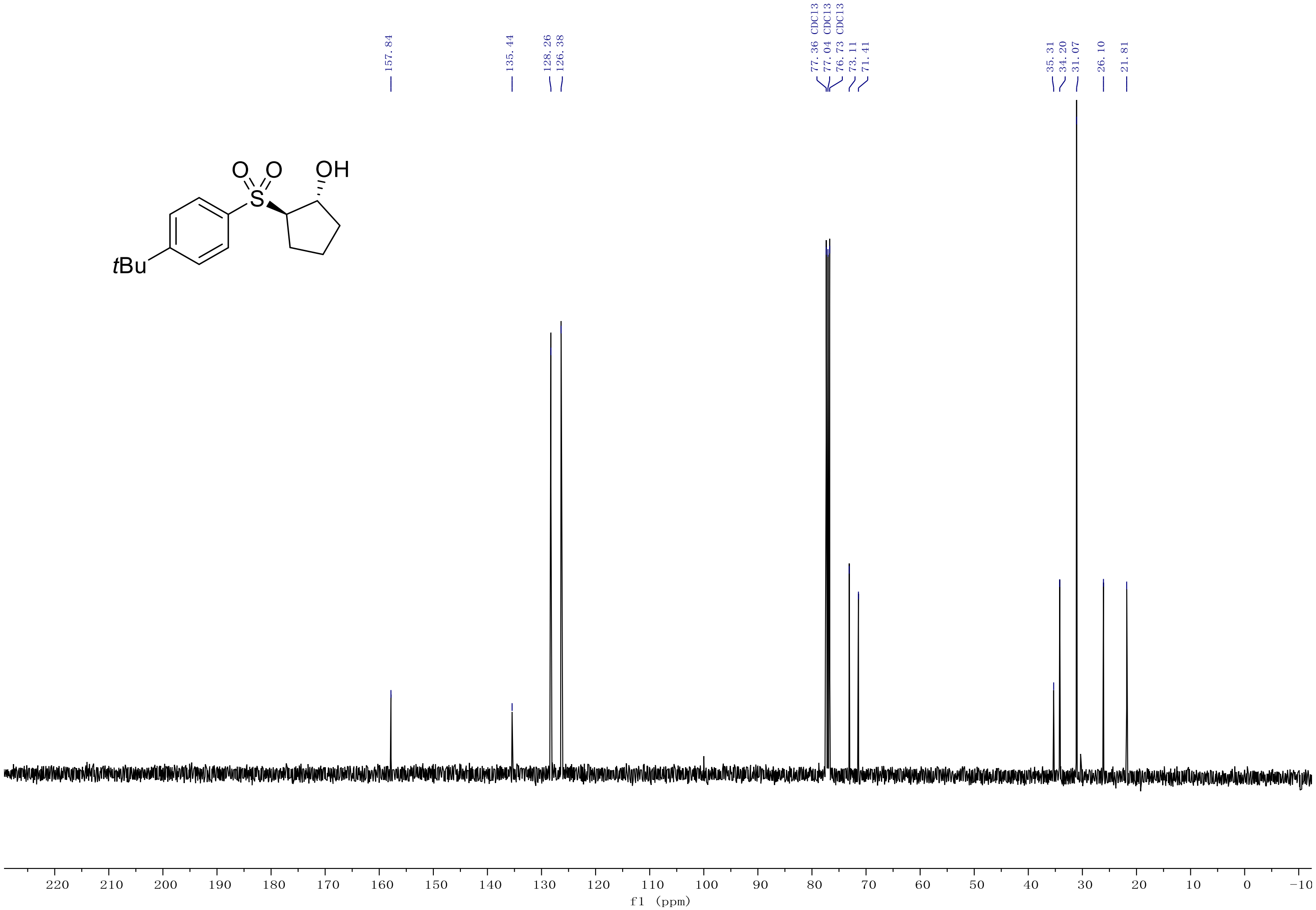

^{13}C NMR spectrum of **2-((4-(*tert*-butyl)phenyl)sulfonyl)cyclopentan-1-ol 6j**

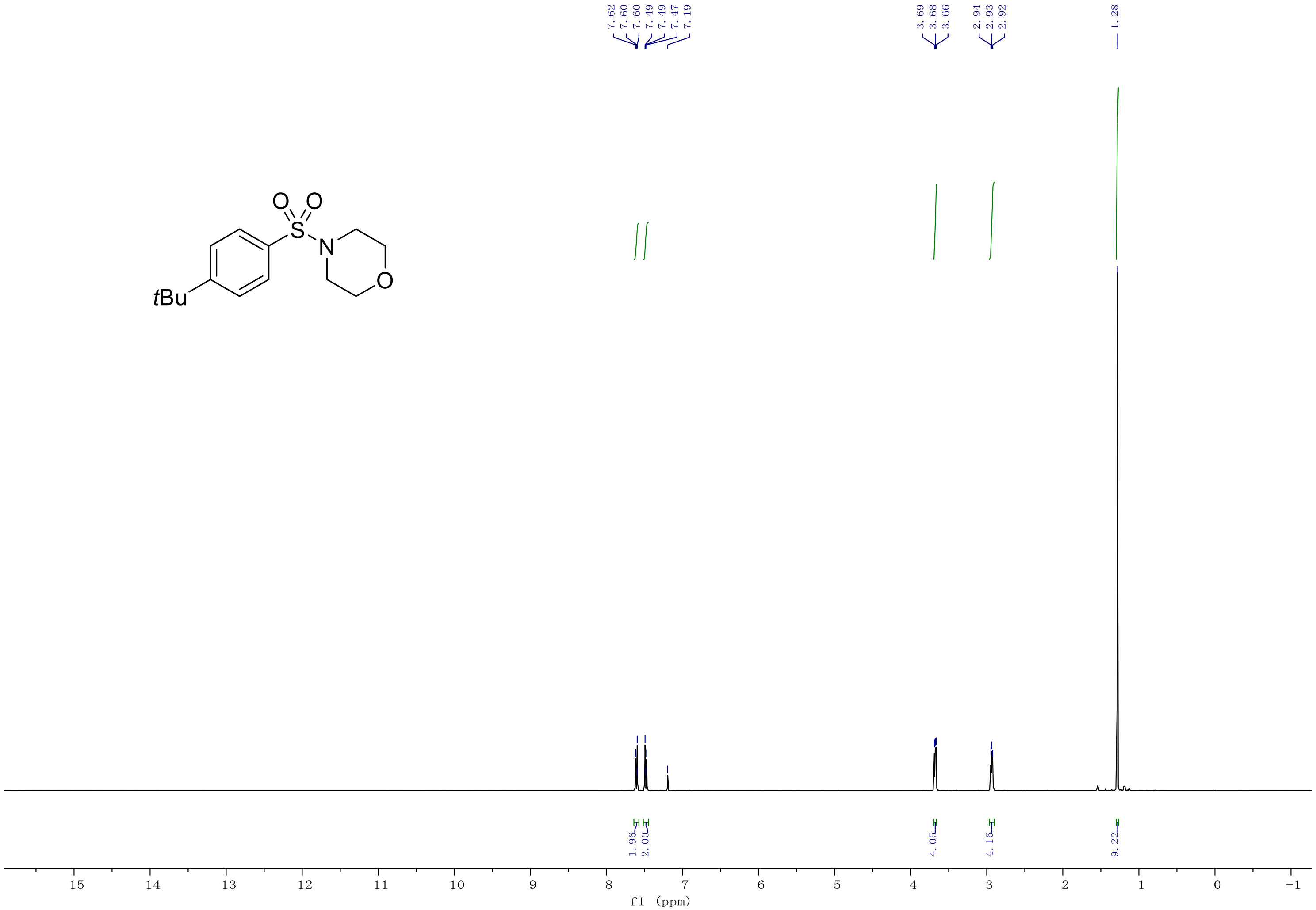

^{1}H NMR spectrum of **4-{[4-(*tert*-butyl)phenyl]sulfonyl}morpholine 6k**

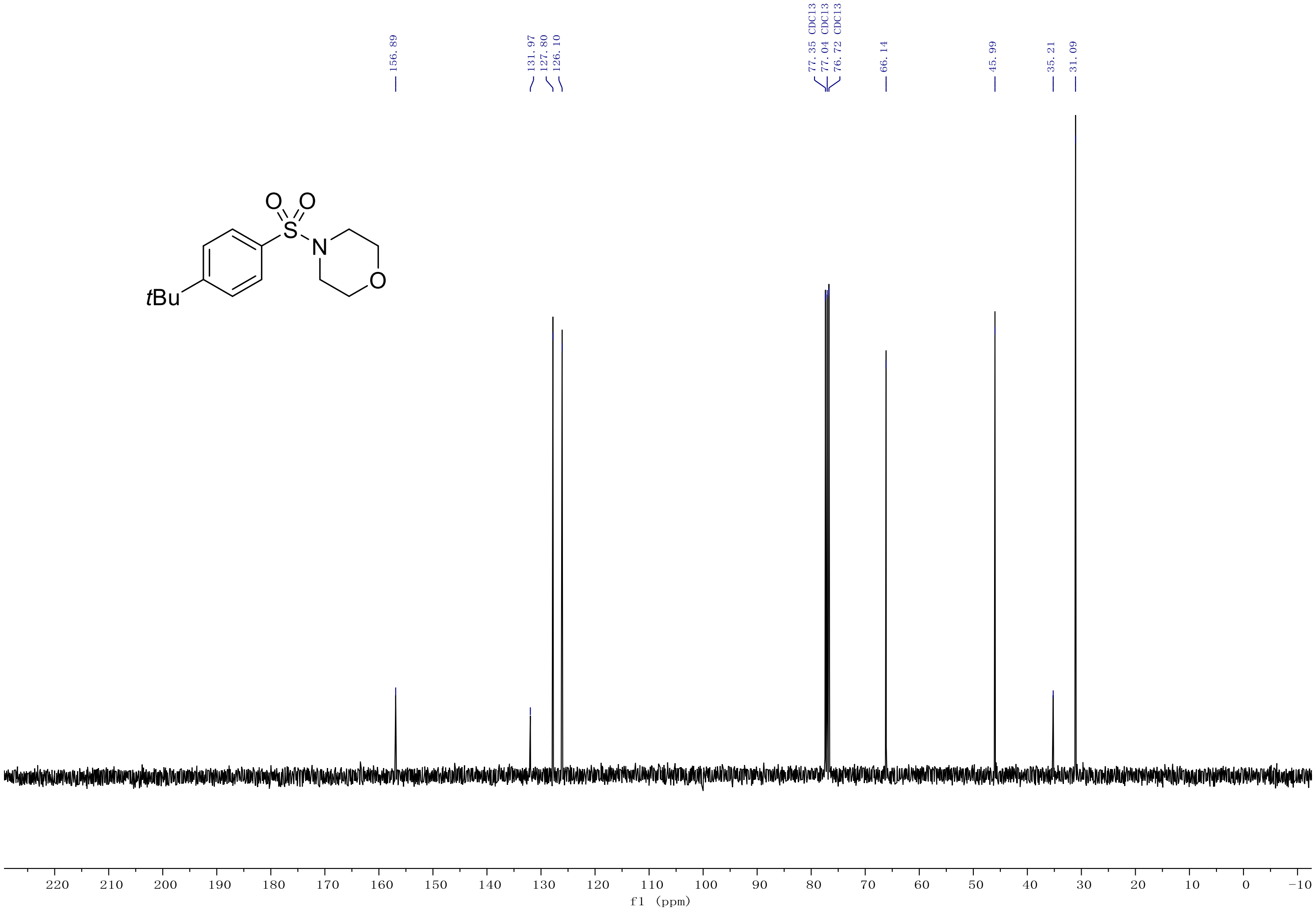

^{13}C NMR spectrum of **4-{[4-(*tert*-butyl)phenyl]sulfonyl}morpholine 6k**

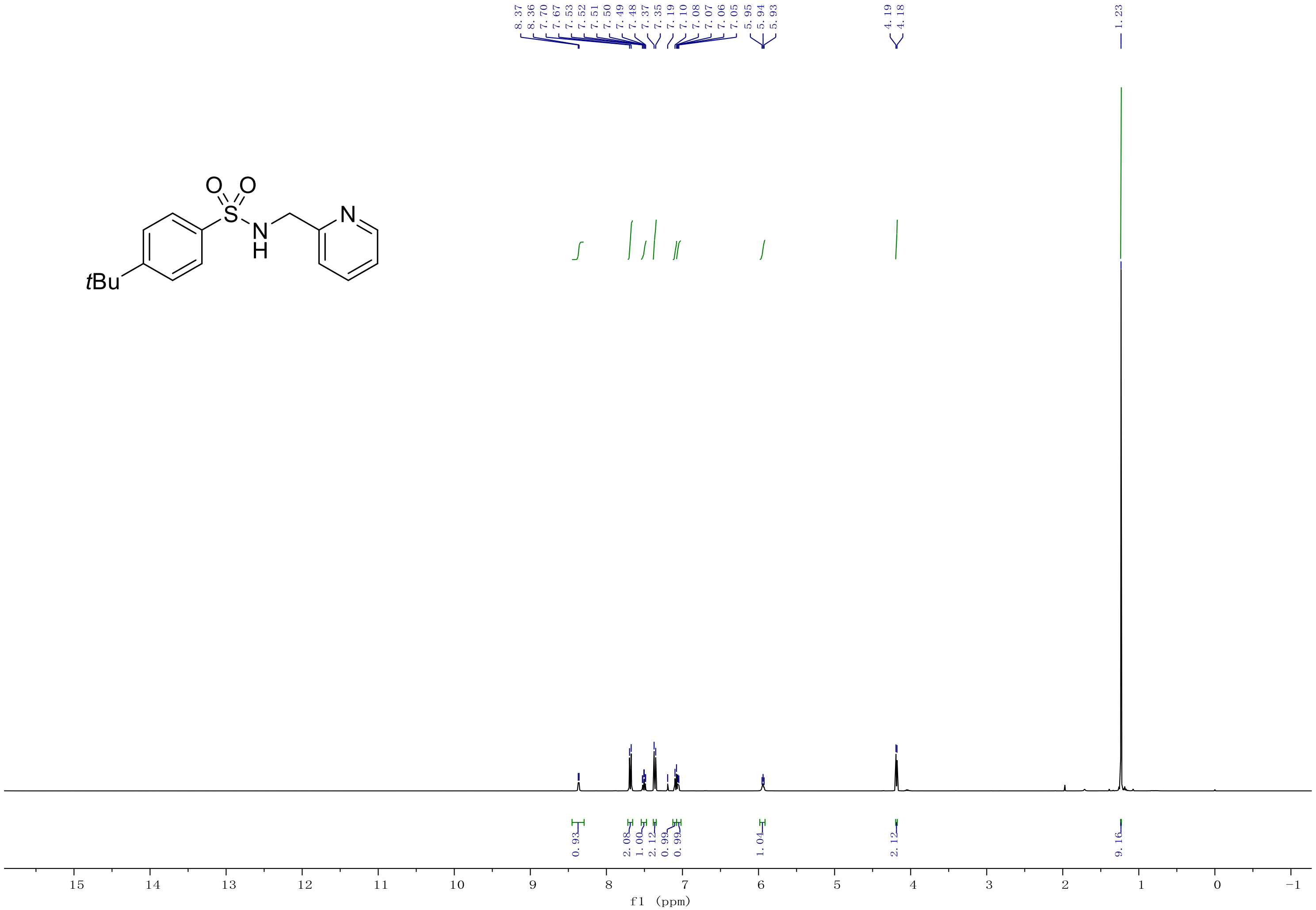

^{1}H NMR spectrum of **4-(*tert*-butyl)-N-(pyridin-2-ylmethyl)benzenesulfonamide 6l**

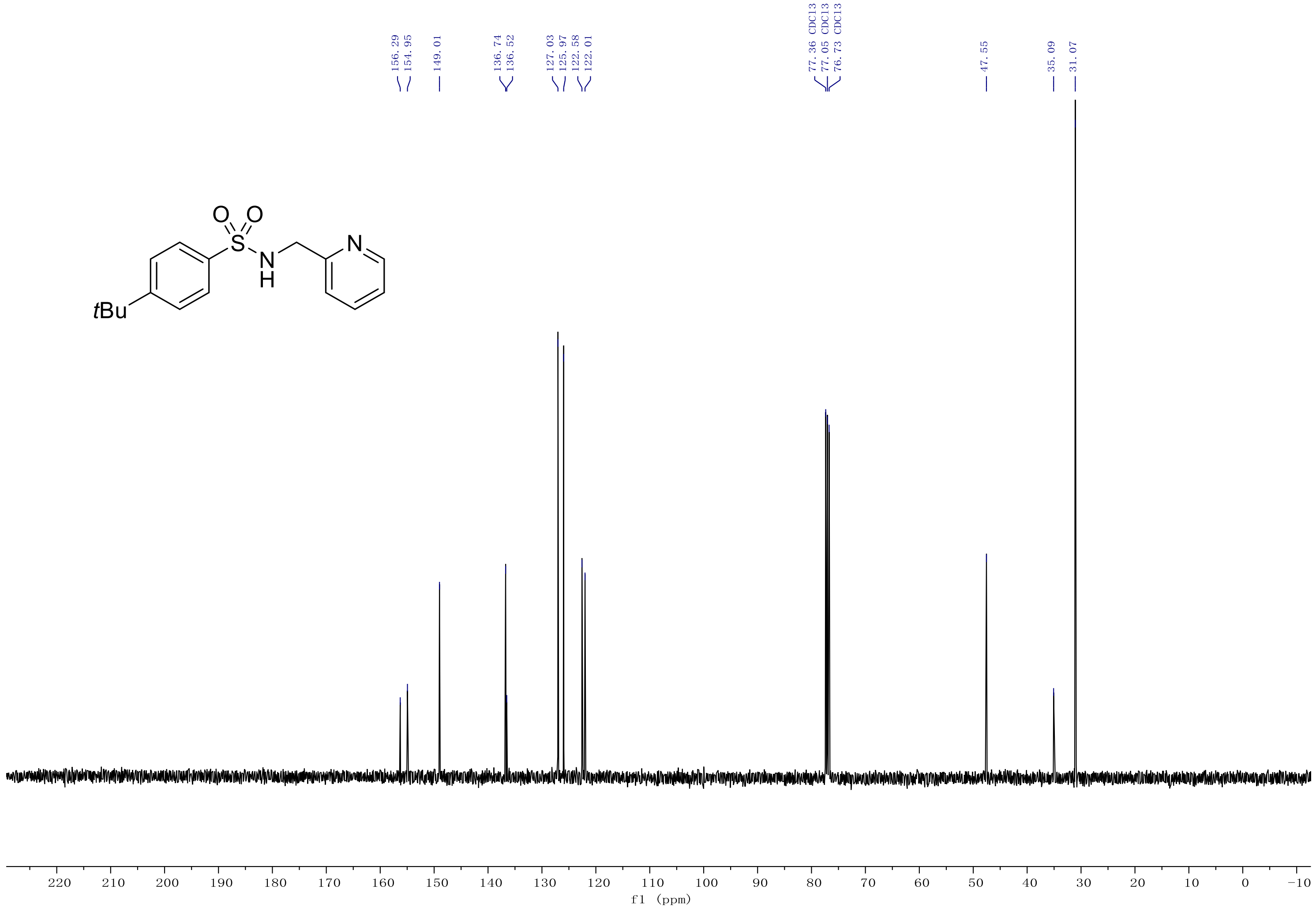

^{13}C NMR spectrum of **4-(*tert*-butyl)-N-(pyridin-2-ylmethyl)benzenesulfonamide 6l**

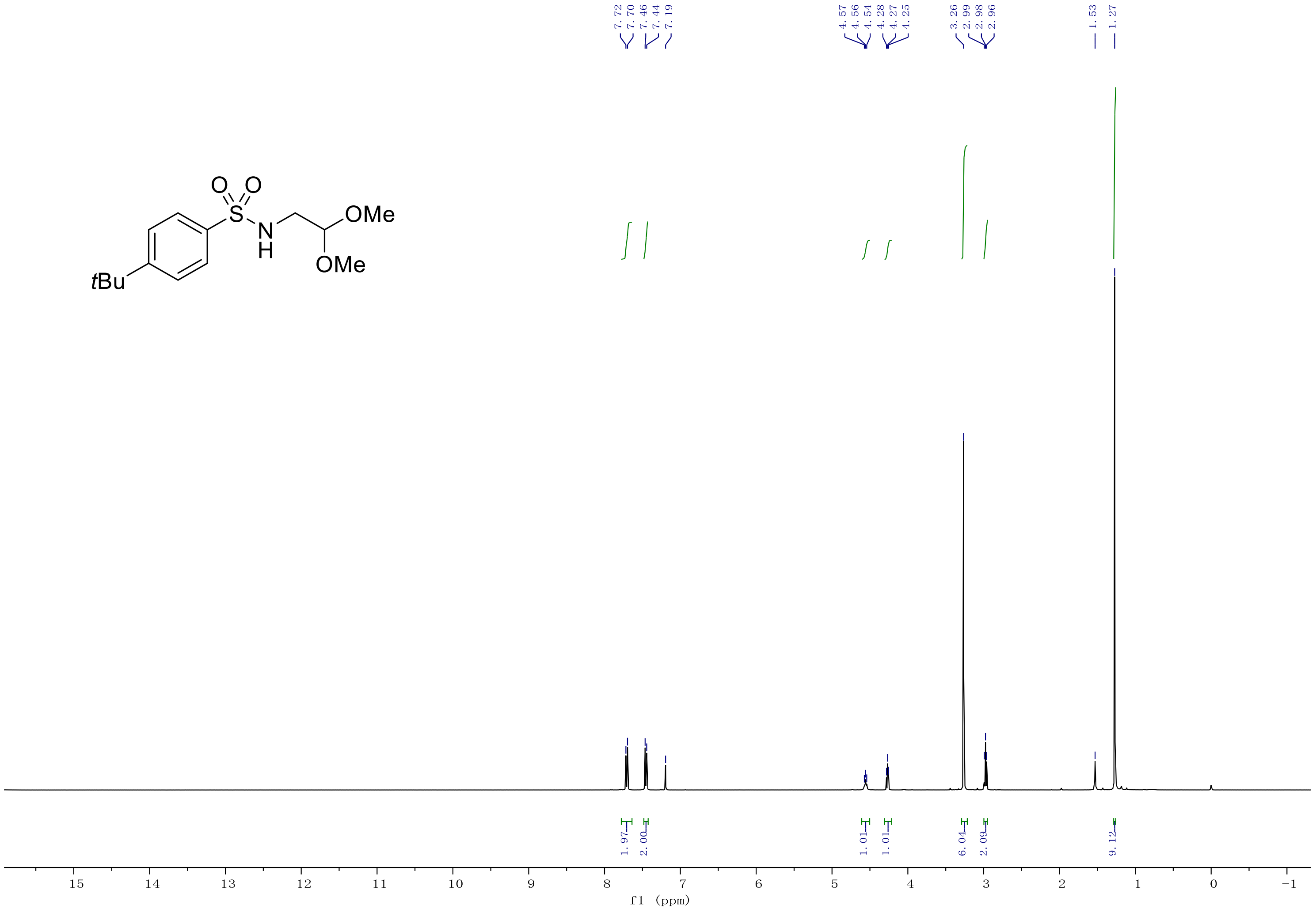

^{1}H NMR spectrum of **4-(*tert*-butyl)-N-(2,2-dimethoxyethyl)benzenesulfonamide 6m**

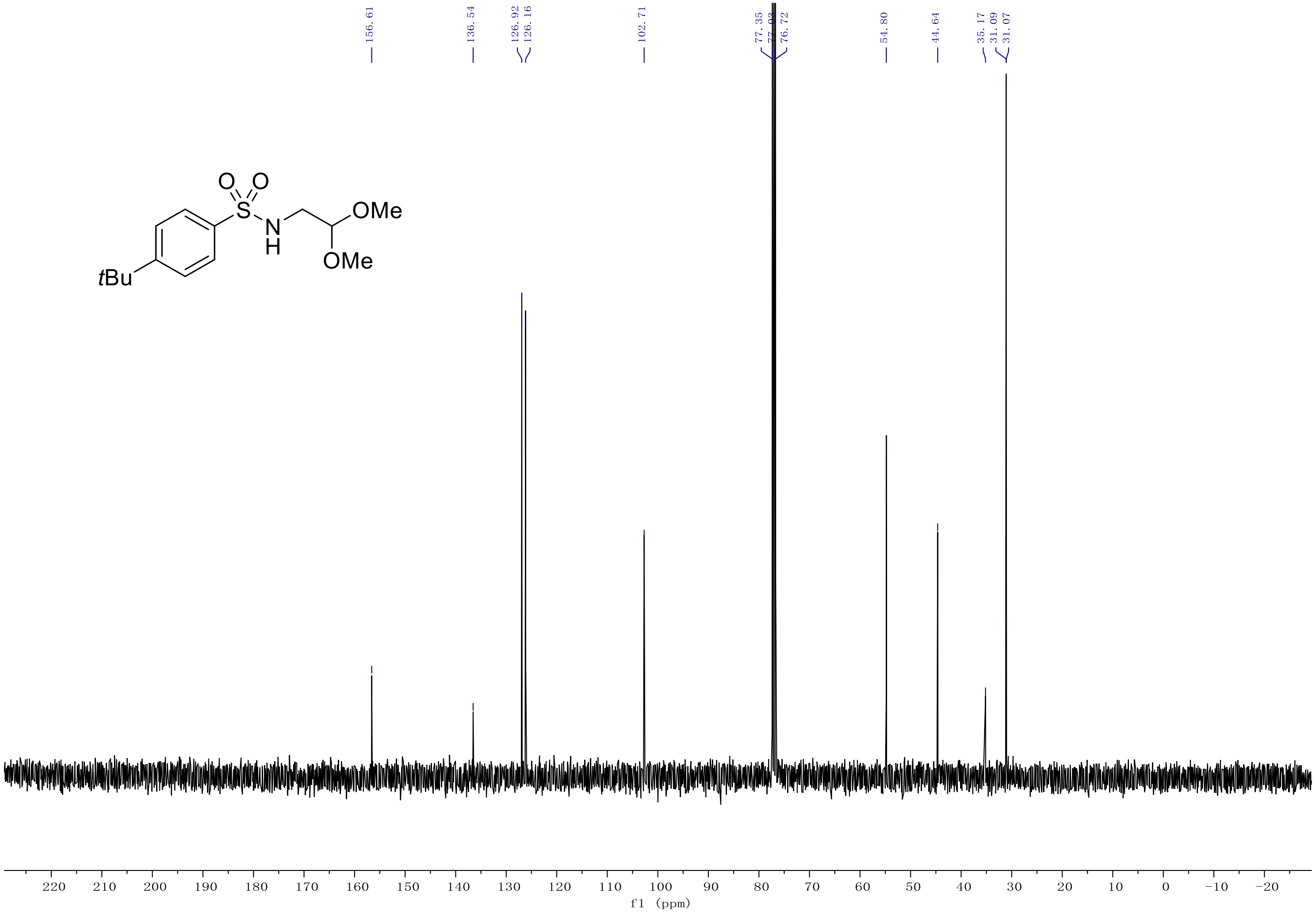

^{13}C NMR spectrum of **4-(*tert*-butyl)-N-(2,2-dimethoxyethyl)benzenesulfonamide 6m**

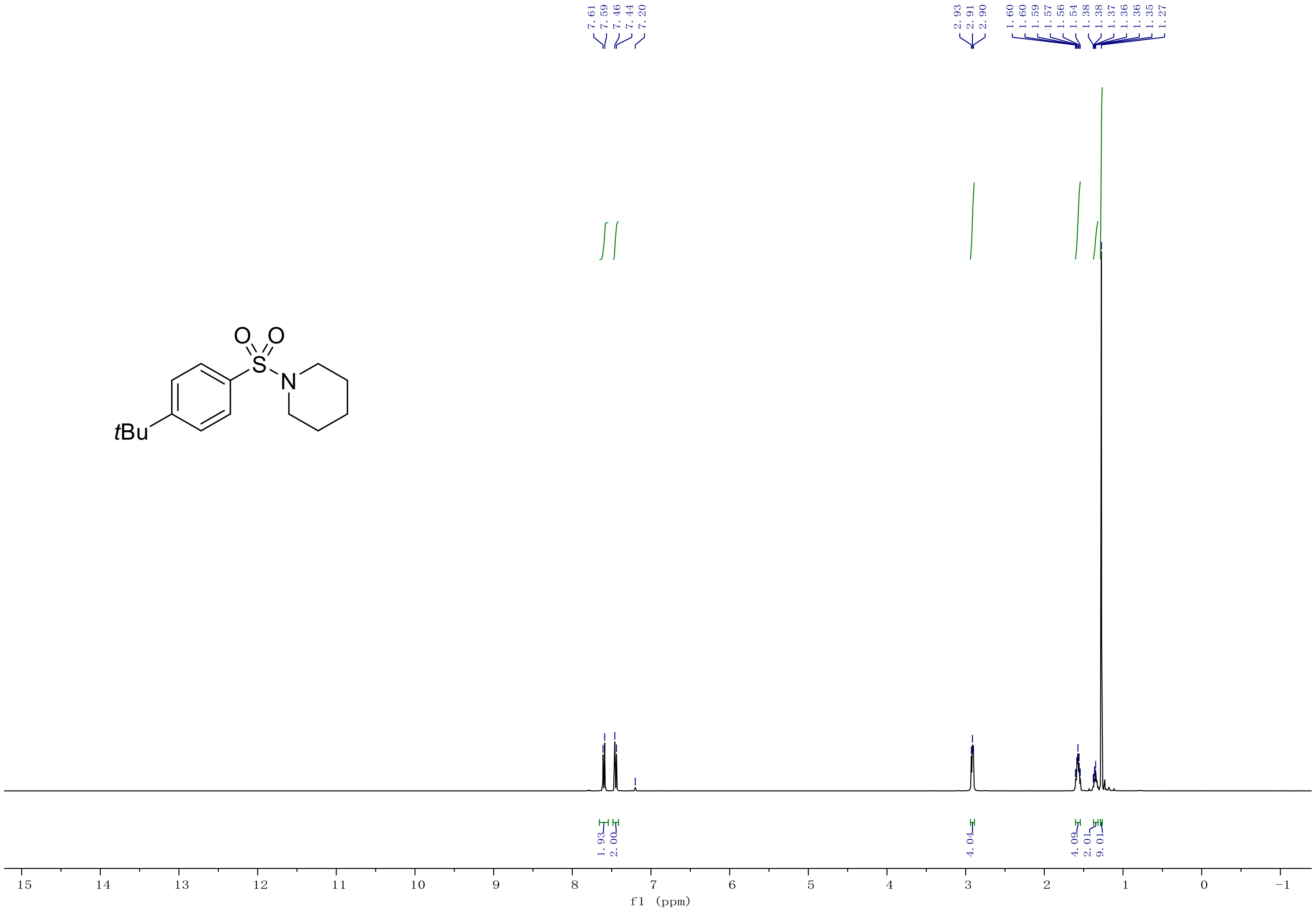

¹H NMR spectrum of **1-((4-(*tert*-butyl)phenyl)sulfonyl)piperidine 6n**

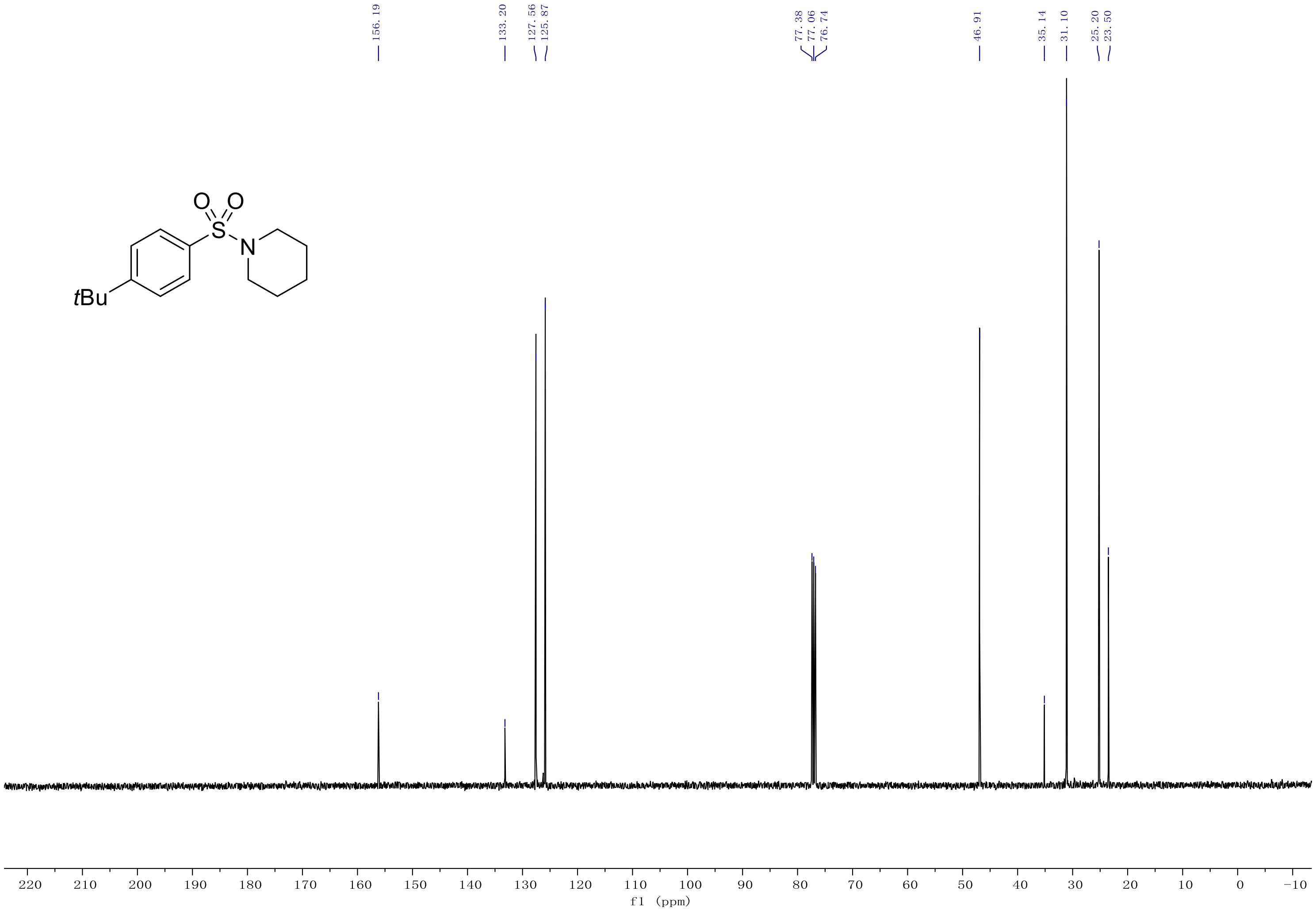

^{1}H NMR and ^{13}C NMR spectra of **1-((4-(*tert*-butyl)phenyl)sulfonyl)piperidine 6n**

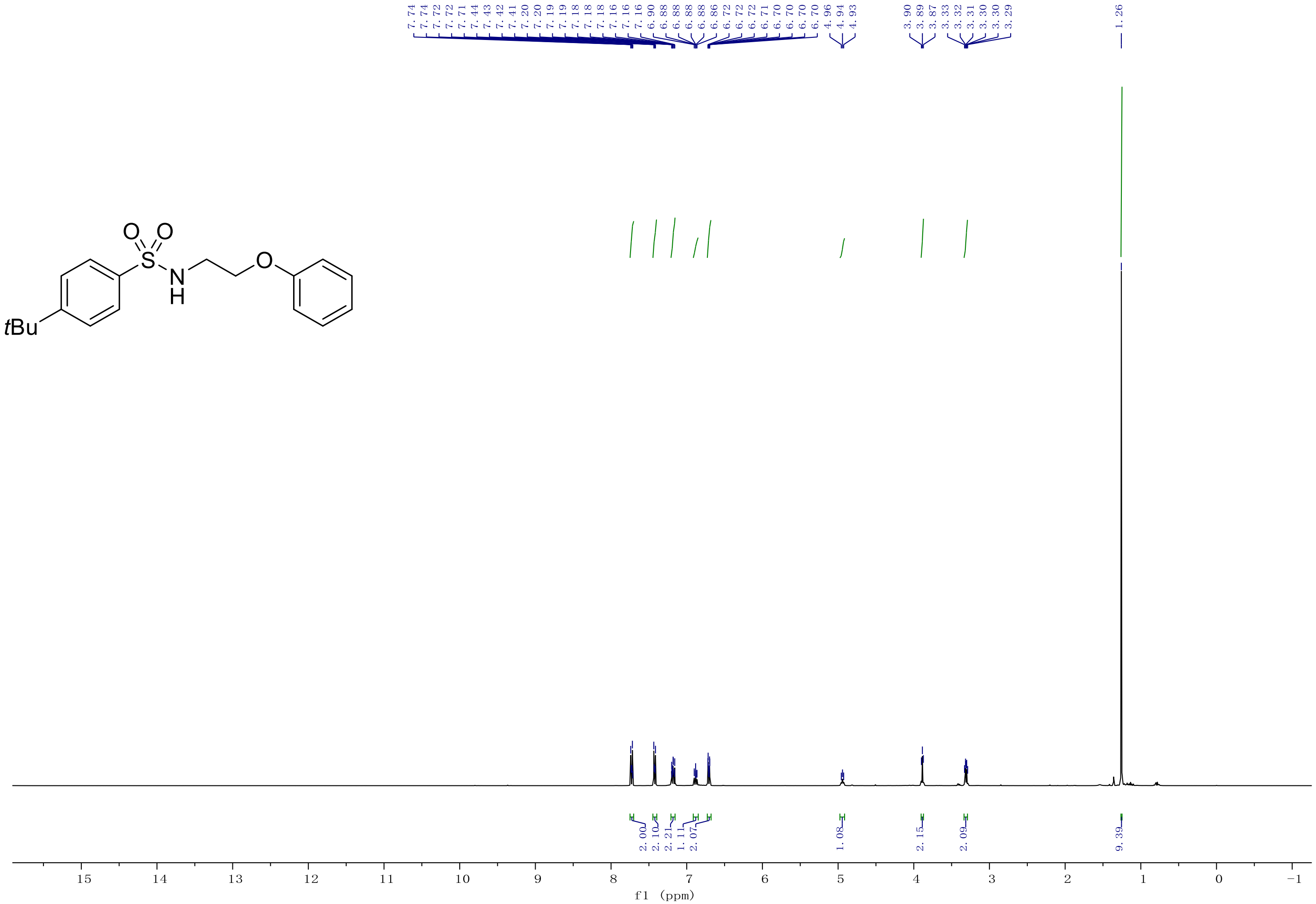

^{1}H NMR spectrum of **4-(*tert*-butyl)-*N*-(2-phenoxyethyl)benzenesulfonamide 6o**

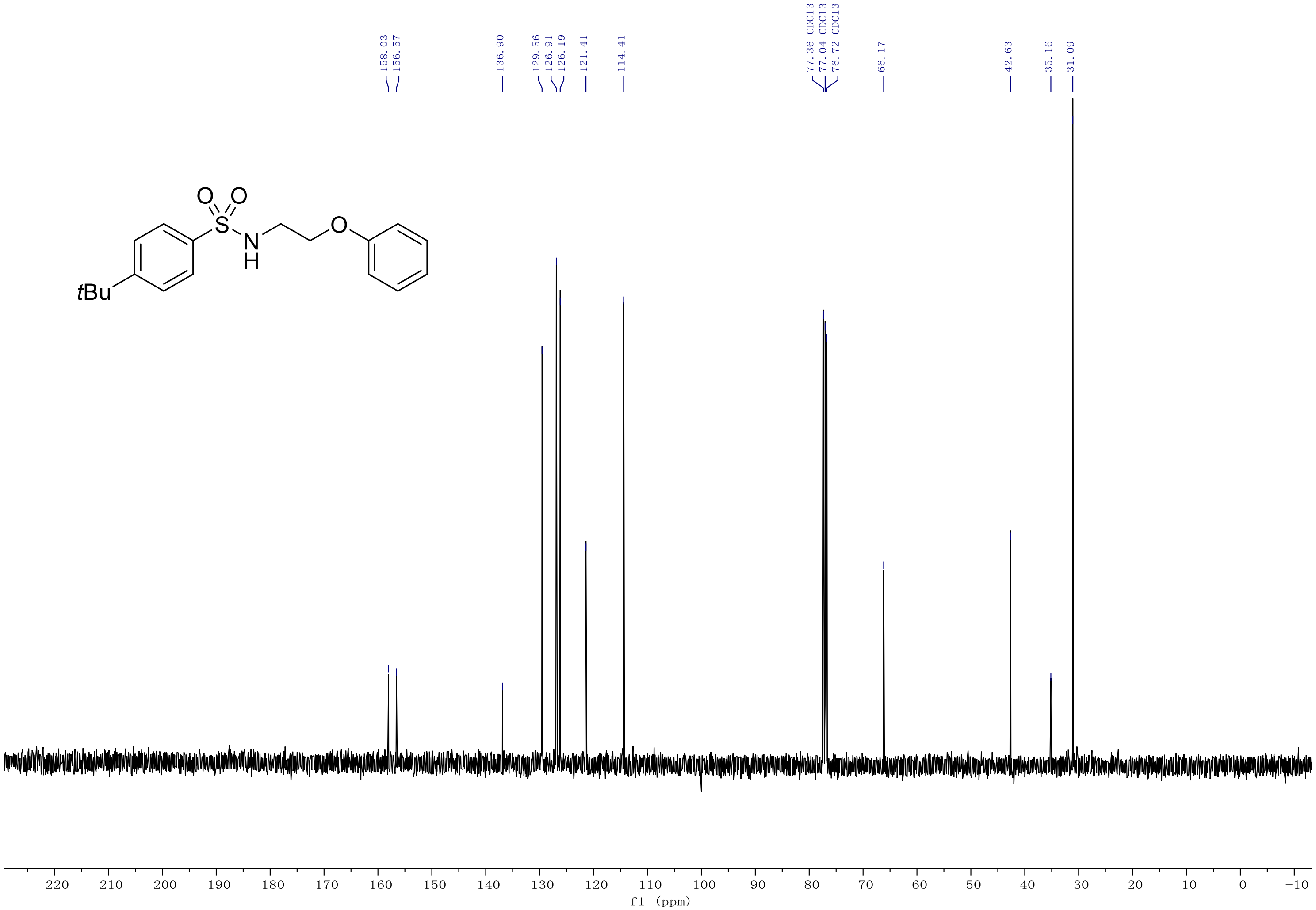

^{13}C NMR spectrum of **4-(*tert*-butyl)-*N*-(2-phenoxyethyl)benzenesulfonamide 6o**

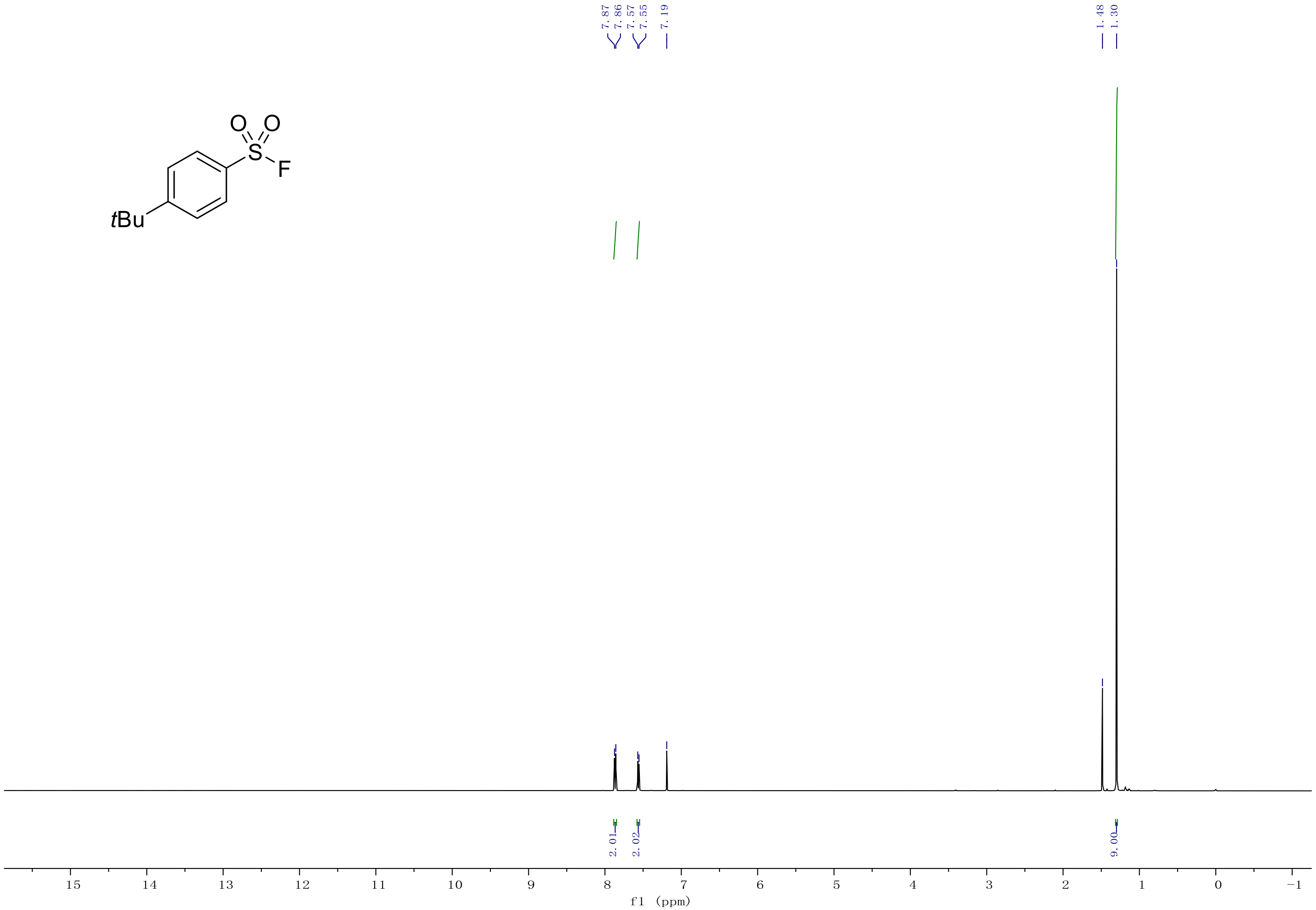

^{1}H NMR spectrum of **4-(*tert*-butyl)benzenesulfonyl fluoride 6p**

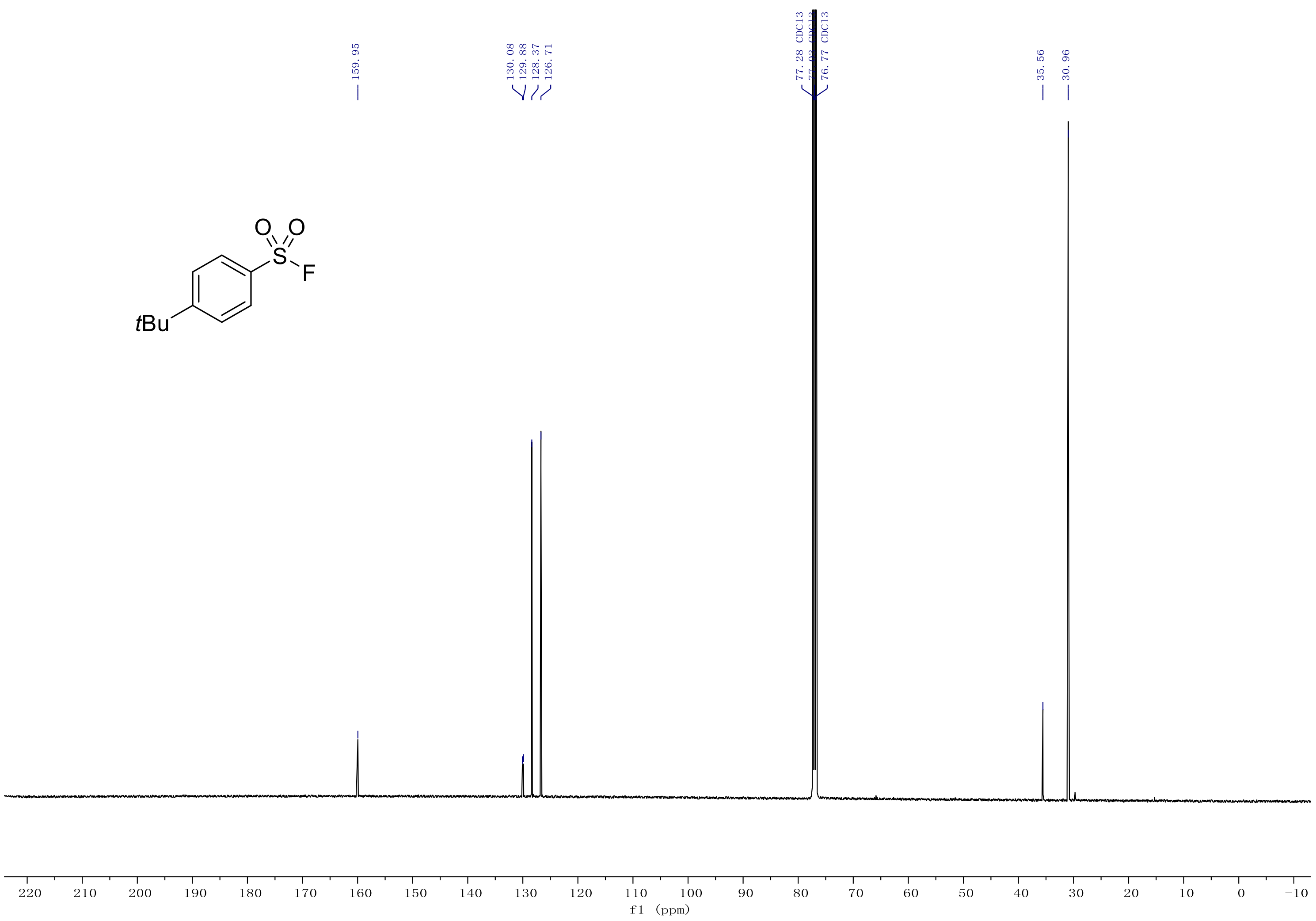

^{13}C NMR spectrum of **4-(*tert*-butyl)benzenesulfonyl fluoride 6p**

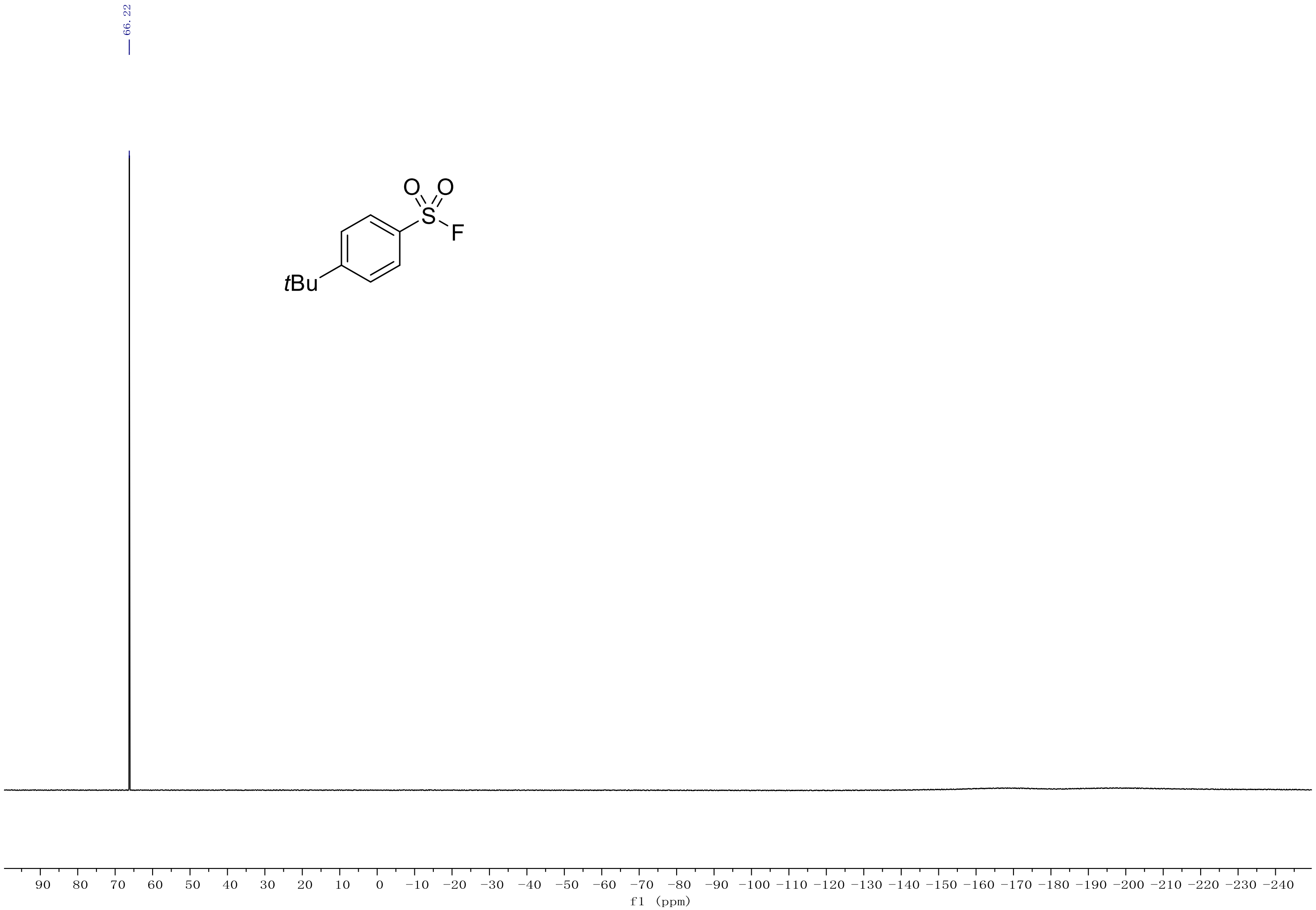

^{19}F NMR spectrum of **4-(*tert*-butyl)benzenesulfonyl fluoride 6p**

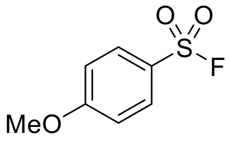

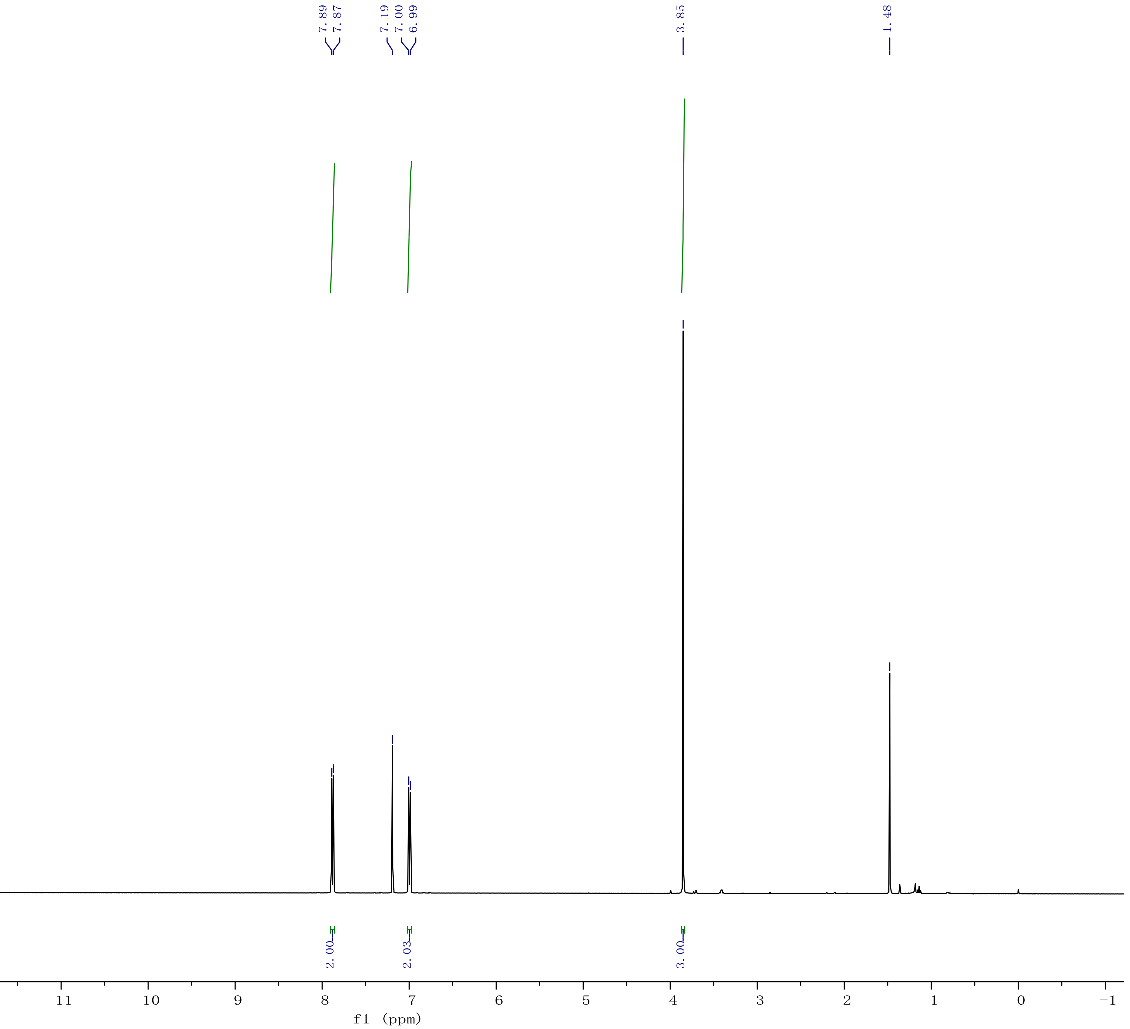

^{1}H NMR spectrum of **4-methoxybenzenesulfonyl fluoride 6q**

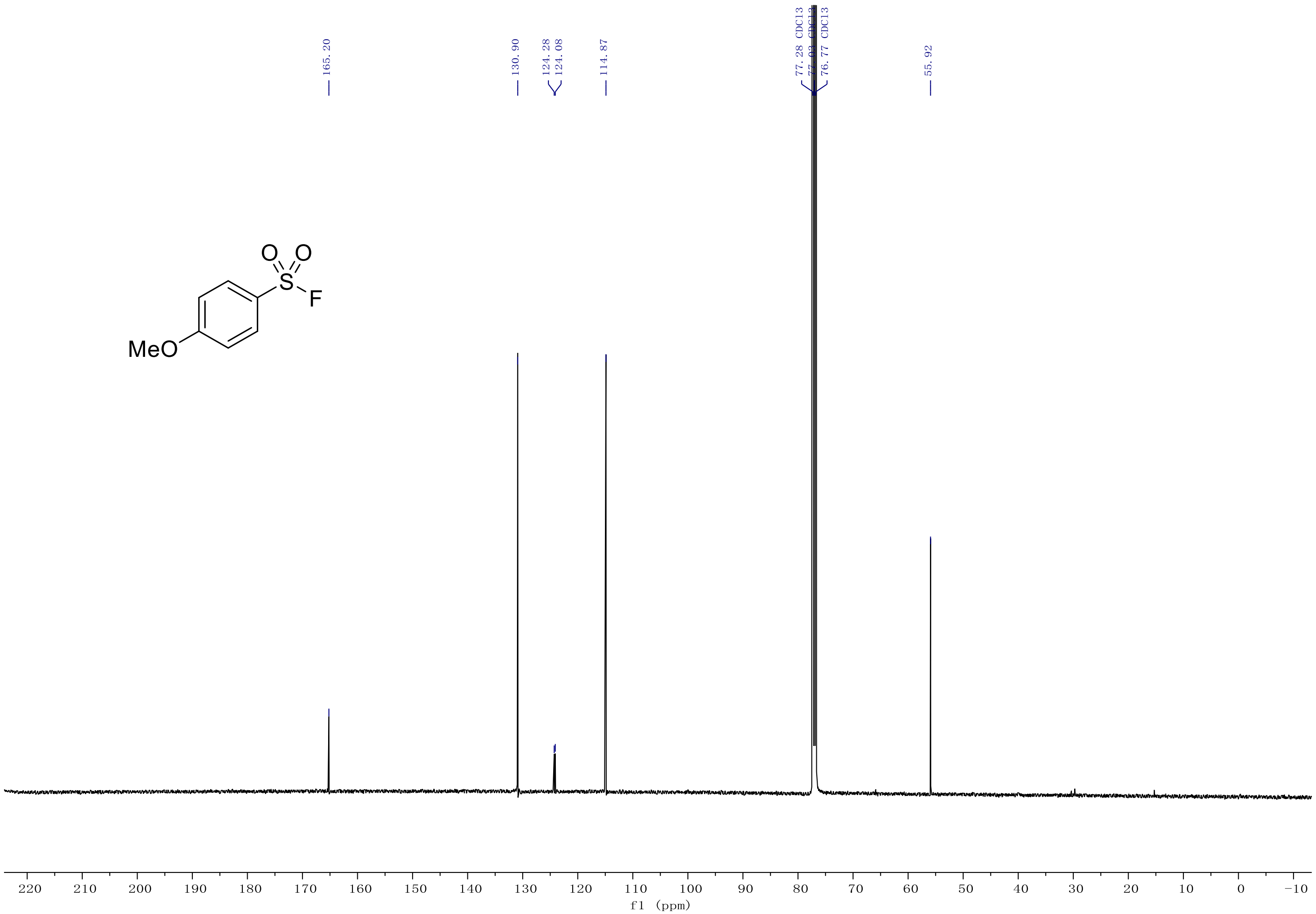

^{1}H NMR and ^{13}C NMR spectra of **4-methoxybenzenesulfonyl fluoride 6q**

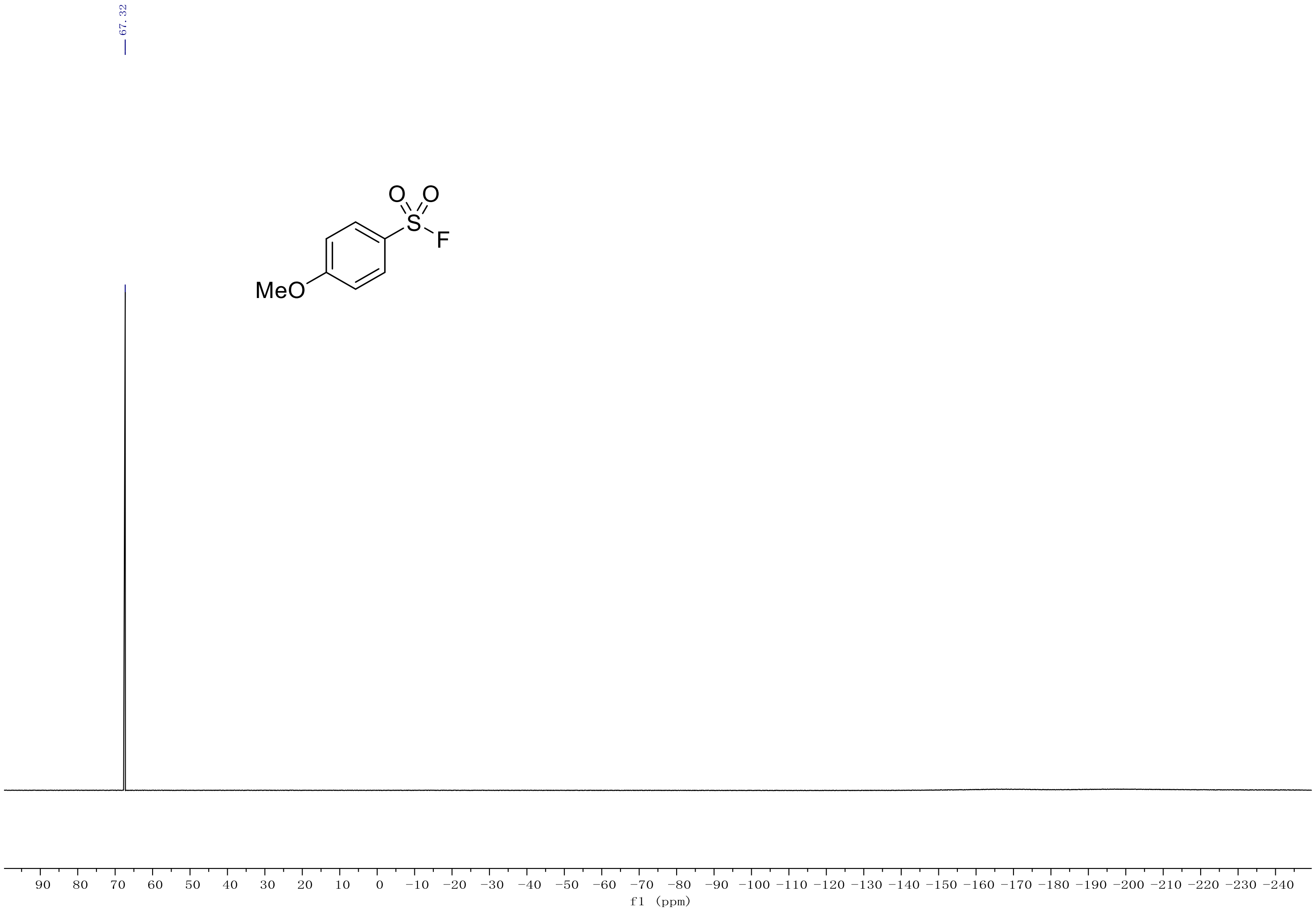

^{19}F NMR spectrum of **4-methoxybenzenesulfonyl fluoride 6q**

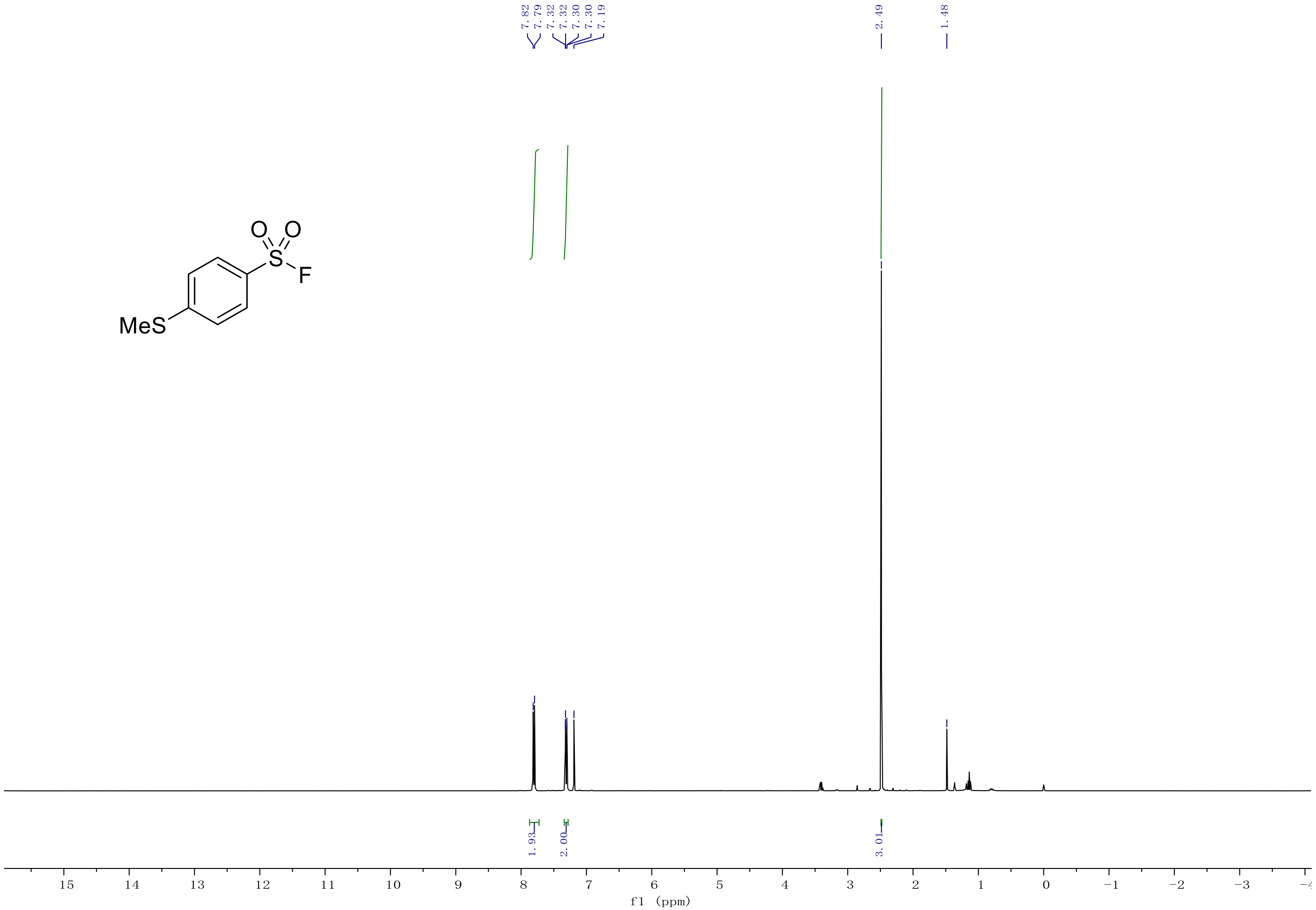

^{1}H NMR spectrum of **4-methylthiobenzenesulfonyl fluoride 6r**

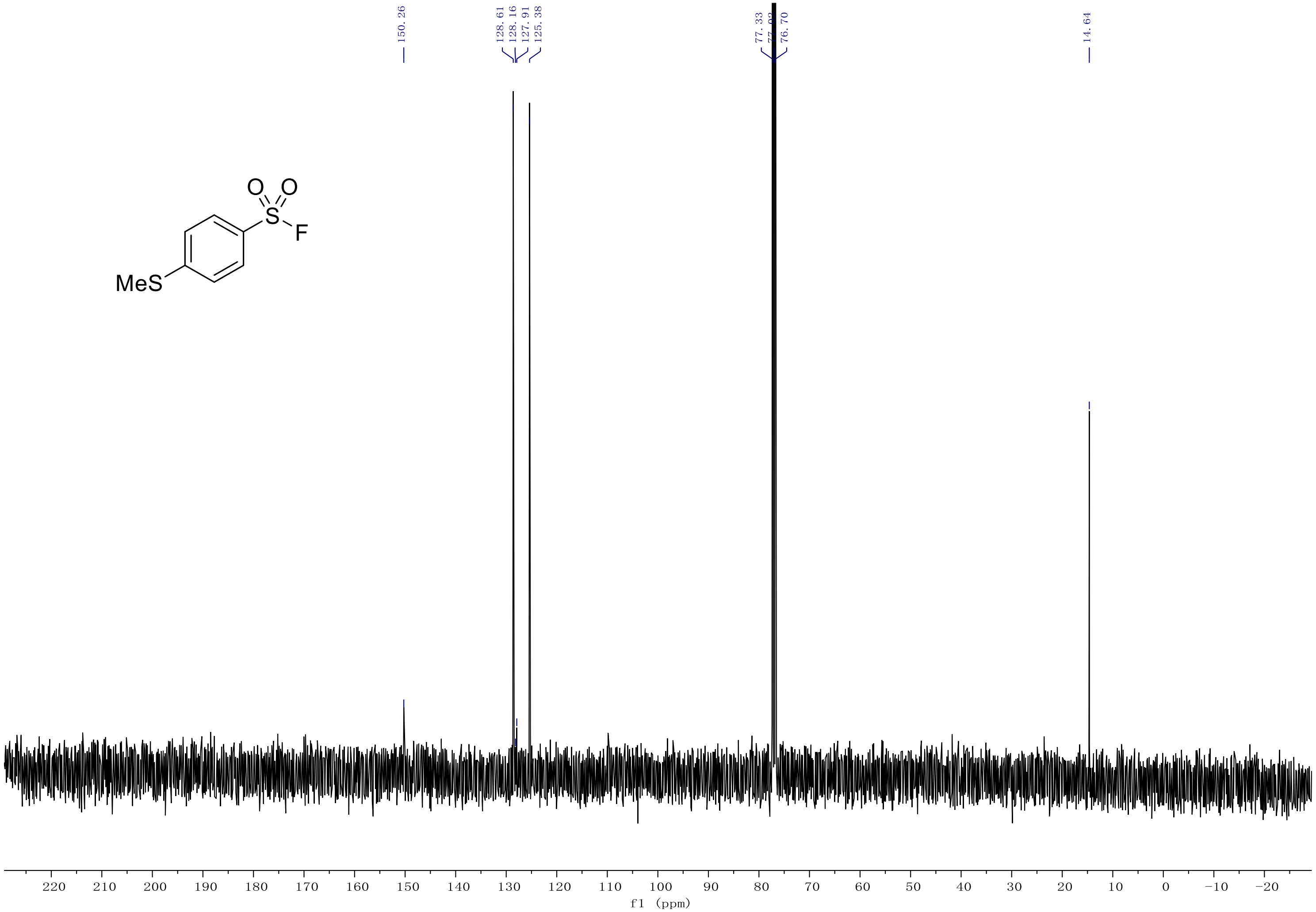

^{13}C NMR spectrum of **4-methylthiobenzenesulfonyl fluoride 6r**

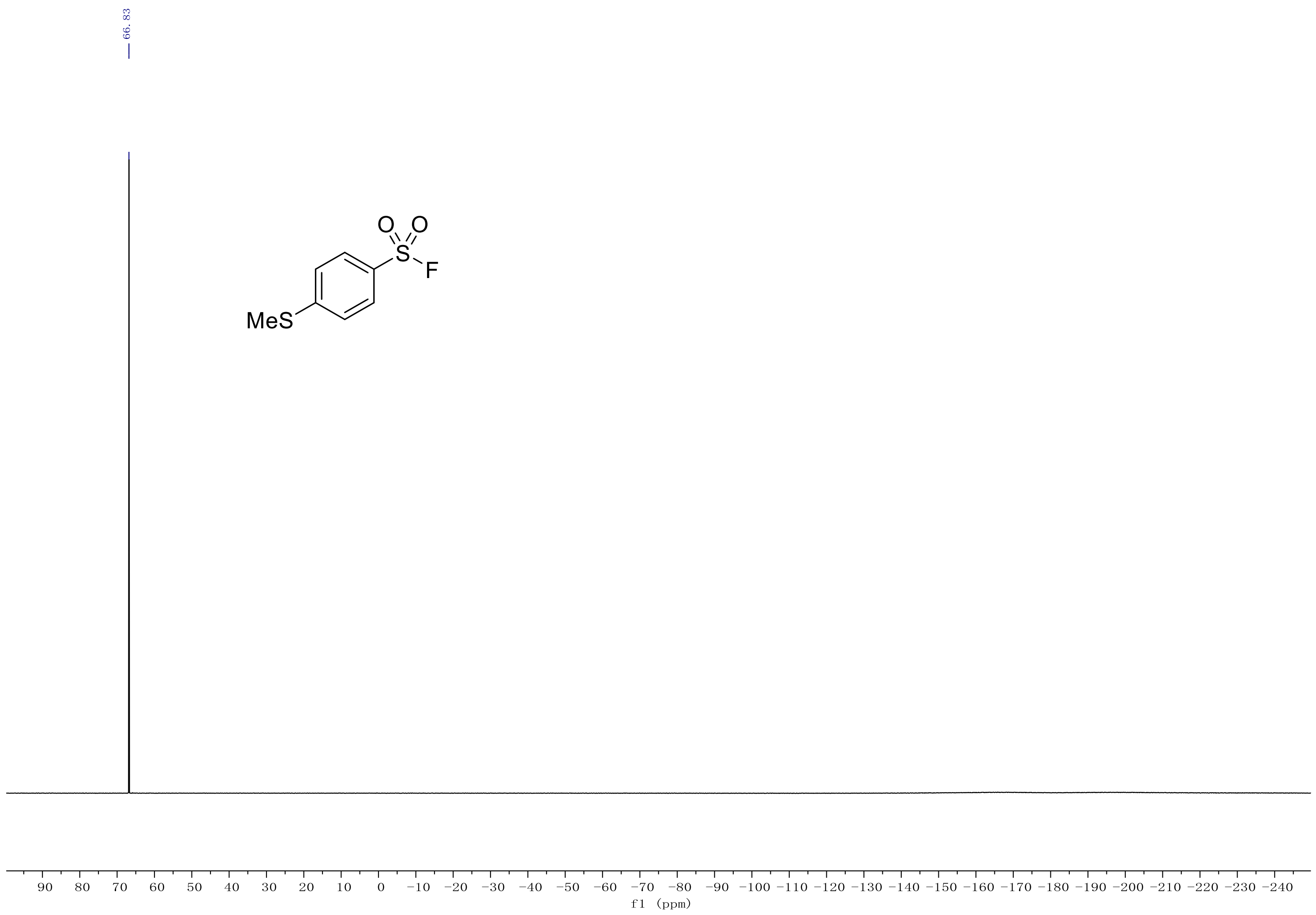

^{19}F NMR spectrum of **4-methylthiobenzenesulfonyl fluoride 6r**

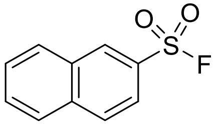

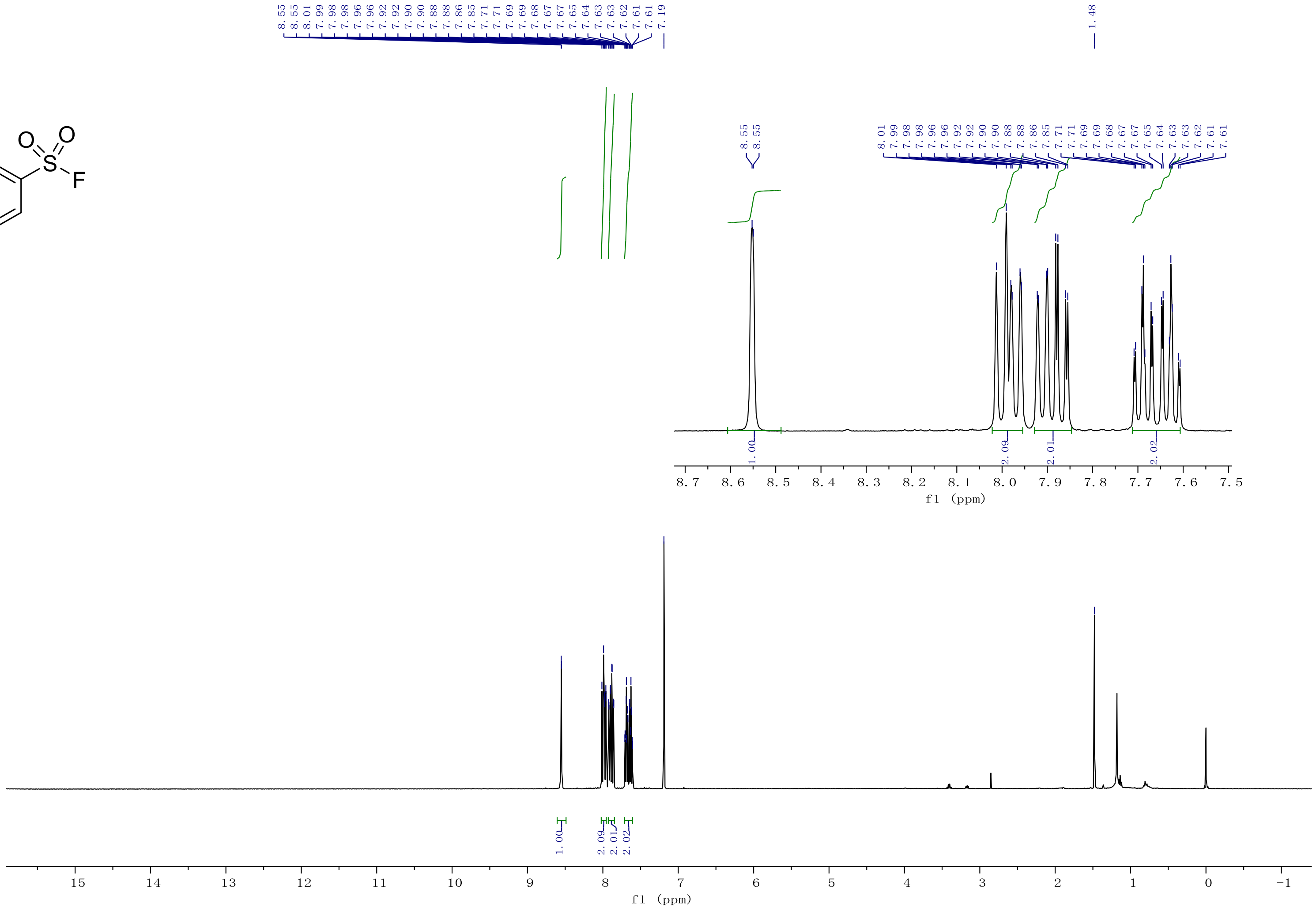

^{1}H NMR spectrum of **naphthalene-2-sulfonyl fluoride 6s**

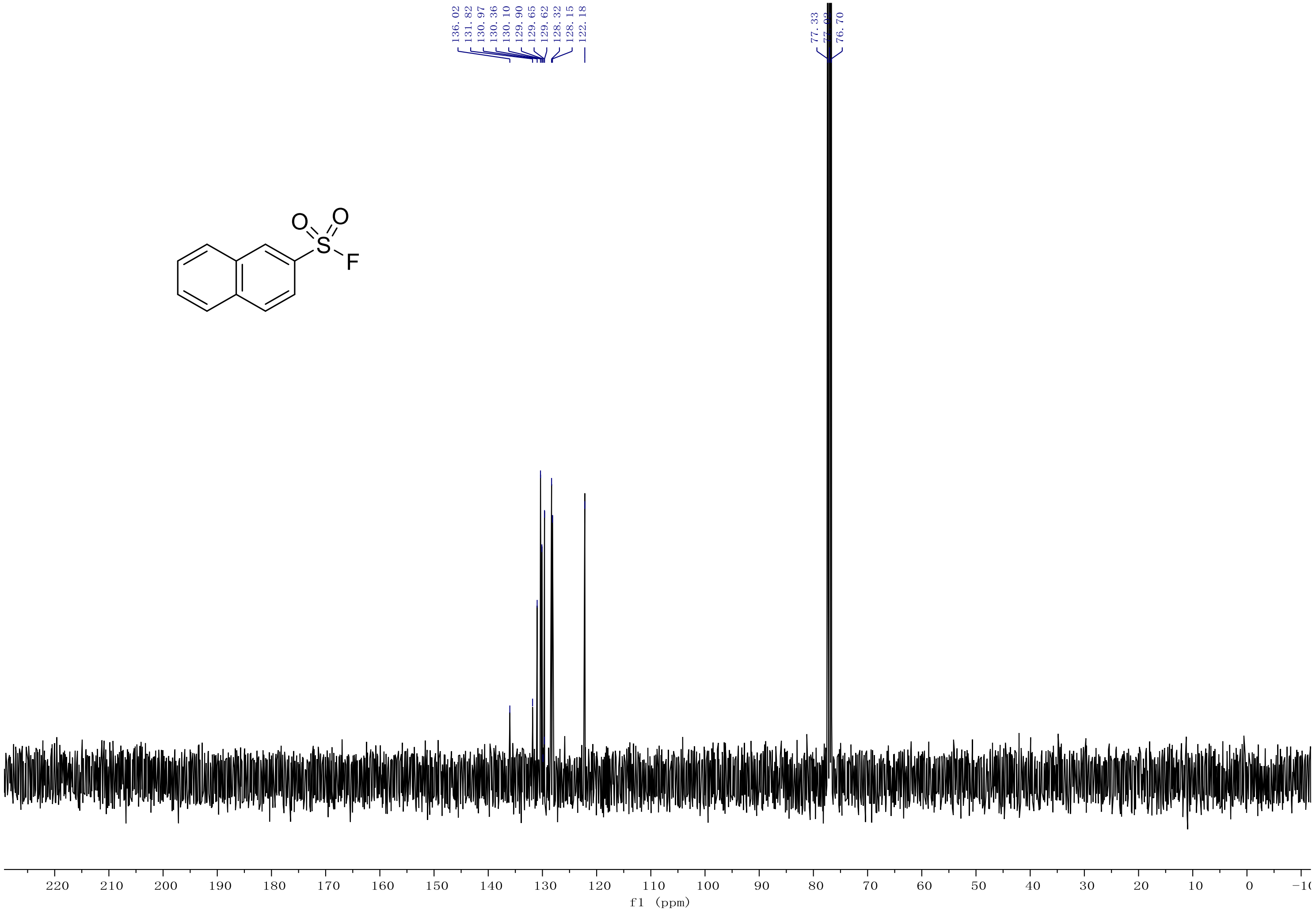

^{13}C NMR spectrum of **naphthalene-2-sulfonyl fluoride 6s**

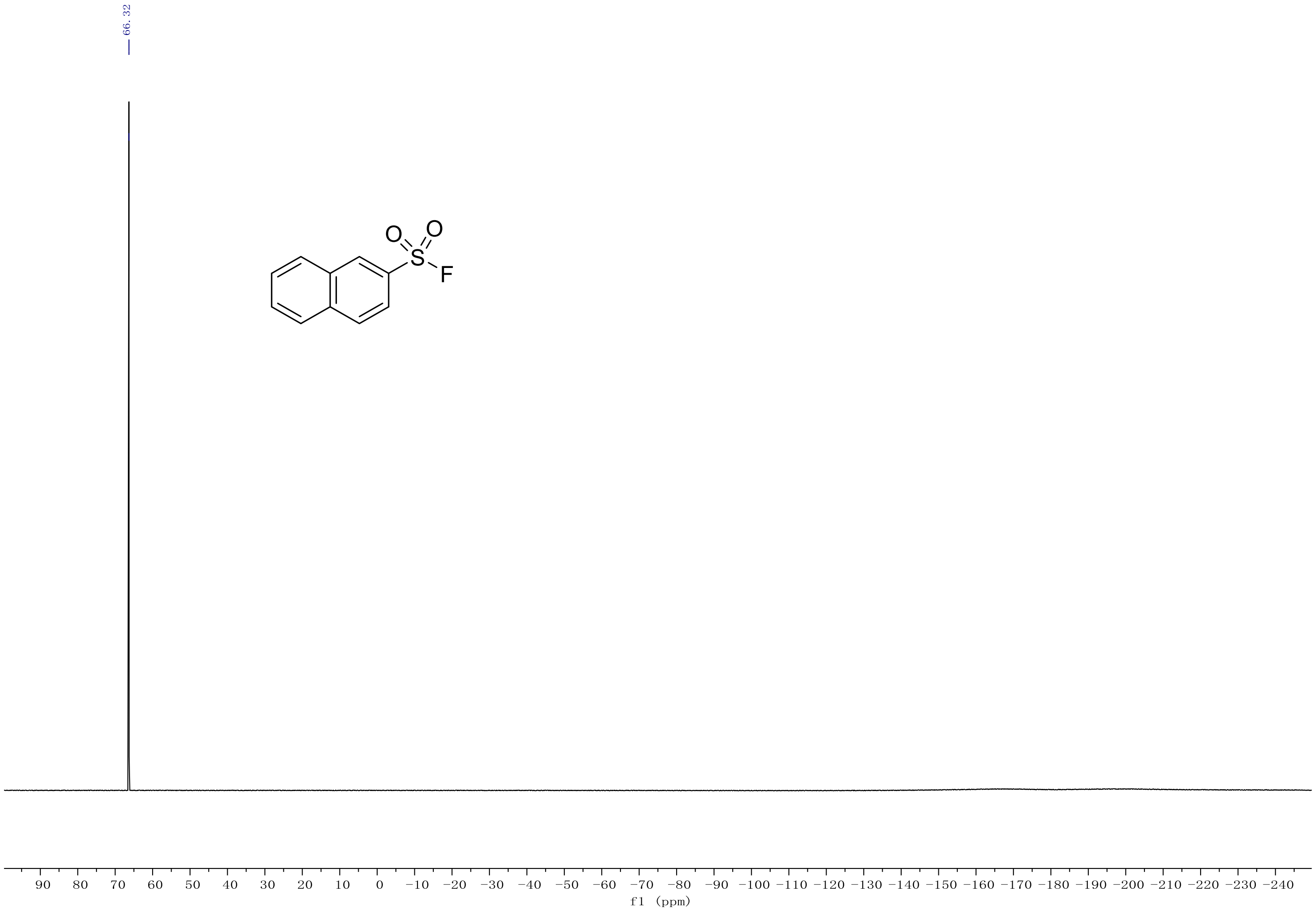

^{19}F NMR spectrum of **naphthalene-2-sulfonyl fluoride 6s**

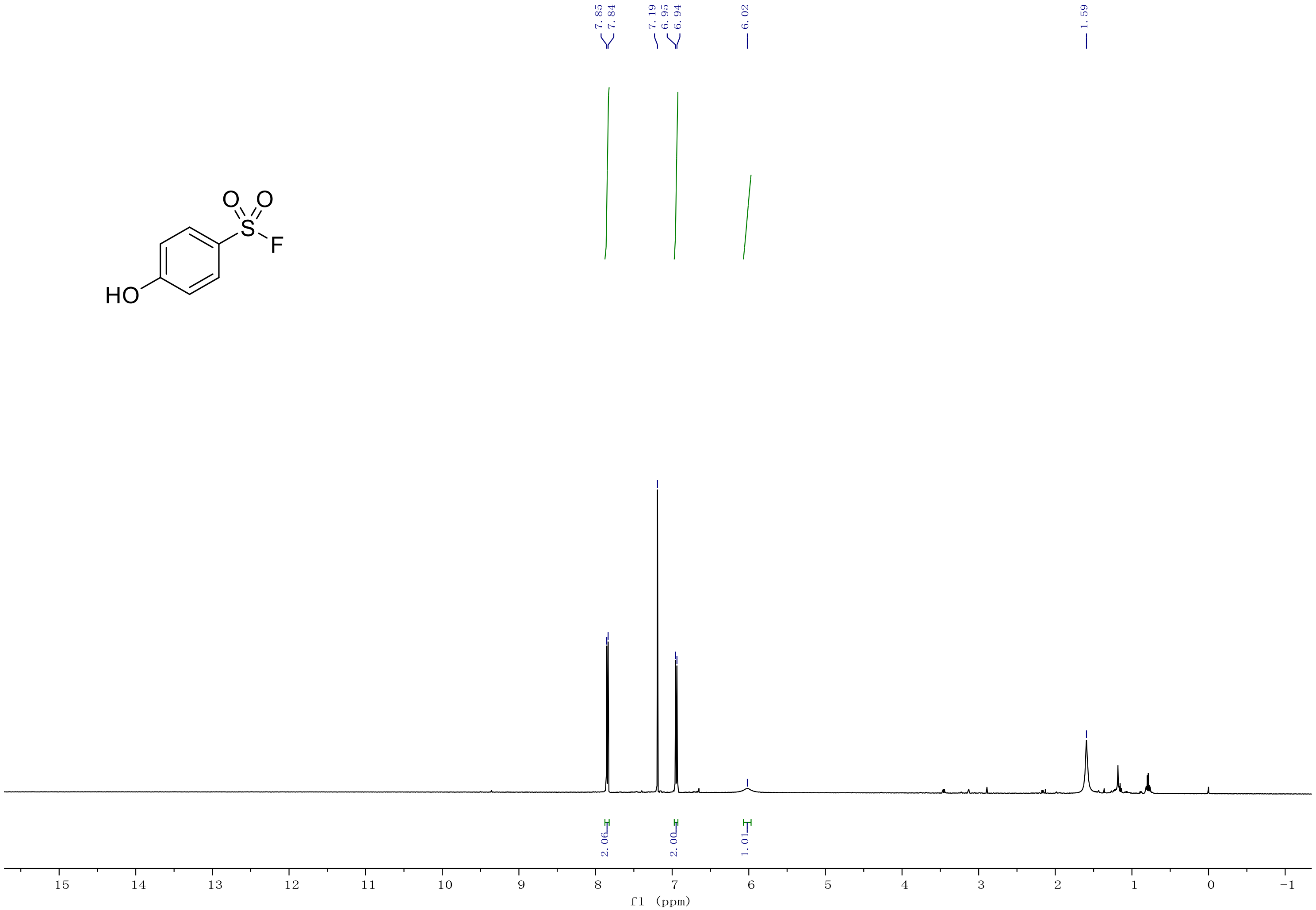

^{1}H NMR and ^{13}C NMR spectra of **4-hydroxybenzenesulfonyl fluoride 6t**

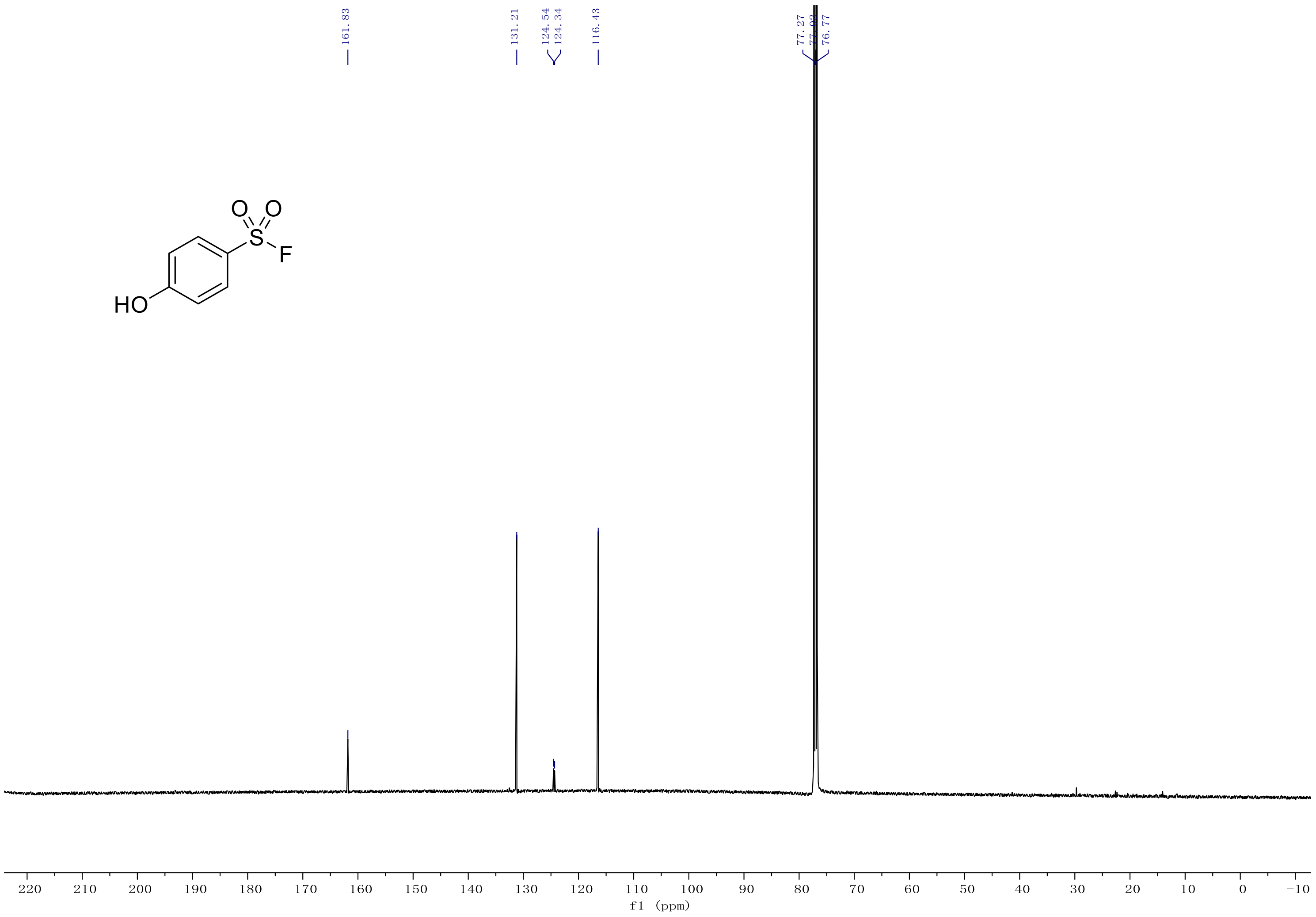

^{13}C NMR spectrum of **4-hydroxybenzenesulfonyl fluoride 6t**

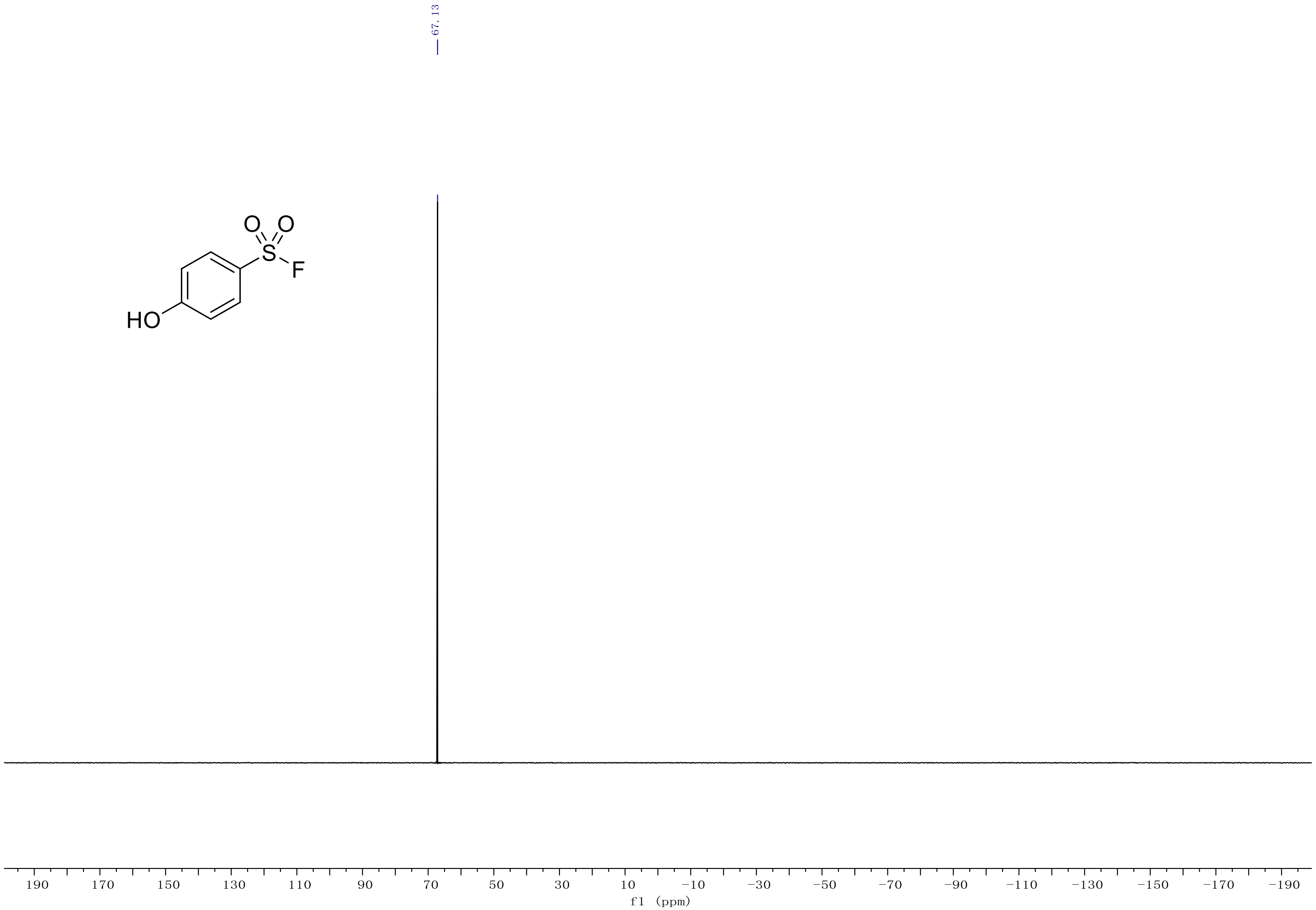

^{19}F NMR spectrum of **4-hydroxybenzenesulfonyl fluoride 6t**
